# Light-responsive expression atlas reveals the effects of light quality and intensity in *Kalanchoë fedtschenkoi*, a plant with crassulacean acid metabolism

**DOI:** 10.1093/gigascience/giaa018

**Published:** 2020-03-05

**Authors:** Jin Zhang, Rongbin Hu, Avinash Sreedasyam, Travis M Garcia, Anna Lipzen, Mei Wang, Pradeep Yerramsetty, Degao Liu, Vivian Ng, Jeremy Schmutz, John C Cushman, Anne M Borland, Asher Pasha, Nicholas J Provart, Jin-Gui Chen, Wellington Muchero, Gerald A Tuskan, Xiaohan Yang

**Affiliations:** 1 Biosciences Division, Oak Ridge National Laboratory, 1 Bethel Valley Rd, Oak Ridge, TN 37831, USA; 2 The Center for Bioenergy Innovation, Oak Ridge National Laboratory, 1 Bethel Valley Rd, Oak Ridge, TN 37831, USA; 3 HudsonAlpha Institute for Biotechnology, 601 Genome Way, Huntsville, AL 35801, USA; 4 Department of Biochemistry and Molecular Biology, University of Nevada, 1664 N. Virginia St, Reno, NV 89557, USA; 5 US Department of Energy Joint Genome Institute, Lawrence Berkeley National Laboratory, 1 Cyclotron Road, Berkeley, CA 94720, USA; 6 School of Natural and Environmental Science, Newcastle University, Newcastle upon Tyne NE1 7RU, UK; 7 Department of Cell and Systems Biology, Centre for the Analysis of Genome Evolution and Function, University of Toronto, 25 Willcocks St #4038, Toronto, ON M5S 3B2, Canada

**Keywords:** eFP browser, gene atlas, transcriptome, *Kalanchoë fedtschenkoi*, crassulacean acid metabolism

## Abstract

**Background:**

Crassulacean acid metabolism (CAM), a specialized mode of photosynthesis, enables plant adaptation to water-limited environments and improves photosynthetic efficiency via an inorganic carbon-concentrating mechanism. *Kalanchoë fedtschenkoi* is an obligate CAM model featuring a relatively small genome and easy stable transformation. However, the molecular responses to light quality and intensity in CAM plants remain understudied.

**Results:**

Here we present a genome-wide expression atlas of *K. fedtschenkoi* plants grown under 12 h/12 h photoperiod with different light quality (blue, red, far-red, white light) and intensity (0, 150, 440, and 1,000 μmol m^–2^ s^–1^) based on RNA sequencing performed for mature leaf samples collected at dawn (2 h before the light period) and dusk (2 h before the dark period). An eFP web browser was created for easy access of the gene expression data. Based on the expression atlas, we constructed a light-responsive co-expression network to reveal the potential regulatory relationships in *K. fedtschenkoi*. Measurements of leaf titratable acidity, soluble sugar, and starch turnover provided metabolic indicators of the magnitude of CAM under the different light treatments and were used to provide biological context for the expression dataset. Furthermore, CAM-related subnetworks were highlighted to showcase genes relevant to CAM pathway, circadian clock, and stomatal movement. In comparison with white light, monochrome blue/red/far-red light treatments repressed the expression of several CAM-related genes at dusk, along with a major reduction in acid accumulation. Increasing light intensity from an intermediate level (440 μmol m^−2^ s^−1^) of white light to a high light treatment (1,000 μmol m^–2^ s^–1^) increased expression of several genes involved in dark CO_2_ fixation and malate transport at dawn, along with an increase in organic acid accumulation.

**Conclusions:**

This study provides a useful genomics resource for investigating the molecular mechanism underlying the light regulation of physiology and metabolism in CAM plants. Our results support the hypothesis that both light intensity and light quality can modulate the CAM pathway through regulation of CAM-related genes in *K. fedtschenkoi*.

## Background

Sunlight is a critical energy resource for plant growth and development, and it functions as an important input signal for the circadian clock, stomatal movement, and photosynthesis pathway. The light spectra that affect plant photosynthesis are UV-A/blue, red, and far-red [1, 2]. Blue light (BL), with wavelength of 400–500 nm, has a higher energy than red light (RL) (wavelength of 600–700 nm) and far-red light (FRL) (wavelength >700 nm) [1, 3]. There are 3 types of photoreceptors (i.e., cryptochromes, phototropins, and phytochromes) that perform important roles in plant light response [2, 4]. Cryptochromes and phototropins have been identified as important photoreceptors of UV-A/blue light [2, 5, 6]. Phytochromes are known to play a role in detecting red and far-red spectra [2, 7]. In addition to the light quality, light intensity is another essential factor that affects plant growth and development, where either too much or little light can cause stress, including serious damage to photosynthetic apparatus under excess light exposure and limited photosynthetic activity with insufficient light input [8–10].

Plants using the crassulacean acid metabolism (CAM) pathway for photosynthesis show enhanced water-use efficiency and heat/drought stress tolerance in comparison with C_3_ and C_4_ photosynthesis plants [11, 12]. The CAM pathway has 2 major features: (i) a carboxylation process that takes place at night where stomata are open for nocturnal CO_2_ fixation and accumulation of malic acid in the vacuole and (ii) a decarboxylation process that occurs during the daytime where CO_2_ is released from malate for refixation via ribulose-1,5-bisphosphate carboxylase/oxygenase (Rubisco)-mediated photosynthesis, along with stomatal closure, which reduces evapotranspiration [11, 13, 14]. *Kalanchoë fedtschenkoi* is a model eudicot CAM species, featuring a relatively small genome and a facile stable transformation system [11, 15]. The genome of *K. fedtschenkoi* was recently sequenced and annotated [13], providing a foundation for CAM genomics research. Comparative and evolutionary genomics analyses revealed convergent signatures in diel gene expression pattern and protein sequences underlying independent emergences of CAM from the C_3_ ancestor, providing new insights into CAM evolution [13]. However, the complex regulatory mechanisms that underpin the diel optimization of the CAM pathway under various light conditions remain largely unexplored.

The temporal separation of C_3_ and C_4_ carboxylation processes that defines CAM provides plasticity for optimizing carbon gain and water use in response to changing environmental conditions by extending or curtailing the period of net CO_2_ uptake over a 24-h period [16]. Light intensity (photosynthetic photon flux density) and light quality are critical factors for controlling the performance of CAM, which implies cardinal roles for the light reactions of photosynthesis and for different photoreceptors in achieving metabolic and circadian synchronization of carboxylation processes across the diel cycle. In some facultative CAM species, high light intensity can trigger the switch from C_3_ photosynthesis to CAM, which is mediated by a UV-A/blue light receptor [17]. Metabolic and physiological adaptation in constitutive CAM species plants to light quantity and quality has been reported previously [18–21]. For instance, physiological and metabolic responses under severe light stress under short- and long-term treatments were reported [18]. Metabolic changes under different light spectra (i.e., blue, green, and red light) were also reported in the obligate CAM species *Aechmea “Maya”* [19]. Exposure of the woody CAM species *Clusia hilariana* to low light (LL) or high light (HL) affected the production of malate and citrate [21]. However, the molecular basis of the metabolic and signaling pathways that underpin changes to the operation of CAM in response to various spectral light qualities and intensity has not been investigated extensively.

High-throughput, next-generation sequencing has been widely applied to genome-wide expression analysis. As a new type of web-based tool, a genome-wide atlas of gene expression can provide comprehensive gene expression profiles in different tissues or different development stages. To date, gene expression atlases have been established for several C_3_ photosynthesis species, including eudicot *Arabidopsis* [22], *Medicago* [23], tomato [24], monocot wheat [25], and *Brachypodium* [26]. However, a genome-wide expression atlas has not been created for a CAM species. In addition, gene expression patterns and gene modules associated with plant response to light quality and light intensity are largely unknown, especially for CAM plants.

We hypothesize that both light intensity and light quality can influence the expression of CAM-related metabolic and signaling genes in the obligate CAM species *K. fedtschenkoi*. To test this hypothesis, we performed transcriptome sequencing (RNA-Seq) of mature *K. fedtschenkoi* leaf samples collected at dawn (i.e., 2 h before the light period) and dusk (2 h before the dark period) from plants grown under 12 h/12 h photoperiod with different light quality (i.e., BL, RL, FRL, white light [WL]) and intensity (0, 150, 440, and 1,000 μmol m^–2^ s^–1^). On the basis of our analysis of the RNA-Seq data, we generated a comprehensive light-responsive gene expression atlas for this obligate CAM species. We also constructed a genome-wide co-expression network based on the light-responsive gene expression atlas. To provide a metabolic context for the genome-wide co-expression network, we measured the titratable acidity (nocturnal malate and citrate accumulation) and soluble sugar and starch turnover. Nocturnal acid accumulation provides a quantitative measure of CAM activity while diel turnover of sugars and starch provides an indication of the nocturnal supply and daytime demand for carbon processing. The subnetworks of CAM-related genes, photoreceptors, and stomatal movement–related genes indicated that the light-responsive expression atlas provides molecular clues for various physiological phenotypes. As a comprehensive light-responsive gene atlas and co-expression network for CAM plants, this study provides an unprecedented genomics resource for investigating molecular mechanisms underlying the light regulation of biological processes in CAM plants.

## Data Description

A total of 42 libraries (7 light conditions × 2 time points × 3 biological replicates) were constructed, and RNA-seq was performed independently. In total, we obtained 981 million read pairs (2 × 150 bp) with 287.78 Gb high-quality data (Q-score ≥ 25) from the 42 libraries, with a mean size of ∼23.4 million read pairs per library (Supplementary Table S1).

## Analyses

### Light-responsive expression atlas for *K. fedtschenkoi*

**Figure 1:**
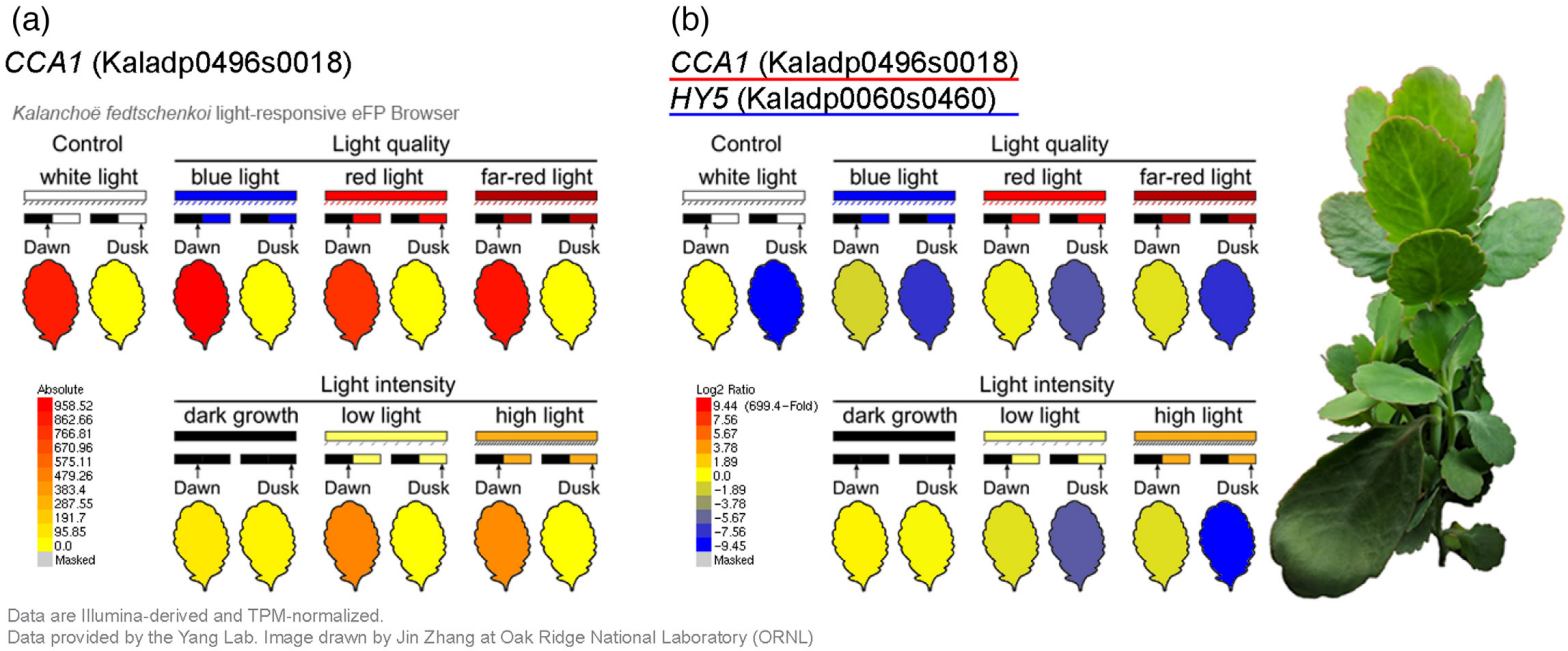
*Kalanchoë* light-responsive eFP browser. **(a)** View of the eFP browser including the RNA-Seq dataset described in this study. Expression values in the samples are indicated by a color gradient, where yellow indicates low expression and red indicates high expression. The legend describing the color gradient and expression values is shown in the bottom left corner. *CCA1* gene *Kaladp0496s0018* is used as an example. The leaf samples were collected at dawn (i.e., 2 h before the light period) and dusk (i.e., 2 h before the dark period) under control condition (white light) and various light quality conditions (blue, red, and far-red light) and light intensity conditions (dark growth, low light intensity, and high light intensity). TPM: transcripts per million.**(b)***Kalanchoë* genes *CCA1* (*Kaladp0496s0018*) and *HY5* (*Kaladp0060s0460*) displayed in a comparative view of the expression level extracted from the eFP browser. The legend describing the color gradient and log_2_ ratio is shown in the bottom left corner.

To obtain a comprehensive light-responsive gene expression atlas of the CAM plant *K. fedtschenkoi*, we treated plants under a control condition (WL with 440 μmol m^−2^ s^−1^ intensity) and various light quality including BL, RL, FRL, and different light intensities, including dark grown (DG), LL, and HL (Supplementary Table S1). Because the CAM pathway is likely regulated by the circadian clock, we investigated whether circadian rhythm–related processes were also affected by different light conditions. The samples were collected at 2 time points (dawn [2 h before light period] and dusk [2 h before dark period]) for each light condition. To provide easy access to the expression data, we created a *Kalanchoë* light-responsive eFP browser [27], which provides a color-coding tissue visualization in an image corresponding to the average gene expression level (Fig. 1).

**Figure 2:**
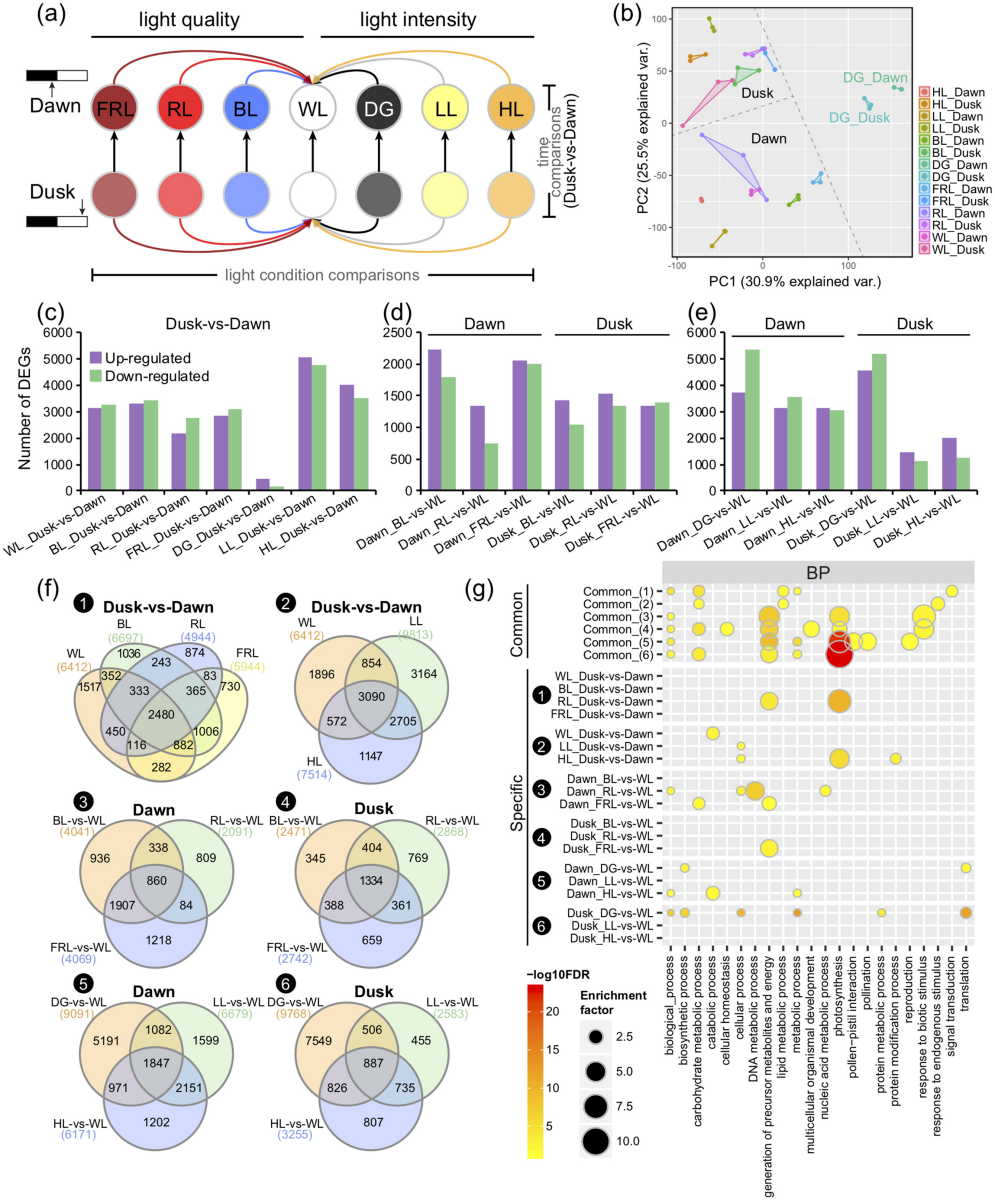
Transcriptomic comparison of *Kalanchoë fedtschenkoi* under various light quality and light intensity conditions. **(a)** Schematic of sample collection and comparisons. The leaf samples were collected at dawn (2 h before the light period) and dusk (2 h before the dark period) under control condition (white light [WL]) and various light quality conditions (blue light [BL], red light [RL], and far-red light [FRL]) and light intensity conditions (dark grown [DG], low light intensity [LL], and high light intensity [HL]). For differentially expressed gene (DEG) identification, the comparisons were classified into time comparisons (dusk vs dawn) and light condition comparisons (BL/RL/FRL vs WL for light quality comparisons and DG/LL/HL vs WL for light intensity comparisons). **(b)** Principal component analysis (PCA) of the 14 groups of transcriptome data. **(c–e)** Statistic of DEGs between dawn and dusk in time comparisons **(c)** and among various quality **(d)** or intensity **(e)** in light condition comparisons. **(f)** Venn diagrams represent DEGs overlapped in different comparisons. (1) dusk vs dawn under different light quality; (2) dusk vs dawn under different light intensity; (3) different light quality (BL/RL/FRL vs WL) at dawn; (4) different light quality at dusk; (5) different light intensity (DG/LL/HL vs WL) at dawn; (6) different light intensity at dusk. **(g)** Gene ontology (GO) enrichment of common DEGs shared by different comparisons in (f) Venn diagrams (DEGs shared by all the comparisons in a given set) or specific DEGs (genes only differentially expressed in 1 of the overlapped comparisons). BP: Biological process. Detailed enrichment of molecular function (MF) and cellular component (CC) is shown in Supplementary Fig. S3. GOslim terms are shown here.

The Pearson correlation analysis and principal component analysis showed that the biological replicates of each treatment group were closely clustered, indicating the high reproducibility and reliability of our RNA-seq data (Fig. 2 and Supplementary Fig. S1). The principal component 1 (PC1) and PC2 explained 30.9% and 25.5% of the variance in the expression data, respectively. As expected, the samples collected at dawn and dusk were grouped separately under different light quality and light intensity except DG (Fig. 2b), and the expression variation of samples under various light conditions was stronger at dawn than at dusk.

### Differentially expressed genes regulated by light quality and light intensity

As shown in Fig. 2a, we performed a comparative transcriptomic analysis for screening of differentially expressed genes (DEGs) by using 2 different strategies (i.e., time comparison and light condition comparison). The time comparison was defined as the comparison between 2 samples collected at 2 different time points (i.e., dawn and dusk) under each light condition. The light condition comparison reflected a comparison between treatments and control at the same sample collection time point (i.e., BL/RL/FRL vs WL) at dawn or dusk for light quality (i.e., DG/LL/HL vs WL) and at dawn or dusk for light intensities, respectively (Fig.   2a and Supplementary Table S2).

Under normal light condition (WL), 6,412 DEGs were identified in dusk vs dawn. Of these DEGs, 3,137 and 3,275 genes were revealed to be either up- or down-regulated, respectively (Fig. 2). For different light intensity, both the low- and high-intensity light treatments enhanced the differential gene expression between dusk and dawn. Under the DG condition, only 631 DEGs (458 up-regulated and 173 down-regulated) were identified in dusk vs dawn comparison, which was significantly less than the comparisons made under the other light conditions (Fig. 2 and Supplementary Table S2).

Under the different light qualities, a total of 2,480 DEGs between dusk and dawn were shared by the 4 light spectra (i.e., WL, BL, RF, and FRL), indicating that these genes might play essential roles in response to changes in light quality (Fig. 2f). Under various light intensity conditions, 3,090 genes were consistently differentially expressed under the 3 light intensities (WL, LL, and HL), suggesting that these genes might play key roles in responding to diel or circadian cues and were not affected by light intensity (Fig.   2f).

Light condition comparisons were based on differences between different light quality or light intensity (Fig. 2a). The DEG number in most light-quality comparisons was greater at dawn than those at dusk (Fig. 2d). However, the overlapped DEGs were fewer at dawn (860 common DEGs, Fig. 2f) than at dusk (1,334 common DEGs, Fig. 2f) under the different light quality treatments. In contrast, the light quality–specific DEGs were greater at dawn than at dusk under BL and FRL. For the different light intensities, more than half of the DEGs under LL and HL were shared at both dawn and dusk (Fig. 2f).

### Predicted function of DEGs

To explore the functional differences of DEGs induced by various light quality and light intensity treatments, we performed a gene ontology (GO) enrichment analysis of DEGs in different comparisons according to the 3 major GO categories of biological process (BP), molecular function (MF), and cellular component (CC) (Supplementary Fig. S2). Notably, all DEGs in the light intensity and light quality comparisons were enriched in “photosynthesis” process; but when the up-regulated or down-regulated DEGs were separated, only up-regulated genes in light quality comparisons and down-regulated genes in light intensity comparisons were highly enriched in “photosynthesis” process. Similarly, the “response to biotic stimulus” term in light quality for all DEGs was depleted (Supplementary Fig. S2 and Table S3).

In addition, we compared the enriched GO terms of common and light-specific DEGs in response to the light treatments (Fig. 2g, Supplementary Fig. S3 and Table S4). Among the DEGs between dusk and dawn, the 2,480 DEGs that overlapped across different light quality treatments (Fig. 2f) were enriched in “carbohydrate metabolic process”, “lipid metabolic process”, “metabolic process”, and “signal transduction”; whereas the 3,090 DEGs that overlapped across different light intensity treatments were enriched in “carbohydrate metabolic process” and “response to endogenous stimulus” (Fig.   2g). When comparing the common DEGs in different light quality or light intensity at dawn and dusk separately (Fig. 2f), we found that the “generation of precursor metabolites and energy” term was enriched in all 4 common DEG sets of different light quality and light intensity at both dawn and dusk. In contrast, the “photosynthesis” term was enriched in common DEGs of light quality at dusk and common DEGs of light intensity at both dawn and dusk (Fig. 2g), whereas the “carbohydrate metabolic process” term was only enriched at the dusk time point of different light quality and light intensity (Fig. 2g).

For condition-specific DEGs at different time points or different light conditions, the “photosynthesis” term was strongly enriched in dusk vs dawn DEGs in RL-specific and HL-specific from the light quality comparison and light intensity comparison, respectively (Fig. 2g). This indicates that RL and HL significantly affected photosynthesis-related changes in transcript abundance between dawn and dusk. When different light quality or light intensity treatments were compared to the WL control at dawn and dusk, the “photosynthesis” term was also enriched in the DG vs WL (Fig. 2f) and HL vs WL comparisons (Fig. 2g) at both dawn and dusk, indicating that HL and DG strongly affected photosynthesis-related changes in transcript abundance independent of dawn or dusk sampling.

**Figure 3:**
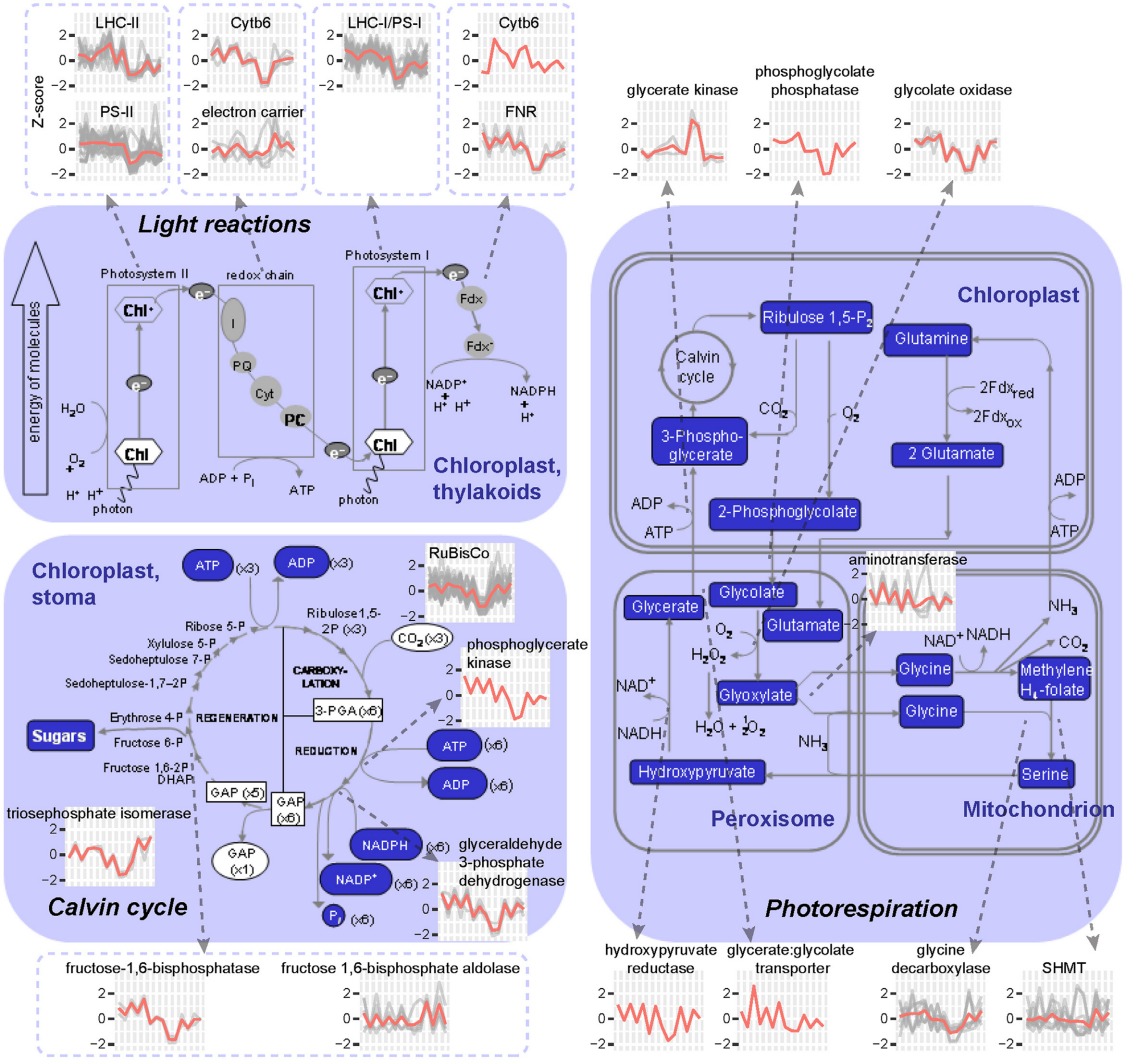
Schematic representation of gene expression patterns in the photosynthesis pathway. The figure was modified on the basis of the MapMan visualization platform. Line plots represent *Z*-score–normalized (*y*-axis) expression patterns of DEGs under various light quality and light intensity conditions. The *x*-axis from left to right represents the samples of WL_Dawn, WL_Dusk, BL_Dawn, BL_Dusk, RL_Dawn, RL_Dusk, FRL_Dawn, FRL_Dusk, DG_Dawn, DG_Dusk, LL_Dawn, LL_Dusk, HL_Dawn, and HL_Dusk. Detailed gene list and expression data are presented in Supplementary Table S4. ADP: adenosine diphosphate; ATP: adenosine triphosphate. Fdx: ferredoxin. GAP: Glyceraldehyde 3-phosphate. PGA: 3-phosphoglycerate.

To further understand the functional differences of DEGs under different light quality and intensity treatments, we then classified the DEGs into hierarchical categories “BINs” using MapMan. On the basis of the DEG number and percentage in each BIN, we found that the percentage of photosynthesis class genes was higher in the comparison “DG_Dusk-vs-Dawn” than the other comparisons (Supplementary Fig. S4). And the expression pattern of photosynthesis class genes showed down-regulation in samples of DG_Dawn and DG_Dusk (Supplementary Fig. S5). To better understand the gene expression patterns, we mapped the DEGs in the photosynthetic pathway. As shown in Fig. 3, most of the DEGs with functions involved in the light reactions (e.g., LHC-II, PS-II, Cytb6, LHC-I/PS-I, and FNR), Calvin cycle (e.g., Rubisco, phosphoglycerate kinase, glyceraldehyde 3-phosphate dehydrogenase, and fructose-1,6-bisphosphatase), and photorespiration (e.g., phosphoglycolate phosphatase, glycolate oxidase, and glycine decarboxylase) showed down-regulation in DG_Dawn and DG_Dusk. However, glycerate kinases in photorespiration were highly expressed in DG_Dawn and DG_Dusk.

### Co-expression network

**Figure 4:**
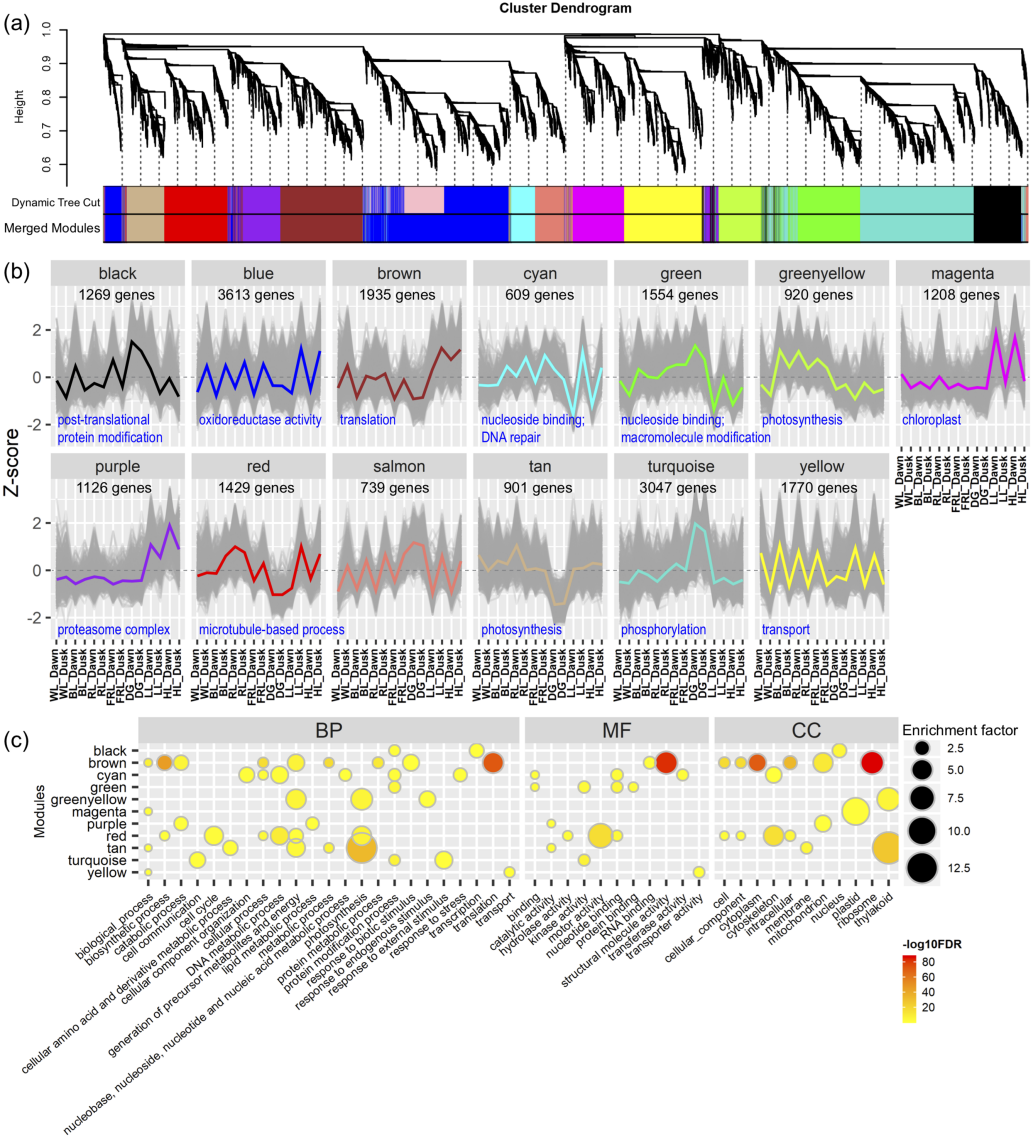
Weighted gene co-expression network analysis (WGCNA) of DEGs in *Kalanchoë fedtschenkoi* under various light quality and light intensity conditions. **(a)** Cluster dendrogram of DEGs in *Kalanchoë fedtschenkoi* under various light quality and light intensity conditions. Different colors in merged modules column represent 13 different modules (MEs). **(b)** Z-score–normalized expression patterns of DEGs in different modules. **(c)** GO enrichment analysis of DEGs in different MEs. Node color represents log_10_-transformed FDR-corrected *P* value. Node size represents rich factor. Full list of enriched GO terms is provided in Supplementary Table S5.

To determine the relationships among genes responsive to different light quality and light intensity treatments in *K. fedtschenkoi*, we performed a weighted gene co-expression network analysis (WGCNA) using the DEGs identified from the previous comparisons (Fig.   2). After combining the modules with highly similar expression patterns, a total of 13 co-expression modules were obtained and labeled as different colors (Fig. 4a). The module size ranged from 609 genes (module “cyan”) to 3,047 genes (module “turquoise”). Among the 13 modules, modules “blue” and “salmon” showed low expression at dawn and high expression at dusk under most of the light conditions, except module “blue” that showed low expression and module “salmon” that showed high expression under DG. On the basis of the GO enrichment analysis, module “blue” was enriched in “oxidoreductase activity” but no significantly enriched GO terms were identified in module “salmon.” Module “yellow” showed the opposite expression pattern from modules “blue” and “salmon,” which showed high expression at dawn and low expression at dusk and was functionally enriched in “transport.” The expression of modules “magenta” and “purple” were induced at dawn by LL and HL, but the induction of module “purple” was stronger under HL. These 2 modules were functionally enriched in “chloroplast” (module “magenta”) and “proteasome complex” (module “purple”), respectively. Enriched TF analysis indicated that MYB TFs in module “purple” was stronger than that in module “magenta” (Supplementary Fig. S6). Two opposite modules “tan” and “turquoise” showed low and high expression under DG, which were enriched in “photosynthesis” and “phosphorylation,” respectively (Fig. 4 and Supplementary Table S5).

**Figure 5:**
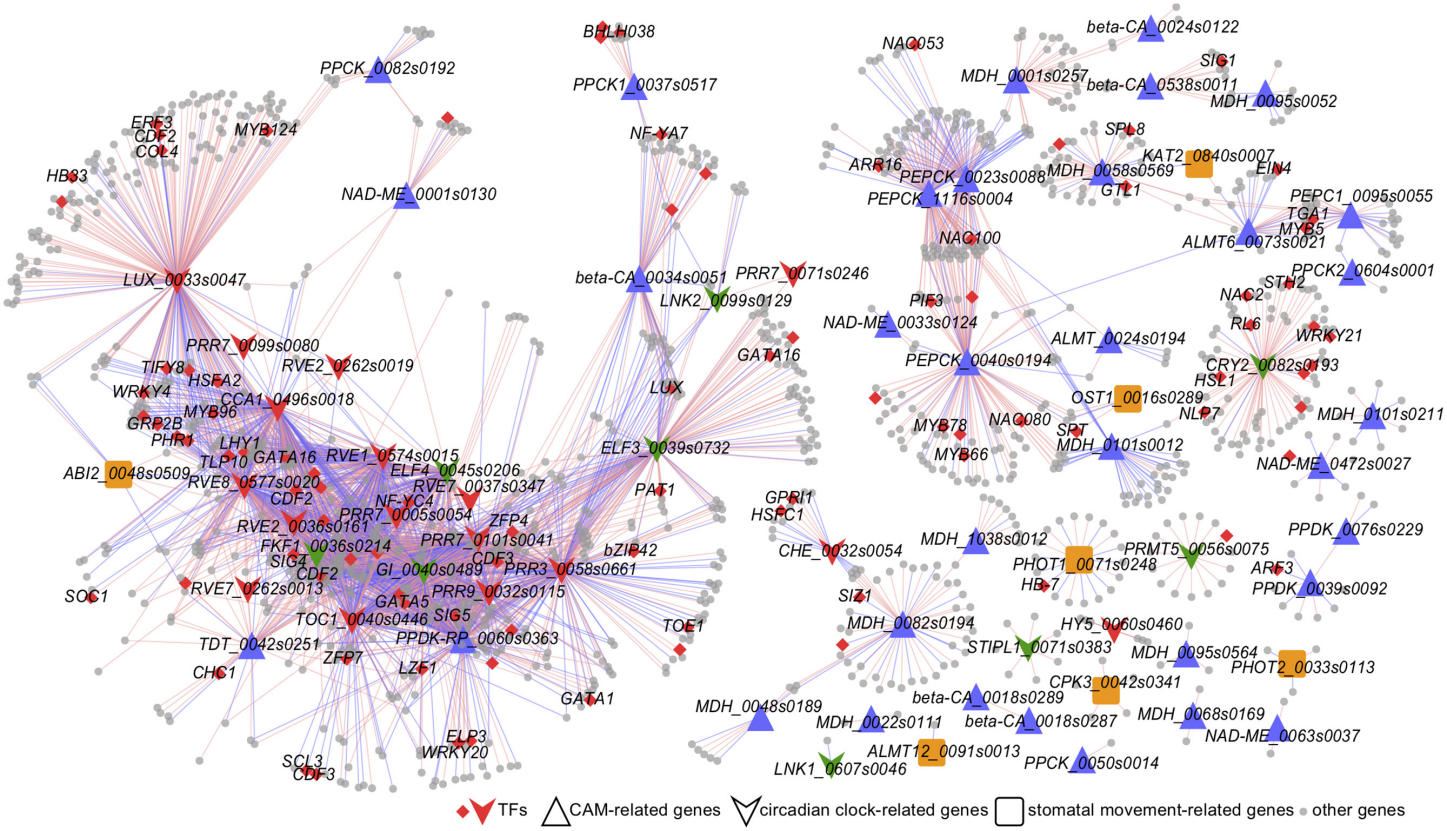
Subnetwork of CAM, circadian clock, and stomatal movement. Red nodes represent transcription factors (TFs). Triangle, arrowhead, and rounded-rectangle shapes of nodes represent CAM-, circadian clock–, and stomatal movement–related genes, respectively. Red and blue edges represent positive correlation (PCC > 0.95 and *P* ≤ 0.01) and negative correlation (PCC < -0.95 and *P* ≤ 0.01), respectively.

To further understand the response of different pathways in CAM plants under various light quality and light intensity treatments, we extracted subnetworks from the global co-expression network. Here, we selected genes related to CAM, the circadian clock, and stomatal movement [13] as case studies to demonstrate the subnetworks. To simplify the subnetwork, we set a high threshold of Pearson correlation coefficient (|PCC| > 0.95 and *P* ≤ 0.01) to show the strong co-expression relationships. The genes involved in CAM, circadian clock, and stomatal movement pathways were highly associated and were co-expressed with numerous transcription factors (TFs) (Fig. 5), implying that the expression of CAM pathway genes may be directly or indirectly regulated by circadian clock TFs. On the basis of the subnetwork, we identified several known and novel TFs that were related with these pathways. For instance, *LHY1* (*Kaladp0066s0115*) was positively co-expressed with *CCA1* (*Kaladp0496s0018*, PCC = 0.995), *RVE8* (*Kaladp0577s0020*, PCC = 0.993), and *RVE1* (*Kaladp0574s0015*, PCC = 0.983) and was negatively co-expressed with *ELF4* (*Kaladp0045s0206*, PCC = −0.978) and *LUX* (*Kaladp0033s0047*, PCC = −0.969). Similarly, *MYB96* (*Kaladp0095s0568*) and *WRKY4* (*Kaladp0096s0082*) were positively co-expressed with *CCA1* and *RVE8* and were negatively co-expressed with *LUX* (Fig. 5 and Supplementary Table S7). In addition, several TFs not previously reported to be associated with CAM were identified in the subnetwork, such as *LZF1* (*Kaladp0192s0026*), *SOC1* (*Kaladp0016s0148*), *CDF2* (*Kaladp0009s0042* and *Kaladp0095s0211*), *COL4* (*Kaladp0029s0144*), *ZFP4* (*Kaladp0035s0036*), *ZFP7* (*Kaladp0001s0233*), *SIG1* (*Kaladp0538s0007*), *SIG4* (*Kaladp0515s0145*), and *SIG5* (*Kaladp0055s0328*).

### Metabolic changes of *K. fedstchenkoi* response to light quality and light intensity

**Figure 6:**
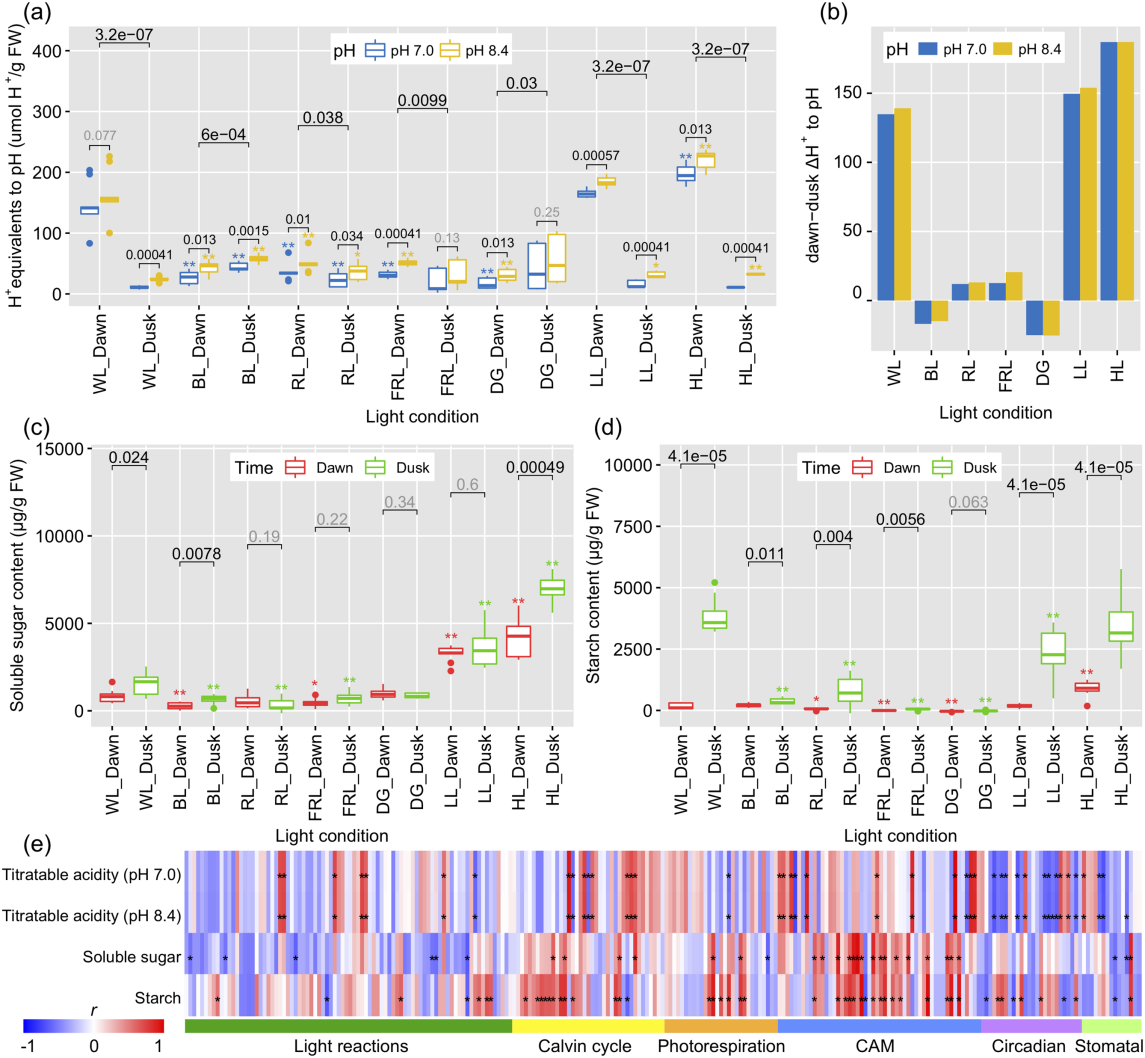
Physiological changes of *Kalanchoë fedtschenkoi* under various light quality and light intensity conditions. **(a)** Nocturnal malic and citric acid accumulation was assessed by the difference in H^+^ concentration between dawn and dusk samples under different light conditions (white light [WL], blue light [BL], red light [RL], far-red light [FRL], dark grown [DG], low light intensity [LL], and high light intensity [HL]). **(b)** Dawn–dusk ΔH^+^ values under different light conditions. **(c)** Soluble sugar contents under different light conditions. **(d)** Starch accumulation under different light conditions. Embedded *P* values indicate statistical differences between dawn and dusk in (a), (c), and (d) and differences between 2 pH treatments (a); while asterisks (*, *P* < 0.05; **, *P* < 0.01) indicate significant differences between light quality/intensity treatments and WL control at dawn or dusk. In each box plot, the central rectangle spans the first quartile to the third quartile, the line inside the rectangle shows the median, and the whiskers denote 1.5 interquartile ranges from the box and outlying values plotted beyond the whiskers. **(e)** Correlation of physiological parameters and gene expression.

To explore whether this light-responsive expression atlas could provide molecular clues for metabolic changes that are diagnostic of CAM activity, we measured leaf titratable acidity (nocturnal malate and citrate accumulation) and diel turnover of soluble sugars and starch. Nocturnal malic and citric acid accumulation was assessed by the difference in H^+^ concentration between dawn and dusk samples (ΔH^+^). Under full-spectrum light, ΔH^+^ increased with the intensity of light, indicating elevated CAM activity. The levels of nocturnal malate and citrate accumulation for these samples were comparable to that observed under acclimating conditions. In contrast, filtered monochrome light (i.e., BL, RL, and FRL) and dark treatment dampened the acid accumulation. RL and FRL treatments, both at photon flux densities of 280 μmol m^−2^ s^−1^, resulted in very low ΔH^+^ values between dawn and dusk samples, indicating reduced CAM activity. Extremely low or inverted dawn/dusk ΔH^+^ values were observed for BL and DG plants, with higher acid accumulation in dusk samples relative to dawn samples (Fig. 6a and b).

Full-spectrum light treatments of both LL and HL resulted in elevated quantities of soluble sugars in *Kalanchoë* leaf tissue, with somewhat higher levels observed in dusk samples relative to the corresponding dawn samples. Soluble sugar accumulation in WL was markedly lower than in LL- and HL-grown plants, although control conditions used a full-spectrum intermediate light intensity. All colored-light and dark treatments resulted in dramatically reduced soluble sugar contents compared with the LL and HL treatments, which is likely a result of the lower photon flux densities delivered to the plants under these conditions (Fig. 6c). Similar to the trends noted for soluble sugars, high levels of leaf starch accumulation were observed in dusk samples for both LL and HL treatments. WL also resulted in large starch content at dusk. Significant accumulation of starch from dawn to dusk was observed for all full-spectrum samples. Lower starch contents were observed under all filtered light treatments, with no detectable starch in dawn FRL samples or dawn and dusk DG plants. The greatest starch content observed for any filtered-light treatment was seen in dusk samples grown under RL, although these starch levels were still much lower than in plants grown under full-spectrum light (Fig. 6d). Again, these low starch accumulation patterns were likely due to the lower photon flux densities delivered to the plants under these light-filtering conditions.

To understand the potential molecular mechanisms regulating these metabolic phenotypes, we performed a “gene expression—phenotypes” correlation analysis. We selected photosynthetic genes including genes involved in light reactions, Calvin cycle, and photorespiration (Fig. 3) and CAM pathway genes including CAM-, circadian-, and stomatal-related genes (Supplementary Table S8) as the candidate genes. Owing to the highly positive correlation between titratable acidities (PCC= 1.00, *P* < 0.001) and between soluble sugar and starch contents (PCC= 0.62, *P* < 0.01) (Supplementary Fig. S7), the correlation patterns of genes with titratable acidities or genes with soluble sugar and starch contents were similar. Notably, different gene sets in the Calvin cycle or CAM pathway were positively correlated with titratable acidities and soluble sugar/starch contents. Most of the circadian-related genes were positively correlated with starch content but negatively correlated with titratable acidity (Fig. 6e and Supplementary Table S9).

### CAM-related genes responsive to light quality and light intensity

**Figure 7:**
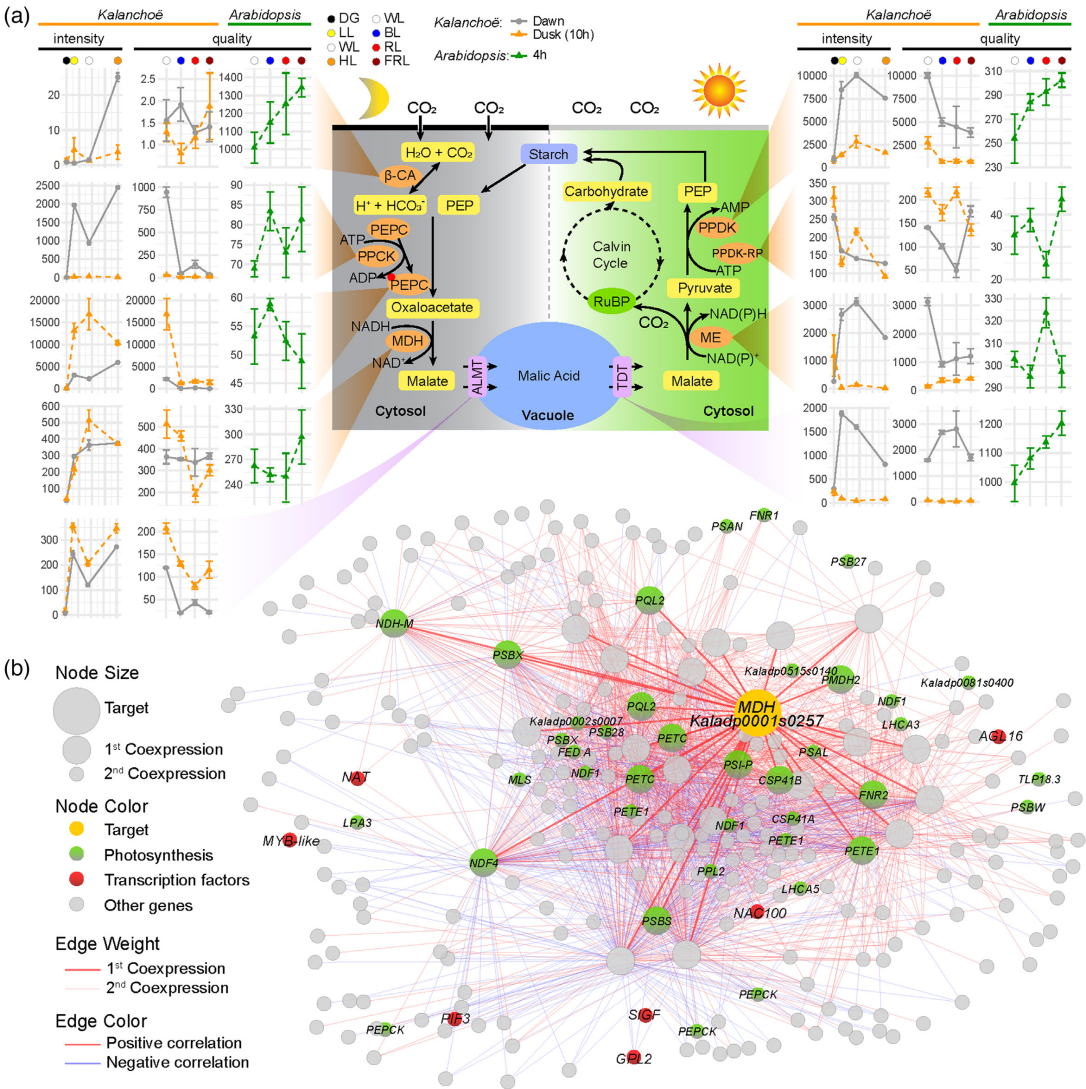
Expression profiles of genes involved in CAM pathway. **(a)** Expression pattern of CAM-related genes under different light intensity (DG, 0 μmol m^−2^ s^−1^; LL, 150 μmol m^−2^ s^−1^; WL, 440 μmol m^−2^ s^−1^; HL, 1,000 μmol m^−2^ s^−1^) and under different light quality (WL, BL, RL, and FRL). The CAM pathway was modified from Yang et al. [13]. Gene name (*Kalanchoë* gene ID, *Arabidopsis* gene ID): *β-CA* (Kaladp0018s0287, AT5G14740), *PPCK1* (Kaladp0037s0517, AT3G04530), *PEPC1* (Kaladp0095s0055, AT3G14940), *MDH2* (Kaladp0001s0257, AT5G09660), *ALMT6* (Kaladp0073s0021, AT1G25480–missed expression data), *PPDK* (Kaladp0076s0229, AT4G15530), *PPDK-RP* (Kaladp0010s0106, AT4G21210), *NADP-ME* (Kaladp0092s0166, AT1G79750), *TDT* (Kaladp0042s0251, AT5G47560). The expression of *Kalanchoë* genes was detected at dawn (2 h before the light period) and dusk (2 h before the dark period, i.e., 10 h after light treatments). The expression data of *Arabidopsis* responsive to light quality (WL, BL, RL, and FRL) was obtained from *Arabidopsis* eFP browser (light series). *Arabidopsis* seeds were plated on 1.2% Murashige and Skoog agar and stratified at 8°C for 48 h in the dark. Germination was induced with 2 h red light, followed by growth for 94 h in complete darkness at 22°C. Plants were then irradiated at different light conditions. Samples (mainly hypocotyl and cotyledons) were collected at 4 h after treatments. **(b)** Subnetwork of *MDH2* (Kaladp0001s0257). Orange nodes represent center of the subnetworks; red and green nodes represent TFs and photosynthesis-related genes, respectively. Large and small nodes represent the first and second co-expressed genes, respectively. Thick and thin edges indicate the first and second co-expression relationships, respectively. Red and blue edges indicate the positive and negative correlation, respectively.

To further investigate the CAM-specific response to various light quality and light intensity treatments, the expression patterns of CAM-related genes were analyzed (Fig. 7a). The key genes that are involved in nocturnal CO_2_ assimilation and malate storage include *β-CA* (*β*-carbonic anhydrase), *PEPC* (phosphoenolpyruvate carboxylase), *PPCK* (phosphoenolpyruvate carboxylase kinase), *MDH*[NAD(P)-malate dehydrogenase], and *ALMT* (aluminum-activated malate transporter). *β-CA* (*Kaladp0018s0287*) was highly expressed at dawn under high light compared to other light quality and light intensity treatments. *PEPC1* (*Kaladp0095s0055*) showed relatively higher expression levels at dusk than dawn under different light quality and light intensity conditions, yet *PEPC1* expression levels at both dawn and dusk were repressed by filtered monochrome light (i.e., BL, RL, and FRL). Similarly, the expression of *PPCK1* (*Kaladp0037s0517*) was also repressed by filtered monochrome light. *MDH2* (*Kaladp0001s0257*) was constitutively induced by increasing light intensity at dawn but had a peak value under WL at dusk (Fig. 7a).

During daytime, the CO_2_ release from malate and refixation is mediated by a series of genes that include *TDT*(tonoplast dicarboxylate transporter), *NAD(P)-ME*[NAD(P)-malic enzyme], and *PPDK* (pyruvate phosphate dikinase). As the first-step transporter for the daytime reactions, *TDT* (*Kaladp0042s0251*) showed an obvious trend of greater transcript abundance at dawn and down-regulation at dusk, and expression at dawn was stronger under BL and RL than under the WL control. In contrast, *NADP-ME* (*Kaladp0092s0166*) showed similar expression patterns with TDT under light intensity treatment, but its expression at dawn was low under BL and RL. *PPDK* (*Kaladp0076s0229*) was increased by light intensity increase and reached the highest level under WL at both dawn and dusk but was decreased by filtered monochrome light (Fig. 7a). In addition, we compared the light quality responses of CAM-related genes between *Kalanchoë* and *Arabidopsis*, although the sampling time was different compared to our study—*Arabidopsis* samples were collected at 4 h after treatments and our *Kalanchoë* dusk samples were collected at 10 h after treatments. All these CAM-related genes showed different expression patterns between *Kalanchoë* and *Arabidopsis* (Fig. 7a).

Based on our light-responsive co-expression network, we extracted the subnetwork of *MDH*. This subnetwork consists of 274 genes, 39 of which are photosynthesis-related and 7 of which are TFs (Fig. 7b). Those photosynthesis-related genes in the subnetwork include photosystem I subunits (i.e., PSAL, PSAN, PSAP), photosystem II subunits (i.e., PSB17, PSB28, PSBP, PSBW, and PSBX), photosystem I light harvesting complex genes (i.e., LHCA3, LHCA5). Three TFs, *AGL16* (*Kaladp0067s0150*), *SIGF* (*SIG6, Kaladp0872s0002*), and *PIF3* (*Kaladp0076s0003*), in this subnetwork are known regulators in photosynthesis or circadian rhythm. In addition, we compared the expression pattern of circadian rhythm–related genes at dawn and dusk under different light conditions. Most of the circadian rhythm–related genes such as *CCA1, CRY2, ELF3/4, HY5, PRR7/9, RVE1/6/8*, and *TOC1* have disordered expression patterns between dawn and dusk under DG (Supplementary Fig. S8), indicating that the circadian clock is likely disrupted under DG.

### Photoreceptors and stomatal movement–related genes responsive to light quality in *K. fedtschenkoi*

**Figure 8:**
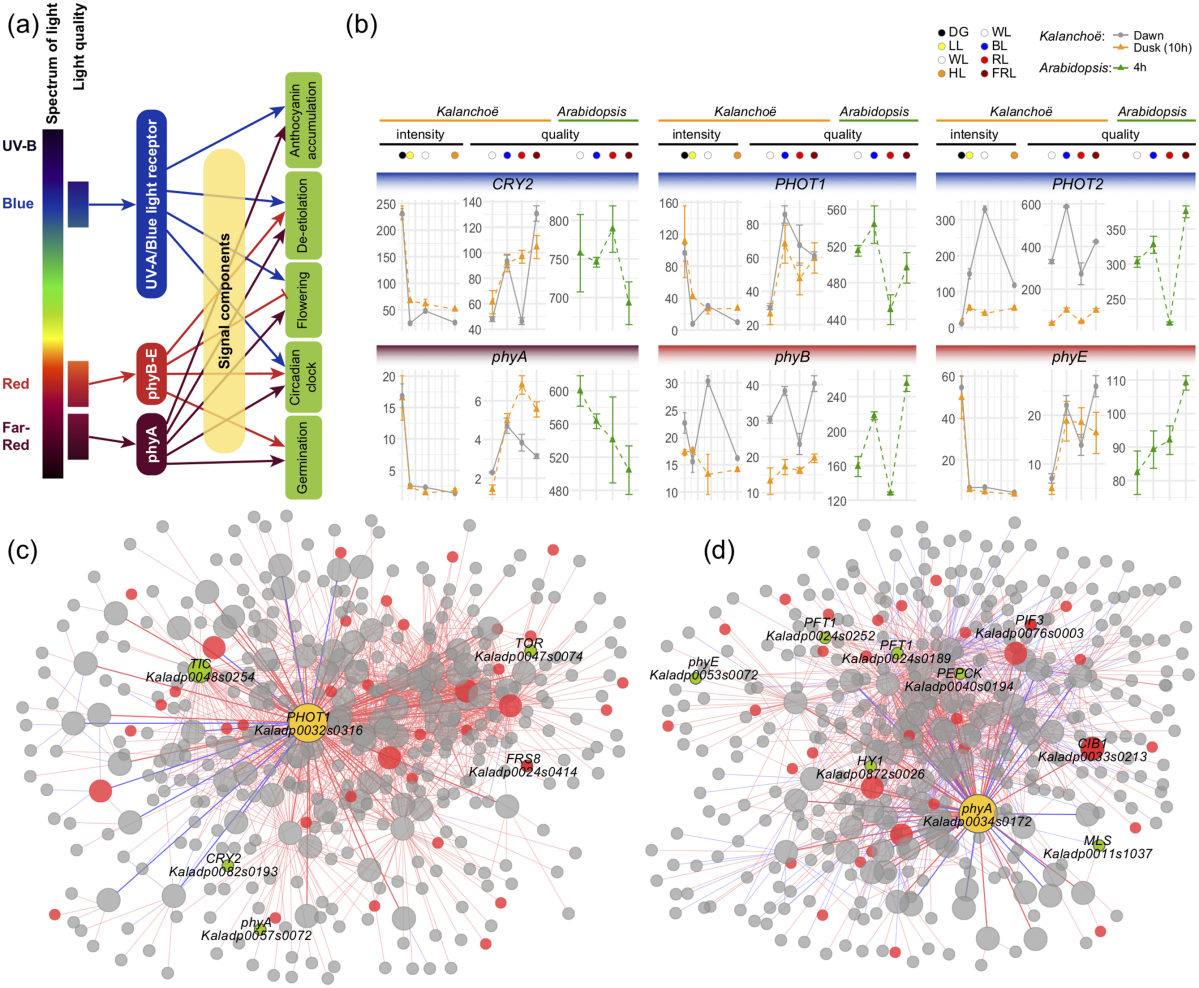
Expression patterns of photoreceptors in *Kalanchoë* leaf. **(a)** Light signaling model from photoreceptor to photo-responsiveness. **(b)** Expression patterns of photoreceptors under different light conditions. Gene name (*Kalanchoë* gene ID, *Arabidopsis* gene ID): *CRY2* (Kaladp0082s0193, AT1G04400), *PHOT1* (Kaladp0032s0316, AT3G45780), *PHOT2* (Kaladp0055s0063, AT5G58140), *phyA* (Kaladp0034s0172, AT1G09570), *phyB* (Kaladp0039s0298, AT2G18790), *phyE* (Kaladp0053s0072, AT4G18130). The expression data of *Arabidopsis* responsive to light quality (WL, BL, RL, and FRL) were obtained from *Arabidopsis* eFP browser (light series). **(c)** Subnetwork of *PHOT1*. **(d)** Subnetwork of *phyA*. Orange nodes represent center of the subnetworks; red and green nodes represent TFs and circadian-/light-responsive genes, respectively. Large and small nodes represent the first and second co-expressed genes, respectively. Thick and thin edges indicate the first and second co-expression relationships, respectively.

We tested the expression patterns of photoreceptors in our *Kalanchoë* dataset. As shown in Fig. 8, blue-light receptors *PHOT1* (*Kaladp0032s0316*) and *PHOT2* (*Kaladp0055s0063*) showed up-regulation under BL at both dawn and dusk. *phyA* (*Kaladp0034s0172*) showed up-regulation under both RL and FRL at dusk. The expression of *PHOT1, PHOT2*, and *phyB* at dusk (10 h after treatments) in *Kalanchoë* showed similar patterns with their orthologs at 4 h after treatments in *Arabidopsis* (Fig. 8b). We then constructed the subnetworks of *PHOT1* (*Kaladp0032s0316*) and *phyA* (*Kaladp0034s0172*) on the basis of our co-expression database. Five known regulators involved in light responses were co-expressed with *PHOT1* (*Kaladp0032s0316*), which included *TIC* (*Kaladp0048s0254*) *phyA* (*Kaladp0057s0072*), *FRS8* (*Kaladp0024s0414*), *TOR* (*Kaladp0047s0074*), and *CRY2* (*Kaladp0082s0193*) (Fig. 8c). Similarly, we identified 6 known light-responsive regulators that were co-expressed with *phyA* (*Kaladp0034s0172*), which included cryptochrome-interacting basic-helix-loop-helix TF CIB1 (*Kaladp0033s0213*), 2 copies of phytochrome and flowering time regulatory protein PFT1 (*Kaladp0024s0189* and *Kaladp0024s0252*), phyE (Kaladp0053s0072), phytochrome-interacting factor 3 (*PIF3, Kaladp0076s0003*), and HY1 (*Kaladp0872s0026*). In addition, 2 CAM-related genes, *PEPCK* (*Kaladp0040s0194*) and *MLS* (*Kaladp0011s1037*), were co-expressed with *phyA* (*Kaladp0034s0172*) (Fig. 8d).

**Figure 9:**
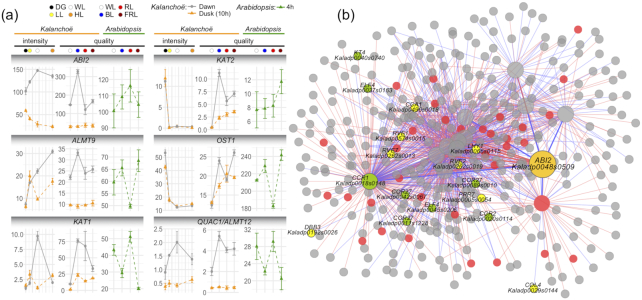
The expression pattern of *Kalanchoë fedtschenkoi* stomatal movement–related genes. **(a)** Expression of stomatal movement–related genes under different light conditions. Gene name (*Kalanchoë* gene ID, *Arabidopsis* gene ID): *ABI2* (Kaladp0048s0509, AT5G57050), *ALMT9* (Kaladp0062s0038, AT3G18440), *KAT1* (Kaladp0008s0789, AT5G46240), *KAT2* (Kaladp0840s0007, AT4G18290), *OST1* (Kaladp0016s0289, AT4G33950), *QUAC1/ALMT12* (Kaladp0091s0013, AT4G17970). The expression data of *Arabidopsis* responsive to light quality (WL, BL, RL, and FRL) was obtained from *Arabidopsis* eFP browser (light series). **(b)** Subnetwork of stomatal movement–related gene *ABI2*. Orange nodes represent center of the subnetworks; green, yellow, and red nodes represent TFs and circadian genes, circadian TFs, and other TFs, respectively. Large and small nodes represent the first and second co-expressed genes, respectively. Thick and thin edges indicate the first and second co-expression relationships, respectively.

Most of the stomatal movement–related genes were induced by BL, especially at dawn (e.g., *ABI2, ALMT9, KAT1, KAT2*, and *QUAC1/ALMT12*) (Fig. 9a). To explore the potential regulatory mechanism, we tested the subnetwork of *ABI2*. Notably, numerous circadian rhythm–related regulators were co-expressed with *ABI2*, which included *CCA1* (*Kaladp0496s0018*), *CCR1* (*Kaladp0018s0148*), *CCR2* (*Kaladp0020s0114*), COL4 (*Kaladp0029s0144*), 3 copies of *COR27* (*Kaladp0011s1228, Kaladp0042s0067*, and *Kaladp0089s0010*), *DBB3* (*Kaladp0192s0026*), 2 copies of *ELF4* (*Kaladp0037s0163* and *Kaladp0045s0206*), *KT4* (*Kaladp0040s0740*), *LHY1* (*Kaladp0066s0115*), *PRR7* (*Kaladp0005s0054*), *RVE1* (*Kaladp0574s0015*), *RVE2* (*Kaladp0262s0019*), and *RVE7* (*Kaladp0262s0013*).

## Discussion

As one of the most important environmental factors, light affects plant growth and development, plant physiology, and metabolism [28, 29]. Although light quality effects at the metabolic and molecular levels have been studied in several plant species [30–34], genome-wide transcriptomic studies of the effects of light quality and light intensity on CAM species are lacking. Low-fluorescence red and blue light were shown to modulate the diel metabolic processes in an obligate CAM species, *Aechmea* “Maya” [19]. However, the regulatory mechanisms underpinning such light-induced metabolic reprogramming in CAM species remains largely unexplored.

In this study, we created a comprehensive, genome-wide, light-responsive gene expression atlas for *K. fedtschenkoi*. We used 1 plant for each of the 3 biological replicates for RNA-Seq analysis, which could potentially reduce the false-positive rate of differential gene expression in comparison with pooling multiple plants into each biological replicate. The eFP browser provides a useful web interface for easy data access, facilitating comparative and functional genomics research. Furthermore, the RNA-Seq data were analyzed by pairwise comparisons between different light conditions and different time points to identify DEGs in *K. fedtschenkoi*. These DEGs were then subject to clustering and co-expression analyses. A similar approach was effectively used to discover the regulatory networks in *Brachypodium distachyon* [26], pigeon pea [35], and chickpea [36]. Combined with functional analysis, such as GO enrichment analysis, we found that the overlapped DEGs at dusk were mainly involved in “carbohydrate metabolism” and “response to endogenous stimulus” processes, consistent with previous studies showing that light quality affects the regulation of endogenous hormone stimulus involving gibberellin, auxins, cytokinins, and abscisic acid [3, 37–39].

We found that a high level (1,000 μmol m^−2^ s^−1^) of WL increased expression of 3 dark CO_2_ fixation genes (i.e., *β-CA, PPCK, PEPC*) and 1 malate transporter gene (*ALMT*) at dawn (i.e., 2 h before the beginning of the light period) in comparison with an intermediate level (440 μmol m^−2^ s^−1^) of WL (Fig. 7a). This up-regulation of genes involved in dark CO_2_ fixation and malate import into the vacuoles was consistent with the higher acid accumulation (i.e., dawn–dusk ΔH^+^) under HL relative to intermediate light intensity (Fig. 6b). On the other hand, we found that a high level of WL repressed expression of several CAM pathway genes (e.g., *PEPC, MDH, PPDK, PPDK-RP*) at dusk (i.e., 2 h before the beginning of the dark period) in comparison with an intermediate level of WL (Fig. 7a). These results suggest that the high-light treatment was still within the normal physiological range of the plant whilst not saturating the photosynthetic machinery.

In comparison with WL, BL repressed the expression of 3 genes (*β-CA, PEPC*, and *ALMT*) involved in dark CO_2_ fixation and malate transport as well as 2 genes (*PPDK* and *PPDK-RP*) in the light phase of the CAM pathway at dusk (i.e., 2 h before the beginning of the dark period) (Fig. 7a). Similarly, RL/FRL repressed the expression of 3 genes (*PEPC, MDH*, and *ALMT*) involved in dark CO_2_ fixation and malate transport as well as 1 gene (*PPDK*) in the light phase of the CAM pathway at dusk (i.e., 2 h before the beginning of the dark period) (Fig. 7a). This monochrome light–induced gene repression was consistent with the much lower acid accumulation (i.e., dawn–dusk ΔH^+^) under BL/RL/FRL conditions (Fig.   6b). These results indicate that BL/RL/FRL treatment interferes with the optimal performance of the CAM pathway in *K. fedtschenkoi*.

*K. fedtschenkoi* is a model plant species for CAM functional genomics research [13, 15]. Our co-expression analysis highlighted a subnetwork of CAM-related regulatory/signaling genes, such as *LHY1*, which was positively co-expressed with *CCA1, RVE1*, and *RVE8* but was negatively co-expressed with *ELF4* and *LUX* (Fig. 5). In *Arabidopsis*, ELF4, ELF3, and LUX can form an ELF4-ELF3-LUX protein complex (the evening complex), which is regulated by light and the circadian clock [40]. MYB-related protein CCA1 and LHY1 can form homodimers and regulate the expression of evening-element–containing genes [41]. There is a negative-feedback loop among these transcription factors. EFL4 and LUX are required for the red-light induction of *CCA1* and *LHY1*, whereas CCA1 and LHY1 negatively regulate the expression of ELF4 [42] and LUX [43]. The co-expression relationships of these TFs in *K. fedtschenkoi* reported here indicate that the circadian rhythm regulatory mechanism among these genes is conserved between *K. fedtschenkoi* and *Arabidopsis*.

*MYB96*, a TF involved in the circadian clock in *Arabidopsis*, was also identified in our subnetwork as positively co-expressed with *CCA1* and *RVE8* (Fig. 5). As a key regulator connecting the circadian clock and the environment, *MYB96* is induced by high levels of ABA and can directly bind to the promoter of *TOC1* to activate its expression. *MYB96* is directly regulated by CCA1 through multiple CCA1-binding sites (CBS: AAAATCT) and evening elements (EE: AAATATCT). Interestingly, CCA1 binds to the promoter of *MYB96* at dawn but not at dusk [44, 45]. These findings suggest that our constructed co-expression network is reliable for conserved light-responsive regulator identification.

Several transcription factors with unknown CAM function were identified in the *K. fedtschenkoi* co-expression network. These transcription factors potentially represent novel regulatory mechanisms. As shown in Fig. 5, *WRKY4* was positively co-expressed with *CCA1* and *RVE8* but negatively co-expressed with *LUX*. Although there is no direct evidence for the involvement of the *WRKY4* gene in circadian regulation, its homolog in tomato showed up-regulation at 8 hours after dawn, presumptive dusk, and 4 hours after dusk in comparison with presumptive dawn under long-day conditions [46]. Furthermore, some abscisic acid and light signaling–related genes were identified in our CAM gene-enriched subnetwork (e.g., zinc finger proteins (*ZFP4* and *ZFP7*), σ factors (*SIG1, SIG4*, and *SIG5*), and B-BOX protein (*STH2/BBX21*). In *Arabidopsis*, homologs of *ZFP* are involved in light-responsive pathways, where *ZFP3* can interfere with ABA and light signal in plant development and seed germination [47], while *ZFP1* is expressed in a pathway that is downstream of photomorphogenesis [48]. In prokaryotes, σ factors are well known for their participation in the control of RNA polymerase activity. The phosphorylation of SIG1 selectively inhibits the expression of gene-encoding photosystem I [49]. *SIG1* is strongly induced by RL and BL, but *SIG5* is only induced by BL under the mediation of *CRY1* and *CRY2* [50, 51]. As a key component involved in the COP1-HY5 hub, *STH2/BBX21* is controlled by COP1 through its E3 ubiquitin ligase activity in darkness and promotes photomorphogenesis by activating *HY5* in the light [52]. Our results provide a powerful resource for light-responsive regulator identification.

In addition, the subnetworks of specific genes provide insight on the molecular mechanisms underpinning the different light-induced metabolic phenotypes. For example, the subnetwork of *MDH* (*Kaladp0001s0257*) shows an enrichment of photosynthetic genes (Fig. 7b). The TFs (*AGL16, SIGF*, and *PIF3*) in this subnetwork might be the dominant regulators of this subnetwork. The ortholog of *AGL16* in *Arabidopsis* is targeted for sequence-specific degradation by *miR824*; expression of a *miR824*-resistant *AGL16* increased the incidence of stomata in high-order complexes in transgenic plants [53]. *SIGF* (*SIG6*) encodes a general σ factor in chloroplasts. Expressing chimeric σ factor genes in *Arabidopsis sigf* mutant affects the expression of numerous plastid genes [54]. *PIF3* is a key basic helix-loop-helix transcription factor of *Arabidopsis* that negatively regulates light responses, repressing chlorophyll biosynthesis, photosynthesis, and photomorphogenesis in the dark. PIF3 and HDA15 are dissociated from the target genes upon exposure to RL. PIF3 associates with HDA15 to repress chlorophyll biosynthetic and photosynthetic genes in etiolated seedlings [55].

In the subnetwork of stomatal movement–related gene *ABI2*, 16 known circadian rhythm–related regulators were identified (Fig. 9b), which included synergistically functional *CCA1* and *LHY1*, and their downstream gene *PPR7* [56]. *RVE1* is homologous to *CCA1* and *LHY1*, but inactivation of *RVE1* does not affect circadian rhythmicity but instead causes a growth phenotype [57]. However, another 2 *RVE* genes (*RVE2* and *RVE7*) in this subnetwork are directly involved in circadian regulation. RVE2 (CIR1) is possibly part of a regulatory feedback loop that controls a subset of the circadian outputs and modulates the central oscillator [58]. RVE7 (EPR1) is a component of a slave oscillator that contributes to the refinement of output pathways, ultimately mediating the correct oscillatory behavior of target genes [59]. Our dataset provides additional insights into the photo-responsiveness of CAM plants. In addition, the “gene expression—metabolic phenotypes” correlation analysis identified a series of positively and negatively correlated genes associated with photosynthesis and CAM pathways, which provided molecular clues for understanding the physiological changes that accompany light quality and light intensity responses in *Kalanchoë*.

In conclusion, the comprehensive light-responsive gene expression atlas of *K. fedtschenkoi* provides a useful genomics resource for investigating the molecular mechanisms underlying responses to light quantity and quality in CAM plants. The genome-wide co-expression network lays a solid foundation for discovering novel gene function in CAM plants. Furthermore, the results from our comparative analyses of gene expression and acid accumulation between different light treatments support our hypothesis that both light intensity and light quality can affect the expression of CAM-related genes in *K. fedtschenkoi*.

## Methods

### Plant material and experimental treatments

*Kalanchoë fedtschenkoi* (ORNL diploid accession M2) plants originally started from meristem cuttings were grown in soil for 4 weeks in a Percival Model AR-75L2 growth chamber on a 12-h light (26°C)/12-h dark (18°C) cycle at a photon flux density of 280 μmol m^−2^ s^−1^. For acclimation prior to light quality or light intensity treatments, plants were placed for ≥2 d in the growth chamber on a 12-h light (26°C)/12-h dark (18°C) cycle at a photon flux density of 440 μmol m^−2^ s^−1^. Light quality treatments consisted of then growing plants under BL (270 μmol m^−2^ s^−1^) provided by a dark blue gel filter (No. 119), RL (280 μmol m^−2^ s^−1^) provided by primary red gel filter (No. 106), FRL (280 μmol m^−2^ s^−1^) provided by a medium red Roscolux filter (Barndoor Lighting Outfitters, Inc., North Branford, CT), or constant darkness. For all treatments, except constant darkness, a 12-h light (26°C)/12-h dark (18°C) cycle was used. Light intensity treatments consisted of growing the plants under DG, LL (150 μmol m^−2^ s^−1^), or HL (1,000 μmol m^−2^ s^−1^) with a 12-h light (26°C)/12-h dark (18°C) cycle, respectively. All plants used for light quality and light intensity experiments were grown under the indicated conditions for 48 h prior to any tissue collection. All photon flux density measurements described above were taken at leaf level of the apical meristem because these leaves were closest to the light source.

### Tissue collection and RNA isolation

Fully expanded leaves (i.e., leaf pair 4–5 counting from the top of the plants) were collected from 3 biological replicates of plants (each biological replicate was 1 independent plant) grown under each of the light quality and light intensity experimental conditions. Each sample was collected at both dawn (2 h before the light period) and dusk (2 h before the dark period) time points, wrapped in aluminum foil, immediately frozen in liquid nitrogen, and stored at −80°C until processing. For RNA isolation, frozen leaf tissue samples were ground to a fine powder under liquid nitrogen with a mortar and pestle. Isolation of total RNA then proceeded by using the QIAGEN RNeasy^®^ Plant Mini Kit (Cat No. 74,904, Qiagen, Inc., Valencia, CA, USA) with the following modifications: 600 mg of frozen ground tissue from each sample was mixed thoroughly with 2.57 mL of Fruit Mate™ (TaKaRa Bio USA, Inc., Mountain View, CA). The resulting suspension was centrifuged at 14,000*g* at 4°C for 5 min. The supernatant was then mixed with 1.8 mL of QIAGEN buffer RLT/2-mercaptoethanol mix. This solution was centrifuged at 14,000*g* at 25°C for 1 min. The supernatant was then mixed with 0.5 volumes of 100% ethanol and the remaining steps were performed according to kit instructions. On-column DNase digestions were performed for all samples according to RNeasy^®^ kit instructions with the Qiagen RNase-Free DNase Set (Cat No. 79,254). Final RNA elution was performed with 50 μL of RNase-free water, which was run through the column twice. RNA purity and approximate quantity was assessed with a Thermo Scientific™ NanoDrop 2000c spectrophotometer and precise quantity assessed with Quant-iT^TM^ RiboGreen^®^ fluorescence (Thermo Scientific, Rockford, IL). RNA integrity was evaluated on a 1% (w/v) agarose gel using 300 ng RNA.

### RNA-Seq library construction and RNA-seq

A total of 42 libraries (7 light conditions × 2 time points × 3 biological replicates) were constructed, and RNA-seq was performed independently. The total RNA samples were sequenced in the Department of Energy Joint Genome Institute (Walnut Creek, CA). Briefly, the integrity and concentration of the RNA preparations were checked initially using Nano-Drop ND-1000 (Nano-Drop Technologies) and then by BioAnalyzer (Agilent Technologies). Plate-based RNA sample preparation was performed on the PerkinElmer Sciclone NGS robotic liquid handling system using Illumina's TruSeq Stranded mRNA HT sample preparation kit using poly-A selection of mRNA with the following conditions: total RNA starting material was 1 µg per sample and 8 cycles of PCR was used for library amplification. The prepared libraries were then quantified by qPCR using the Kapa SYBR Fast Illumina Library Quantification Kit (Kapa Biosystems, , Wilmington, MA) and run on a Roche LightCycler 480 real-time PCR instrument. The quantified libraries were then prepared utilizing a TruSeq paired-end cluster kit, v4, and Illumina's cBot instrument to generate a clustered flowcell for sequencing. Sequencing of the flowcell was performed on the Illumina HiSeq2500 platform using HiSeq TruSeq SBS sequencing kits, v4, following a 2 × 150 indexed run recipe.

### Read mapping and data analysis

After filtering out low-quality reads, RNA-seq reads from each library were aligned to the *K. fedtschenkoi* reference genome [13] using GSNAP v2018-07-04 (GSNAP, RRID:SCR_005483) [60]. The FeatureCounts function of Subread v1.6.1 (Subread, RRID:SCR_009803) [61] was used to generate raw gene counts, and only reads that mapped uniquely to 1 locus were counted. Gene expression was estimated as transcripts per million (TPM) [62]. DESeq2 v1.2.10 (DESeq2, RRID:SCR_015687) [63] was subsequently used to determine which genes were differentially expressed between pairs of conditions. The parameters used to “call a gene” between conditions were determined at a false discovery rate (FDR) adjusted *P-*value of ≤0.05. DEGs were classified into hierarchical categories “BINs” using MapMan version 3.6.0RC1 (MapMan, RRID:SCR_003543) [64]. Gene Ontology (GO) enrichment analysis was applied to predict gene function and calculate the functional category using BiNGO (BiNGO, RRID:SCR_005736) [65]. Heat map and bubble plots were generated by the R package ggplot2. All tools were run with default parameters.

### *Kalanchoë* light-responsive eFP browser

TPM-normalized values of the RNA-Seq datasets were uploaded into the *Kalanchoë* eFP browser of the Bio-Analytic Resource (BAR). Representative images of *Kalanchoë* leaf under different light conditions were created and an XML file was generated to power a view within the *Kalanchoë* eFP browser [27].

### Co-expression analysis

For co-expression analysis, the log_2_-normalized TPM values of all the samples were used to construct a weighted gene co-expression network using the R package WGCNA [66].

### Titratable acidity assays of leaf tissue

For titratable acidity experiments, ∼0.5 g of frozen, finely ground leaf tissue was added to 10.0 mL of 50% (v/v) methanol and mixed well. This suspension was then boiled at 80°C for 10 min. Additional 50% (v/v) methanol was added after boiling to any samples that showed volume loss. The boiled samples were then centrifuged at 2,000*g* for 10 min at room temperature. Supernatants were titrated with 10 mM KOH to pH = 7.0 and 8.4, corresponding to malate and citrate, respectively. Leaf titratable acidities were expressed as µmol H^+^ g^−1^ fresh weight.

### Carbohydrate analysis of leaf tissue

For soluble sugar and starch assays, ground leaf tissue was boiled in 10.0 mL of 50% (v/v) methanol at 80°C for 30 minutes. Sample volumes were adjusted back to original volumes with 50% (v/v) methanol. Samples were then centrifuged at 2,000*g* for 10 min at room temperature. The resulting supernatants were reserved for soluble sugar analysis. For starch extraction, tissue pellets were washed twice with 10 mL of Nanopure water with centrifugation at 2,000*g* after each wash. A total of 1.2 mL of acetate buffer (prepared from 86 mL of 0.1 M sodium acetate and 114 mL of 0.1 M acetic acid, pH 4.5) was added to the washed pellet and resuspended by vortexing. Then, 0.2 mL of starch digestion solution (300 units α-amyloglucosidase and 25 units α-amylase prepared in 20 mL acetate buffer) was added and the mixture incubated overnight at 45°C. Starch-digested samples were centrifuged at 2,000*g* for 10 min at room temperature and the supernatant used for starch analysis. Colorimetric assays for determination of starch and soluble sugar content were performed as described [67].

## Availability of Supporting Data and Materials

All raw short reads are available in the NCBI SRA database (SRA accessions: SRP146136, SRP146139, SRP146175, SRP146204, SRP146205, SRP146213, SRP148019–SRP148030, SRP148037–SRP148060) (Supplementary Table S1). Supporting data are also available via the *Kalanchoë* eFP Browser [27] and via the *GigaScience* database GigaDB [68].

## Additional Files

**Figure S1**. Expression distribution and correlation of 42 RNA-seq libraries. (a) Distribution of gene expression levels of all the samples in this study. The gene expression levels were transformed by log_10_(TPM + 1). (b) Pearson correlation between samples.

**Figure S2**. Gene ontology (GO) enrichment of DEGs in different comparisons. BP, biological process; MF, molecular function; and CC, cellular component. GOslim terms are shown here; full list of enriched GO terms is presented in Supplementary Table S3.

**Figure S3**. Functional classification of DEGs in MapMan BINs. DEG number (a) and DEG percentage (b) of different comparisons in 29 MapMan BINs.

**Figure S4**. Expression pattern of DEGs in different MapMan BINs. Color scale of blue-white-red represents *Z*-score–normalized relative expression in the 14 samples.

**Figure S5**. Transcription factor (TF) number and enrichment in the co-expression modules.

**Figure S6**. Scatter plots (lower triangle) and correlations (upper triangle) among 4 physiological traits of *K. fedtschenkoi* under different light treatments.

**Figure S7**. The expression pattern of *Kalanchoë fedtschenkoi* circadian rhythm–related genes at dawn and dusk under different light conditions.

**Table S1**. Experimental conditions and statistics of RNA-Seq data in this study.

**Table S2**. Differentially expressed genes (DEGs) in pairwise comparisons.

**Table S3**. Full list of enriched GO terms of DEGs in different comparisons.

**Table S4**. Expression patterns of photosynthetic genes.

**Table S5**. Full list of enriched GO terms of different WGCNA modules.

**Table S6**. Transcription factors in WGCNA modules.

**Table S7**. Gene list and functional annotation of the subnetwork.

**Table S8**. Expression patterns of CAM-, circadian-, stomatal movement–related, and photosynthetic genes.

**Table S9**. Correlation of physiological parameters and gene expression.

giaa018_GIGA-D-19-00095_Original_Submission

giaa018_GIGA-D-19-00095_Revision_1

giaa018_GIGA-D-19-00095_Revision_2

giaa018_Response_to_Reviewer_Comments_Original_Submission

giaa018_Response_to_Reviewer_Comments_Revision_1

giaa018_Reviewer_1_Report_Original_Submission Aureliano Bombarely, Ph.D. -- 4/15/2019 Reviewed

giaa018_Reviewer_1_Report_Revision_1 Aureliano Bombarely, Ph.D. -- 11/24/2019 Reviewed

giaa018_Reviewer_2_Report_Original_Submission Alisandra Denton -- 4/25/2019 Reviewed

giaa018_Reviewer_2_Report_Revision_1 Alisandra Denton -- 11/22/2019 Reviewed

giaa018_Supplemental_Files

## Abbreviations

ALMT: aluminum-activated malate transporter; BAR: Bio-Analytic Resource; β-CA;*β*-carbonic anhydrase; BiNGO: Biological Networks Gene Ontology; BL: blue light; bp: base pairs; BP, biological process; CAM: crassulacean acid metabolism; CBS: CCA1 binding sites; CC, cellular component; DEG: differentially expressed gene; DG: dark grown; EE: evening elements; FDR: false discovery rate; FRL: far-red light; Gb: gigabase pairs; GO: gene ontology; HL: high light; LL: low light; MDH: NAD(P)-malate dehydrogenase; MDS: multidimensional scaling; MF, molecular function; mRNA: messenger RNA; NAD(P)-ME: NAD(P)-malic enzyme; NCBI: National Center for Biotechnology Information; ORNL: Oak Ridge National Laboratory; PC: principal component; PCC: Pearson correlation coefficient; PEPC: phosphoenolpyruvate carboxylase; PPCK: phosphoenolpyruvate carboxylase kinase; PPDK: pyruvate phosphate dikinase; RL: red light; RNA-Seq: RNA sequencing; Rubisco: ribulose-1,5-bisphosphate carboxylase/oxygenase; SRA: Sequence Read Archive: TDT: tonoplast dicarboxylate transporter; TF: transcription factor; TPM: transcripts per million; WGCNA: weighted gene co-expression network analysis; WL: white light.

## Competing Interests

The authors declare that they have no competing interests.

## Funding

This research was supported by the U.S. Department of Energy, Office of Science, Genomic Science Program under Award No. DE-SC0008834. Additional support was provided by the Community Science Program (project 503025) at the Department of Energy Joint Genome Institute and the DOE Center for Bioenergy Innovation at the Oak Ridge National Laboratory. This manuscript has been authored by UT-Battelle, LLC, under Contract No. DE-AC05-00OR22725 with the U.S. Department of Energy. The work conducted by the U.S. Department of Energy Joint Genome Institute is supported by the Office of Science of the U.S. Department of Energy under Contract No. DE-AC02-05CH11231. This research used resources of the Compute and Data Environment for Science (CADES) and the Oak Ridge Leadership Computing Facility at the Oak Ridge National Laboratory.

## Authors' Contributions

X.Y., J.Z., and R.H. conceived and designed the research. R.H., T.M.G., P.Y., and J.C.C. performed the experiments. J.Z., A.S., A.L., M.W., D.L., V.N., J.S., and A.M.B. analyzed the data. J.Z., A.P., and N.J.P. provided the eFP browser instance. J.Z. and R.H. drafted the manuscript. X.Y., J.G.C., W.M., J.C.C., and G.A.T. revised the manuscript. All authors read and approved the manuscript.

## References

[1] Mølmann JA, Junttila O, Johnsen Ø, et al. Effects of red, far‐red and blue light in maintaining growth in latitudinal populations of Norway spruce (*Picea abies*). Plant Cell Environ. 2006;29(2):166–72. 17080632 10.1111/j.1365-3040.2005.01408.x

[2] Quail PH Phytochrome photosensory signalling networks. Nat Rev Mol Cell Biol. 2002;3(2):85. 11836510 10.1038/nrm728

[3] OuYang F, Mao J-F, Wang J, et al. Transcriptome analysis reveals that red and blue light regulate growth and phytohormone metabolism in Norway spruce [*Picea abies* (L.) Karst.]. PLoS One. 2015;10(8):e0127896. 26237749 10.1371/journal.pone.0127896 PMC4523189

[4] Briggs WR, Olney MA Photoreceptors in plant photomorphogenesis to date. Five phytochromes, two cryptochromes, one phototropin, and one superchrome. Plant Physiol. 2001;125(1):85–8. 11154303 10.1104/pp.125.1.85 PMC1539332

[5] Lin C, Shalitin D Cryptochrome structure and signal transduction. Annu Rev Plant Biol. 2003;54(1):469–96. 14503000 10.1146/annurev.arplant.54.110901.160901

[6] Briggs W, Beck C, Cashmore A, et al. The phototropin family of photoreceptors. Plant Cell. 2001;13(5):993–7. 11424903 10.1105/tpc.13.5.993 PMC1464709

[7] Nagy F, Schäfer E Phytochromes control photomorphogenesis by differentially regulated, interacting signaling pathways in higher plants. Annu Rev Plant Biol. 2002;53(1):329–55. 12221979 10.1146/annurev.arplant.53.100301.135302

[8] Fan X-X, Xu Z-G, Liu X-Y, et al. Effects of light intensity on the growth and leaf development of young tomato plants grown under a combination of red and blue light. Sci Hort. 2013;153:50–5.

[9] Rossel JB, Wilson IW, Pogson BJ Global changes in gene expression in response to high light in *Arabidopsis*. Plant Physiol. 2002;130(3):1109–20. 12427978 10.1104/pp.005595 PMC166632

[10] Zavala J, Ravetta D Allocation of photoassimilates to biomass, resin and carbohydrates in *Grindelia chiloensis* as affected by light intensity. Field Crops Res. 2001;69(2):143–9.

[11] Yang X, Cushman JC, Borland AM, et al. A roadmap for research on crassulacean acid metabolism (CAM) to enhance sustainable food and bioenergy production in a hotter, drier world. New Phytol. 2015;207(3):491–504. 26153373 10.1111/nph.13393

[12] Borland AM, Hartwell J, Weston DJ, et al. Engineering crassulacean acid metabolism to improve water-use efficiency. Trends Plant Sci. 2014;19(5):327–38. 24559590 10.1016/j.tplants.2014.01.006 PMC4065858

[13] Yang X, Hu R, Yin H, et al. The *Kalanchoë* genome provides insights into convergent evolution and building blocks of crassulacean acid metabolism. Nat Commun. 2017;8(1):1899. 29196618 10.1038/s41467-017-01491-7 PMC5711932

[14] Borland AM, Wullschleger SD, Weston DJ, et al. Climate‐resilient agroforestry: physiological responses to climate change and engineering of crassulacean acid metabolism (CAM) as a mitigation strategy. Plant Cell Environ. 2015;38(9):1833–49. 25366937 10.1111/pce.12479

[15] Hartwell J, Dever LV, Boxall SF Emerging model systems for functional genomics analysis of crassulacean acid metabolism. Curr Opin Plant Biol. 2016;31:100–8. 27082281 10.1016/j.pbi.2016.03.019

[16] Dodd AN, Borland AM, Haslam RP, et al. Crassulacean acid metabolism: plastic, fantastic. J Exp Bot. 2002;53(369):569–80. 11886877 10.1093/jexbot/53.369.569

[17] Grams TE, Thiel S High light‐induced switch from C 3‐photosynthesis to crassulacean acid metabolism is mediated by UV‐A/blue light. J Exp Bot. 2002;53(373):1475–83. 12021295

[18] Ceusters J, Borland AM, Godts C, et al. Crassulacean acid metabolism under severe light limitation: a matter of plasticity in the shadows?. J Exp Bot. 2011;62(1):283–91. 20861137 10.1093/jxb/erq264

[19] Ceusters J, Borland AM, Taybi T, et al. Light quality modulates metabolic synchronization over the diel phases of crassulacean acid metabolism. J Exp Bot. 2014;65(13):3705–14. 24803500 10.1093/jxb/eru185 PMC4085966

[20] Kornas A, Fischer-Schliebs E, Lüttge U, et al. Adaptation of the obligate CAM plant *Clusia alata* to light stress: metabolic responses. J Plant Physiol. 2009;166(17):1914–22. 19592134 10.1016/j.jplph.2009.06.005

[21] Miszalski Z, Kornas A, Rozpądek P, et al. Independent fluctuations of malate and citrate in the CAM species *Clusia hilariana* Schltdl. under low light and high light in relation to photoprotection. J Plant Physiol. 2013;170(5):453–8. 23253483 10.1016/j.jplph.2012.11.005

[22] Klepikova AV, Kasianov AS, Gerasimov ES, et al. A high resolution map of the *Arabidopsis thaliana* developmental transcriptome based on RNA-seq profiling. Plant J. 2016;88(6):1058–70. 27549386 10.1111/tpj.13312

[23] Benedito VA, Torres-Jerez I, Murray JD, et al. A gene expression atlas of the model legume *Medicago truncatula*. Plant J. 2008;55(3):504–13. 18410479 10.1111/j.1365-313X.2008.03519.x

[24] Matas AJ, Yeats TH, Buda GJ, et al. Tissue- and cell-type specific transcriptome profiling of expanding tomato fruit provides insights into metabolic and regulatory specialization and cuticle formation. Plant Cell. 2011;23(11):3893–910. 22045915 10.1105/tpc.111.091173 PMC3246317

[25] Ramírez-González RH, Borrill P, Lang D, et al. The transcriptional landscape of polyploid wheat. Science. 2018;361(6403):eaar6089. 30115782 10.1126/science.aar6089

[26] Sibout R, Proost S, Hansen BO, et al. Expression atlas and comparative coexpression network analyses reveal important genes involved in the formation of lignified cell wall in *Brachypodium distachyon*. New Phytol. 2017;215(3):1009–25. 28617955 10.1111/nph.14635

[27] The *Kalanchoë* eFP Browser. http://bar.utoronto.ca/efp_kalanchoe/cgi-bin/efpWeb.cgi. Accessed 11 February 2020.

[28] Li Q, Kubota C Effects of supplemental light quality on growth and phytochemicals of baby leaf lettuce. Environ Exp Bot. 2009;67(1):59–64.

[29] Fukuda N, Fujita M, Ohta Y, et al. Directional blue light irradiation triggers epidermal cell elongation of abaxial side resulting in inhibition of leaf epinasty in geranium under red light condition. Sci Hort. 2008;115(2):176–82.

[30] Kitazaki K, Fukushima A, Nakabayashi R, et al. Metabolic reprogramming in leaf lettuce grown under different light quality and intensity conditions using narrow-band LEDs. Sci Rep. 2018;8(1):7914. 29784957 10.1038/s41598-018-25686-0 PMC5962576

[31] Li C-X, Xu Z-G, Dong R-Q, et al. An RNA-seq analysis of grape plantlets grown in vitro reveals different responses to blue, green, red LED light, and white fluorescent light. Front Plant Sci. 2017;8:78. 28197159 10.3389/fpls.2017.00078 PMC5281588

[32] Tardu M, Dikbas UM, Baris I, et al. RNA-seq analysis of the transcriptional response to blue and red light in the extremophilic red alga, *Cyanidioschyzon merolae*. Funct Integr Genomics. 2016;16(6):657–69. 27614431 10.1007/s10142-016-0521-0

[33] Hao X, Li L, Hu Y, et al. Transcriptomic analysis of the effects of three different light treatments on the biosynthesis of characteristic compounds in the tea plant by RNA-Seq. Tree Genet Genome. 2016;12(6):118.

[34] Sellaro R, Hoecker U, Yanovsky M, et al. Synergism of red and blue light in the control of *Arabidopsis* gene expression and development. Curr Biol. 2009;19(14):1216–20. 19559617 10.1016/j.cub.2009.05.062 PMC2730174

[35] Pazhamala LT, Purohit S, Saxena RK, et al. Gene expression atlas of pigeonpea and its application to gain insights into genes associated with pollen fertility implicated in seed formation. J Exp Bot. 2017;68(8):2037–54. 28338822 10.1093/jxb/erx010 PMC5429002

[36] Kudapa H, Garg V, Chitikineni A, et al. The RNA-Seq-based high resolution gene expression atlas of chickpea (*Cicer arietinum* L.) reveals dynamic spatio-temporal changes associated with growth and development. Plant Cell Environ. 2018;41(9):2209–25. 29637575 10.1111/pce.13210

[37] Kurepin LV, Emery RN, Pharis RP, et al. The interaction of light quality and irradiance with gibberellins, cytokinins and auxin in regulating growth of *Helianthus annuus* hypocotyls. Plant Cell Environ. 2007;30(2):147–55. 17238906 10.1111/j.1365-3040.2006.01612.x

[38] Zhang Z, Ji R, Li H, et al. Constans-like 7 (COL7) is involved in phytochrome B (phyB)-mediated light-quality regulation of auxin homeostasis. Mol Plant. 2014;7(9):1429–40. 24908267 10.1093/mp/ssu058

[39] Gubler F, Hughes T, Waterhouse P, et al. Regulation of dormancy in barley by blue light and after-ripening: effects on abscisic acid and gibberellin metabolism. Plant Physiol. 2008;147(2):886–96. 18408047 10.1104/pp.107.115469 PMC2409010

[40] Nusinow DA, Helfer A, Hamilton EE, et al. The ELF4-ELF3-LUX complex links the circadian clock to diurnal control of hypocotyl growth. Nature. 2011;475(7356):398–402. 21753751 10.1038/nature10182 PMC3155984

[41] Lu SX, Knowles SM, Andronis C, et al. Circadian clock associated1 and late elongated hypocotyl function synergistically in the circadian clock of *Arabidopsis*. Plant Physiol. 2009;150(2):834–43. 19218364 10.1104/pp.108.133272 PMC2689956

[42] Kikis EA, Khanna R, Quail PH ELF4 is a phytochrome-regulated component of a negative-feedback loop involving the central oscillator components CCA1 and LHY. Plant J. 2005;44(2):300–13. 16212608 10.1111/j.1365-313X.2005.02531.x

[43] Hazen SP, Schultz TF, Pruneda-Paz JL, et al. Lux arrhythmo encodes a Myb domain protein essential for circadian rhythms. Proc Natl Acad Sci U S A. 2005;102(29):10387–92. 16006522 10.1073/pnas.0503029102 PMC1177380

[44] Lee HG, Mas P, Seo PJ MYB96 shapes the circadian gating of ABA signaling in *Arabidopsis*. Sci Rep. 2016;6:17754. 26725725 10.1038/srep17754 PMC4698719

[45] Muchapirei CI, Valentine S-L, Roden LC Plant circadian networks and responses to the environment. Functional Plant Biol. 2018;45(4):393–9. 10.1071/FP17150 32290979

[46] Facella P, Lopez L, Carbone F, et al. Diurnal and circadian rhythms in the tomato transcriptome and their modulation by cryptochrome photoreceptors. PLoS One. 2008;3(7):e2798. 18665253 10.1371/journal.pone.0002798 PMC2474677

[47] Joseph MP, Papdi C, Kozma-Bognar L, et al. The *Arabidopsis* zinc finger protein3 interferes with abscisic acid and light signaling in seed germination and plant development. Plant Physiol. 2014;165(3):1203–20. 24808098 10.1104/pp.113.234294 PMC4081332

[48] Chrispeels HE, Oettinger H, Janvier N, et al. *AtZFP1*, encoding *Arabidopsis thaliana* C2H2 zinc-finger protein 1, is expressed downstream of photomorphogenic activation. Plant Mol Biol. 2000;42(2):279–90. 10794528 10.1023/a:1006352809700

[49] Shimizu M, Kato H, Ogawa T, et al. Sigma factor phosphorylation in the photosynthetic control of photosystem stoichiometry. Proc Natl Acad Sci U S A. 2010;107(23):10760–4. 20498041 10.1073/pnas.0911692107 PMC2890857

[50] Tsunoyama Y, Morikawa K, Shiina T, et al. Blue light specific and differential expression of a plastid σ factor, Sig5 in *Arabidopsis thaliana*. FEBS Lett. 2002;516(1):225–8. 11959137 10.1016/s0014-5793(02)02538-3

[51] Onda Y, Yagi Y, Saito Y, et al. Light induction of *Arabidopsis SIG1* and *SIG5* transcripts in mature leaves: differential roles of cryptochrome 1 and cryptochrome 2 and dual function of SIG5 in the recognition of plastid promoters. Plant J. 2008;55(6):968–78. 18532976 10.1111/j.1365-313X.2008.03567.x

[52] Xu D, Jiang Y, Li J, et al. BBX21, an *Arabidopsis* B-box protein, directly activates HY5 and is targeted by COP1 for 26S proteasome-mediated degradation. Proc Natl Acad Sci U S A. 2016;113(27):7655–60. 27325768 10.1073/pnas.1607687113 PMC4941485

[53] Kutter C, Schob H, Stadler M, et al. MicroRNA-mediated regulation of stomatal development in *Arabidopsis*. Plant Cell. 2007;19(8):2417–29. 17704216 10.1105/tpc.107.050377 PMC2002609

[54] Schweer J, Geimer S, Meurer J, et al. *Arabidopsis* mutants carrying chimeric sigma factor genes reveal regulatory determinants for plastid gene expression. Plant Cell Physiol. 2009;50(7):1382–6. 19439445 10.1093/pcp/pcp069

[55] Liu X, Chen CY, Wang KC, et al. Phytochrome interacting factor3 associates with the histone deacetylase HDA15 in repression of chlorophyll biosynthesis and photosynthesis in etiolated *Arabidopsi*s seedlings. Plant Cell. 2013;25(4):1258–73. 23548744 10.1105/tpc.113.109710 PMC3663266

[56] Nagel DH, Doherty CJ, Pruneda-Paz JL, et al. Genome-wide identification of CCA1 targets uncovers an expanded clock network in *Arabidopsis*. Proc Natl Acad Sci U S A. 2015;112(34):E4802–10. 26261339 10.1073/pnas.1513609112 PMC4553765

[57] Rawat R, Schwartz J, Jones MA, et al. REVEILLE1, a Myb-like transcription factor, integrates the circadian clock and auxin pathways. Proc Natl Acad Sci U S A. 2009;106(39):16883–8..b 19805390 10.1073/pnas.0813035106 PMC2757846

[58] Zhang X, Chen Y, Wang ZY, et al. Constitutive expression of CIR1 (RVE2) affects several circadian‐regulated processes and seed germination in *Arabidopsis*. Plant J. 2007;51(3):512–25. 17587236 10.1111/j.1365-313X.2007.03156.x

[59] Kuno N, Moller SG, Shinomura T, et al. The novel MYB protein early-phytochrome-responsive1 is a component of a slave circadian oscillator in *Arabidopsis*. Plant Cell. 2003;15(10):2476–88. 14523250 10.1105/tpc.014217 PMC197310

[60] Wu TD, Nacu S Fast and SNP-tolerant detection of complex variants and splicing in short reads. Bioinformatics. 2010;26(7):873–81. 20147302 10.1093/bioinformatics/btq057 PMC2844994

[61] Liao Y, Smyth GK, Shi W featureCounts: an efficient general purpose program for assigning sequence reads to genomic features. Bioinformatics. 2014;30(7):923–30. 24227677 10.1093/bioinformatics/btt656

[62] Li B, Dewey CN RSEM: accurate transcript quantification from RNA-Seq data with or without a reference genome. BMC Bioinformatics. 2011;12(1):323. 21816040 10.1186/1471-2105-12-323 PMC3163565

[63] Love MI, Huber W, Anders S Moderated estimation of fold change and dispersion for RNA-seq data with DESeq2. Genome Biol. 2014;15(12):550. 25516281 10.1186/s13059-014-0550-8 PMC4302049

[64] Thimm O, Bläsing O, Gibon Y, et al. MAPMAN: a user‐driven tool to display genomics data sets onto diagrams of metabolic pathways and other biological processes. Plant J. 2004;37(6):914–39. 14996223 10.1111/j.1365-313x.2004.02016.x

[65] Maere S, Heymans K, Kuiper M BiNGO: a Cytoscape plugin to assess overrepresentation of gene ontology categories in biological networks. Bioinformatics. 2005;21(16):3448–9. 15972284 10.1093/bioinformatics/bti551

[66] Langfelder P, Horvath S WGCNA: an R package for weighted correlation network analysis. BMC Bioinformatics. 2008;9(1):559. 19114008 10.1186/1471-2105-9-559 PMC2631488

[67] Dubois M, Gilles KA, Hamilton JK, et al. Colorimetric method for determination of sugars and related substances. Anal Chem. 1956;28(3):350–6.

[68] Zhang J, Hu R, Garcia T, et al. Supporting data for “Light-responsive expression atlas reveals the effects of light quality and intensity in *Kalanchoë fedtschenkoi*, a plant with crassulacean acid metabolism.”. GigaScience Database. 2020 10.5524/100706 PMC7058158 32135007

