# Supplementary material for: Light-responsive expression atlas reveals the effects of light quality and intensity in Kalanchoë fedtschenkoi, a plant with crassulacean acid metabolism
Source: Gigascience. 2020 Mar 5;9(3):giaa018. doi: 10.1093/gigascience/giaa018 (PMC7058158; doi:10.1093/gigascience/giaa018)
Supplement: giaa018_GIGA-D-19-00095_Original_Submission [file giaa018_giga-d-19-00095_original_submission.pdf]

GigaScience

Expression atlas and co-expression network reveal effects of light quality and intensity in Kalanchoë fedtschenkoi, a plant with crassulacean acid metabolism

--Manuscript Draft--

Manuscript Number:	GIGA-D-19-00095
Full Title:	Expression atlas and co-expression network reveal effects of light quality and intensity in Kalanchoë fedtschenkoi, a plant with crassulacean acid metabolism
Article Type:	Research
Funding Information:	Genomic Science Program (DE-SC0008834) — Dr Xiaohan Yang<br>Community Science Program (503025) — Dr Xiaohan Yang
Abstract:	Background: Crassulacean acid metabolism (CAM), a specialized mode of photosynthesis, enables plant adaptation to water-limited environments and improves photosynthetic efficiency via a carbon concentrating mechanism. Kalanchoë fedtschenkoi is an obligate CAM model featuring a relatively small genome and easy stable transformation. But the molecular responses of regulation by light quality and intensity in CAM plants is still unknown.<br>Results: Here we present a genome-wide expression atlas of K. fedtschenkoi plants grown under 12h/12h photoperiod with different light quality (blue, red, far-red, white light) and intensity (0, 150, 250 and 1000 μmol m–2 s–1) based on RNA-Seq performed for mature leaf samples collected at dawn (2-h before the starting of lighting period) and dusk (2-h before the dark period). An eFP web browser was created for easy access of the gene expression data. Based on the expression atlas, we constructed a light-responsive co-expression network to reveal the potential regulatory relationships in K. fedtschenkoi. Furthermore, CAM-related sub-networks were highlighted to showcase genes relevant to CAM pathway, circadian clock and stomatal movement.<br>Conclusions: This study provides a novel genomics resource for investigating the molecular mechanism underlying the light regulation of physiology and metabolism in CAM plants.
Corresponding Author:	Jin Zhang<br>Oak Ridge National Laboratory<br>oak ridge, TN UNITED STATES
Corresponding Author Secondary Information:	
Corresponding Author's Institution:	Oak Ridge National Laboratory
Corresponding Author's Secondary Institution:	
First Author:	Jin Zhang
First Author Secondary Information:	
Order of Authors:	Jin Zhang
	Rongbin Hu
	Avinash Sreedasyam
	Anna Lipzen
	Mei Wang
	Travis Garcia
	Pradeep Yerramsetty
	Degao Liu
	Vivian Ng

Powered by Editorial Manager® and ProduXion Manager® from Aries Systems Corporation

	Jeremy Schmutz
	John Cushman
	Anne Borland
	Asher Pasha
	Nicholas Provart
	Jin-Gui Chen
	Wellington Muchero
	Gerald Tuskan
	Xiaohan Yang
**Order of Authors Secondary Information:**	
**Additional Information:**	
**Question**	**Response**
Are you submitting this manuscript to a special series or article collection?	No
**Experimental design and statistics**<br><br>Full details of the experimental design and statistical methods used should be given in the Methods section, as detailed in our Minimum Standards Reporting Checklist. Information essential to interpreting the data presented should be made available in the figure legends.<br><br>Have you included all the information requested in your manuscript?	Yes
**Resources**<br><br>A description of all resources used, including antibodies, cell lines, animals and software tools, with enough information to allow them to be uniquely identified, should be included in the Methods section. Authors are strongly encouraged to cite Research Resource Identifiers (RRIDs) for antibodies, model organisms and tools, where possible.<br><br>Have you included the information requested as detailed in our Minimum Standards Reporting Checklist?	Yes

**Availability of data and materials**<br><br>All datasets and code on which the conclusions of the paper rely must be either included in your submission or deposited in publicly available repositories (where available and ethically appropriate), referencing such data using a unique identifier in the references and in the “Availability of Data and Materials” section of your manuscript.<br><br>Have you have met the above requirement as detailed in our Minimum Standards Reporting Checklist?	Yes

Powered by Editorial Manager® and ProduXion Manager® from Aries Systems Corporation

Manuscript
Click here to download Manuscript Jin et al Kalanchoe_Light_mainText_draft5.5_gigaSci_Fig.docx

Article type: Research Article

Expression atlas and co-expression network reveal effects of light quality and intensity in *Kalanchoë fedtschenkoi*, a plant with crassulacean acid metabolism

Jin Zhang[1,2,a], Rongbin Hu[1,a], Avinash Sreedasyam[3], Anna Lipzen[4], Mei Wang[4], Travis Garcia[5], Pradeep Yerramsetty[5], Degao Liu[1], Vivian Ng[4], Jeremy Schmutz[3,4], John C. Cushman[5], Anne M. Borland[1,6], Asher Pasha[7], Nicholas J. Provart[7], Jin-Gui Chen[1,2], Wellington Muchero[1,2], Gerald A. Tuskan[1,2], Xiaohan Yang[1,2,*]

[1] Biosciences Division, Oak Ridge National Laboratory, Oak Ridge, TN 37831-6422, USA; [2] The Center for Bioenergy Innovation, Oak Ridge National Laboratory, Oak Ridge, TN 37831, USA; [3] HudsonAlpha Institute for Biotechnology, 601 Genome Way, Huntsville, AL 35801, USA; [4] US Department of Energy Joint Genome Institute, 2800 Mitchell Drive, Walnut Creek, CA 94598, USA; [5] Department of Biochemistry and Molecular Biology, University of Nevada, Reno, NV 89557, USA; [6] School of Natural and Environmental Science, Newcastle University, Newcastle upon Tyne NE1 7RU, UK; [7] Department of Cell and Systems Biology, Centre for the Analysis of Genome Evolution and Function, University of Toronto, Toronto, ON M5S 3B2, Canada

[a] These authors contributed equally to this manuscript.
*Correspondence: Xiaohan Yang (yangx@ornl.gov) Tel +1 865 241 6895; fax +1 865 576 9939

Emails: Jin Zhang (zhangj1@ornl.gov), Rongbin Hu (hu.rongbin@gmail.com), Avinash Sreedasyam (asreedasyam@hudsonalpha.org), Anna Lipzen (alipzen@lbl.gov), Mei Wang (mwang@lbl.gov), Travis Garcia (tgarcia2@unr.edu), Pradeep Yerramsetty (ypradeepkalyan@gmail.com), Degao Liu (liudegao909@gmail.com), Vivian Ng (vng@lbl.gov), Jeremy Schmutz (jschmutz@hudsonalpha.com), John C. Cushman (jcushman@unr.edu), Anne M. Borland (anne.borland@newcastle.ac.uk), Asher Pasha (asher.pasha@utoronto.ca), Nicholas J. Provart (nicholas.provart@utoronto.ca), Jin-Gui Chen (chenj@ornl.gov), Wellington Muchero (mucherow@ornl.gov), Gerald A. Tuskan (tuskanga@ornl.gov), Xiaohan Yang (yangx@ornl.gov)

Figures: 7 figures (in color).
Supplementary Data: 8 supplementary figures and 9 supplementary tables.

Abstract

Background: Crassulacean acid metabolism (CAM), a specialized mode of photosynthesis, enables plant adaptation to water-limited environments and improves photosynthetic efficiency via a carbon concentrating mechanism. *Kalanchoë fedtschenkoi* is an obligate CAM model featuring a relatively small genome and easy stable transformation. But the molecular responses of regulation by light quality and intensity in CAM plants is still unknown.

Results: Here we present a genome-wide expression atlas of *K. fedtschenkoi* plants grown under 12h/12h photoperiod with different light quality (blue, red, far-red, white light) and intensity (0, 150, 250 and 1000 μmol m^{-2} s^{-1}) based on RNA-Seq performed for mature leaf samples collected at dawn (2-h before the starting of lighting period) and dusk (2-h before the dark period). An eFP web browser was created for easy access of the gene expression data. Based on the expression atlas, we constructed a light-responsive co-expression network to reveal the potential regulatory relationships in *K. fedtschenkoi*. Furthermore, CAM-related sub-networks were highlighted to showcase genes relevant to CAM pathway, circadian clock and stomatal movement.

Conclusions: This study provides a novel genomics resource for investigating the molecular mechanism underlying the light regulation of physiology and metabolism in CAM plants.

Keywords: eFP browser; gene atlas; transcriptome; *Kalanchoë fedtschenkoi*; crassulacean acid metabolism (CAM)

Background

Sunlight is a critical energy resource for plant growth and development, which function as an important input signal for circadian clock, stomatal movement and photosynthesis pathway. The light spectra that affect plant photosynthesis are UV-A/blue, red and far-red lights [1, 2]. Blue light, with wavelength of 400 to 500 nm, has relative higher energy than red light (wavelength from 600 to 700 nm) and far-red light (wavelength above 700 nm) [1, 3]. There are three types of photoreceptors (i.e., cryptochromes, phototropins, phytochromes that perform important roles in plant light response [2, 4]. Cryptochromes and phototropins have been identified as important photoreceptors of UV-A/blue light [2, 5, 6]. Phytochromes are known to play a role in detecting red and far-red spectra [2, 7]. In addition to the light quality, light intensity is another essential factor that affects plant growth and development, where both too much light or low light intensity can cause stress, including serious damage to photosynthetic apparatus under excess light exposure and limited photosynthetic activity with insufficient light input [8-10].

Plants using crassulacean acid metabolism (CAM) pathway for photosynthesis show enhanced water-use efficiency (WUE) and heat/drought stress tolerance in comparison with C_3 and C_4 photosynthesis plants [11, 12]. CAM pathway that regulated by circadian clock has two major features: (1) a carboxylation process that takes place at night where stomata are open for nocturnal CO_2 fixation and accumulation of malic acid in the vacuole and (2) a decarboxylation process that occurs during the daytime where CO_2 is released from malate for refixation via ribulose-1,5-bisphosphate carboxylase/oxygenase (RuBISCO) mediated photosynthesis, along with the stomata closure for reduced water transpiration [11, 13, 14]. *Kalanchoë fedtschenkoi* is a model dicot CAM species, featuring a relatively small genome and a facile stable transformation system [11, 15]. The genome of *K. fedtschenkoi* was recently sequenced and annotated [13], providing a foundation for CAM genomics research. Through comparative and evolutionary genomics analyses, Yang, Hu [13] revealed convergent signatures in diel gene expression pattern and protein sequences underlying independent emergences of CAM from C_3 ancestor, providing new insights into CAM evolution. But the complex regulatory mechanisms of CAM pathway under various light conditions are still largely unknown.

The temporal separation of C3 and C4 carboxylation processes that defines CAM provides plasticity for optimizing carbon gain and water use in response to changing environmental

conditions by extending or curtailing the period of net CO_2 uptake over a 24 h period [16]. Light intensity (photosynthetic photon flux density, PPFD) and light quality are critical factors for determining the magnitude of CAM which implies cardinal roles for the light reactions of photosynthesis and for different photoreceptors in achieving metabolic and circadian synchronization of carboxylation processes across the diel cycle. In some facultative CAM species, high light intensity can trigger the switch from C_3-photosynthesis to CAM, which is mediated by a UV-A/blue light receptor [17]. Metabolic and physiological adaptation in constitutive CAM species plants to light quantity and quality has been reported previously [18-21]. For instance, Ceusters, Borland [18] reported physiological and metabolic responses under severe light stress under short- and long-term treatments. They also reported metabolic synchronization under different light spectrum, i.e., blue, green and red light in an obligate CAM species *Aechmea 'Maya'* [19]. Exposure of a woody CAM species *Clusia hilariana* to low- or high-light affects the production of malate and citrate [21]. However, gene regulation at the transcriptional level in CAM plants in response to various spectral light qualities and intensity has not been reported yet.

High-throughput next-generation sequencing has been widely applied to genome-wide expression analysis. As a new type of web-based tool, a genome-wide atlas of gene expression can provide comprehensive gene expression profiles in different tissues or different development stages. To date, gene expression atlases have been established for several C_3 photosynthesis species, including dicot *Arabidopsis [22]*, *Medicago* [23] and tomato [24] and monocot wheat [25] and *Brachypodium* [26]. However, a genome-wide expression atlas has not been created for CAM plants yet. In addition, gene expression patterns and gene modules associated with plant response to light quality and light intensity are largely unknown, especially for CAM plants.

To address these limitations, we performed transcriptome-sequencing (RNA-Seq) of mature *K. fedtschenkoi* leaf samples collected at dawn (i.e., 2-h before the starting of lighting period) and dusk (2-h before the dark period) from plants grown under 12h/12h photoperiod with different light quality (i.e., blue, red, far-red, white light) and intensity (0, 150 and 1000 μmol m^{-2} s^{-1}). Based on our analysis of the RNA-Seq data, we generated a comprehensive light-responsive gene expression atlas for this obligate CAM species. We also constructed a genome-wide co-expression network based on the light-responsive gene expression atlas. As the first light-responsive gene atlas and co-expression network for CAM plants, this study provides unprecedented novel

genomics resource for investigating the molecular mechanism underlying the light regulation of biological processes in CAM plants.

Data Description

A total of 42 libraries (7 light conditions × 2 time points × 3 biological replicates) were constructed and performed RNA-seq independently. In total, we obtained ~138 Gb of high-quality data from the 42 libraries, with an average size of ~3.28 Gb per library (Supplementary Table S1).

Analyses

Light-responsive expression atlas for *K. fedtschenkoi*

To obtain a comprehensive light-responsive gene-expression atlas of the CAM plant *K. fedtschenkoi*, we cultured plants under control condition (white light with 250 μmol m^{-2} s^{-1} intensity), various light quality including blue light, red light, far-red light, and different light intensity, including dark grown, low light intensity and high light intensity (Supplementary Table S1). As CAM pathway is regulated by circadian clock, we compared if circadian rhythm-related processes were also affected by different light conditions. The samples were collected at two time points [dawn (2 h before light period) and dusk (2 h before dark period)] for each light condition. To provide easy access to the expression data, we created a *Kalanchoë* light-responsive eFP browser instance (http://bar.utoronto.ca/~asher/efp_kalanchoe/cgi-bin/efpWeb.cgi), which provides a color-coding tissue visualization in an image corresponding to the average gene expression level (Fig. 1).

According to the correlation analysis of the biological replicates, four obvious outlier samples (i.e., white light dusk rep 2, red light dawn rep 2, far red-light dusk rep 3 and dark grown dusk rep 1) were excluded for further analysis. And the expression distribution of the left 38 libraries was similar (Supplementary Fig. S1). The Pearson correlation analysis and principle component analysis showed that the biological replicates of each treatment group were closely clustered, indicating the high reproducibility and reliability of our RNA-seq data (Fig. 2 and Supplementary

Fig. S1). The principal component 1 (PC1) and PC2 explained 31.2% and 27.8% of the variance in the expression data, respectively. As expected, the samples collected at dawn and dusk were grouped separately under different light quality and light intensity except dark grown (two dash line ellipses in Fig. 2b) and the expression variation of samples under various light conditions were stronger at dawn than that at dusk.

Differentially expressed genes (DEGs) regulated by light quality and light intensity

As shown in Fig. 2a, we performed a transcriptomic comparative analysis for screening of DEGs by using two different strategies, i.e., time comparison and light condition comparison. The time comparison was defined as the comparison between two samples collected at two different time points (i.e., dawn and dusk) under each light condition, i.e., dusk-vs-dawn (comparisons C1_1 to C1_7, Supplementary Table S2). The light condition comparison reflects a comparison between treatments and control at the same sample collection time point, i.e., blue light/red light/far-red light-vs-white light at dawn or dusk (comparisons C2_1 to C2_6) for light quality and low light/high light-vs-normal light condition (white light, control) at dawn or dusk (comparisons C3_1 to C3_6) for light intensity, respectively (Fig. 2a and Supplementary Table S2).

Under normal light condition (white light, control), 5925 DEGs were identified between dusk and dawn. Of these DEGs, 3257 and 2668 genes were down- and up-regulated, respectively, at dusk compared to dawn (C1_1 in Fig. 2). For different light quality, the DEG number between dusk and dawn were increased under blue light (6697 DEGs) and far-red light (6200 DEGs) but reduced under red light (4909 DEGs). For different light intensity, both the low intensity and high intensity enhanced the gene differential expression between dusk and dawn. Compare to white light, the DEG number was 1.66-fold (9813/5926) and 1.27-fold (7514/5926) higher under low intensity and high intensity, respectively, and the induction of up-regulated DEGs (1.89-fold and 1.50-fold, respectively) was stronger than that of down-regulated DEGs (1.46-fold and 1.08-fold, respectively). Interestedly, under dark grown condition, only 908 DEGs (591 up and 317 down) were identified between dusk and dawn, which was significantly less than that under various light conditions (Fig. 2a and Supplementary Table S2).

Under different light spectrum, a total of 2669 DEGs between dusk and dawn were shared by the four light types (i.e., white light, blue light, red light and far-red light), indicating these genes

may be play essential roles in response to changes in light quality (Fig. 3a-f). For light quality-specific DEGs between dusk and dawn, more genes were specifically differentially expressed under white light (1164 DEGs) than those under blue light, red light and far-red light (1112, 631 and 957 DEGs, respectively). Under various light intensity conditions, 3143 genes were consistently differentially expressed under the three light intensities (white light, low light and high light), suggesting that these genes might play key roles in circadian rhythms and are not affected by light intensity. The light intensity-specific DEGs varied between dusk and dawn across low light (3193 DEGs) and high light (1181 DEGs) conditions.

Light condition comparisons were based on differences between dawn and dusk (Fig. 2a). For the light quality experiment, the number of up-regulated DEGs were greater than that of down-regulated DEGs under blue light, red light and far-red light at both dawn and dusk (cite a figure or table here). Under blue light and far-red light (C2_1 and C2_3, respectively) the number of DEGs at dawn were greater than that at dusk (C2_4 and C2_6, respectively). Only a few DEGs (701 up and 595 down) were identified at dawn under red light (C2_2) (Fig. 2d). For the light intensity experiment, numerous genes were differentially expressed under dark grown at both dawn and dusk. The number of DEGs was significantly greater at dawn than dusk under both low light and high light conditions, and the number of up-regulated genes were greater than that of down-regulated genes at dusk (Fig. 2e).

Although the DEG number in most light quality comparisons were greater at dawn than that at dusk, the overlapped DEGs were fewer at dawn (732 common DEGs, Fig. 3c) than dusk (1102 common DEGs, Fig. 3d) under different light quality. In contrast, the light quality-specific DEGs were greater at dawn than dusk under blue light and far-red light. For different light intensity, more than half of the DEGs under low light and high light were shared at both dawn and dusk (Fig. 3e,f).

Predicted function of DEGs

To explore the functional differences of DEGs induced by various light quality and light intensity treatments, we performed a gene ontology (GO) enrichment analysis of common and light-specific DEGs in response to the light treatments according to the three major GO categories of biological process, molecular function, and cellular component (Fig. 3g; Supplementary Fig. S2-S4; Supplementary Table S3). Among the DEGs between dusk and dawn, the 2699 DEGs that

overlapped across different light quality treatments (Fig. 3a) were enriched in “carbohydrate metabolic process”, “lipid metabolic process”, “metabolic process” and “signal transduction”; whereas the 3143 DEGs that overlapped across different light intensity treatments were enriched in “carbohydrate metabolic process” and “response to endogenous stimulus” (Fig. 3g). When comparing the common DEGs in different light quality or light intensity at dawn and dusk separately (Fig. 3c-f), we found that “generation of precursor metabolites and energy” term was enriched in all the four common DEGs sets of different light quality and light intensity at both dawn and dusk. In contrast, “photosynthesis” term was enriched in common DEGs of light quality at dusk and common DEGs of light intensity at both dawn and dusk (Fig. 3g). While “carbohydrate metabolic process” term was only enriched at dusk time point of different light quality and light intensity (Fig. 3g).

For condition-specific DEGs at different time points or different light conditions, “photosynthesis” term was strongly enriched in dusk-vs-dawn DEGs in red light-specific and high light-specific from light quality comparison and light intensity comparison, respectively (Fig. 3g). This indicates that red light and high light significantly affects photosynthesis changes between dawn and dusk. When different light quality or light intensity treatments were compared to white light control at dawn and dusk, “photosynthesis” term was also enriched in dark grown-vs-white light (Fig. 3c,d) and high light-vs-white light (Fig. 3g) at both dawn and dusk, indicating high light and dark grown strongly affect photosynthesis independent of dawn or dusk sampling.

Clusters of DEGs

To further reveal the expression patterns and functional divergence of DEGs affected by light quality and light intensity, the DEGs identified from previous comparisons were then subjected to a K-means clustering analysis with 20 clusters. As shown in Fig. 4, clusters 1 and 13 showed similar expression pattern with down regulation at both dawn and dusk under dark grown. The DEGs in the two clusters were significantly enriched in “photosynthesis” and “thylakoid” terms. Compare to cluster 13, the down-regulation of DEGs under the dark condition is stronger in cluster 1, which were specifically enriched in “generation of precursor metabolites and energy” term. Although the DEGs in cluster 8 were also down-regulated under the dark condition, they were induced under low light and high light conditions. Oppositely, the DEGs in clusters 9 and 18 were up-regulated under the dark condition, and the DEGs in cluster 5 were slightly up-regulated under

the dark condition but down-regulated by low light and high light. Noticeably, the DEGs in several clusters were always repressed from dawn to dusk changes under different light quality and light intensity treatments. For instance, the DEGs in clusters 2, 3, 12 and 16 showed high expression at dawn and low expression at dusk under all the tested light quality (white light, blue light, red light and far-red light) and light intensity (low light and high light) treatments except for the dark treatment. And among these clusters, clusters 2, 3, and 12 showed down-regulation under the dark condition, while cluster 16 showed up-regulation. Similarly, clusters 4, 7, 11, 20 were down-regulated at dawn and were up-regulated at dusk. And the expression induction of DEGs in cluster 7 was enhanced under light intensity conditions than light quality conditions. Compare to the time point sensitive genes, DEGs in clusters 5, 6, 8, and 18 showed more responsive to light intensity than light quality treatment. Clusters 6 and 8 were up-regulated by low light and high light, whereas clusters 5 and 18 were down-regulated by low light and high light. The DEGs in clusters 6 and 8 were enriched with "lipid metabolic process" (Fig. 4, Supplementary Fig. S5-S8, Supplementary Tables S4-S5).

Co-expression network

To determine the relationship of genes responsive to different light quality and light intensity in CAM plant *K. fedtschenkoi*, we constructed a co-expression network using the DEGs identified from the previous comparisons (Fig. 2). After combining the modules with highly similar expression patterns, a total of 39 co-expression modules were obtained and labelled as different colors (Fig. 5a, merged dynamic panel). The module size ranged from 121 genes (module 'mediumpurple3') to 1312 genes (module 'turquoise') (Supplementary Table S6). The multidimensional scaling (MDS) plot showed that some genes in different modules were clustered together (Fig. 5b), and the cluster and correlation analyses proved that several modules showed high correlation (Fig. 5c,d).

To further explore if the modules with similar expression patterns are also conserved in function, we performed a GO enrichment analysis using genes in each module. Noticeably, several modules with similar expression patterns were enriched in same GO terms. For instance, the genes in modules 'yellow', 'turquoise', 'purple', 'darkorange' and 'sienna3' were distributed in the right bottom corner of the MDS plot and were enriched in "protein modification" terms (Fig. 5b and 5e, Supplementary Table S7). Closely clustered modules 'red', 'blue' and 'skyblue' were enriched in

“translation”, while modules ‘red’, ‘blue’ and ‘black’ were enriched in “catabolic” terms. In addition, modules ‘black’, ‘pink’ and ‘saddlebrown’ were enriched in “lipid metabolic” terms, whereas three modules with relative far distributions (‘darkgrey’, ‘darkturquoise’ and ‘salmon’) were enriched in “photosynthesis” terms. Several modules were enriched in specific biological process although they had similar expression patterns with other modules. For example, module ‘turquoise’ was enriched in “cell communication” terms, although it was also enriched in “protein modification” terms as were modules ‘yellow’, ‘purple’, ‘darkorange’ and ‘sienna3’.

To further understand the response of different pathways in CAM plants under various light quality and light intensity treatments, we extracted sub-networks from the global co-expression network. Here we selected genes related to CAM, circadian clock and stomatal movement [13] as a case study to demonstrate the sub-network. In order to simplify the sub-network, we set a high threshold of Pearson correlation coefficient (|PCC|>0.95 and $p \leq 0.01$) to show the strong co-expression relationships. The genes involved in CAM, circadian clock and stomatal movement pathways were highly associated and were co-expressed with numerous transcription factors (TFs) (Fig. 6), implying the expression of CAM pathway genes may be directly or indirectly regulated by circadian clock TFs. Based on the sub-network, we identified several known and novel TFs that were related with these pathways. For instance, *LHY1* (*Kaladp0066s0115*) was positively co-expressed with *CCA1* (*Kaladp0496s0018*, PCC=0.995), *RVE8* (*Kaladp0577s0020*, PCC=0.993) and *RVE1* (*Kaladp0574s0015*, PCC=0.983); and was negatively co-expressed with *ELF4* (*Kaladp0045s0206*, PCC=-0.978) and *LUX* (*Kaladp0033s0047*, PCC=-0.969). Similarly, *MYB96* (*Kaladp0095s0568*) and *WRKY4* (*Kaladp0096s0082*) were positively co-expressed with *CCA1* and *RVE8* and were negatively co-expressed with *LUX* (Fig. 6 and Supplementary Table S8). In addition, several TFs not previously reported to be associated with CAM were identified in the sub-network, such as *LZF1* (*Kaladp0192s0026*), *SOC1* (*Kaladp0016s0148*), *CDF2* (*Kaladp0009s0042* and *Kaladp0095s0211*), *COL4* (*Kaladp0029s0144*), *ZFP4* (*Kaladp0035s0036*), *ZFP7* (*Kaladp0001s0233*), *SIG1* (*Kaladp0538s0007*), *SIG4* (*Kaladp0515s0145*), and *SIG5* (*Kaladp0055s0328*).

CAM-related genes responsive to light quality and light intensity

To further investigate the CAM-specific response to various light quality and light intensity treatments, the expression patterns of CAM-related genes were analyzed (Fig. 7). The key genes

that are involved in nocturnal CO_2 assimilation and malate storage include *beta-carbonic anhydrase* (*β-CA*), *phosphoenolpyruvate carboxylase* (*PEPC*), *phosphoenolpyruvate carboxylase kinase* (*PPCK*), *NAD(P)-malate dehydrogenase* (*MDH*) and *aluminum-activated malate transporter* (*ALMT*). Among the six *β-CA* genes detected in this study, two *β-CA* genes (*Kaladp0018s0287* and *Kaladp0018s0289*) were highly expressed at dawn under high light compare to other light quality and light intensity treatments. Noticeably, the third *β-CA* gene *Kaladp0034s0051* was consistently induced at dawn and repressed at dusk under various light quality and light intensity treatments, except in the dark treatment. The fourth *β-CA* gene *Kaladp0538s0011* exhibited induction at both dawn and dusk under blue light. One *PEPC* member (*Kaladp0095s0055*) showed relative high expression levels at dusk than dawn under different light quality and light intensity conditions (white light, blue light, red light, far-red light, low light and high light), yet its expression levels at both dawn and dusk were repressed under blue light, red light and far-red light. In contrast, two other PEPC genes (*Kaladp0011s0355* and *Kaladp0011s1355*) showed opposite pattern with high expression at dawn, and the expression of *Kaladp0011s0355* showed strong induction at dawn under red light and blue light. The expression of two kinases PPCKs (*Kaladp0050s0014* and *Kaladp0037s0517*) were repressed under blue light, red light and far-red light. Most of the MDH genes showed relatively high expression at dusk and low expression at dawn, and most of them were repressed by dark grown conditions except one (*Kaladp0095s0052*). Three MDH genes (*Kaladp0058s0569*, *Kaladp0068s0169* and *Kaladp0082s0194*) were induced by low light and high light but repressed under various light quality treatments (Fig. 7 and Supplementary Table S9).

During daytime, the CO_2 release from malate and refixation in *K. fedtschenkoi* is mediated by a series of genes such as *tonoplast dicarboxylate transporter* (*TDT*), *NAD(P)-malic enzyme* [*NAD(P)-ME*], and *pyruvate phosphate dikinase* (*PPDK*). As the first-step transporter for the daytime reactions, *TDT* (*Kaladp0042s0251*) showed an obvious trend of up-regulation at dawn and down-regulation at dusk, and its expression at dawn was stronger under red light and blue light than under the white light control. In contrast, among the members of *NAD-ME* and *NADP-ME*, only one *NADP-ME* (*Kaladp0092s0166*) showed similar expression patterns with TDT, but its expression at dawn were low under blue light and red light. The two PPDK genes (*Kaladp0039s0092* and *Kaladp0076s0229*) were down-regulated at dusk compare to dawn under

each light condition and their expression levels were further down-regulated under blue light, red light and far-red light (Fig. 7 and Supplementary Table S9).

Discussion

Light quality and light intensity are important environmental factors that affect plant growth and development, plant physiology and metabolism [27, 28]. Although the light quality effects at the metabolic and molecular levels have been studied in several plant species [29-33], the genome-wide transcriptomic studies of the effects of light quality and light intensity on CAM species are lacking. It has been demonstrated that the low-fluorescence red and blue light modulated the diel metabolic synchronization in an obligate CAM species *Aechmea* 'Maya' [19]. However, the regulatory mechanisms underpinning the metabolic reprogramming in CAM species remains unknown.

In this study, we created a comprehensive light-responsive gene-expression atlas for *K. fedtschenkoi*, which is the first genome-wide expression atlas for a CAM plant. The eFP browser provides a useful web interface for easy data access, facilitating comparative and functional genomics research. Furthermore, the RNA-Seq data was analyzed by pairwise comparisons between different light conditions and different time points to identify DEGs in *K. fedtschenkoi*. These DEGs were then subject to clustering and co-expression analyses. A similar approach was effectively utilized to discover the regulatory networks in *Brachypodium distachyon* [26], pigeon pea [34], and chickpea [35]. Combined with functional analysis, such as GO enrichment analysis, we found that the overlapped DEGs at dusk were mainly involved in "carbohydrate metabolic" and "response to endogenous stimulus" processes, consistent with previous studies showing that light quality affects the regulation of endogenous hormone stimulus such as gibberellin, auxins, cytokinins and abscisic acid [3, 36-38].

K. fedtschenkoi is a new model plant species for CAM functional genomics research [13, 15]. In this study, we highlighted a sub-network of CAM-related genes, e.g., *LHY1* was positively co-expressed with *CCA1*, *RVE1* and *RVE8*, but it was negatively co-expressed with *ELF4* and *LUX* (Fig. 6). In *Arabidopsis*, ELF4, ELF3 and LUX can form an ELF4-ELF3-LUX protein complex (the evening complex), which is regulated by the light and clock [39]. MYB-related protein CCA1

and LHY1 can form homodimers and regulate the expression of evening-element-containing genes [40]. There is a negative-feedback loop among these transcription factors. EFL4 and LUX are required for the red-light induction of *CCA1* and *LHY1*, whereas CCA1 and LHY1 negatively regulate the expression of ELF4 [41] and LUX [42]. The co-expression relationships of these TFs in *K. fedtschenkoi* reported here indicate that the circadian rhythm regulatory mechanism among these genes are conserved in *K. fedtschenkoi* and *Arabidopsis*.

MYB96, a TF involved in circadian clock in *Arabidopsis*, was also identified in our sub-network that was positively co-expressed with *CCA1* and *RVE8* (Fig. 6). As a key regulator connecting circadian clock and environment, *MYB96* is induced by high level of ABA and can directly bind to the promoter of *TOC1* to active its expression. It is directly regulated by CCA1 through multiple CCA1-binding sites (CBS: AAAATCT) and evening elements (EE: AAATATCT). Interestingly, CCA1 bind to the promoter of MYB96 at dawn but not at dusk [43, 44]. These findings suggest that our constructed co-expression network is reliable for conserved light-responsive regulator identification.

Several transcription factors with unknown CAM function were identified in the *K. fedtschenkoi* co-expression network. These transcription factors potentially represent novel regulatory mechanisms. As shown in Fig. 6, *WRKY4* was positively co-expressed with *CCA1* and *RVE8*, but negatively co-expressed with *LUX*. Although there is no direct evidence for the involvement of *WRKY4* gene in circadian rhythm, its homolog in tomato is up-regulated at eight hours after dawn, presumptive dusk and four hours after dusk in comparison with presumptive dawn in long day condition [45]. Furthermore, some abscisic acid and light signaling-related genes were identified in our CAM gene-enriched sub-network, e.g., *ZINC FINGER PROTEINS* (*ZFP4* and *ZFP7*), *SIGMA FACTORS* (*SIG1*, *SIG4* and *SIG5*) and B-BOX protein *STH2/BBX21*. In *Arabidopsis*, homologs of *ZFP* are involved in light-responsive pathways, where *ZFP3* can interfere with ABA and light signal in plant development and seed germination [46], while *ZFP1* is expressed in downstream of photomorphogenesis [47]. In prokaryotes, sigma factors are well known for their participation in the control of RNA polymerase activity. The phosphorylation of SIG1 selectively inhibits the expression of gene encoding photosystem I [48]. *SIG1* is strongly induced by red and blue light, but *SIG5* is only induced by blue light under mediating of *CRY1* and *CRY2* [49, 50]. As a key component involved in the COP1-HY5 hub, *STH2/BBX21* is controlled

by COP1 through its E3 ubiquitin ligase activity in darkness and promotes photomorphogenesis by activating *HY5* in the light [51]. Our results provide a powerful resource for novel light-responsive regulator identification.

In conclusion, the comprehensive light-responsive gene expression atlas of *K. fedtschenkoi* provides a novel genomics resource for investigating the molecular mechanism underlying the light regulation in CAM plants. The genome-wide co-expression network lays a solid foundation for discovering novel gene function in CAM plants.

Methods

Plant Material and Experimental Treatments

Kalanchoë fedtschenkoi (ORNL diploid accession M2) plants originally started from meristem cuttings were grown in soil for 4 weeks in a Percival Model AR-75L2 growth chamber on a 12-h light (26°C)/12-h dark (18°C) cycle at a photon flux density of 280 µmol m^{-2} s^{-1}. For acclimation prior to light quality or light intensity treatments, plants were placed for at least 2 d in the growth chamber on a 12-h light (26°C)/ 12-h dark (18°C) cycle at a photon flux density of 440 µmol m^{-2} s^{-1}. Light quality treatments consisted of then growing plants under blue light (blue light, 270 µmol m^{-2} s^{-1}) provided by a dark blue gel filter (#119), red light (red light, 280 µmol m^{-2} s^{-1}) provided by primary red gel filter (#106), far-red light (far-red light, 280 µmol m^{-2} s^{-1}) provided by a medium red Roscolux filter (Barndoor Lighting Outfitters, Inc., North Branford, CT), or constant darkness. For all treatments, except constant darkness, a 12-h light (26°C)/12-h dark (18°C) cycle was used. Light intensity treatments consisted of growing the plants under dark growth (dark grown), low light (low light, 150 µmol m^{-2} s^{-1}) or high light (high light, 1,000 µmol m^{-2} s^{-1}) with a 12-h light (26°C) / 12-h dark (18°C) cycle, respectively. All plants used for light quality and light intensity experiments were grown under the indicated conditions for 48 h prior to any tissue collection. All photon flux density measurements described above were taken at leaf level of the apical meristem as these leaves were closest to the light source.

Tissue Collection and RNA Isolation

Fully-expanded leaves (i.e., leaf pair 4-5 counting from the top of the plants) were collected from three biological replicates of plants grown under each of the light quality and light intensity experimental conditions. Each sample was collected at both dawn (2 h before the starting of lighting period) and dusk (2 h before the dark period) time points, wrapped in aluminum foil, immediately frozen in liquid nitrogen, and stored at -80°C until processing. For RNA isolation, frozen leaf tissue samples were ground to a fine powder under liquid nitrogen with a mortar and pestle. Isolation of total RNA then proceeded by using the QIAGEN RNeasy® Plant Mini Kit (Cat No. 74904, Qiagen Inc., Valencia, CA, USA) with the following modifications: 600 mg of frozen ground tissue from each sample was mixed thoroughly with 2.57 ml of Fruit Mate™ (TaKaRa Bio USA, Inc., Mountain View, CA). The resulting suspension was centrifuged at 14,000 × *g* at 4°C for 5 min. The supernatant was then mixed with 1.8 ml of QIAGEN buffer RLT/2-mercaptoethanol mix. This solution was centrifuged at 14,000 × *g* at 25°C for 1 min. The supernatant was then mixed with 0.5 volumes of 100% ethanol and remaining steps were performed according to kit instructions. On-column DNase digestions were performed for all samples according to RNeasy® kit instructions with the QIAGEN RNase-Free DNase Set (Cat No. 79254). Final RNA elution was performed with 50 µl of RNase free water which was run through the column twice. RNA purity and approximate quantity was assessed with a Thermo Scientific™ NanoDrop 2000c spectrophotometer and precise quantity assessed with Quant-iT™ RiboGreen® fluorescence (Thermo Scientific, Rockford, IL). RNA integrity was evaluated on a 1% (w/v) agarose gel using 300 ng RNA.

cDNA Library construction and RNA-seq

Stranded RNA-seq libraries were generated and quantified using qPCR. Sequencing was performed on an Illumina HiSeq 2500 (150 mer paired end sequencing). Raw fastq file reads were filtered and trimmed using the QC pipeline in the US Department of Energy Joint Genome Institute (JGI).

Reads mapping and data analysis

After filtering out low-quality reads, RNA-seq reads from each library were aligned to the *Kalanchoë fedtschenkoi* reference genome [13] using TopHat2 [52]. FeatureCounts [53] was used to generate raw gene counts and only reads that mapped uniquely to one locus were counted. Gene

expression was estimated as transcripts per million (TPM) [54]. DESeq2 (v1.2.10) [55] was subsequently used to determine which genes were differentially expressed between pairs of conditions. The parameters used to "call a gene" between conditions was determined at a false discovery rate (FDR) adjusted p-value$\leq$0.05.

Kalanchoë light-responsive eFP browser

TPM-normalized values of the RNA-Seq data sets were uploaded into the *Kalanchoë* eFP browser of the Bio-Analytic Resource (BAR). Representative images of *Kalanchoë* leaf under different light condition were created and an XML file was generated to power a view within the *Kalanchoë* eFP browser at http://bar.utoronto.ca/~asher/efp_kalanchoe/cgi-bin/efpWeb.cgi.

K-means clustering and Co-expression analysis

Cluster analysis of gene expression patterns was performed according to K-means method in R software. For co-expression analysis, the log2 normalized TPM values of all the samples were used to construct a weighted gene co-expression network using the R package WGCNA [56]. Gene Ontology (GO) enrichment analysis was applied to predict gene function and calculate the functional category using BiNGO [57]. Heatmap and bubble plots were generated by the R package ggplot2. All tools were run with default parameters.

Availability of supporting data

All raw short reads are available in the NCBI SRA database (SRA accessions: SRP146136, SRP146139, SRP146175, SRP146204, SRP146205, SRP146213, SRP148019 – SRP148030, SRP148037 – SRP148060) (Supplementary Table S1).

Abbreviations

BAR: Bio-Analytic Resource; CAM: Crassulacean acid metabolism; CBS: CCA1 binding sites; DEG: differentially expressed gene; EE: evening elements; FDR: false discovery rate; GO: gene ontology; MDS: multidimensional scaling; PC: principal component; PCC: Pearson correlation coefficient; PPFD: photosynthetic photon flux density; RNA-Seq: RNA-Sequencing; RuBISCO:

ribulose-1,5-bisphosphate carboxylase/oxygenase; TF: transcription factor; TPM: transcripts per million; WGCNA: weighted gene co-expression network analysis; WUE: water-use efficiency.

Competing interests

The authors declare that they have no competing interests.

Funding

This research was supported by the U.S. Department of Energy, Office of Science, Genomic Science Program under Award Number DE-SC0008834. Additional support was provided by the Community Science Program (project 503025) at the Department of Energy Joint Genome Institute and the DOE Center for Bioenergy Innovation at the Oak Ridge National Laboratory.

Author contributions

X.Y., J.Z. and R.H. conceived and designed the research. R.H., T.G., P.Y., and J.C.C. performed the experiments. J.Z., A.S., A.L., M.W., D.L., V.N., J.S. and A.M.B. analyzed the data. J.Z., A.P. and N.J.P. provided eFP browser instance. J.Z. and R.H. drafted the manuscript. X.Y., J.G.C., W.M. and G.A.T. revised the manuscript. All authors read and approved the manuscript.

Acknowledgements

This manuscript has been authored by UT-Battelle, LLC under Contract No. DE-AC05-00OR22725 with the U.S. Department of Energy. The work conducted by the U.S. Department of Energy Joint Genome Institute is supported by the Office of Science of the U.S. Department of Energy under Contract No. DE-AC02-05CH11231. This research used resources of the Compute and Data Environment for Science (CADES) and the Oak Ridge Leadership Computing Facility at the Oak Ridge National Laboratory.

481

References

1. Mølmann JA, Junttila O, Johnsen Ø and Olsen JE. Effects of red, far-red and blue light in maintaining growth in latitudinal populations of Norway spruce (Picea abies). Plant, Cell & Environment. 2006;29 2:166-72.
2. Quail PH. Phytochrome photosensory signalling networks. Nature Reviews Molecular Cell Biology. 2002;3 2:85.
3. OuYang F, Mao J-F, Wang J, Zhang S and Li Y. Transcriptome analysis reveals that red and blue light regulate growth and phytohormone metabolism in Norway spruce [Picea abies (L.) Karst.]. PloS One. 2015;10 8:e0127896.
4. Briggs WR and Olney MA. Photoreceptors in plant photomorphogenesis to date. Five phytochromes, two cryptochromes, one phototropin, and one superchrome. Plant Physiology. 2001;125 1:85-8.
5. Lin C and Shalitin D. Cryptochrome structure and signal transduction. Annual Review of Plant Biology. 2003;54 1:469-96.
6. Briggs W, Beck C, Cashmore A, Christie J, Hughes J, Jarillo J, et al. The phototropin family of photoreceptors. Plant Cell. 2001;13 5:993-7.
7. Nagy F and Schäfer E. Phytochromes control photomorphogenesis by differentially regulated, interacting signaling pathways in higher plants. Annual Review of Plant Biology. 2002;53 1:329-55.
8. Fan X-X, Xu Z-G, Liu X-Y, Tang C-M, Wang L-W and Han X-l. Effects of light intensity on the growth and leaf development of young tomato plants grown under a combination of red and blue light. Scientia Horticulturae. 2013;153:50-5.
9. Rossel JB, Wilson IW and Pogson BJ. Global changes in gene expression in response to high light in *Arabidopsis*. Plant Physiology. 2002;130 3:1109-20.
10. Zavala J and Ravetta D. Allocation of photoassimilates to biomass, resin and carbohydrates in *Grindelia chiloensis* as affected by light intensity. Field Crops Research. 2001;69 2:143-9.
11. Yang X, Cushman JC, Borland AM, Edwards EJ, Wullschleger SD, Tuskan GA, et al. A roadmap for research on crassulacean acid metabolism (CAM) to enhance sustainable food and bioenergy production in a hotter, drier world. New Phytologist. 2015;207 3:491-504.
12. Borland AM, Hartwell J, Weston DJ, Schlauch KA, Tschaplinski TJ, Tuskan GA, et al. Engineering crassulacean acid metabolism to improve water-use efficiency. Trends in Plant Science. 2014;19 5:327-38.
13. Yang X, Hu R, Yin H, Jenkins J, Shu S, Tang H, et al. The *Kalanchoë* genome provides insights into convergent evolution and building blocks of crassulacean acid metabolism. Nature Communications. 2017;8 1:1899.
14. Borland AM, Wullschleger SD, Weston DJ, Hartwell J, Tuskan GA, Yang X, et al. Climate-resilient agroforestry: physiological responses to climate change and engineering of crassulacean acid metabolism (CAM) as a mitigation strategy. Plant, Cell & Environment. 2015;38 9:1833-49.
15. Hartwell J, Dever LV and Boxall SF. Emerging model systems for functional genomics analysis of Crassulacean acid metabolism. Current Opinion in Plant Biology. 2016;31:100-8.

16. Dodd AN, Borland AM, Haslam RP, Griffiths H and Maxwell K. Crassulacean acid metabolism: plastic, fantastic. Journal of experimental botany. 2002;53 369:569-80.
17. Grams TE and Thiel S. High light-induced switch from C 3-photosynthesis to Crassulacean acid metabolism is mediated by UV-A/blue light. Journal of Experimental Botany. 2002;53 373:1475-83.
18. Ceusters J, Borland AM, Godts C, Londers E, Croonenborghs S, Van Goethem D, et al. Crassulacean acid metabolism under severe light limitation: a matter of plasticity in the shadows? Journal of Experimental Botany. 2010;62 1:283-91.
19. Ceusters J, Borland AM, Taybi T, Frans M, Godts C and De Proft MP. Light quality modulates metabolic synchronization over the diel phases of crassulacean acid metabolism. Journal of Experimental Botany. 2014;65 13:3705-14.
20. Kornas A, Fischer-Schliebs E, Lüttge U and Miszalski Z. Adaptation of the obligate CAM plant *Clusia alata* to light stress: metabolic responses. Journal of Plant Physiology. 2009;166 17:1914-22.
21. Miszalski Z, Kornas A, Rozpądek P, Fischer-Schliebs E and Lüttge U. Independent fluctuations of malate and citrate in the CAM species *Clusia hilariana* Schltdl. under low light and high light in relation to photoprotection. Journal of Plant Physiology. 2013;170 5:453-8.
22. Klepikova AV, Kasianov AS, Gerasimov ES, Logacheva MD and Penin AA. A high resolution map of the Arabidopsis thaliana developmental transcriptome based on RNA-seq profiling. Plant Journal. 2016;88 6:1058-70. doi:doi:10.1111/tpj.13312.
23. Benedito VA, Torres-Jerez I, Murray JD, Andriankaja A, Allen S, Kakar K, et al. A gene expression atlas of the model legume *Medicago truncatula*. Plant Journal. 2008;55 3:504-13. doi:doi:10.1111/j.1365-313X.2008.03519.x.
24. Matas AJ, Yeats TH, Buda GJ, Zheng Y, Chatterjee S, Tohge T, et al. Tissue- and cell-type specific transcriptome profiling of expanding tomato fruit provides insights into metabolic and regulatory specialization and cuticle formation. Plant Cell. 2011;23 11:3893-910. doi:10.1105/tpc.111.091173 %J The Plant Cell.
25. Ramírez-González RH, Borrill P, Lang D, Harrington SA, Brinton J, Venturini L, et al. The transcriptional landscape of polyploid wheat. Science. 2018;361 6403:eaar6089. doi:10.1126/science.aar6089 %J Science.
26. Sibout R, Proost S, Hansen BO, Vaid N, Giorgi FM, Ho-Yue-Kuang S, et al. Expression atlas and comparative coexpression network analyses reveal important genes involved in the formation of lignified cell wall in *Brachypodium distachyon*. New Phytologist. 2017;215 3:1009-25. doi:10.1111/nph.14635.
27. Li Q and Kubota C. Effects of supplemental light quality on growth and phytochemicals of baby leaf lettuce. Environmental and Experimental Botany. 2009;67 1:59-64.
28. Fukuda N, Fujita M, Ohta Y, Sase S, Nishimura S and Ezura H. Directional blue light irradiation triggers epidermal cell elongation of abaxial side resulting in inhibition of leaf epinasty in geranium under red light condition. Scientia Horticulturae. 2008;115 2:176-82.
29. Kitazaki K, Fukushima A, Nakabayashi R, Okazaki Y, Kobayashi M, Mori T, et al. Metabolic reprogramming in leaf lettuce grown under different light quality and intensity conditions using narrow-band LEDs. Scientific Reports. 2018;8 1:7914.

30. Li C-X, Xu Z-G, Dong R-Q, Chang S-X, Wang L-Z, Khalil-Ur-Rehman M, et al. An RNA-seq analysis of grape plantlets grown in vitro reveals different responses to blue, green, red LED light, and white fluorescent light. Frontiers in Plant Science. 2017;8:78.

31. Tardu M, Dikbas UM, Baris I, Kavakli IHJF and genomics i. RNA-seq analysis of the transcriptional response to blue and red light in the extremophilic red alga, *Cyanidioschyzon merolae*. Functional & Integrative Genomics. 2016;16 6:657-69.

32. Hao X, Li L, Hu Y, Zhou C, Wang X, Wang L, et al. Transcriptomic analysis of the effects of three different light treatments on the biosynthesis of characteristic compounds in the tea plant by RNA-Seq. Tree Genetics & Genomes. 2016;12 6:118.

33. Sellaro R, Hoecker U, Yanovsky M, Chory J and Casal JJ. Synergism of red and blue light in the control of Arabidopsis gene expression and development. Current Biology. 2009;19 14:1216-20.

34. Pazhamala LT, Purohit S, Saxena RK, Garg V, Krishnamurthy L, Verdier J, et al. Gene expression atlas of pigeonpea and its application to gain insights into genes associated with pollen fertility implicated in seed formation. Journal of Experimental Botany. 2017;68 8:2037-54.

35. Kudapa H, Garg V, Chitikineni A and Varshney RK. The RNA-Seq-based high resolution gene expression atlas of chickpea (*Cicer arietinum* L.) reveals dynamic spatio-temporal changes associated with growth and development. Plant, Cell & Environment. 2018;41 9:2209-25. doi:doi:10.1111/pce.13210.

36. Kurepin LV, Emery RN, Pharis RP and Reid DM. The interaction of light quality and irradiance with gibberellins, cytokinins and auxin in regulating growth of *Helianthus annuus* hypocotyls. Plant, Cell & Environment. 2007;30 2:147-55.

37. Zhang Z, Ji R, Li H, Zhao T, Liu J, Lin C, et al. CONSTANS-LIKE 7 (COL7) is involved in phytochrome B (phyB)-mediated light-quality regulation of auxin homeostasis. Molecular Plant. 2014;7 9:1429-40.

38. Gubler F, Hughes T, Waterhouse P and Jacobsen J. Regulation of dormancy in barley by blue light and after-ripening: effects on abscisic acid and gibberellin metabolism. Plant Physiology. 2008;147 2:886-96.

39. Nusinow DA, Helfer A, Hamilton EE, King JJ, Imaizumi T, Schultz TF, et al. The ELF4-ELF3-LUX complex links the circadian clock to diurnal control of hypocotyl growth. Nature. 2011;475 7356:398-402. doi:10.1038/nature10182.

40. Lu SX, Knowles SM, Andronis C, Ong MS and Tobin EM. CIRCADIAN CLOCK ASSOCIATED1 and LATE ELONGATED HYPOCOTYL function synergistically in the circadian clock of *Arabidopsis*. Plant Physiology. 2009;150 2:834-43. doi:10.1104/pp.108.133272.

41. Kikis EA, Khanna R and Quail PH. ELF4 is a phytochrome-regulated component of a negative-feedback loop involving the central oscillator components CCA1 and LHY. Plant Journal. 2005;44 2:300-13. doi:10.1111/j.1365-313X.2005.02531.x.

42. Hazen SP, Schultz TF, Pruneda-Paz JL, Borevitz JO, Ecker JR and Kay SA. LUX ARRHYTHMO encodes a Myb domain protein essential for circadian rhythms. Proc Natl Acad Sci U S A. 2005;102 29:10387-92. doi:10.1073/pnas.0503029102.

43. Lee HG, Mas P and Seo PJ. MYB96 shapes the circadian gating of ABA signaling in *Arabidopsis*. Scientific Reports. 2016;6:17754. doi:10.1038/srep17754.

44. Muchapirei CI, Valentine S-L and Roden LC. Plant circadian networks and responses to the environment. Functional Plant Biology. 2018;45 4:393-9.

45. Facella P, Lopez L, Carbone F, Galbraith DW, Giuliano G and Perrotta G. Diurnal and Circadian Rhythms in the Tomato Transcriptome and Their Modulation by Cryptochrome Photoreceptors. PLOS ONE. 2008;3 7:e2798. doi:10.1371/journal.pone.0002798.
46. Joseph MP, Papdi C, Kozma-Bognar L, Nagy I, Lopez-Carbonell M, Rigo G, et al. The *Arabidopsis* ZINC FINGER PROTEIN3 interferes with abscisic acid and light signaling in seed germination and plant development. Plant Physiology. 2014;165 3:1203-20. doi:10.1104/pp.113.234294.
47. Chrispeels HE, Oettinger H, Janvier N and Tague BW. *AtZFP1*, encoding *Arabidopsis thaliana* C2H2 zinc-finger protein 1, is expressed downstream of photomorphogenic activation. Plant Molecular Biology. 2000;42 2:279-90.
48. Shimizu M, Kato H, Ogawa T, Kurachi A, Nakagawa Y and Kobayashi H. Sigma factor phosphorylation in the photosynthetic control of photosystem stoichiometry. Proc Natl Acad Sci U S A. 2010;107 23:10760-4. doi:10.1073/pnas.0911692107.
49. Tsunoyama Y, Morikawa K, Shiina T and Toyoshima Y. Blue light specific and differential expression of a plastid σ factor, Sig5 in *Arabidopsis thaliana*. FEBS Letters. 2002;516 1:225-8. doi:10.1016/S0014-5793(02)02538-3.
50. Onda Y, Yagi Y, Saito Y, Takenaka N and Toyoshima Y. Light induction of *Arabidopsis SIG1* and *SIG5* transcripts in mature leaves: differential roles of cryptochrome 1 and cryptochrome 2 and dual function of SIG5 in the recognition of plastid promoters. Plant Journal. 2008;55 6:968-78. doi:10.1111/j.1365-313X.2008.03567.x.
51. Xu D, Jiang Y, Li J, Lin F, Holm M and Deng XW. BBX21, an *Arabidopsis* B-box protein, directly activates HY5 and is targeted by COP1 for 26S proteasome-mediated degradation. Proceedings of the National Academy of Sciences of the United States of America. 2016;113 27:7655-60. doi:10.1073/pnas.1607687113.
52. Kim D, Pertea G, Trapnell C, Pimentel H, Kelley R and Salzberg SL. TopHat2: accurate alignment of transcriptomes in the presence of insertions, deletions and gene fusions. Genome Biology. 2013;14 4:R36.
53. Liao Y, Smyth GK and Shi W. featureCounts: an efficient general purpose program for assigning sequence reads to genomic features. Bioinformatics. 2013;30 7:923-30.
54. Li B and Dewey CN. RSEM: accurate transcript quantification from RNA-Seq data with or without a reference genome. BMC Bioinformatics. 2011;12 1:323.
55. Love MI, Huber W and Anders S. Moderated estimation of fold change and dispersion for RNA-seq data with DESeq2. Genome Biology. 2014;15 12:550.
56. Langfelder P and Horvath S. WGCNA: an R package for weighted correlation network analysis. BMC Bioinformatics. 2008;9 1:559.
57. Maere S, Heymans K and Kuiper M. BiNGO: a Cytoscape plugin to assess overrepresentation of gene ontology categories in biological networks. Bioinformatics. 2005;21 16:3448-9.

Figure Legends

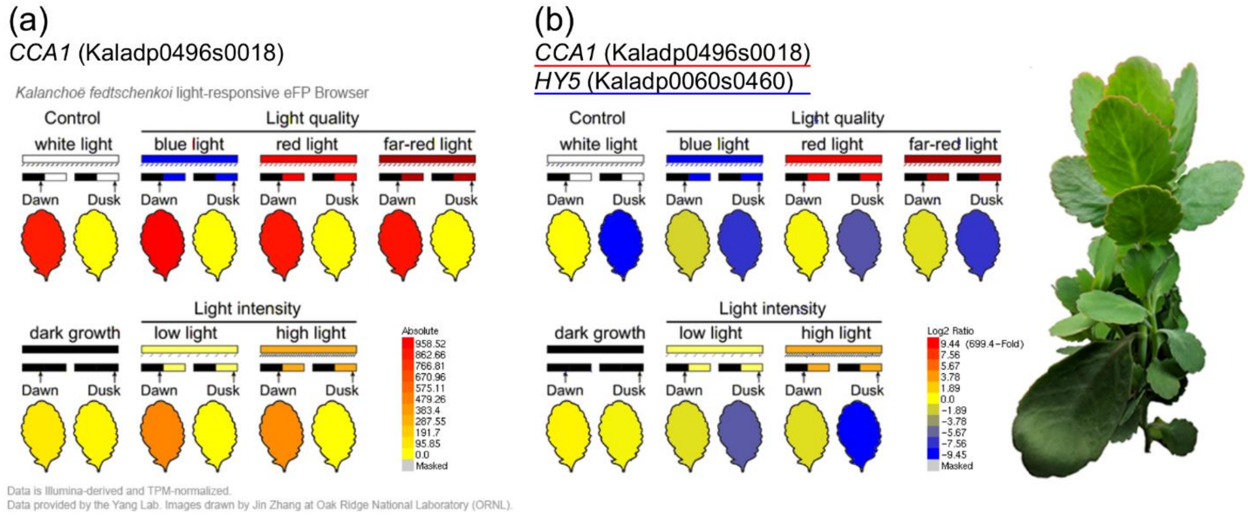

Fig. 1. ***Kalanchoë*** **light-responsive eFP browser.**

(**a**) View of the eFP browser including the RNA-Seq data set described in this study. Expression values in the samples are indicated by a color gradient, where yellow indicates low expression and red indicates high expression. The legend describing the color gradient and expression values is shown in the bottom right corner. *CCA1* gene Kaladp0496s0018 is used as an example.

(**b**) *Kalanchoë* genes *CCA1* (Kaladp0496s0018) and *HY5* (Kaladp0060s0460) displayed in a comparative view of the expression level extracted from the eFP browser. The legend describing the color gradient and log2 ratio is shown in the bottom right corner.

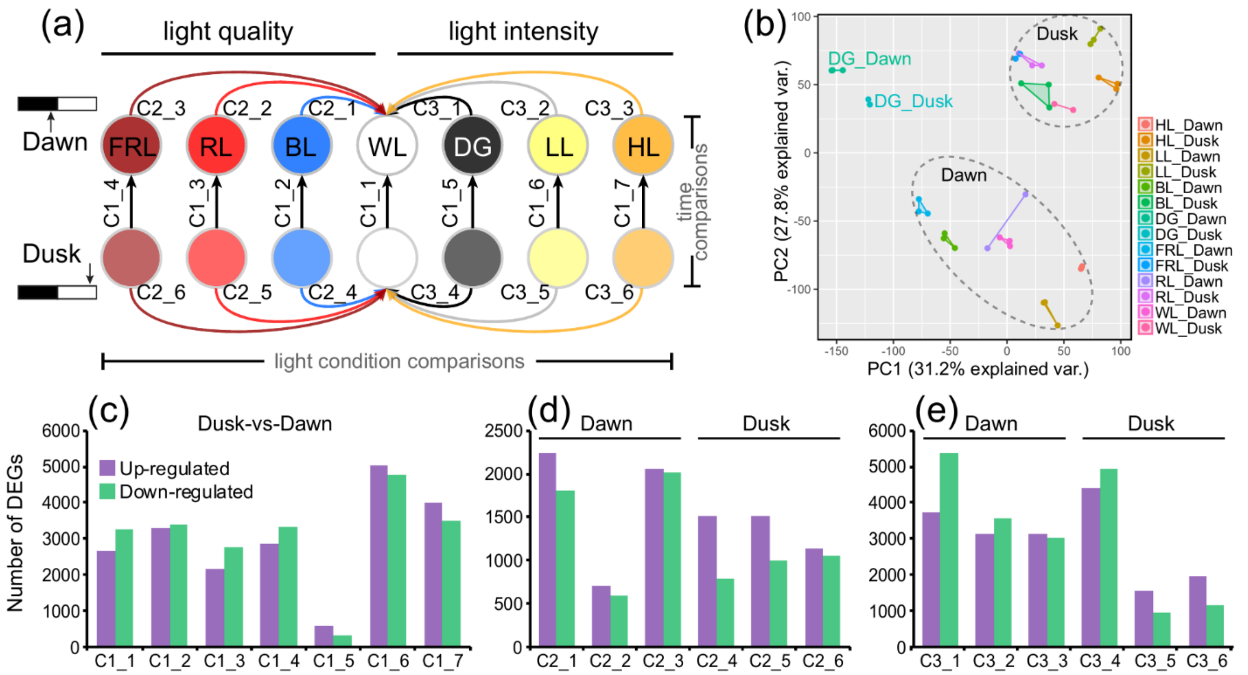

Fig. 2. Transcriptomic comparison of *Kalanchoë fedtschenkoi* under various light quality and light intensity conditions.

(a) Schematic of sample collection and comparisons. The leaf samples were collected at dawn (2-hour before light period) and dusk (2-hour before dark period) under control condition (white light, white light) and various light quality conditions (blue light, blue light; red light, red light; and far-red light, far-red light) and light intensity conditions (dark growth, dark grown; low light intensity, low light; and high light intensity, high light). For differentially expressed genes (DEGs) identification, the comparisons were classified into time comparisons (C1_1 to C1_7 between dusk and dawn) and light condition comparisons (C2_1 to C2_6 for light quality comparisons and C3_1 to C3_6 for light intensity comparisons).

(b) Principal component analysis (PCA) of the 14 groups of transcriptome data. Two dashed ellipses indicate samples collected at dawn and dusk, respectively.

(c-e) Statistic of DEGs between dawn and dusk in time comparisons **(c)** and among various light quality **(d)** or light intensity **(e)** in light condition comparisons.

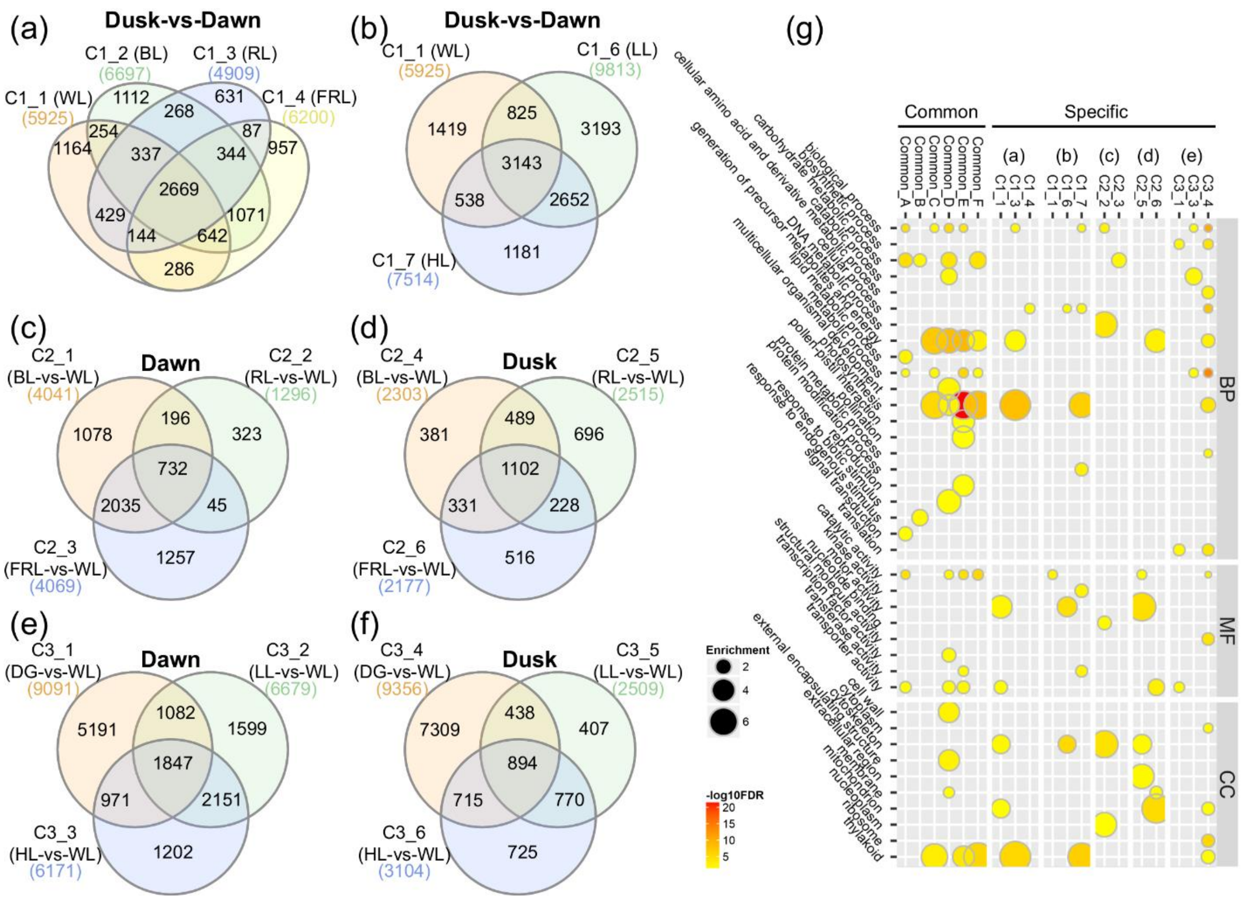

Fig. 3. Comparison of DEGs in time comparisons and light condition comparisons.

(a, b) Venn diagrams represent DEGs of dusk-vs-dawn overlapped under different light quality **(a)** and light intensity **(b)**. **(c, d)** Venn diagrams represent DEGs under various light quality (blue light/red light/far-red light-vs-white light) at dawn **(c)** and dusk **(d)**. **(e, f)** Venn diagrams represent DEGs under different light intensity (dark grown/low light/high light-vs-white light) at dawn **(e)** and dusk **(f)**. **(g)** Gene ontology (GO) enrichment of DEGs shared by different comparisons in A-F Venn diagrams (Common) or specific DEGs in each Venn diagram. BP, biological process; MF, molecular function; and CC, cellular component. GOslim terms were shown in here, full list of enriched GO terms was listed in Supplementary Table S3 and Supplementary Fig. S2-S4.

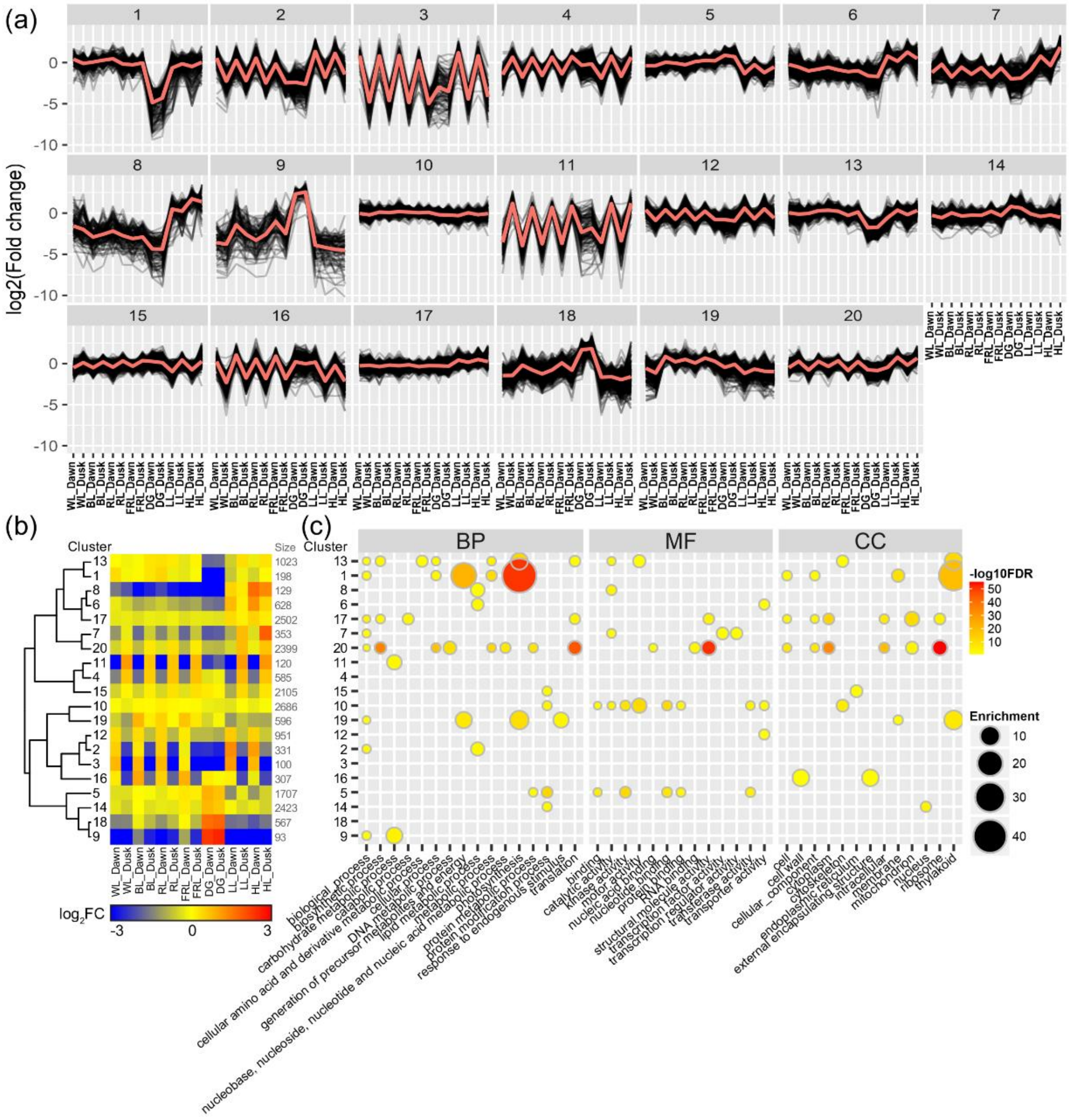

Fig. 4. K-means clustering of DEGs in *K. fedtschenkoi* under light quality and light intensity conditions.

(a) K-means clustering of DEGs across 14 samples under various light quality and light intensity conditions (K=20 clusters). **(b)** Correlation dendrogram and expression heatmap of the 20 clusters. **(c)** GO enrichment of DEGs in 20 clusters. BP, biological process; MF, molecular function; and CC, cellular component. GOslim terms were shown in here, full list of enriched GO terms was listed in Supplementary Table S4 and Supplementary Fig. S5-S7. Node color represents -log10 transformed FDR corrected *P* value. Node size represents enrichment factor.

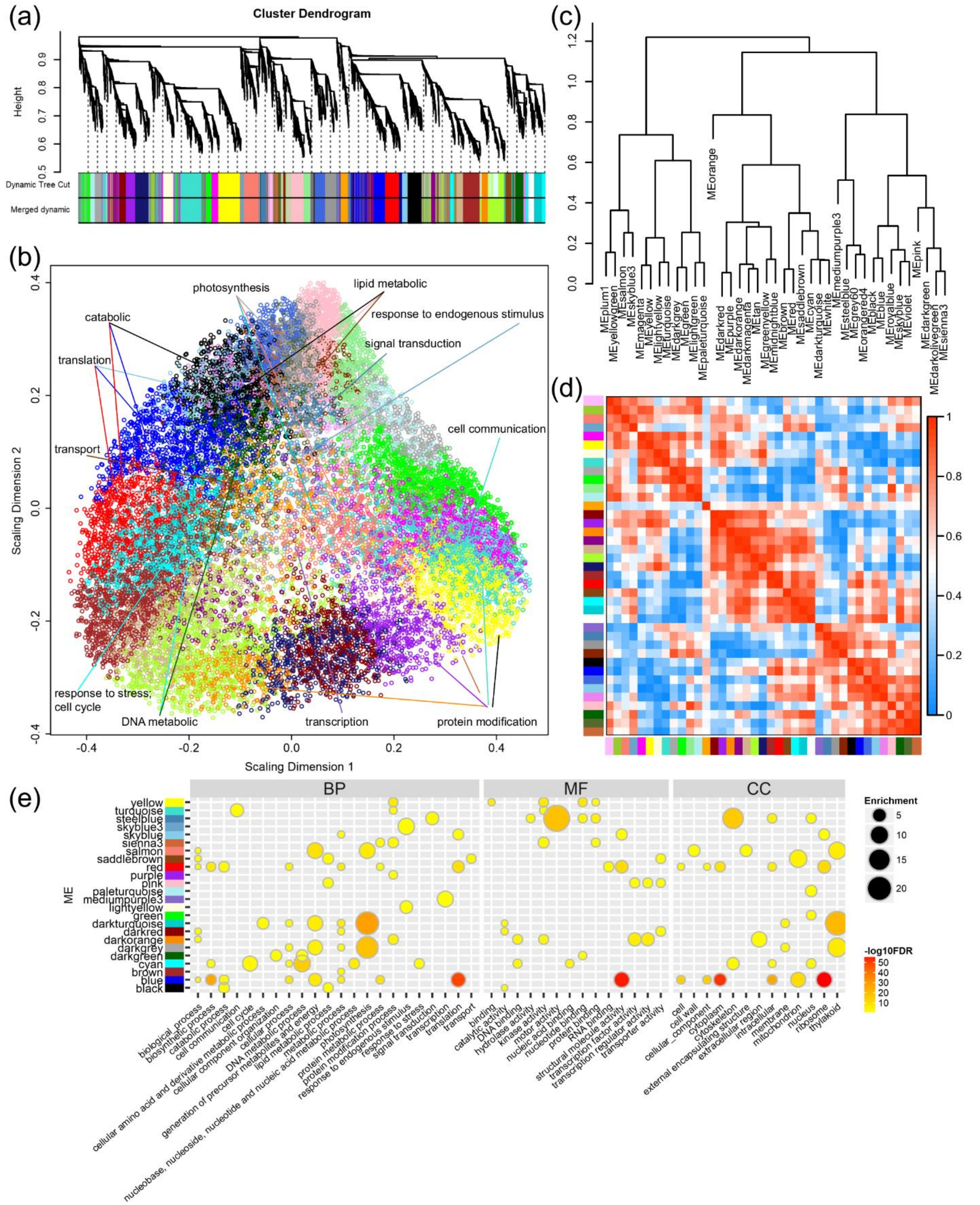

Fig. 5. Weighted Gene Co-expression Network Analysis (WGCNA) of DEGs in *Kalanchoë fedtschenkoi* under various light quality and light intensity conditions.

(a) Cluster dendrogram of DEGs in *Kalanchoë fedtschenkoi* under various light quality and light intensity conditions. Different colors in merged dynamic column represent different modules (MEs).

(b) Multidimensional scaling (MDS) plot of DEGs in different modules.

(c) Cluster dendrogram of different MEs.

(d) Correlation analysis of different MEs.

(e) GO enrichment analysis of DEGs in different MEs.

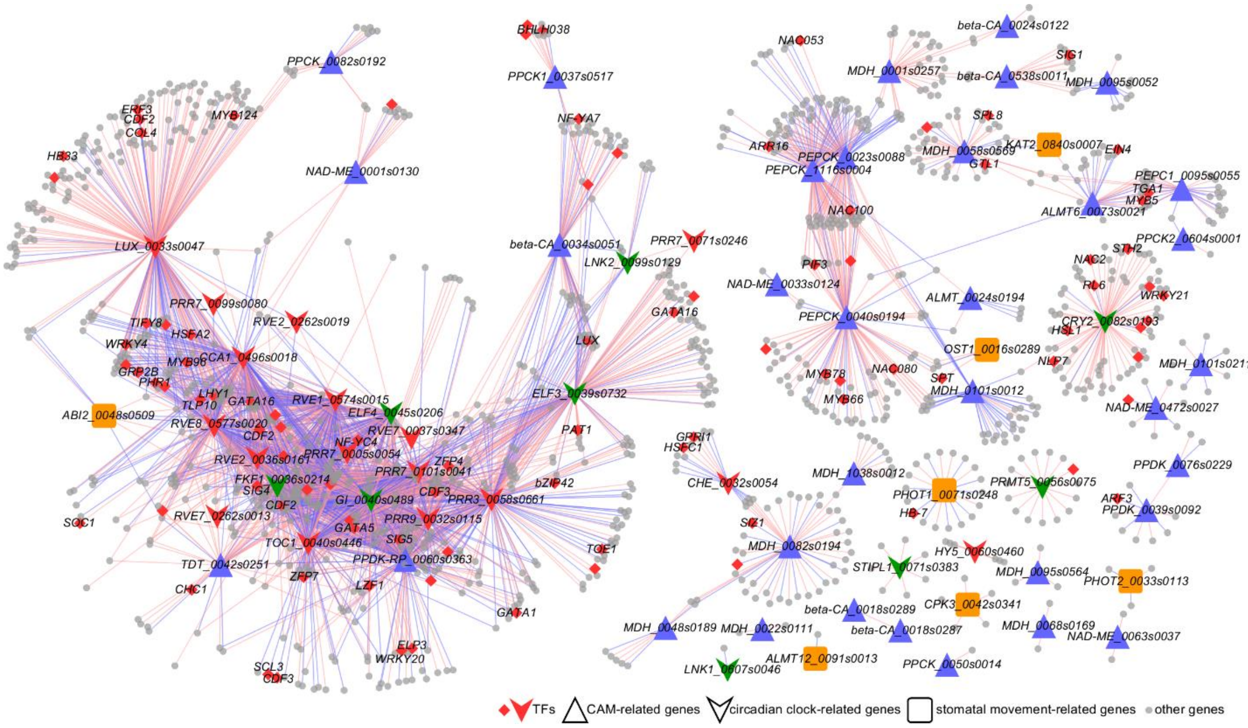

Fig. 6. Sub-network of CAM, circadian clock and stomatal movement.

Red nodes represent transcription factors (TFs). Triangle, vee and roundrec shapes of nodes represent CAM-, circadian clock- and stomatal movement-related genes, respectively. Red and blue edges represent positive correlation (PCC>0.95 and $p \leq 0.01$) and negative correlation (PCC<-0.95 and $p \leq 0.01$), respectively.

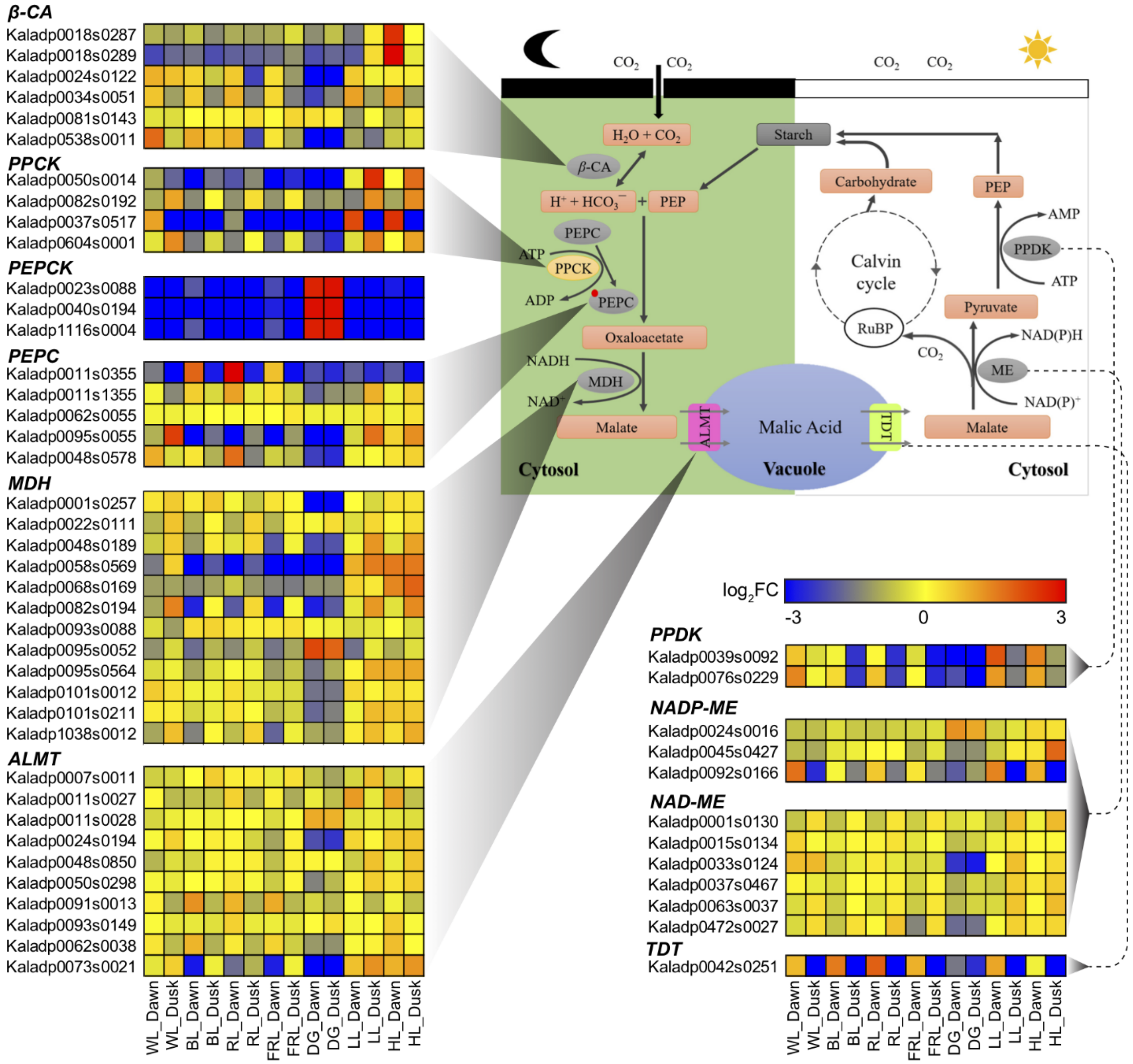

Fig. 7. Expression profiles of genes involved in CAM pathway.

The CAM pathway was modified from Yang et al. (2017). Red and green color indicate up- and down-regulation of log2 transformed fold changes across the 14 samples.

Supporting information

Additional Supporting Information may be found online in the Supporting Information section at the end of the article:

Fig. S1. Expression distribution and correlation of 42 RNA-seq libraries.

(a) Distribution of gene expression levels of all the samples in this study. The gene expression levels were transformed by log_{10}(TPM+1). (b) Pearson correlation between samples.

Fig. S2. GO enrichment analysis of unique and common DEGs under light quality and light intensity treatments at the category of biological process (BP).

Fig. S3. GO enrichment analysis of unique and common DEGs under light quality and light intensity treatments at the category of molecular function (MF).

Fig. S4. GO enrichment analysis of unique and common DEGs under light quality and light intensity treatments at the category of cellular component (CC).

Fig. S5. GO enrichments analysis of DEGs in the 20 clusters at the category of BP.

Fig. S6. GO enrichments analysis of DEGs in the 20 clusters at the category MF.

Fig. S7. GO enrichments analysis of DEGs in the 20 clusters at the category of CC.

Fig. S8. Transcription factor (TF) number and enrichment in the 20 clusters.

Table S1. Experimental conditions and samples used in this study.

Table S2. Differentially expressed genes (DEGs) in pairwise comparisons.

Table S3. GO enrichment of common genes and specific genes in Venn diagrams.

Table S4. GO enrichment of genes in the 20 K-means clusters.

Table S5. Transcription factors in the 20 K-means clusters.

Table S6. Genes in the 39 modules from WGCNA analysis.

Table S7. GO enrichment of genes in the 39 modules.

Table S8. Gene list and functional annotation of the sub-network.

Table S9. Expression patterns of CAM-, circadian- and stomatal movement-related genes.

Supporting information

Click here to access/download
Supplementary Material
Supporting_Information_V2.docx

Table S3

Click here to access/download

Supplementary Material

Table S3.xlsx

Table S4

Click here to access/download
Supplementary Material
Table S4.xlsx

Table S5

Click here to access/download
Supplementary Material
Table S5.xlsx

Table S7

Click here to access/download
Supplementary Material
Table S7.xlsx

Table S8

Click here to access/download
Supplementary Material
Table S8.xlsx

Table S9

Click here to access/download
Supplementary Material
Table S9.xlsx
