# Supplementary material for: Light-responsive expression atlas reveals the effects of light quality and intensity in Kalanchoë fedtschenkoi, a plant with crassulacean acid metabolism
Source: Gigascience. 2020 Mar 5;9(3):giaa018. doi: 10.1093/gigascience/giaa018 (PMC7058158; doi:10.1093/gigascience/giaa018)
Supplement: giaa018_GIGA-D-19-00095_Revision_1 [file giaa018_giga-d-19-00095_revision_1.pdf]

GigaScience

Light-responsive expression atlas reveals the effects of light quality and intensity in Kalanchoë fedtschenkoi, a plant with crassulacean acid metabolism

--Manuscript Draft--

**Manuscript Number:**	GIGA-D-19-00095R1
**Full Title:**	Light-responsive expression atlas reveals the effects of light quality and intensity in Kalanchoë fedtschenkoi, a plant with crassulacean acid metabolism
**Article Type:**	Research
**Funding Information:**	Genomic Science Program (DE-SC0008834) — Dr Xiaohan Yang<br>Community Science Program (503025) — Dr Xiaohan Yang
**Abstract:**	Background: Crassulacean acid metabolism (CAM), a specialized mode of photosynthesis, enables plant adaptation to water-limited environments and improves photosynthetic efficiency via an inorganic carbon-concentrating mechanism. Kalanchoë fedtschenkoi is an obligate CAM model featuring a relatively small genome and easy stable transformation. However, the molecular responses to light quality and intensity in CAM plants remain understudied.<br><br>Results: Here we present a genome-wide expression atlas of K. fedtschenkoi plants grown under 12h/12h photoperiod with different light quality (blue, red, far-red, white light) and intensity (0, 150, 440, and 1000 μmol m–2 s–1) based on RNA-Seq performed for mature leaf samples collected at dawn (2-h before the starting of lighting period) and dusk (2-h before the dark period). An eFP web browser was created for easy access of the gene expression data. Based on the expression atlas, we constructed a light-responsive co-expression network to reveal the potential regulatory relationships in K. fedtschenkoi. Measurements of leaf titratable acidity, soluble sugar and starch turnover provided metabolic indicators of the magnitude of CAM under the different light treatments and were used to provide biological context for the expression dataset. Furthermore, CAM-related subnetworks were highlighted to showcase genes relevant to CAM pathway, circadian clock and stomatal movement. In comparison with white light, monochrome blue/red/far-red light treatments repressed the expression of several CAM-related genes at dusk, along with a major reduction in acid accumulation. Increasing light intensity from an intermediate level (440 mol m-2 s-1) of white light to a high light treatment (1000 μmol m–2 s–1) increased expression of several genes involved in dark CO2 fixation and malate transport at dawn, along with an increase in organic acid accumulation.<br><br>Conclusions: This study provides a highly useful genomics resource for investigating the molecular mechanism underlying the light regulation of physiology and metabolism in CAM plants. Our results support the hypothesis that both light intensity and light quality can modulate the CAM pathway through regulation of CAM-related genes in K. fedtschenkoi.
**Corresponding Author:**	Jin Zhang<br>Oak Ridge National Laboratory<br>oak ridge, TN UNITED STATES
**Corresponding Author Secondary Information:**	
**Corresponding Author's Institution:**	Oak Ridge National Laboratory
**Corresponding Author's Secondary Institution:**	
**First Author:**	Jin Zhang
**First Author Secondary Information:**	
**Order of Authors:**	Jin Zhang

Powered by Editorial Manager® and ProduXion Manager® from Aries Systems Corporation

	Rongbin Hu
	Avinash Sreedasyam
	Travis Garcia
	Anna Lipzen
	Mei Wang
	Pradeep Yerramsetty
	Degao Liu
	Vivian Ng
	Jeremy Schmutz
	John Cushman
	Anne Borland
	Asher Pasha
	Nicholas Provart
	Jin-Gui Chen
	Wellington Muchero
	Gerald Tuskan
	Xiaohan Yang
**Order of Authors Secondary Information:**	
**Response to Reviewers:**	The Editor<br>Dr. Hans Zauner<br>GigaScience<br><br>November 8th, 2019<br><br>Dear Editor,<br>Thank you for your evaluation of our manuscript GIGA-D-19-00095 (Expression atlas and co-expression network reveal effects of light quality and intensity in Kalanchoë fedtschenkoi, a plant with crassulacean acid metabolism) and the invitation to submit a revised manuscript. Enclosed please find the revised manuscript in which all the comments raised by the reviewers and you have been addressed. A detailed, point-by-point rebuttal to these comments in included below. For your convenience, we highlighted all changes in the manuscript text file. We hope this modified version is now suitable for publication in GigaScience.<br><br>Sincerely,<br>Xiaohan Yang<br>Senior Staff Scientist<br>Biosciences Division<br>Oak Ridge National Laboratory, USA<br>Phone: (865)-241-6895<br>E-mail: yangx@ornl.gov<br>https://www.ornl.gov/staff-profile/xiaohan-yang<br><br><br>Dear Dr. Zhang,<br><br>Your manuscript "Expression atlas and co-expression network reveal effects of light quality and intensity in Kalanchoë fedtschenkoi, a plant with crassulacean acid metabolism" (GIGA-D-19-00095) has been assessed by our reviewers. Although it is of interest, we are unable to consider it for publication in its current form. The reviewers have raised a number of points which we believe would improve the manuscript and may allow a revised version to be published in GigaScience.

Powered by Editorial Manager® and ProduXion Manager® from Aries Systems Corporation

Their reports are below.
Generally speaking, in light of the reviewers's reports, I get the impression that your article falls in between two article types we publish in GigaScience:
We publish "Data Notes", which present useful resources and describe in detail how the data have been generated, including test / validation data and discussing use cases. "Data Notes" don't need to answer biological questions.
In contrast, our "Research Articles" should answer biological questions, in addition to describing the datasets.
Both reviewers agree that your manuscript presents a useful resource, but they got the impression that it is lacking a bit in terms of the biological insights presented. I suggest there are two ways forward during the revision: You may choose to re-submit as a "Research Article". To convince the reviewers I feel you will need to expand on the biological insights gained from your work, also including some further analyses.
Alternatively, you may decide to reformat the submission as a "Data Note", and focus on presenting the data as a resource and showing some use cases.
[Response]: Thanks for your suggestion. We have added the additional experiments according to reviewers' comments, performed further data analyses and added a substantial amount of additional description and discussion into the biological insights. Thus, we have re-submitted the manuscript as a "Research Article".

In any case, please address the questions and comments from both reviewers and fill in missing details regarding the methodology (e.g. number of replicates, method for library construction etc - see below).
Please also carefully address reviewer 2's comments regarding the clustering procedures. In particular, I agree with the reviewer that "outliers" should not be arbitrarily removed, please include all replicates in the analyses.
[Response]: Yes, we have added all the replicates in the revised manuscript according to your and reviewer #2's suggestions.

If you are able to fully address these points, we would encourage you to submit a revised manuscript to GigaScience. Once you have made the necessary corrections, please submit online at: https://www.editorialmanager.com/giga/

If you have forgotten your username or password please use the "Send Login Details" link to get your login information. For security reasons, your password will be reset.

Please include a point-by-point within the 'Response to Reviewers' box in the submission system. Please ensure you describe additional experiments that were carried out and include a detailed rebuttal of any criticisms or requested revisions that you disagreed with. Please also ensure that your revised manuscript conforms to the journal style, which can be found in the Instructions for Authors on the journal homepage.

The due date for submitting the revised version of your article is 01 Aug 2019.

I look forward to receiving your revised manuscript soon.

Best wishes,

Hans Zauner
GigaScience
www.gigasciencejournal.com

Reviewer reports:
Reviewer #1: Summary of the key results
==================
The manuscript titled "Expression atlas and co-expression network reveal effects of light quality and intensity in Kalanchoë fedtschenkoi, a plant with crassulacean acid metabolism" describe c es the transcriptomic characterization of the K. fedtschenkoi leaves under different light spectrums (blue, red, infrared and white), intensities (0, 150, 250 and 1000 µmol m-2 s-1) and time points associated to the circadian clock (2h after dusk and after dawn). The authors analyze the differential expression between different light quality conditions (light spectrums and intensity) and time points. Additionally the authors performed a clustering and co-expression network analysis

associated with gene ontology terms. Finally to increase the usability of the data for the scientific community, the authors implemented a gene expression graphical interface using the eFP browser at the BAR.

Overall evaluation
===========
This manuscript presents an interesting and useful resource for the plant scientific community, specially the one that it is working in photosynthesis and CAM metabolism. Nevertheless, I found that the manuscript is mostly descriptive without a clear hypothesis and a discussion of the results in agreement with that hypothesis. Probably there is more than enough data to discuss specific aspects of the experiment (e.g. how the light spectrum activate some pathways but not others, why the transcriptomic response is higher with low and high light...). Overall, I think that the authors develop an useful resource but at the same time, I think that the presentation of the results and the discussion should be improved with a stronger biological support.
[Response]: Thanks for your critical review and constructive suggestions. We have revised the manuscript according to your suggestions. Please see the following point-by-point response.

Major concerns
==========
*Lack of a clear hypothesis behind the experiment. The manuscript is mostly descriptive. I see the article as an useful resource to mine information as the author mentioned at the end of the "Background" section. Nevertheless, in my opinion, this manuscript does not have a clear hypothesis behind the experiment or at least it is not clear from the narrative of the results and the discussion.
[Response]:
We added this following hypothesis to the Introduction section (Lines 116 in revised manuscript):
"We hypothesize that both light intensity and light quality can influence the expression of CAM-related metabolic and signaling genes in the obligate CAM species K. fedtschenkoi."
Also, we added the following text to the end of the Discussion section (Lines 491 in revised manuscript):
"Furthermore, the results from our comparative analyses of gene expression and acid accumulation between different light treatments support our hypothesis that both light intensity and light quality can affect the expression of CAM-related genes in K. fedtschenkoi".

*Poor result discussion. The discussion does not contain any element related with the light quality being in more or the cases a comparison which what is known in Arabidopsis rather than a discussion that drives to new hypothesis about the regulatory cascades of the CAM.
[Response]: We added the following text to the Discussion section: (lines 404 – 414 in revised manuscript):
"We found that high level (1,000 mol m-2 s-1) of white light increased expression of three dark CO_2 fixation genes (i.e., β-CA, PPCK, PEPC) and one malate transporter gene (ALMT) at dawn (i.e., 2 h before the beginning of light period) in comparison with intermediate level (440 mol m-2 s-1) of white light (Fig. 7a). This increased transcript abundance of genes involved in dark CO_2 fixation and malate import into the vacuoles was consistent with the higher acid accumulation (i.e., dawn-dusk ΔH^+) under high light relative to intermediate light intensity (Fig. 6b). On the other hand, we found that high level of white light repressed expression of several CAM pathway genes (e.g., PEPC, MDH, PPDK, PPDK-RP) at dusk (i.e., 2 h before the beginning of dark period) in comparison with intermediate level of white light (Fig. 7a). These results suggest that the high-light treatment was still within the normal physiological range of the plant and not yet necessarily saturating the photosynthetic machinery."

Minor concerns
=========
*Language polishing: Although the manuscript is easy to read and there is not major mistakes that I can detect, probably a polishing in the writing will help to do the manuscript more fluid. For example, there are long sentences that written in a different

Powered by Editorial Manager® and ProduXion Manager® from Aries Systems Corporation

way will improve the readability (e.g. Abstract lines 37-40. Background lines 59-62…).
[Response]: We have revised those long sentences and the current draft of the manuscript has gone through several cycles of editing.

*No summary of number of sequenced and mapped reads per replicate. The authors should provide this information in the supplementary data to have a better evaluation of the process (e.g. if only half of the reads map to the genome is an indication of a problem with the samples).
[Response]: We have added the summary of number of sequenced and mapped reads per replicate in Supplementary Table S1. The mapping rate is ranged from 95.02% to 98.21%.

*Section "Differentially expressed genes (DEGs) regulated by light quality and light intensity": This result section is already summarized in the figure 2 and 3. It is interesting to know if the transcriptomic response is higher depending of the light spectrum, quality and/or time point, but I think that the continuous description of what it can be observed in the figures 2 and 3 blurs the result.
[Response]: Thanks for your suggestion, we removed some excessive descriptions in this section to make the results clearer.

*Section "Predicted function of DEGs": I think that it could be convenient to complement the GSEA with a metabolic pathway analysis using tools such as Mapman (https://mapman.gabipd.org/home) to have a better idea of the members involve in the response to those stimuli rather than a general view. It makes sense that "photosynthesis" is an enriched term comparing dawn and dusk, but probably it could be more interesting to know specifically which members of the photosynthetic apparatus are involved.
[Response]: Yes, we re-analyzed the DEGs using MapMan and added the results in Supplementary Figure S3, S4 and new Figure 3. To explore the role of photosynthetic pathway genes in different light responses, we added the expression patterns of photosynthetic genes in new Figure 3. In addition, we analyzed the correlation between selected genes (related to photosynthetic light reactions, Calvin cycle and photorespiration, CAM pathway, circadian rhythm and stomatal movement) and the physiological parameters (titratable acidity at pH 7.0, titratable acidity at pH 8.4, soluble sugar content and starch content) in new Figure 6 panel (e).

*Section "Clusters of DEGs": As in previous sections, the authors describe the composition of the clusters, but not the meaning of those in relation with the biological experiment. For example, "genes from clusters 3 and 11 show an strong response to the circadian clock" could be more informative than "clusters 3 and 11 are composed by genes with DE between dawn and dusk".
[Response]: According to Reviewer #2's suggestion, we have removed the section of K-means clustering analysis.

*Lack of details in the Material and Methods: How many plants per biological replicate did the authors used? How the RNA-Seq libraries were prepared? Did the authors perform a size selection of the fragments? Where the samples were sequenced? Which version was used for TopHat2 and FeatureCounts? (other software versions are also missing).
[Response]: We used one plant per biological replicate and three biological replicates per treatment condition. A total of 42 RNA-Seq libraries were prepared. RNAs were fragmented into ~300bp sizes and <200bp sizes of libraries (RNA+adapter) were removed by SPRI beads. The RNA samples were sequenced in US Department of Energy Joint Genome Institute (Walnut Creek, CA). We used GSNAP (v2018-07-04), (not TopHat2), for mapping and FeatureCounts (in Subread v1.6.1) for reads counting. We have added the technical details in the Material and Methods. (line 539-554).

Discretionary revisions
==============
*Background, line 56: Parenthesis is opened but not close.
[Response]: We have revised the sentence: "There are three types of photoreceptors (i.e., cryptochromes, phototropins and phytochromes) that perform important roles in plant light response [2, 4]."

Powered by Editorial Manager® and ProduXion Manager® from Aries Systems Corporation

*Background, line 92: "However, gene regulation at the transcriptional level in CAM plants in response to various spectral light qualities and intensity has not been reported yet". I am not sure that "reported" is the most adequate verb here. I will suggest "analyzed", "studied" or "investigated". "Report" is more concrete and linked to a result or an observation.
[Response]: We have revised the sentence: “However, gene regulation at the transcriptional level in CAM plants in response to various light qualities and intensities has not been investigated yet.”

*Data description, line 116: "we obtained ~138 Gb of high-quality data". High-quality data in this context is an unprecise term. Please, be more precise (e.g. qscore > 30…).
[Response]: We have added the description in line 137-139: “In total, we obtained 981 million read pairs (2×150 bp) with 287.78 Gb high quality data (Qscore≥25) from the 42 libraries, with an average size of ~23.4 million read pairs per library (Supplementary Table S1).”

*Analysis, line 176: Probably the authors forget to replace the note "cite a figure or table here" by a real figure.
[Response]: The relevant excessive description was removed.

*Analysis, line 217: Why 20 clusters?
[Response]: We have removed the cluster analysis according to your and reviewer #2’s suggestion.

*Discussion, line 335: "carbohydrate metabolic" should be "carbohydrate metabolism".
[Response]: Yes, revised.

*Figure 2: Abbreviations for each conditions should be described in the legend (e.g. LL=Low Light…).
[Response]: Yes, we have added the explanation of the abbreviations in the figure legend.

Reviewer #2: The Authors present their comparative transcriptomics work examining the effect of different light regimes on the new model CAM plant, Kalanchoë fedtschenkoi. The authors perform some large scale typical analyses including differential gene expression and clustering, which indicate the data is of workable quality. Finally the authors touch briefly on a few co-expressed known and novel regulatory factors that may-be of interest in understanding how light signals are integrated into regulating CAM photosynthesis.

Major comments:
1-
The dataset that the authors have generated appears to be of good quality and I anticipate that it will be very useful for the CAM research community.
I also appreciate that both the raw data and the included eFP browser are already available. That said, if the authors are still able to provide some contextual data; I think this might make it much easier to interpret the transcriptomics data.
[Response]: We added some contextual data to the caption of Fig. 1.
“The leaf samples were collected at dawn (i.e., 2 h before the beginning of light period) and dusk (i.e., 2 h before the beginning of dark period) under control condition (WL, white light) and various light quality conditions (BL, blue light; RL, red light; and FRL, far-red light) and light intensity conditions (DG, dark grown; LL, low-light intensity; and HL, high-light intensity).”

For instance, the authors cite a variety of papers focusing on more metabolic or physiological read outs of how CAM plants respond to light quality or intensity; yet at a glance (and please correct me if I'm wrong) none of these cited papers is very comparable in species and experimental design to what was presented here. In as much, I found myself at a loss to basic questions like (A): does the blue/red light treatment disrupt signaling and CAM cycle integration enough that carbon fixation is substantially reduced over the time period (e.g. does one expect a mixture of _hunger_ and _light signalling_ response from the plants).
[Response]: We added the following text to the Discussion section (line 415-424):

"In comparison with white light, blue light repressed the expression of three genes (β-CA, PEPC and ALMT) involved in dark CO2 fixation and malate transport as well as two genes (PPDK and PPDK-RP) in the light phase of CAM pathway at dusk (i.e., 2 h before the beginning of dark period) (Fig. 7a). Similarly, red light/far-red light repressed the expression of three genes (PEPC, MDH and ALMT) involved in dark CO2 fixation and malate transport as well as one gene (PPDK) in the light phase of CAM pathway at dusk (i.e., 2 h before the beginning of dark period) (Fig. 7a). This monochrome light-induced gene repression was consistent with the much lower acid accumulation (i.e., dawn-dusk ΔH+) under blue/red/far-red light conditions (Fig. 6b). These results indicate that blue/red/far-red light treatment interfere with the optimal performance of the CAM pathway in K. fedtschenkoi."

Or (B): is the high light stressing the plant (e.g. causing excess reactive oxygen species production and high non-photochemical quenching), or is it perhaps still within it's normal physiological range / not yet necessarily saturating the photosynthetic machinery?
[Response]: We added the following text to the Discussion section (line 404-414):
"We found that high level (1,000 mol m-2 s-1) of white light increased expression of three dark CO2 fixation genes (i.e., β-CA, PPCK, PEPC) and one malate transporter gene (ALMT) at dawn (i.e., 2 h before the beginning of light period) in comparison with intermediate level (440 mol m-2 s-1) of white light (Fig. 7a). This increased transcript abundance of genes involved in dark CO2 fixation and malate import into the vacuoles was consistent with the higher acid accumulation (i.e., dawn-dusk ΔH+) under high light relative to intermediate light intensity (Fig. 6b). On the other hand, we found that high level of white light repressed expression of several CAM pathway genes (e.g., PEPC, MDH, PPDK, PPDK-RP) at dusk (i.e., 2 h before the beginning of dark period) in comparison with intermediate level of white light (Fig. 7a). These results suggest that the high-light treatment was still within the normal physiological range of the plant and not yet necessarily saturating the photosynthetic machinery."

I appreciate that the authors can neither know nor address the questions of each individual reader, but providing a few basics would be very helpful.

In particular, if the authors still have material in the freezer:
Measurements of titratable acidity and starch and sugar content, would provide a (comparatively) easy and reliable way to check the overall functioning of the CAM cycle.
[Response]: Yes, we have added the nocturnal malic and citric acid accumulation, soluble sugar content and starch content in new Figure 6 and the relevant description in Results and Discussion sections.

If the authors are able to reliably reproduce their setup:
Measurements on live plants of photosynthetic traits/activity (such as gas-exchange, Fv/Fm, NPQ), would help a lot in understanding what's ultimately active in the plant.
Where further analyses are unfeasible, it would help a lot if the authors would bring to bear more literature or background information (e.g. simply knowing the typical light intensity the plant experiences in the wild would go a ways towards helping answer question B, above).
[Response]: Thanks for your suggestion. Unfortunately, at the moment we do not have enough living plants for the photosynthetic traits' measurement. It will take at least another six months to prepare the materials. Although we cannot include living plant photosynthetic result in current study, we have added titratable acidity and sugar and starch results in the new Figure 6 to provide the physiological clues for understanding the association between genes and physiological traits. We believe it will not affect the conclusion for this study. Thanks for your consideration!

2-
This dataset certainly provides intrinsic value to the scientific (and particularly the CAM) community. But the analyses they have performed on the data unfortunately strike me as primarily untargeted and thin.
Don't get me wrong, I appreciate the data overview, and very much like seeing that the replicates cluster closely in the PCA, but there are a couple reasons (in my opinion) to provide some more targeted analyses on top.
First, the authors presumably generated this dataset in order to answer particular

questions of interest to their lab. Perhaps they were unable to answer their questions (it happens) or perhaps the dataset was used to generate candidates which will be analyzed one by one for years down the line. Fair enough.
Second however, (and not unrelated to major point #1) anyone else considering working with this data probably wants to have some sort of general summary of what is happening in this dataset before they know if they could / should use it for their own research. In my experience this is true from people looking up their favorite gene of interest in an eFP browser to people looking at including the study in some large-scale meta analysis.
Understanding the data quality goes beyond a bit of clustering and co-expression.
In as much, I would highly encourage the authors to spend less of the paper talking about different clustering runs or numbers of differentially expressed genes and instead focus some on providing a functional summary of what is happening in the plants. In particular, the authors state that they provide "a novel genomics resource for investigating the molecular mechanism underlying the light regulation in CAM plants". So please, walk us through some examples:
- do we see the same pathways responding to the phytochromes as we would expect from other plants in red/blue light?
[Response]: According to your suggestions, we have added a new Figure 8 to indicate the expression patterns of photoreceptors in Kalanchoë leaves.

- do we rather see stress response or photosynthesis going up in high light?
[Response]: Based on the GO enrichment analysis of DEGs under high light (Figure 2), we didn't find the stress response going up in high light. We also compared the expression of CAM-related photosynthetic genes in response to light intensity (Figure 6b), we found several genes were constitutively up-regulated with the increasing of the light intensity at dawn or dusk (such as MDH and ALMT). We have added the additional analysis (subnetwork of MDH in Figure 6c) and the relevant description in Section of Results and Discussion.

- dark grown plants show very few DEGs between Dusk/Dawn, is the clock still working properly or already strongly tapering?
[Response]: Most of the circadian rhythm-related genes such as CCA1, CRY2, ELF3/4, HY5, PRR7/9, RVE1/6/8 and TOC1 have disordered expression patterns between dawn and dusk under DG (Supplementary Fig. S7), indicating that circadian clock regulation is strongly repressed under DG.

These are just a few examples, I'd be happy to see anything the authors think is important in understanding the data and in making sure the plants are responding give or take as expected to the treatment.

However, considering the introduction and experimental design, I would strongly encourage the authors to consider making a graphic for what's known about molecular light response, not unlike what they did with the CAM pathway; and generally discuss if the observed expression here is in line with what is known from other plants/studies or not.
[Response]: According to your suggestions, we have added a new Figure 8 to indicate the expression patterns of photoreceptors in Kalanchoë leaf. We tried to find the similar expression dataset from other plant species, but we can only find a dataset of Arabidopsis which used seedlings treated 45 min and 4 h under BL, RL or FRL conditions. Concerning the differences of detected tissue and treatment time, we just included the 4h treatments data in Arabidopsis and compared it with our dusk data (10h treatment) in Kalanchoë. In addition, we analyzed the subnetworks of specific photoreceptors (Fig. 8c and d). The results indicate that conserved and novel regulatory mechanisms existed in circadian rhythm and light response in CAM plant Kalanchoë.

I'm sorry to brush aside the GO-enrichments and the bubble plots here but I'm looking for a summary, not a data-dump.
What the authors have provided in terms of functional analyses could be modified to make it more helpful.
I would need the enrichments of DEGs to be broken down by up/down regulated to make sense of them.
I'd like to see terms like "response to endogenous stimulus" or "cellular processes" be

pre-processed by the author's a little bit (if and where they are focused on in the prose) so the reader knows what major sub-terms or perhaps even what a few example genes are that are found in this term with this expression pattern. The reader can't readily connect the WGCNA clusters to an actual expression pattern from what I can see? So I really don't know what to make of these enrichments.
Is it necessary to put molecular function or cellular compartment into the same plots? Or could some of this be transferred to the supplement, so the rest could be larger and interpreted more readily?
[Response]: Yes, we have pointed out the key terms of the enrichment analysis in the results.
As well as all DEGs, we also performed the functional enrichment analysis of DEGs with increased or decreased relative transcript abundance patterns (Supplementary Figure S2 and Table S3).
According to your suggestion, we have added the expression patterns of each WGCNA clusters in new Figure 4, and have listed the GO enrichment of each cluster. The full list of GO enrichment including biological process, molecular function and cellular compartment were listed as Supplementary Table S3 and S5. We just provided the GOslim terms (top levels of GO terms) in the main text figures.

3-
The clustering is a bit overwhelming. I counted _7_ clustering analyses, _5_ of which were directly or indirectly clustering by gene.
It's not that I've never been there, but using this many different rounds/forms of clustering is not making it easier for the reader to follow and I expect it is not making it easier for the authors to analyze the data themselves either.
Here I would suggest a few things:
First, at Figure 4b and figure 5c,d-
I do not see much added value in re-clustering the clusters. This is redundant and confusing for the reader. Particularly so for the re-clustering of the WGCNA clusters, which, as WGCNA implements a type of hierarchical clustering, _already_ have relative positioning.
[Response]: We have removed the original Figure 4b and Figure 5c,d according to your suggestion.

Second, even after removing the clusters of clusters, there would still be several occasions where the authors cluster the same basic information multiple times
- PCA & hierarchical clustering of the samples
- k-means, WGCNA hierarchical, and MDS clustering of the DGEs.
[Response]: We have removed those confusing cluster analyses. We only left PCA clustering to indicate the replicates cluster closely and WGCNA analysis to indicate the co-expression relationships.

I would encourage the authors to think carefully about the different methods and the characteristics / unique value of each and whether they really need (or even particularly talk about) so many different types.
My impression is that the authors could have presented the same essential "story" with only the WGCNA and PCA clustering, assuming at least, that they provided the expression pattern for the WGCNA clusters. But it is not unlikely that I missed something.
[Response]: Thanks for your comments. According to your suggestions, we removed those confusing cluster analyses. We only left PCA clustering to indicate the replicates cluster closely and WGCNA analysis to indicate the co-expression relationships. In the new Figure 5, we have added expression patterns of different modules in panel (b).

Inline / minor comments -
Title, pg 1, line 2-
As far as I have seen "expression Atlas" used before, it has normally referred to a wide survey of different tissues / developmental stages. I would recommend choosing a more precise term for clarity as the presented data is all from mature leaves.
[Response]: Yes, we have revised the title as “Light-responsive expression atlas reveals the effects of light quality and intensity in Kalanchoë fedtschenkoi, a plant with crassulacean acid metabolism”

page 4, line 89-

Is "metabolic synchronization" some sort of standard term? If not, it might be better to write something more descriptive.
[Response]: "metabolic synchronization" was changed to “metabolic changes”

page 5, line 115-
Ultimately the gigabytes of data produced is subject to the selected compression method. Personally, I prefer to know the number (and of course length) of reads/read pairs that were produced.
[Response]: Yes, we have added the detailed information including reads number, unique/multiple mapped reads and mapping rates in the updated Table S1, and revised the gigabytes to the number of read pairs. We added the following text to Line 137: “In total, we obtained 981 million read pairs (2×150 bp) with 287.78 Gb high quality data (Qscore≥25) from the 42 libraries, with an average size of ~23.4 million read pairs per library (Supplementary Table S1).”

page 5, line 121-
The use of the word 'cultured' here makes it sound like plants had been cultivated long-term under the different light conditions instead of simply having had two days to acclimate.
[Response]: We have revised it as “treated”.

page 5, line 131-
Two things: first, "obvious" is not a sufficient reason to throw out "outliers".
Second, I must beg to differ that they were obvious at all, I had to stare at Supplemental Fig. 1b for a while to figure out what the authors were even seeing.
The authors' data is all for mature leaves, and excepting dark grown, it seems to all be quite healthy / active. Considering this, the clustering is very nice and actually separates samples better than I would have expected.
Please put the replicates back in for all the analyses!
[Response]: Yes. Thanks for your suggestion. We have put all the replicates back in the revised manuscript. All the analyses were re-analyzed based on the updated data.

page 6, line 146-
Are the few saved characters really worth it to write "C1_1" instead of giving the comparisons a name that doesn't require a reference figure (e.g. WL_Dawn-vs-Dusk)?
[Response]: Yes, we have replaced the comparison label according to your suggestion.

page 6, line 158-
I would discourage over interpretation of different numbers of DGEs. Yes, you can get fewer DGEs because a pair of samples were more similar to each other than the reference pair, but it can also simply be that there was a little more variance between the replicates.
[Response]: Yes, we have removed the excessive descriptions in this section.

page 7, line 166-
"may be" => "might" or "maybe"
[Response]: Revised as suggested.

page 7, line 176-
fix "(cite a figure or table here)"
[Response]: Fixed, with the relevant excessive description removed.

page 9, line 246-
The authors have projected thousands of DGEs from 39 clusters down to two dimensions, of course there is some overlap. No need to cluster again.
[Response]: Yes, we have removed the two dimensions MDS plot and the following clustering analysis.

page 30, Fig. 7-
The authors might be able to focus more on the expression patterns that are best proxies for the enzymes displayed if this figure more directly represented absolute expression. Either, focusing on the highly expressed paralog(s), summing up the expression of paralogs, or displaying some form of log(TPM) so that the lowly / highly

	expressed paralogs could be identified. If we're lucky and which paralogs are involved in the CAM cycle is established, the authors could focus only on these.<br>[Response]: Thanks for your suggestion, we have removed the heatmap in Figure 7 and use TPM value to generate the line plots in the revised Figure 7.<br><br>page 30, line 722-<br>fold change vs what? I presume it's the average, but this should be stated.<br>[Response]: we have removed the heatmap in Figure 7 and use TPM value to generate the line plots in the revised Figure 7.<br><br>-- End --
**Additional Information:**	
**Question**	**Response**
Are you submitting this manuscript to a special series or article collection?	No
**Experimental design and statistics**<br><br>Full details of the experimental design and statistical methods used should be given in the Methods section, as detailed in our Minimum Standards Reporting Checklist. Information essential to interpreting the data presented should be made available in the figure legends.<br><br>Have you included all the information requested in your manuscript?	Yes
**Resources**<br><br>A description of all resources used, including antibodies, cell lines, animals and software tools, with enough information to allow them to be uniquely identified, should be included in the Methods section. Authors are strongly encouraged to cite Research Resource Identifiers (RRIDs) for antibodies, model organisms and tools, where possible.<br><br>Have you included the information requested as detailed in our Minimum Standards Reporting Checklist?	Yes
**Availability of data and materials**<br><br>All datasets and code on which the	Yes

conclusions of the paper rely must be either included in your submission or deposited in publicly available repositories (where available and ethically appropriate), referencing such data using a unique identifier in the references and in the "Availability of Data and Materials" section of your manuscript.<br><br>Have you have met the above requirement as detailed in our Minimum Standards Reporting Checklist?	

Powered by Editorial Manager® and ProduXion Manager® from Aries Systems Corporation

Manuscript
Click here to access/download;Manuscript;Jin et al Kalanchoe_Light_mainText_draft6.5_gigaSci_Fig.docx
Click here to view linked References

Article type: Research Article

Light-responsive expression atlas reveals the effects of light quality and intensity in *Kalanchoë fedtschenkoi*, a plant with crassulacean acid metabolism

Jin Zhang[1,2,a], Rongbin Hu[1,a], Avinash Sreedasyam[3], Travis Garcia[4], Anna Lipzen[5], Mei Wang[5], Pradeep Yerramsetty[4], Degao Liu[1], Vivian Ng[5], Jeremy Schmutz[3,5], John C. Cushman[4], Anne M. Borland[1,6], Asher Pasha[7], Nicholas J. Provart[7], Jin-Gui Chen[1,2], Wellington Muchero[1,2], Gerald A. Tuskan[1,2], Xiaohan Yang[1,2,*]

[1]Biosciences Division, Oak Ridge National Laboratory, Oak Ridge, TN 37831-6422, USA;
[2]The Center for Bioenergy Innovation, Oak Ridge National Laboratory, Oak Ridge, TN 37831, USA;
[3]HudsonAlpha Institute for Biotechnology, 601 Genome Way, Huntsville, AL 35801, USA;
[4]Department of Biochemistry and Molecular Biology, University of Nevada, Reno, NV 89557, USA;
[5]US Department of Energy Joint Genome Institute, 2800 Mitchell Drive, Walnut Creek, CA 94598, USA;
[6]School of Natural and Environmental Science, Newcastle University, Newcastle upon Tyne NE1 7RU, UK;
[7]Department of Cell and Systems Biology, Centre for the Analysis of Genome Evolution and Function, University of Toronto, Toronto, ON M5S 3B2, Canada

[a] These authors contributed equally to this manuscript.
*Correspondence: Xiaohan Yang (yangx@ornl.gov) Tel +1 865 241 6895; fax +1 865 576 9939

Emails: Jin Zhang (zhangj1@ornl.gov), Rongbin Hu (hu.rongbin@gmail.com), Avinash Sreedasyam (asreedasyam@hudsonalpha.org), Travis Garcia (tgarcia2@unr.edu), Anna Lipzen (alipzen@lbl.gov), Mei Wang (mwang@lbl.gov), Pradeep Yerramsetty (ypradeepkalyan@gmail.com), Degao Liu (liudegao909@gmail.com), Vivian Ng (vng@lbl.gov), Jeremy Schmutz (jschmutz@hudsonalpha.com), John C. Cushman (jcushman@unr.edu), Anne M. Borland (anne.borland@newcastle.ac.uk), Asher Pasha (asher.pasha@utoronto.ca), Nicholas J. Provart (nicholas.provart@utoronto.ca), Jin-Gui Chen (chenj@ornl.gov), Wellington Muchero (mucherow@ornl.gov), Gerald A. Tuskan (tuskanga@ornl.gov), Xiaohan Yang (yangx@ornl.gov)

Figures: 8 figures (in color).
Supplementary Data: 8 supplementary figures and 9 supplementary tables.

Abstract

Background: Crassulacean acid metabolism (CAM), a specialized mode of photosynthesis, enables plant adaptation to water-limited environments and improves photosynthetic efficiency via an inorganic carbon-concentrating mechanism. *Kalanchoë fedtschenkoi* is an obligate CAM model featuring a relatively small genome and easy stable transformation. However, the molecular responses to light quality and intensity in CAM plants remain understudied.

Results: Here we present a genome-wide expression atlas of *K. fedtschenkoi* plants grown under 12h/12h photoperiod with different light quality (blue, red, far-red, white light) and intensity (0, 150, 440, and 1000 μmol m^{-2} s^{-1}) based on RNA-Seq performed for mature leaf samples collected at dawn (2-h before the starting of lighting period) and dusk (2-h before the dark period). An eFP web browser was created for easy access of the gene expression data. Based on the expression atlas, we constructed a light-responsive co-expression network to reveal the potential regulatory relationships in *K. fedtschenkoi*. Measurements of leaf titratable acidity, soluble sugar and starch turnover provided metabolic indicators of the magnitude of CAM under the different light treatments and were used to provide biological context for the expression dataset. Furthermore, CAM-related subnetworks were highlighted to showcase genes relevant to CAM pathway, circadian clock and stomatal movement. In comparison with white light, monochrome blue/red/far-red light treatments repressed the expression of several CAM-related genes at dusk, along with a major reduction in acid accumulation. Increasing light intensity from an intermediate level (440 μmol m^{-2} s^{-1}) of white light to a high light treatment (1000 μmol m^{-2} s^{-1}) increased expression of several genes involved in dark CO_2 fixation and malate transport at dawn, along with an increase in organic acid accumulation.

Conclusions: This study provides a highly useful genomics resource for investigating the molecular mechanism underlying the light regulation of physiology and metabolism in CAM plants. Our results support the hypothesis that both light intensity and light quality can modulate the CAM pathway through regulation of CAM-related genes in *K. fedtschenkoi*.

Keywords: eFP browser; gene atlas; transcriptome; *Kalanchoë fedtschenkoi*; crassulacean acid metabolism

Background

Sunlight is a critical energy resource for plant growth and development, and functions as an important input signal for circadian clock, stomatal movement, and photosynthesis pathway. The light spectra that affect plant photosynthesis are UV-A/blue, red, and far-red [1, 2]. Blue light, with wavelength of 400 to 500 nm, has a higher energy than red light (wavelength from 600 to 700 nm) and far-red light (wavelength above 700 nm) [1, 3]. There are three types of photoreceptors (i.e., cryptochromes, phototropins and phytochromes) that perform important roles in plant light response [2, 4]. Cryptochromes and phototropins have been identified as important photoreceptors of UV-A/blue light [2, 5, 6]. Phytochromes are known to play a role in detecting red and far-red spectra [2, 7]. In addition to the light quality, light intensity is another essential factor that affects plant growth and development, where either too much or little light can cause stress, including serious damage to photosynthetic apparatus under excess light exposure and limited photosynthetic activity with insufficient light input [8-10].

Plants using crassulacean acid metabolism (CAM) pathway for photosynthesis show enhanced water-use efficiency (WUE) and heat/drought stress tolerance in comparison with C_3 and C_4 photosynthesis plants [11, 12]. The CAM pathway has two major features: (1) a carboxylation process that takes place at night where stomata are open for nocturnal CO_2 fixation and accumulation of malic acid in the vacuole and (2) a decarboxylation process that occurs during the daytime where CO_2 is released from malate for refixation via ribulose-1,5-bisphosphate carboxylase/oxygenase (Rubisco)-mediated photosynthesis, along with stomatal closure which reduces evapotranspiration [11, 13, 14]. *Kalanchoë fedtschenkoi* is a model dicot CAM species, featuring a relatively small genome and a facile stable transformation system [11, 15]. The genome of *K. fedtschenkoi* was recently sequenced and annotated [13], providing a foundation for CAM genomics research. Comparative and evolutionary genomics analyses revealed convergent signatures in diel gene expression pattern and protein sequences underlying independent emergences of CAM from C_3 ancestor, providing new insights into CAM evolution [13]. However, the complex regulatory mechanisms that underpin the diel optimization of the CAM pathway under various light conditions remain largely unexplored.

The temporal separation of C_3 and C_4 carboxylation processes that defines CAM provides plasticity for optimizing carbon gain and water use in response to changing environmental

conditions by extending or curtailing the period of net CO_2 uptake over a 24-h period [16]. Light intensity (photosynthetic photon flux density, PPFD) and light quality are critical factors for controlling the performance of CAM, which implies cardinal roles for the light reactions of photosynthesis and for different photoreceptors in achieving metabolic and circadian synchronization of carboxylation processes across the diel cycle. In some facultative CAM species, high-light intensity can trigger the switch from C_3-photosynthesis to CAM, which is mediated by a UV-A/blue light receptor [17]. Metabolic and physiological adaptation in constitutive CAM species plants to light quantity and quality has been reported previously [18-21]. For instance, physiological and metabolic responses under severe light stress under short- and long-term treatments were reported [18]. Metabolic changes under different light spectrum (i.e., blue, green and red light) were also reported in an obligate CAM species *Aechmea 'Maya'* [19]. Exposure of a woody CAM species *Clusia hilariana* to low- or high-light affected the production of malate and citrate [21]. However, the molecular basis of the metabolic and signaling pathways that underpin changes to the operation of CAM in response to various spectral light qualities and intensity has not been investigated extensively.

High-throughput, next-generation sequencing has been widely applied to genome-wide expression analysis. As a new type of web-based tool, a genome-wide atlas of gene expression can provide comprehensive gene expression profiles in different tissues or different development stages. To date, gene expression atlases have been established for several C_3 photosynthesis species, including dicot *Arabidopsis* [22], *Medicago* [23], tomato [24], monocot wheat [25], and *Brachypodium* [26]. However, a genome-wide expression atlas has not been created for a CAM species. In addition, gene expression patterns and gene modules associated with plant response to light quality and light intensity are largely unknown, especially for CAM plants.

We hypothesize that both light intensity and light quality can influence the expression of CAM-related metabolic and signaling genes in the obligate CAM species *K. fedtschenkoi*. To test this hypothesis, we performed transcriptome-sequencing (RNA-Seq) of mature *K. fedtschenkoi* leaf samples collected at dawn (i.e., 2-h before the starting of lighting period) and dusk (2-h before the dark period) from plants grown under 12h/12h photoperiod with different light quality (i.e., blue, red, far-red, white light) and intensity (0, 150, 440, and 1000 μmol m^{-2} s^{-1}). Based on our analysis of the RNA-Seq data, we generated a comprehensive light-responsive gene expression atlas for

this obligate CAM species. We also constructed a genome-wide co-expression network based on the light-responsive gene expression atlas. To provide a metabolic context for the genome-wide co-expression network, we measured the titratable acidity (nocturnal malate and citrate accumulation) and soluble sugar and starch turnover. Nocturnal acid accumulation provides a quantitative measure of CAM activity whilst diel turnover of sugars and starch, provides an indication of the nocturnal supply and day-time demand for carbon processing. The subnetworks of CAM-related genes, photoreceptors, and stomatal movement-related genes indicated that the light-responsive expression atlas provides molecular clues for various physiological phenotypes. As comprehensive light-responsive gene atlas and co-expression network for CAM plants, this study provides an unprecedented genomics resource for investigating molecular mechanisms underlying the light regulation of biological processes in CAM plants.

Data Description

A total of 42 libraries (7 light conditions × 2 time points × 3 biological replicates) were constructed and performed RNA-seq independently. In total, we obtained 981 million read pairs (2×150 bp) with 287.78 Gb high quality data (Qscore≥25) from the 42 libraries, with an average size of ~23.4 million read pairs per library (Supplementary Table S1).

Analyses

Light-responsive expression atlas for *K. fedtschenkoi*

To obtain a comprehensive light-responsive gene-expression atlas of the CAM plant *K. fedtschenkoi*, we treated plants under control condition [white light (WL) with 440 μmol m^{-2} s^{-1} intensity], various light quality including blue light (BL), red light (RL), far-red light (FRL), and different light intensities, including dark grown (DG), low-light (LL) and high-light (HL) (Supplementary Table S1). As CAM pathway is likely regulated by the circadian clock, we compared if circadian rhythm-related processes were also affected by different light conditions. The samples were collected at two time points [dawn (2 h before light period) and dusk (2 h before dark period)] for each light condition. To provide easy access to the expression data, we created a

Kalanchoë light-responsive eFP browser (http://bar.utoronto.ca/efp_kalanchoe/cgi-bin/efpWeb.cgi), which provides a color-coding tissue visualization in an image corresponding to the average gene expression level (Fig. 1).

The Pearson correlation analysis and principle component analysis showed that the biological replicates of each treatment group were closely clustered, indicating the high reproducibility and reliability of our RNA-seq data (Fig. 2 and Supplementary Fig. S1). The principal component 1 (PC1) and PC2 explained 30.9% and 25.5% of the variance in the expression data, respectively. As expected, the samples collected at dawn and dusk were grouped separately under different light quality and light intensity except dark grown (Fig. 2b), and the expression variation of samples under various light conditions was stronger at dawn than the variation at dusk.

Differentially expressed genes (DEGs) regulated by light quality and light intensity

As shown in Fig. 2a, we performed a comparative transcriptomic analysis for screening of DEGs by using two different strategies (i.e., time comparison and light condition comparison). The time comparison was defined as the comparison between two samples collected at two different time points (i.e., dawn and dusk) under each light condition (i.e., dusk *vs*. dawn). The light condition comparison reflected a comparison between treatments and control at the same sample collection time point (i.e., BL/RL/FRL *vs*. WL) at dawn or dusk for light quality (i.e., DG/LL/HL *vs*. WL) at dawn or dusk for light intensity, respectively (Fig. 2a and Supplementary Table S2).

Under normal light condition (WL), 6,412 DEGs were identified in dusk vs. dawn. Of these DEGs, 3,137 and 3,275 genes showed either increased or decreased relative transcript abundance, respectively (Fig. 2). For different light intensity, both the low- and high-intensity light treatments enhanced the differential gene expression between dusk and dawn. Under the dark-grown condition, only 631 DEGs (458 increased and 173 decreased in abundance) were identified in dusk *vs*. dawn comparison, which was significantly less than the comparisons made under the other light conditions (Fig. 2 and Supplementary Table S2).

Under the different light qualities, a total of 2,480 DEGs between dusk and dawn were shared by the four light spectrums (i.e., WL, BL, RF and FRL), indicating these genes might play essential roles in response to changes in light quality (Fig. 3a). Under various light intensity conditions,

3,090 genes were consistently differentially expressed under the three light intensities (WL, LL and HL), suggesting that these genes might play key roles in responding to diel or circadian cues and were not affected by light intensity (Fig. 3b).

Light condition comparisons were based on differences between different light quality or light intensity (Fig. 2a). The DEG number in most light-quality comparisons was greater at dawn than those at dusk (Fig. 2d). However, the overlapped DEGs were fewer at dawn (860 common DEGs, Fig. 2f) than at dusk (1,334 common DEGs, Fig. 2f) under the different light quality treatments. In contrast, the light quality-specific DEGs were greater at dawn than at dusk under blue light and far-red light. For the different light intensities, more than half of the DEGs under low light and high light were shared at both dawn and dusk (Fig. 2f).

Predicted function of DEGs

To explore the functional differences of DEGs induced by various light quality and light intensity treatments, we performed a gene ontology (GO) enrichment analysis of DEGs in different comparisons according to the three major GO categories of biological process (BP), molecular function (MF), and cellular component (CC) (Supplementary Fig. S2). Notably, all DEGs in the light intensity and light quality comparisons were enriched in "photosynthesis" process; but when the increased or decreased abundance-DEGs were separated, only increased abundance-DEGs in light quality comparisons and down-DEGs in light intensity comparisons were highly enriched in "photosynthesis" process. Similarly, the "response to biotic stimulus" term in light quality for all DEGs was depleted (Supplementary Fig. S2 and Table S3).

In addition, we compared the enriched GO terms of common and light-specific DEGs in response to the light treatments (Fig. 2g and Supplementary Table S4). Among the DEGs between dusk and dawn, the 2,480 DEGs that overlapped across different light-quality treatments (Fig. 2f) were enriched in "carbohydrate metabolic process", "lipid metabolic process", "metabolic process" and "signal transduction"; whereas the 3,090 DEGs that overlapped across different light-intensity treatments were enriched in "carbohydrate metabolic process" and "response to endogenous stimulus" (Fig. 2g). When comparing the common DEGs in different light quality or light intensity at dawn and dusk separately (Fig. 2f), we found that "generation of precursor metabolites and energy" term was enriched in all the four common DEGs sets of different light quality and light

intensity at both dawn and dusk. In contrast, the "photosynthesis" term was enriched in common DEGs of light quality at dusk and common DEGs of light intensity at both dawn and dusk (Fig. 2g), whereas the "carbohydrate metabolic process" term was only enriched at the dusk time point of different light quality and light intensity (Fig. 2g).

For condition-specific DEGs at different time points or different light conditions, the "photosynthesis" term was strongly enriched in dusk *vs*. dawn DEGs in RL-specific and HL-specific from the light-quality comparison and light-intensity comparison, respectively (Fig. 2g). This indicates that RL and HL significantly affected photosynthesis-related changes in transcript abundance between dawn and dusk. When different light quality or light intensity treatments were compared to the white light control at dawn and dusk, the "photosynthesis" term was also enriched in the DG *vs*. WL (Fig. 2f) and HL *vs*. WL comparisons (Fig. 2g) at both dawn and dusk, indicating HL and DG strongly affected photosynthesis-related changes in transcript abundance independent of dawn or dusk sampling.

To further understand the functional differences of DEGs under different light quality and intensity treatments, we then classified the DEGs into hierarchical categories 'BINs' using MapMan. Based on the DEG number and percentage in each BIN, we found that the percentage of "photosynthesis" class genes was higher in the comparison "DG_Dusk-vs-Dawn" than the other comparisons (Supplementary Fig. S3). And the expression pattern of "photosynthesis" class genes showed decreased abundance in samples of DG_Dawn and DG_Dusk (Supplementary Fig. S4). To better understand the gene expression patterns, we mapped the DEGs in the photosynthetic pathway. As shown in Fig. 3, most of the DEGs with functions involved in the light reactions (e.g., LHC-II, PS-II, Cytb6, LHC-I/PS-I and FNR), Calvin cycle (e.g., Rubisco, phosphoglycerate kinase, glyceraldehyde 3-phosphate dehydrogenase and fructose-1,6-bisphosphatase), and photorespiration (e.g., phosphoglycolate phosphatase, glycolate oxidase and glycine decarboxylase) showed decreased abundance in DG_Dawn and DG_Dusk. However, glycerate kinases in photorespiration were highly expressed in DG_Dawn and DG_Dusk.

Co-expression network

To determine the relationships among genes responsive to different light quality and light intensity treatments in *K. fedtschenkoi*, we constructed a WGCNA co-expression network using

the DEGs identified from the previous comparisons (Fig. 2). After combining the modules with highly similar expression patterns, a total of 13 co-expression modules were obtained and labelled as different colors (Fig. 4a). The module size ranged from 609 genes (module 'cyan') to 3,047 genes (module 'turquoise'). Among the 13 modules, modules 'blue' and 'salmon' showed low expression at dawn and high expression at dusk under most of the light conditions, except module 'blue' that showed low expression and module 'salmon' that showed high expression under DG. Based on the GO enrichment analysis, module 'blue' was enriched in "oxidoreductase activity" but no significantly enriched GO terms were identified in module 'salmon'. Module 'yellow' showed the opposite expression pattern with modules 'blue' and 'salmon', which showed high expression at dawn and low expression at dusk and was functionally enriched in "transport". The expression of modules 'magenta' and 'purple' were induced at dawn by LL and HL, but the induction of module 'purple' was stronger under HL. These two modules were functionally enriched in "chloroplast" (module 'magenta') and "proteasome complex" (module 'purple'), respectively. Enriched TF analysis indicated that MYB TFs in module 'purple' was stronger than that in module 'magenta' (Supplementary Fig. S5). Two opposite modules 'tan' and 'turquoise' showed low and high expression under DG, which were enriched in "photosynthesis" and "phosphorylation", respectively (Fig. 4 and Supplementary Table S5).

To further understand the response of different pathways in CAM plants under various light quality and light intensity treatments, we extracted sub-networks from the global co-expression network. Here, we selected genes related to CAM, the circadian clock, and stomatal movement [13] as case studies to demonstrate the sub-networks. In order to simplify the sub-network, we set a high threshold of Pearson correlation coefficient ($|PCC|>0.95$ and $P \leq 0.01$) to show the strong co-expression relationships. The genes involved in CAM, circadian clock, and stomatal movement pathways were highly associated and were co-expressed with numerous transcription factors (TFs) (Fig. 5), implying that the expression of CAM pathway genes may be directly or indirectly regulated by circadian clock TFs. Based on the sub-network, we identified several known and novel TFs that were related with these pathways. For instance, *LHY1* (*Kaladp0066s0115*) was positively co-expressed with *CCA1* (*Kaladp0496s0018*, PCC=0.995), *RVE8* (*Kaladp0577s0020*, PCC=0.993) and *RVE1* (*Kaladp0574s0015*, PCC=0.983); and was negatively co-expressed with *ELF4* (*Kaladp0045s0206*, PCC=-0.978) and *LUX* (*Kaladp0033s0047*, PCC=-0.969). Similarly,

MYB96 (*Kaladp0095s0568*) and *WRKY4* (*Kaladp0096s0082*) were positively co-expressed with *CCA1* and *RVE8* and were negatively co-expressed with *LUX* (Fig. 5 and Supplementary Table S7). In addition, several TFs not previously reported to be associated with CAM were identified in the sub-network, such as *LZF1* (*Kaladp0192s0026*), *SOC1* (*Kaladp0016s0148*), *CDF2* (*Kaladp0009s0042* and *Kaladp0095s0211*), *COL4* (*Kaladp0029s0144*), *ZFP4* (*Kaladp0035s0036*), *ZFP7* (*Kaladp0001s0233*), *SIG1* (*Kaladp0538s0007*), *SIG4* (*Kaladp0515s0145*), and *SIG5* (*Kaladp0055s0328*).

Metabolic changes of *K. fedstchenkoi* response to light quality and light intensity

To explore if this light-responsive expression atlas could provide molecular clues for metabolic changes that are diagnostic of CAM activity, we measured leaf titratable acidity (nocturnal malate and citrate accumulation) and diel turnover of soluble sugars and starch. Nocturnal malic and citric acid accumulation was assessed by the difference in H^+ concentration between dawn and dusk samples (ΔH^+). Under full-spectrum light, ΔH^+ increased with the intensity of light, indicating elevated CAM activity. The levels of nocturnal malate and citrate accumulation for these samples was comparable to that observed under acclimating conditions. In contrast, filtered monochrome light (i.e., BL, RL and FRL) and dark treatment dampened the acid accumulation. RL and FRL treatments, both at photon flux densities of 280 μmol m^{-2} s^{-1}, resulted in very low ΔH^+ values between dawn and dusk samples, indicating reduced CAM activity. Extremely low or inverted dawn/dusk ΔH^+ values were observed for BL and DG plants with higher acid accumulation in dusk samples relative to dawn samples (Fig. 6a, b).

Full-spectrum light treatments of both LL and HL resulted in the elevated quantities of soluble sugars in *Kalanchoë* leaf tissue, with somewhat higher levels observed in dusk samples relative to the corresponding dawn samples. Soluble sugar accumulation in WL was markedly lower than in LL and HL grown plants although control conditions utilized a full-spectrum intermediate light intensity. All colored-light and dark treatments resulted in dramatically reduced soluble sugar contents compared with the LL and HL treatments, which is likely a result of the lower photon flux densities delivered to the plants under these conditions (Fig. 6c). Similar to the trends noted for soluble sugars, high levels of leaf starch accumulation were observed in dusk samples for both LL and HL treatments. WL also resulted in large starch content at dusk. Significant accumulation of starch from dawn to dusk was observed for all full-spectrum samples. Lower starch contents

were observed under all filtered light treatments with no detectable starch in dawn FRL samples or dawn and dusk DG plants. The greatest starch content observed for any filtered-light treatment was seen in dusk samples grown under RL, although these starch levels were still much lower than in plants grown under full-spectrum light (Fig. 6d). Again, these low starch accumulation patterns were likely due to the lower photon flux densities delivered to the plants under these light-filtering conditions.

To understand the potential molecular mechanisms regulating these metabolic phenotypes, we performed a "gene expression – phenotypes" correlation analysis. We selected photosynthetic genes including genes involved in light reactions, Calvin cycle and photorespiration (Fig. 3) and CAM pathway genes including CAM-, circadian-, and stomatal-related genes (Supplementary Table S8) as the candidate genes. Due to the highly positive correlation between titratable acidities (r = 1.00, $P < 0.001$) and between soluble sugar and starch contents (r = 0.62, $P < 0.01$) (Supplementary Fig. S6), the correlation patterns of genes with titratable acidities or genes with soluble sugar and starch contents were similar. Notably, different gene sets in the Calvin cycle or CAM pathway were positively correlated with titratable acidities and soluble sugar/starch contents. Most of the circadian-related genes were positively correlated with starch content, but negatively correlated with titratable acidity (Fig. 6e and Supplementary Table S9).

CAM-related genes responsive to light quality and light intensity

To further investigate the CAM-specific response to various light quality and light intensity treatments, the expression patterns of CAM-related genes were analyzed (Fig. 7a). The key genes that are involved in nocturnal CO_2 assimilation and malate storage include *beta-carbonic anhydrase* (*β-CA*), *phosphoenolpyruvate carboxylase* (*PEPC*), *phosphoenolpyruvate carboxylase kinase* (*PPCK*), *NAD(P)-malate dehydrogenase* (*MDH*) and *aluminum-activated malate transporter* (*ALMT*). *β-CA* (*Kaladp0018s0287*) was highly expressed at dawn under high light compared to other light quality and light intensity treatments. *PEPC1* (*Kaladp0095s0055*) showed relatively higher expression levels at dusk than dawn under different light quality and light intensity conditions, yet *PEPC1* expression levels at both dawn and dusk were repressed by filtered monochrome light (i.e., BL, RL and FRL). Similarly, the expression of *PPCK1* (*Kaladp0037s0517*) was also repressed by filtered monochrome light. *MDH2* (*Kaladp0001s0257*) was constitutively induced by increasing light intensity at dawn; but had a peak value under WL at dusk (Fig. 7a).

During daytime, the CO_2 release from malate and refixation is mediated by a series of genes that include *tonoplast dicarboxylate transporter* (*TDT*), *NAD(P)-malic enzyme* [*NAD(P)-ME*], and *pyruvate phosphate dikinase* (*PPDK*). As the first-step transporter for the daytime reactions, *TDT* (*Kaladp0042s0251*) showed an obvious trend of greater transcript abundance at dawn and down-regulation at dusk, and expression at dawn was stronger under BL and RL than under the WL control. In contrast, *NADP-ME* (*Kaladp0092s0166*) showed similar expression patterns with TDT under light intensity treatment, but its expression at dawn were low under BL and RL. *PPDK* (*Kaladp0076s0229*) was increased by light intensity increase and reached the highest level under WL at both dawn and dusk, but was decreased by filtered monochrome light (Fig. 7a). In addition, we compared the light-quality responses of CAM-related genes between *Kalanchoë* and *Arabidopsis*. The *Arabidopsis* eFP browser (light series) is the only publicly available light-quality expression data, although the sampling time is different compared to our study – *Arabidopsis* samples were collected at 4h after treatments and our *Kalanchoë* dusk samples were collected at 10h after treatments. All these CAM-related genes showed different expression patterns between *Kalanchoë* and *Arabidopsis* (Fig. 7a).

Based on our light-responsive co-expression network, we extracted the subnetwork of *MDH*. This subnetwork consists of 274 genes, 39 of which are photosynthesis-related and seven of which are TFs (Fig. 7b). Those photosynthesis-related genes in the subnetwork include photosystem I subunits (i.e., PSAL, PSAN, PSAP), photosystem II subunits (i.e., PSB17, PSB28, PSBP, PSBW and PSBX), photosystem I light harvesting complex genes (i.e., LHCA3, LHCA5). Three TFs, *AGL16* (*Kaladp0067s0150*), *SIGF* (*SIG6*, *Kaladp0872s0002*), and *PIF3* (*Kaladp0076s0003*), in this subnetwork are known regulators in photosynthesis or circadian rhythm. In addition, we compared the expression pattern of circadian rhythm-related genes at dawn and dusk under different light conditions. Most of the circadian rhythm-related genes such as *CCA1*, *CRY2*, *ELF3/4*, *HY5*, *PRR7/9*, *RVE1/6/8* and *TOC1* have disordered expression patterns between dawn and dusk under DG (Supplementary Fig. S7), indicating that the circadian clock is likely disrupted under DG.

Photoreceptors and stomatal movement-related genes responsive to light quality in *Kalanchoë*

We tested the expression patterns of photoreceptors in our *Kalanchoë* dataset. As shown in Fig. 8, blue-light receptors *PHOT1* (*Kaladp0032s0316*) and *PHOT2* (*Kaladp0055s0063*) showed increased transcript abundance under BL at both dawn and dusk. *phyA* (*Kaladp0034s0172*) showed increased transcript abundance under both RL and FRL at dusk. The expression of *PHOT1*, *PHOT2* and *phyB* at dusk (10h after treatments) in *Kalanchoë* showed similar patterns with their orthologs at 4h after treatments in *Arabidopsis* (Fig. 8b). We then constructed the subnetworks of *PHOT1* (*Kaladp0032s0316*) and *phyA* (*Kaladp0034s0172*) based on our co-expression database. Five known regulators involved in light responses were co-expressed with *PHOT1* (*Kaladp0032s0316*), which included *TIC* (*Kaladp0048s0254*) *phyA* (*Kaladp0057s0072*), *FRS8* (*Kaladp0024s0414*), *TOR* (*Kaladp0047s0074*), and *CRY2* (*Kaladp0082s0193*). Similarly, we identified six known light-responsive regulators that were co-expressed with *phyA* (*Kaladp0034s0172*), which included cryptochrome-interacting basic-helix-loop-helix TF CIB1 (*Kaladp0033s0213*), two copies of phytochrome and flowering time regulatory protein PFT1 (*Kaladp0024s0189* and *Kaladp0024s0252*), phyE (Kaladp0053s0072), phytochrome-interacting factor 3 (*PIF3*, *Kaladp0076s0003*), HY1 (*Kaladp0872s0026*). In addition, two CAM-related genes, *PEPCK* (*Kaladp0040s0194*) and *MLS* (*Kaladp0011s1037*) were co-expressed with *phyA* (*Kaladp0034s0172*) (Fig. 8c).

Most of the stomatal movement-related genes were induced by BL, especially at dawn (e.g., *ABI2*, *ALMT9*, *KAT1*, *KAT2* and *QUAC1/ALMT12*) (Fig. 9a). To explore the potential regulatory mechanism, we tested the subnetwork of *ABI2*. Notably, numerous circadian rhythm-related regulators were co-expressed with *ABI2*, which included *CCA1* (*Kaladp0496s0018*), *CCR1* (*Kaladp0018s0148*), *CCR2* (*Kaladp0020s0114*), COL4 (*Kaladp0029s0144*), three copies of *COR27* (*Kaladp0011s1228*, *Kaladp0042s0067* and *Kaladp0089s0010*), *DBB3* (*Kaladp0192s0026*), two copies of *ELF4* (*Kaladp0037s0163* and *Kaladp0045s0206*), *KT4* (*Kaladp0040s0740*), *LHY1* (*Kaladp0066s0115*), *PRR7* (*Kaladp0005s0054*), *RVE1* (*Kaladp0574s0015*), *RVE2* (*Kaladp0262s0019*), and *RVE7* (*Kaladp0262s0013*).

Discussion

As one of the most important environmental factors, light affects plant growth and development, plant physiology and metabolism [27, 28]. Although light quality effects at the metabolic and molecular levels have been studied in several plant species [29-33], genome-wide transcriptomic studies of the effects of light quality and light intensity on CAM species are lacking. Low-fluorescence red and blue light were shown to modulate the diel metabolic processes in an obligate CAM species *Aechmea* 'Maya' [19]. However, the regulatory mechanisms underpinning the metabolic reprogramming such light-induced metabolic reprogramming in CAM species remains largely unexplored.

In this study, we created a comprehensive, genome-wide, light-responsive gene expression atlas for *K. fedtschenkoi*. The eFP browser provides a useful web interface for easy data access, facilitating comparative and functional genomics research. Furthermore, the RNA-Seq data was analyzed by pairwise comparisons between different light conditions and different time points to identify DEGs in *K. fedtschenkoi*. These DEGs were then subject to clustering and co-expression analyses. A similar approach was effectively utilized to discover the regulatory networks in *Brachypodium distachyon* [26], pigeon pea [34], and chickpea [35]. Combined with functional analysis, such as GO enrichment analysis, we found that the overlapped DEGs at dusk were mainly involved in "carbohydrate metabolism" and "response to endogenous stimulus" processes, consistent with previous studies showing that light quality affects the regulation of endogenous hormone stimulus such as gibberellin, auxins, cytokinins and abscisic acid [3, 36-38].

We found that high level (1,000 μmol m^{-2} s^{-1}) of white light increased expression of three dark CO_2 fixation genes (i.e., *β-CA, PPCK, PEPC*) and one malate transporter gene (*ALMT*) at dawn (i.e., 2 h before the beginning of light period) in comparison with intermediate level (440 μmol m^{-2} s^{-1}) of white light (Fig. 7a). This increased transcript abundance of genes involved in dark CO_2 fixation and malate import into the vacuoles was consistent with the higher acid accumulation (i.e., dawn-dusk ΔH^+) under high light relative to intermediate light intensity (Fig. 6b). On the other hand, we found that high level of white light repressed expression of several CAM pathway genes (e.g., *PEPC*, *MDH*, *PPDK*, *PPDK-RP*) at dusk (i.e., 2 h before the beginning of dark period) in comparison with intermediate level of white light (Fig. 7a). These results suggest that the high-light treatment was still within the normal physiological range of the plant and not yet necessarily saturating the photosynthetic machinery.

In comparison with white light, blue light repressed the expression of three genes (*β-CA*, *PEPC* and *ALMT*) involved in dark CO_2 fixation and malate transport as well as two genes (*PPDK* and *PPDK-RP*) in the light phase of CAM pathway at dusk (i.e., 2 h before the beginning of dark period) (Fig. 7a). Similarly, red light/far-red light repressed the expression of three genes (*PEPC*, *MDH* and *ALMT*) involved in dark CO_2 fixation and malate transport as well as one gene (*PPDK*) in the light phase of CAM pathway at dusk (i.e., 2 h before the beginning of dark period) (Fig. 7a). This monochrome light-induced gene repression was consistent with the much lower acid accumulation (i.e., dawn-dusk ΔH^+) under blue/red/far-red light conditions (Fig. 6b). These results indicate that blue/red/far-red light treatment interfere with the optimal performance of the CAM pathway in *K. fedtschenkoi*.

K. fedtschenkoi is a model plant species for CAM functional genomics research [13, 15]. Our co-expression analysis highlighted a sub-network of CAM-related regulatory/signaling genes, such as *LHY1*, which was positively co-expressed with *CCA1*, *RVE1* and *RVE8*, but was negatively co-expressed with *ELF4* and *LUX* (Fig. 5). In *Arabidopsis*, ELF4, ELF3, and LUX can form an ELF4-ELF3-LUX protein complex (the evening complex), which is regulated by light and the circadian clock [39]. MYB-related protein CCA1 and LHY1 can form homodimers and regulate the expression of evening-element-containing genes [40]. There is a negative-feedback loop among these transcription factors. EFL4 and LUX are required for the red-light induction of *CCA1* and *LHY1*, whereas CCA1 and LHY1 negatively regulate the expression of ELF4 [41] and LUX [42]. The co-expression relationships of these TFs in *K. fedtschenkoi* reported here indicate that the circadian rhythm regulatory mechanism among these genes is conserved between *K. fedtschenkoi* and *Arabidopsis*.

MYB96, a TF involved in the circadian clock in *Arabidopsis*, was also identified in our sub-network as positively co-expressed with *CCA1* and *RVE8* (Fig. 5). As a key regulator connecting the circadian clock and the environment, *MYB96* is induced by high levels of ABA and can directly bind to the promoter of *TOC1* to active its expression. It is directly regulated by CCA1 through multiple CCA1-binding sites (CBS: AAAATCT) and evening elements (EE: AAATATCT). Interestingly, CCA1 binds to the promoter of MYB96 at dawn, but not at dusk [43, 44]. These findings suggest that our constructed co-expression network is reliable for conserved light-responsive regulator identification.

Several transcription factors with unknown CAM function were identified in the *K. fedtschenkoi* co-expression network. These transcription factors potentially represent novel regulatory mechanisms. As shown in Fig. 5, *WRKY4* was positively co-expressed with *CCA1* and *RVE8*, but negatively co-expressed with *LUX*. Although there is no direct evidence for the involvement of *WRKY4* gene in circadian regulation, its homolog in tomato showed increased transcript abundance at eight hours after dawn, presumptive dusk, and four hours after dusk in comparison with presumptive dawn under long-day conditions [45]. Furthermore, some abscisic acid and light signaling-related genes were identified in our CAM gene-enriched sub-network (e.g., *ZINC FINGER PROTEINS* (*ZFP4* and *ZFP7*), *SIGMA FACTORS* (*SIG1*, *SIG4* and *SIG5*) and B-BOX protein *STH2/BBX21*). In *Arabidopsis*, homologs of *ZFP* are involved in light-responsive pathways, where *ZFP3* can interfere with ABA and light signal in plant development and seed germination [46], while *ZFP1* is expressed in a pathway that is downstream of photomorphogenesis [47]. In prokaryotes, sigma factors are well known for their participation in the control of RNA polymerase activity. The phosphorylation of SIG1 selectively inhibits the expression of gene encoding photosystem I [48]. *SIG1* is strongly induced by red and blue light, but *SIG5* is only induced by blue light under mediating of *CRY1* and *CRY2* [49, 50]. As a key component involved in the COP1-HY5 hub, *STH2/BBX21* is controlled by COP1 through its E3 ubiquitin ligase activity in darkness and promotes photomorphogenesis by activating *HY5* in the light [51]. Our results provide a powerful resource for light-responsive regulator identification.

In addition, the subnetworks of specific genes provide insight on the molecular mechanisms underpinning the different light-induced metabolic phenotypes. For example, the subnetwork of *MDH* (*Kaladp0001s0257*) shows an enrichment of photosynthetic genes (Fig. 7b). The TFs (*AGL16*, *SIGF* and *PIF3*) in this subnetwork might be the dominant regulators of this subnetwork. The ortholog of *AGL16* in *Arabidopsis* is targeted for sequence-specific degradation by *miR824*, expression of a *miR824*-resistant *AGL16* increased the incidence of stomata in high-order complexes in transgenic plants [52]. *SIGF* (*SIG6*) encodes a general sigma factor in chloroplasts. Expression chimeric sigma factor genes in *Arabidopsis sigf* mutant affects the expression of numerous plastid genes [53]. *PIF3* is a key basic helix-loop-helix transcription factor of *Arabidopsis* that negatively regulates light responses, repressing chlorophyll biosynthesis, photosynthesis, and photomorphogenesis in the dark. PIF3 and HDA15 are dissociated from the

target genes upon exposure to red light. PIF3 associates with HDA15 to repress chlorophyll biosynthetic and photosynthetic genes in etiolated seedlings [54].

In the subnetwork of stomatal movement-related gene *ABI2*, 16 known circadian rhythm-related regulators were identified (Fig. 9b), which included synergistically functional *CCA1* and *LHY1*, and their downstream gene *PPR7* [55]. *RVE1* is homologous to *CCA1* and *LHY1*, but inactivation of *RVE1* does not affect circadian rhythmicity, but instead causes a growth phenotype [56]. However, another two *RVE* genes (*RVE2* and *RVE7*) in this subnetwork are directly involved in circadian regulation. RVE2 (CIR1) is possibly part of a regulatory feedback loop that controls a subset of the circadian outputs and modulates the central oscillator [57]. RVE7 (EPR1) is a component of a slave oscillator that contributes to the refinement of output pathways, ultimately mediating the correct oscillatory behavior of target genes [58]. Our dataset provides a additional insights into the photo-responsiveness of CAM plants. In addition, the "gene expression – metabolic phenotypes" correlation analysis identified a series of positively and negatively correlated genes associated with photosynthesis and CAM pathways, which provided molecular clues for understanding the physiological changes which accompany light quality and light intensity responses in *Kalanchoë*.

In conclusion, the comprehensive light-responsive gene expression atlas of *K. fedtschenkoi* provides an extremely useful genomics resource for investigating the molecular mechanisms underlying responses to light quantity and quality in CAM plants. The genome-wide co-expression network lays a solid foundation for discovering novel gene function in CAM plants. Furthermore, the results from our comparative analyses of gene expression and acid accumulation between different light treatments support our hypothesis that both light intensity and light quality can affect the expression of CAM-related genes in *K. fedtschenkoi*.

Methods

Plant Material and Experimental Treatments

Kalanchoë fedtschenkoi (ORNL diploid accession M2) plants originally started from meristem cuttings were grown in soil for 4 weeks in a Percival Model AR-75L2 growth chamber on a 12-h

light (26°C)/12-h dark (18°C) cycle at a photon flux density of 280 μmol m^{-2} s^{-1}. For acclimation prior to light quality or light intensity treatments, plants were placed for at least 2 d in the growth chamber on a 12-h light (26°C)/ 12-h dark (18°C) cycle at a photon flux density of 440 μmol m^{-2} s^{-1}. Light quality treatments consisted of then growing plants under blue light (blue light, 270 μmol m^{-2} s^{-1}) provided by a dark blue gel filter (#119), red light (red light, 280 μmol m^{-2} s^{-1}) provided by primary red gel filter (#106), far-red light (far-red light, 280 μmol m^{-2} s^{-1}) provided by a medium red Roscolux filter (Barndoor Lighting Outfitters, Inc., North Branford, CT), or constant darkness. For all treatments, except constant darkness, a 12-h light (26°C)/12-h dark (18°C) cycle was used. Light intensity treatments consisted of growing the plants under dark growth (dark grown), low light (low light, 150 μmol m^{-2} s^{-1}) or high light (high light, 1,000 μmol m^{-2} s^{-1}) with a 12-h light (26°C) / 12-h dark (18°C) cycle, respectively. All plants used for light quality and light intensity experiments were grown under the indicated conditions for 48 h prior to any tissue collection. All photon flux density measurements described above were taken at leaf level of the apical meristem as these leaves were closest to the light source.

Tissue Collection and RNA Isolation

Fully-expanded leaves (i.e., leaf pair 4-5 counting from the top of the plants) were collected from three biological replicates of plants (each biological replicate was one independent plant) grown under each of the light quality and light intensity experimental conditions. Each sample was collected at both dawn (2 h before the starting of lighting period) and dusk (2 h before the dark period) time points, wrapped in aluminum foil, immediately frozen in liquid nitrogen, and stored at -80°C until processing. For RNA isolation, frozen leaf tissue samples were ground to a fine powder under liquid nitrogen with a mortar and pestle. Isolation of total RNA then proceeded by using the QIAGEN RNeasy® Plant Mini Kit (Cat No. 74904, Qiagen Inc., Valencia, CA, USA) with the following modifications: 600 mg of frozen ground tissue from each sample was mixed thoroughly with 2.57 ml of Fruit Mate™ (TaKaRa Bio USA, Inc., Mountain View, CA). The resulting suspension was centrifuged at 14,000 × *g* at 4°C for 5 min. The supernatant was then mixed with 1.8 ml of QIAGEN buffer RLT/2-mercaptoethanol mix. This solution was centrifuged at 14,000 × *g* at 25°C for 1 min. The supernatant was then mixed with 0.5 volumes of 100% ethanol and remaining steps were performed according to kit instructions. On-column DNase

digestions were performed for all samples according to RNeasy® kit instructions with the QIAGEN RNase-Free DNase Set (Cat No. 79254). Final RNA elution was performed with 50 µl of RNase free water which was run through the column twice. RNA purity and approximate quantity was assessed with a Thermo Scientific™ NanoDrop 2000c spectrophotometer and precise quantity assessed with Quant-iT™ RiboGreen® fluorescence (Thermo Scientific, Rockford, IL). RNA integrity was evaluated on a 1% (w/v) agarose gel using 300 ng RNA.

RNA-Seq Libraries construction and RNA-seq

A total of 42 libraries (7 light conditions × 2 time points × 3 biological replicates) were constructed and performed RNA-seq independently. The total RNA samples were sequenced in the Department of Energy Joint Genome Institute (Walnut Creek, CA). Briefly, the integrity and concentration of the RNA preparations were checked initially using Nano-Drop ND-1000 (Nano-Drop Technologies) and then by BioAnalyzer (Agilent Technologies). RNAs were fragmented into ~300bp sizes and <200bp sizes of libraries (RNA+adapter) were removed by SPRI beads. Plate-based RNA sample prep was performed on the PerkinElmer Sciclone NGS robotic liquid handling system using Illumina's TruSeq Stranded mRNA HT sample prep kit utilizing poly-A selection of mRNA with the following conditions: total RNA starting material was 1 µg per sample and 8 cycles of PCR was used for library amplification. The prepared libraries were then quantified by qPCR using the Kapa SYBR Fast Illumina Library Quantification Kit (Kapa Biosystems) and run on a Roche LightCycler 480 real-time PCR instrument. The quantified libraries were then prepared utilizing a TruSeq paired-end cluster kit, v4, and Illumina's cBot instrument to generate a clustered flowcell for sequencing. Sequencing of the flowcell was performed on the Illumina HiSeq2500 platform using HiSeq TruSeq SBS sequencing kits, v4, following a 2 x 150 indexed run recipe.

Reads mapping and data analysis

After filtering out low-quality reads, RNA-seq reads from each library were aligned to the *Kalanchoë fedtschenkoi* reference genome [13] using GSNAP (v2018-07-04) [59]. FeatureCounts function of Subread (v1.6.1) [60] was used to generate raw gene counts and only reads that mapped uniquely to one locus were counted. Gene expression was estimated as transcripts per million (TPM) [61]. DESeq2 (v1.2.10) [62] was subsequently used to determine which genes were

differentially expressed between pairs of conditions. The parameters used to “call a gene” between conditions was determined at a false discovery rate (FDR) adjusted *P*-value≤0.05. DEGs were classified into hierarchical categories ‘BINs’ using MapMan (version 3.6.0RC1) [63]. Gene Ontology (GO) enrichment analysis was applied to predict gene function and calculate the functional category using BiNGO [64]. Heatmap and bubble plots were generated by the R package ggplot2. All tools were run with default parameters.

Kalanchoë light-responsive eFP browser

TPM-normalized values of the RNA-Seq data sets were uploaded into the *Kalanchoë* eFP browser of the Bio-Analytic Resource (BAR). Representative images of *Kalanchoë* leaf under different light condition were created and an XML file was generated to power a view within the *Kalanchoë* eFP browser at http://bar.utoronto.ca/efp_kalanchoe/cgi-bin/efpWeb.cgi.

Co-expression analysis

For co-expression analysis, the $\log_2$ normalized TPM values of all the samples were used to construct a weighted gene co-expression network using the R package WGCNA [65].

Titratable acidity assays of leaf tissue

For titratable acidity experiments, approximately 0.5 g of fine frozen, ground leaf tissue was added to 10.0 ml of 50% (v/v) methanol and mixed well. This suspension was then boiled at 80°C for 10 min. Additional 50% (v/v) methanol was added after boiling to any samples that showed volume loss. The boiled samples were then centrifuged at 2,000 × *g* for 10 min at room temperature. Supernatants were titrated with 10 mM KOH to pH = 7.0 and 8.4 corresponding to malate and citrate, respectively. Leaf titratable acidities were expressed as μmol H^+ g^{-1} fresh weight.

Carbohydrate analysis of leaf tissue

For soluble sugar and starch assays, ground leaf tissue was boiled in 10.0 ml of 50% (v/v) methanol at 80°C for 30 minutes. Sample volumes were adjusted back to original volumes with 50% (v/v) methanol. Samples were then centrifuged at 2,000 × *g* for 10 min at room temperature. The resulting supernatants were reserved for soluble sugar analysis. For starch extraction, tissue pellets were washed twice with 10 ml of Nanopure water with centrifugation at 2,000 × *g* after each wash. 1.2 ml of acetate buffer (prepared from 86 ml of 0.1 M sodium acetate and 114 ml of

0.1 N acetic acid, pH 4.5) was added to the washed pellet and resuspended by vortexing. Then, 0.2 ml of starch digestion solution (300 units α-amyloglucosidase and 25 units α-amylase prepared in 20 ml acetate buffer) was added and the mixture incubated overnight at 45°C. Starch-digested samples were centrifuged at 2,000 × *g* for 10 min at room temperature and the supernatant used for starch analysis. Colorimetric assays for determination of starch and soluble sugar content were performed as described [66].

Availability of supporting data

All raw short reads are available in the NCBI SRA database (SRA accessions: SRP146136, SRP146139, SRP146175, SRP146204, SRP146205, SRP146213, SRP148019 – SRP148030, SRP148037 – SRP148060) (Supplementary Table S1).

Abbreviations

BAR: Bio-Analytic Resource; CAM: Crassulacean acid metabolism; CBS: CCA1 binding sites; DEG: differentially expressed gene; EE: evening elements; FDR: false discovery rate; GO: gene ontology; MDS: multidimensional scaling; PC: principal component; PCC: Pearson correlation coefficient; PPFD: photosynthetic photon flux density; RNA-Seq: RNA-Sequencing; Rubisco: ribulose-1,5-bisphosphate carboxylase/oxygenase; TF: transcription factor; TPM: transcripts per million; WGCNA: weighted gene co-expression network analysis; WUE: water-use efficiency.

Competing interests

The authors declare that they have no competing interests.

Funding

This research was supported by the U.S. Department of Energy, Office of Science, Genomic Science Program under Award Number DE-SC0008834. Additional support was provided by the

Community Science Program (project 503025) at the Department of Energy Joint Genome Institute and the DOE Center for Bioenergy Innovation at the Oak Ridge National Laboratory.

Author contributions

X.Y., J.Z. and R.H. conceived and designed the research. R.H., T.G., P.Y., and J.C.C. performed the experiments. J.Z., A.S., A.L., M.W., D.L., V.N., J.S. and A.M.B. analyzed the data. J.Z., A.P. and N.J.P. provided eFP browser instance. J.Z. and R.H. drafted the manuscript. X.Y., J.G.C., W.M., J.C.C., and G.A.T. revised the manuscript. All authors read and approved the manuscript.

Acknowledgements

This manuscript has been authored by UT-Battelle, LLC under Contract No. DE-AC05-00OR22725 with the U.S. Department of Energy. The work conducted by the U.S. Department of Energy Joint Genome Institute is supported by the Office of Science of the U.S. Department of Energy under Contract No. DE-AC02-05CH11231. This research used resources of the Compute and Data Environment for Science (CADES) and the Oak Ridge Leadership Computing Facility at the Oak Ridge National Laboratory.

References

1. Mølmann JA, Junttila O, Johnsen Ø and Olsen JE. Effects of red, far- red and blue light in maintaining growth in latitudinal populations of Norway spruce (Picea abies). Plant, Cell & Environment. 2006;29 2:166-72.
2. Quail PH. Phytochrome photosensory signalling networks. Nature Reviews Molecular Cell Biology. 2002;3 2:85.
3. OuYang F, Mao J-F, Wang J, Zhang S and Li Y. Transcriptome analysis reveals that red and blue light regulate growth and phytohormone metabolism in Norway spruce [Picea abies (L.) Karst.]. PloS One. 2015;10 8:e0127896.
4. Briggs WR and Olney MA. Photoreceptors in plant photomorphogenesis to date. Five phytochromes, two cryptochromes, one phototropin, and one superchrome. Plant Physiology. 2001;125 1:85-8.
5. Lin C and Shalitin D. Cryptochrome structure and signal transduction. Annual Review of Plant Biology. 2003;54 1:469-96.
6. Briggs W, Beck C, Cashmore A, Christie J, Hughes J, Jarillo J, et al. The phototropin family of photoreceptors. Plant Cell. 2001;13 5:993-7.
7. Nagy F and Schäfer E. Phytochromes control photomorphogenesis by differentially regulated, interacting signaling pathways in higher plants. Annual Review of Plant Biology. 2002;53 1:329-55.
8. Fan X-X, Xu Z-G, Liu X-Y, Tang C-M, Wang L-W and Han X-l. Effects of light intensity on the growth and leaf development of young tomato plants grown under a combination of red and blue light. Scientia Horticulturae. 2013;153:50-5.
9. Rossel JB, Wilson IW and Pogson BJ. Global changes in gene expression in response to high light in *Arabidopsis*. Plant Physiology. 2002;130 3:1109-20.
10. Zavala J and Ravetta D. Allocation of photoassimilates to biomass, resin and carbohydrates in *Grindelia chiloensis* as affected by light intensity. Field Crops Research. 2001;69 2:143-9.
11. Yang X, Cushman JC, Borland AM, Edwards EJ, Wullschleger SD, Tuskan GA, et al. A roadmap for research on crassulacean acid metabolism (CAM) to enhance sustainable food and bioenergy production in a hotter, drier world. New Phytologist. 2015;207 3:491-504.
12. Borland AM, Hartwell J, Weston DJ, Schlauch KA, Tschaplinski TJ, Tuskan GA, et al. Engineering crassulacean acid metabolism to improve water-use efficiency. Trends in Plant Science. 2014;19 5:327-38.
13. Yang X, Hu R, Yin H, Jenkins J, Shu S, Tang H, et al. The *Kalanchoë* genome provides insights into convergent evolution and building blocks of crassulacean acid metabolism. Nature Communications. 2017;8 1:1899.
14. Borland AM, Wullschleger SD, Weston DJ, Hartwell J, Tuskan GA, Yang X, et al. Climate-resilient agroforestry: physiological responses to climate change and engineering of crassulacean acid metabolism (CAM) as a mitigation strategy. Plant, Cell & Environment. 2015;38 9:1833-49.
15. Hartwell J, Dever LV and Boxall SF. Emerging model systems for functional genomics analysis of Crassulacean acid metabolism. Current Opinion in Plant Biology. 2016;31:100-8.
16. Dodd AN, Borland AM, Haslam RP, Griffiths H and Maxwell K. Crassulacean acid metabolism: plastic, fantastic. Journal of experimental botany. 2002;53 369:569-80.
17. Grams TE and Thiel S. High light- induced switch from C 3- photosynthesis to Crassulacean acid metabolism is mediated by UV- A/blue light. Journal of Experimental Botany. 2002;53 373:1475-83.
18. Ceusters J, Borland AM, Godts C, Londers E, Croonenborghs S, Van Goethem D, et al. Crassulacean acid metabolism under severe light limitation: a matter of plasticity in the shadows? Journal of Experimental Botany. 2010;62 1:283-91.

19. Ceusters J, Borland AM, Taybi T, Frans M, Godts C and De Proft MP. Light quality modulates metabolic synchronization over the diel phases of crassulacean acid metabolism. Journal of Experimental Botany. 2014;65 13:3705-14.
20. Kornas A, Fischer-Schliebs E, Lüttge U and Miszalski Z. Adaptation of the obligate CAM plant *Clusia alata* to light stress: metabolic responses. Journal of Plant Physiology. 2009;166 17:1914-22.
21. Miszalski Z, Kornas A, Rozpądek P, Fischer-Schliebs E and Lüttge U. Independent fluctuations of malate and citrate in the CAM species *Clusia hilariana* Schltdl. under low light and high light in relation to photoprotection. Journal of Plant Physiology. 2013;170 5:453-8.
22. Klepikova AV, Kasianov AS, Gerasimov ES, Logacheva MD and Penin AA. A high resolution map of the Arabidopsis thaliana developmental transcriptome based on RNA-seq profiling. Plant Journal. 2016;88 6:1058-70. doi:doi:10.1111/tpj.13312.
23. Benedito VA, Torres-Jerez I, Murray JD, Andriankaja A, Allen S, Kakar K, et al. A gene expression atlas of the model legume *Medicago truncatula*. Plant Journal. 2008;55 3:504-13. doi:doi:10.1111/j.1365-313X.2008.03519.x.
24. Matas AJ, Yeats TH, Buda GJ, Zheng Y, Chatterjee S, Tohge T, et al. Tissue- and cell-type specific transcriptome profiling of expanding tomato fruit provides insights into metabolic and regulatory specialization and cuticle formation. Plant Cell. 2011;23 11:3893-910. doi:10.1105/tpc.111.091173 %J The Plant Cell.
25. Ramírez-González RH, Borrill P, Lang D, Harrington SA, Brinton J, Venturini L, et al. The transcriptional landscape of polyploid wheat. Science. 2018;361 6403:eaar6089. doi:10.1126/science.aar6089 %J Science.
26. Sibout R, Proost S, Hansen BO, Vaid N, Giorgi FM, Ho-Yue-Kuang S, et al. Expression atlas and comparative coexpression network analyses reveal important genes involved in the formation of lignified cell wall in *Brachypodium distachyon*. New Phytologist. 2017;215 3:1009-25. doi:10.1111/nph.14635.
27. Li Q and Kubota C. Effects of supplemental light quality on growth and phytochemicals of baby leaf lettuce. Environmental and Experimental Botany. 2009;67 1:59-64.
28. Fukuda N, Fujita M, Ohta Y, Sase S, Nishimura S and Ezura H. Directional blue light irradiation triggers epidermal cell elongation of abaxial side resulting in inhibition of leaf epinasty in geranium under red light condition. Scientia Horticulturae. 2008;115 2:176-82.
29. Kitazaki K, Fukushima A, Nakabayashi R, Okazaki Y, Kobayashi M, Mori T, et al. Metabolic reprogramming in leaf lettuce grown under different light quality and intensity conditions using narrow-band LEDs. Scientific Reports. 2018;8 1:7914.
30. Li C-X, Xu Z-G, Dong R-Q, Chang S-X, Wang L-Z, Khalil-Ur-Rehman M, et al. An RNA-seq analysis of grape plantlets grown in vitro reveals different responses to blue, green, red LED light, and white fluorescent light. Frontiers in Plant Science. 2017;8:78.
31. Tardu M, Dikbas UM, Baris I, Kavakli IHJF and genomics i. RNA-seq analysis of the transcriptional response to blue and red light in the extremophilic red alga, *Cyanidioschyzon merolae*. Functional & Integrative Genomics. 2016;16 6:657-69.
32. Hao X, Li L, Hu Y, Zhou C, Wang X, Wang L, et al. Transcriptomic analysis of the effects of three different light treatments on the biosynthesis of characteristic compounds in the tea plant by RNA-Seq. Tree Genetics & Genomes. 2016;12 6:118.
33. Sellaro R, Hoecker U, Yanovsky M, Chory J and Casal JJ. Synergism of red and blue light in the control of Arabidopsis gene expression and development. Current Biology. 2009;19 14:1216-20.
34. Pazhamala LT, Purohit S, Saxena RK, Garg V, Krishnamurthy L, Verdier J, et al. Gene expression atlas of pigeonpea and its application to gain insights into genes associated with pollen fertility implicated in seed formation. Journal of Experimental Botany. 2017;68 8:2037-54.
35. Kudapa H, Garg V, Chitikineni A and Varshney RK. The RNA-Seq-based high resolution gene expression atlas of chickpea (*Cicer arietinum* L.) reveals dynamic spatio-temporal changes

associated with growth and development. Plant, Cell & Environment. 2018;41 9:2209-25. doi:doi:10.1111/pce.13210.

36. Kurepin LV, Emery RN, Pharis RP and Reid DM. The interaction of light quality and irradiance with gibberellins, cytokinins and auxin in regulating growth of *Helianthus annuus* hypocotyls. Plant, Cell & Environment. 2007;30 2:147-55.

37. Zhang Z, Ji R, Li H, Zhao T, Liu J, Lin C, et al. CONSTANS-LIKE 7 (COL7) is involved in phytochrome B (phyB)-mediated light-quality regulation of auxin homeostasis. Molecular Plant. 2014;7 9:1429-40.

38. Gubler F, Hughes T, Waterhouse P and Jacobsen J. Regulation of dormancy in barley by blue light and after-ripening: effects on abscisic acid and gibberellin metabolism. Plant Physiology. 2008;147 2:886-96.

39. Nusinow DA, Helfer A, Hamilton EE, King JJ, Imaizumi T, Schultz TF, et al. The ELF4-ELF3-LUX complex links the circadian clock to diurnal control of hypocotyl growth. Nature. 2011;475 7356:398-402. doi:10.1038/nature10182.

40. Lu SX, Knowles SM, Andronis C, Ong MS and Tobin EM. CIRCADIAN CLOCK ASSOCIATED1 and LATE ELONGATED HYPOCOTYL function synergistically in the circadian clock of *Arabidopsis*. Plant Physiology. 2009;150 2:834-43. doi:10.1104/pp.108.133272.

41. Kikis EA, Khanna R and Quail PH. ELF4 is a phytochrome-regulated component of a negative-feedback loop involving the central oscillator components CCA1 and LHY. Plant Journal. 2005;44 2:300-13. doi:10.1111/j.1365-313X.2005.02531.x.

42. Hazen SP, Schultz TF, Pruneda-Paz JL, Borevitz JO, Ecker JR and Kay SA. LUX ARRHYTHMO encodes a Myb domain protein essential for circadian rhythms. Proc Natl Acad Sci U S A. 2005;102 29:10387-92. doi:10.1073/pnas.0503029102.

43. Lee HG, Mas P and Seo PJ. MYB96 shapes the circadian gating of ABA signaling in *Arabidopsis*. Scientific Reports. 2016;6:17754. doi:10.1038/srep17754.

44. Muchapirei CI, Valentine S-L and Roden LC. Plant circadian networks and responses to the environment. Functional Plant Biology. 2018;45 4:393-9.

45. Facella P, Lopez L, Carbone F, Galbraith DW, Giuliano G and Perrotta G. Diurnal and Circadian Rhythms in the Tomato Transcriptome and Their Modulation by Cryptochrome Photoreceptors. PLOS ONE. 2008;3 7:e2798. doi:10.1371/journal.pone.0002798.

46. Joseph MP, Papdi C, Kozma-Bognar L, Nagy I, Lopez-Carbonell M, Rigo G, et al. The *Arabidopsis* ZINC FINGER PROTEIN3 interferes with abscisic acid and light signaling in seed germination and plant development. Plant Physiology. 2014;165 3:1203-20. doi:10.1104/pp.113.234294.

47. Chrispeels HE, Oettinger H, Janvier N and Tague BW. *AtZFP1*, encoding *Arabidopsis thaliana* C2H2 zinc-finger protein 1, is expressed downstream of photomorphogenic activation. Plant Molecular Biology. 2000;42 2:279-90.

48. Shimizu M, Kato H, Ogawa T, Kurachi A, Nakagawa Y and Kobayashi H. Sigma factor phosphorylation in the photosynthetic control of photosystem stoichiometry. Proc Natl Acad Sci U S A. 2010;107 23:10760-4. doi:10.1073/pnas.0911692107.

49. Tsunoyama Y, Morikawa K, Shiina T and Toyoshima Y. Blue light specific and differential expression of a plastid σ factor, Sig5 in *Arabidopsis thaliana*. FEBS Letters. 2002;516 1:225-8. doi:10.1016/S0014-5793(02)02538-3.

50. Onda Y, Yagi Y, Saito Y, Takenaka N and Toyoshima Y. Light induction of *Arabidopsis SIG1* and *SIG5* transcripts in mature leaves: differential roles of cryptochrome 1 and cryptochrome 2 and dual function of SIG5 in the recognition of plastid promoters. Plant Journal. 2008;55 6:968-78. doi:10.1111/j.1365-313X.2008.03567.x.

51. Xu D, Jiang Y, Li J, Lin F, Holm M and Deng XW. BBX21, an *Arabidopsis* B-box protein, directly activates HY5 and is targeted by COP1 for 26S proteasome-mediated degradation. Proceedings of the National Academy of Sciences of the United States of America. 2016;113 27:7655-60. doi:10.1073/pnas.1607687113.

52. Kutter C, Schob H, Stadler M, Meins F, Jr. and Si-Ammour A. MicroRNA-mediated regulation of stomatal development in Arabidopsis. Plant Cell. 2007;19 8:2417-29. doi:10.1105/tpc.107.050377.
53. Schweer J, Geimer S, Meurer J and Link G. Arabidopsis mutants carrying chimeric sigma factor genes reveal regulatory determinants for plastid gene expression. Plant Cell Physiol. 2009;50 7:1382-6. doi:10.1093/pcp/pcp069.
54. Liu X, Chen CY, Wang KC, Luo M, Tai R, Yuan L, et al. PHYTOCHROME INTERACTING FACTOR3 associates with the histone deacetylase HDA15 in repression of chlorophyll biosynthesis and photosynthesis in etiolated Arabidopsis seedlings. Plant Cell. 2013;25 4:1258-73. doi:10.1105/tpc.113.109710.
55. Nagel DH, Doherty CJ, Pruneda-Paz JL, Schmitz RJ, Ecker JR and Kay SA. Genome-wide identification of CCA1 targets uncovers an expanded clock network in Arabidopsis. Proc Natl Acad Sci U S A. 2015;112 34:E4802-10. doi:10.1073/pnas.1513609112.
56. Rawat R, Schwartz J, Jones MA, Sairanen I, Cheng Y, Andersson CR, et al. REVEILLE1, a Myb-like transcription factor, integrates the circadian clock and auxin pathways. Proc Natl Acad Sci U S A. 2009;106 39:16883-8. doi:10.1073/pnas.0813035106.
57. Zhang X, Chen Y, Wang ZY, Chen Z, Gu H and Qu LJ. Constitutive expression of CIR1 (RVE2) affects several circadian- regulated processes and seed germination in Arabidopsis. The Plant Journal. 2007;51 3:512-25.
58. Kuno N, Moller SG, Shinomura T, Xu XM, Chua NH and Furuya M. The novel MYB protein EARLY-PHYTOCHROME-RESPONSIVE1 is a component of a slave circadian oscillator in Arabidopsis. Plant Cell. 2003;15 10:2476-88. doi:DOI 10.1105/tpc.014217.
59. Wu TD and Nacu S. Fast and SNP-tolerant detection of complex variants and splicing in short reads. Bioinformatics. 2010;26 7:873-81.
60. Liao Y, Smyth GK and Shi W. featureCounts: an efficient general purpose program for assigning sequence reads to genomic features. Bioinformatics. 2013;30 7:923-30.
61. Li B and Dewey CN. RSEM: accurate transcript quantification from RNA-Seq data with or without a reference genome. BMC Bioinformatics. 2011;12 1:323.
62. Love MI, Huber W and Anders S. Moderated estimation of fold change and dispersion for RNA-seq data with DESeq2. Genome Biology. 2014;15 12:550.
63. Thimm O, Bläsing O, Gibon Y, Nagel A, Meyer S, Krüger P, et al. MAPMAN: a user- driven tool to display genomics data sets onto diagrams of metabolic pathways and other biological processes. The Plant Journal. 2004;37 6:914-39.
64. Maere S, Heymans K and Kuiper M. BiNGO: a Cytoscape plugin to assess overrepresentation of gene ontology categories in biological networks. Bioinformatics. 2005;21 16:3448-9.
65. Langfelder P and Horvath S. WGCNA: an R package for weighted correlation network analysis. BMC Bioinformatics. 2008;9 1:559.
66. Dubois M, Gilles KA, Hamilton JK, Rebers Pt and Smith F. Colorimetric method for determination of sugars and related substances. Analytical chemistry. 1956;28 3:350-6.

Figure Legends

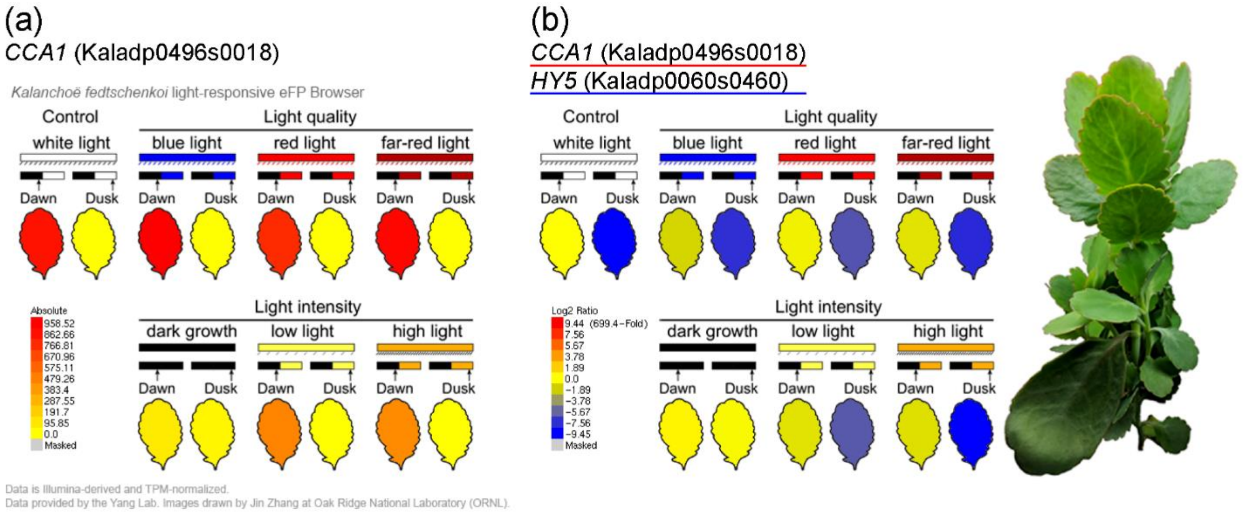

Fig. 1. *Kalanchoë* light-responsive eFP browser.

(a) View of the eFP browser including the RNA-Seq data set described in this study. Expression values in the samples are indicated by a color gradient, where yellow indicates low expression and red indicates high expression. The legend describing the color gradient and expression values is shown in the bottom left corner. *CCA1* gene Kaladp0496s0018 is used as an example. The leaf samples were collected at dawn (i.e., 2 h before the beginning of light period) and dusk (i.e., 2 h before the beginning of dark period) under control condition (WL, white light) and various light quality conditions (BL, blue light; RL, red light; and FRL, far-red light) and light intensity conditions (DG, dark grown; LL, low-light intensity; and HL, high-light intensity).

(b) *Kalanchoë* genes *CCA1* (Kaladp0496s0018) and *HY5* (Kaladp0060s0460) displayed in a comparative view of the expression level extracted from the eFP browser. The legend describing the color gradient and log_2 ratio is shown in the bottom left corner.

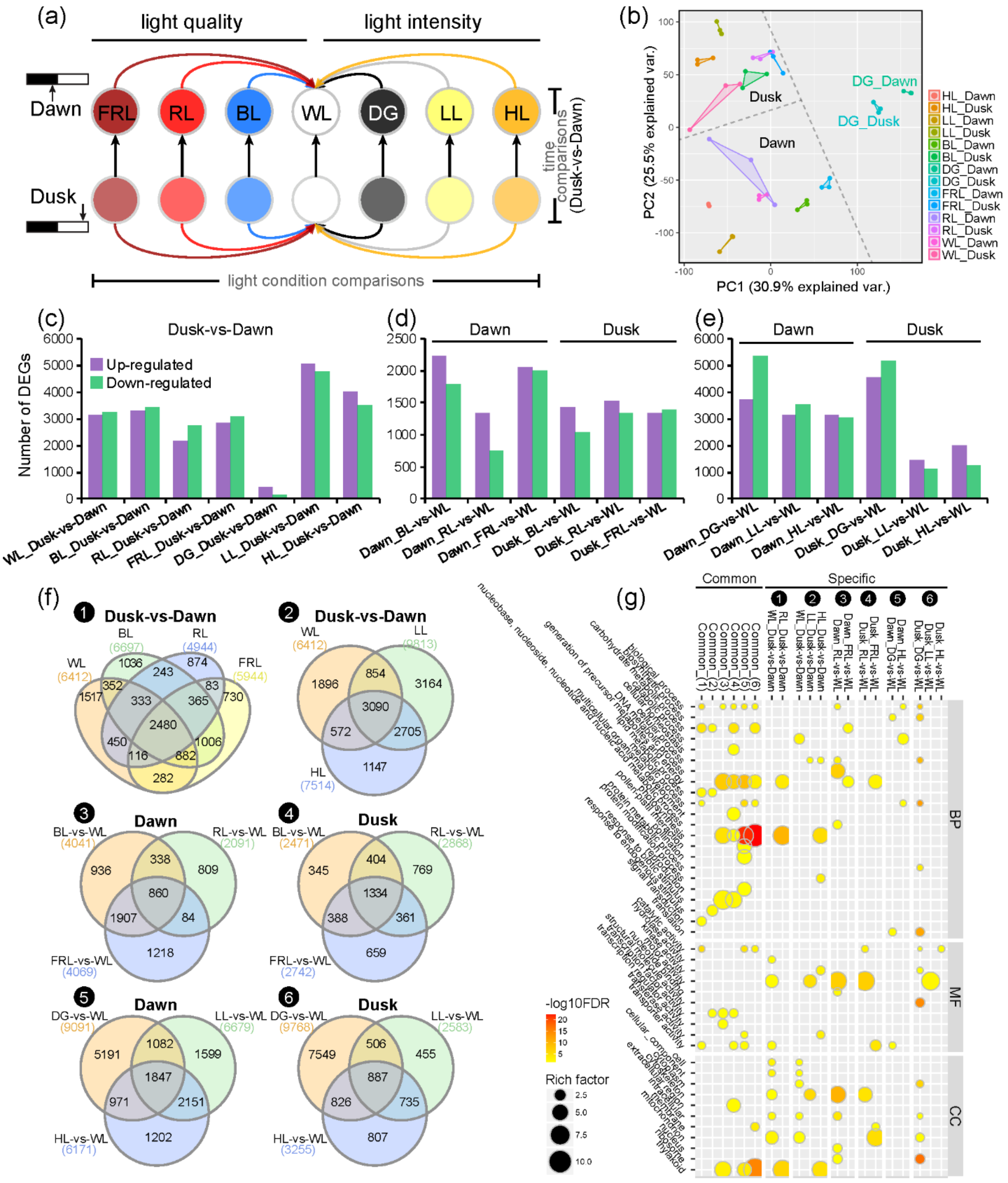

Fig. 2. Transcriptomic comparison of *Kalanchoë fedtschenkoi* under various light quality and light intensity conditions.

(a) Schematic of sample collection and comparisons. The leaf samples were collected at dawn (2-hour before light period) and dusk (2-hour before dark period) under control condition (WL, white light) and various light quality conditions (BL, blue light; RL, red light; and FRL, far-red light) and light intensity conditions (DG, dark grown; LL, low-light intensity; and HL, high-light intensity). For differentially expressed genes (DEGs) identification, the comparisons were classified into time comparisons (Dusk-vs-Dawn) and light condition comparisons (BL/RL/FRL-vs-WL for light quality comparisons and DG/LL/HL-vs-WL for light intensity comparisons).

(b) Principal component analysis (PCA) of the 14 groups of transcriptome data.

(c-e) Statistic of DEGs between dawn and dusk in time comparisons **(c)** and among various light quality **(d)** or light intensity **(e)** in light condition comparisons.

(f) Venn diagrams represent DEGs overlapped in different comparisons. ① dusk-vs-dawn under different light quality; ② dusk-vs-dawn under different light intensity; ③ different light quality (BL/RL/FRL-vs-WL) at dawn; ④ different light quality at dusk; ⑤ different light intensity (DG/LL/HL-vs-WL) at dawn; ⑥ different light intensity at dusk.

(g) Gene ontology (GO) enrichment of DEGs shared by different comparisons in (f) Venn diagrams (Common) or specific DEGs in each Venn diagram. BP, biological process; MF, molecular function; and CC, cellular component. GOslim terms were shown in here.

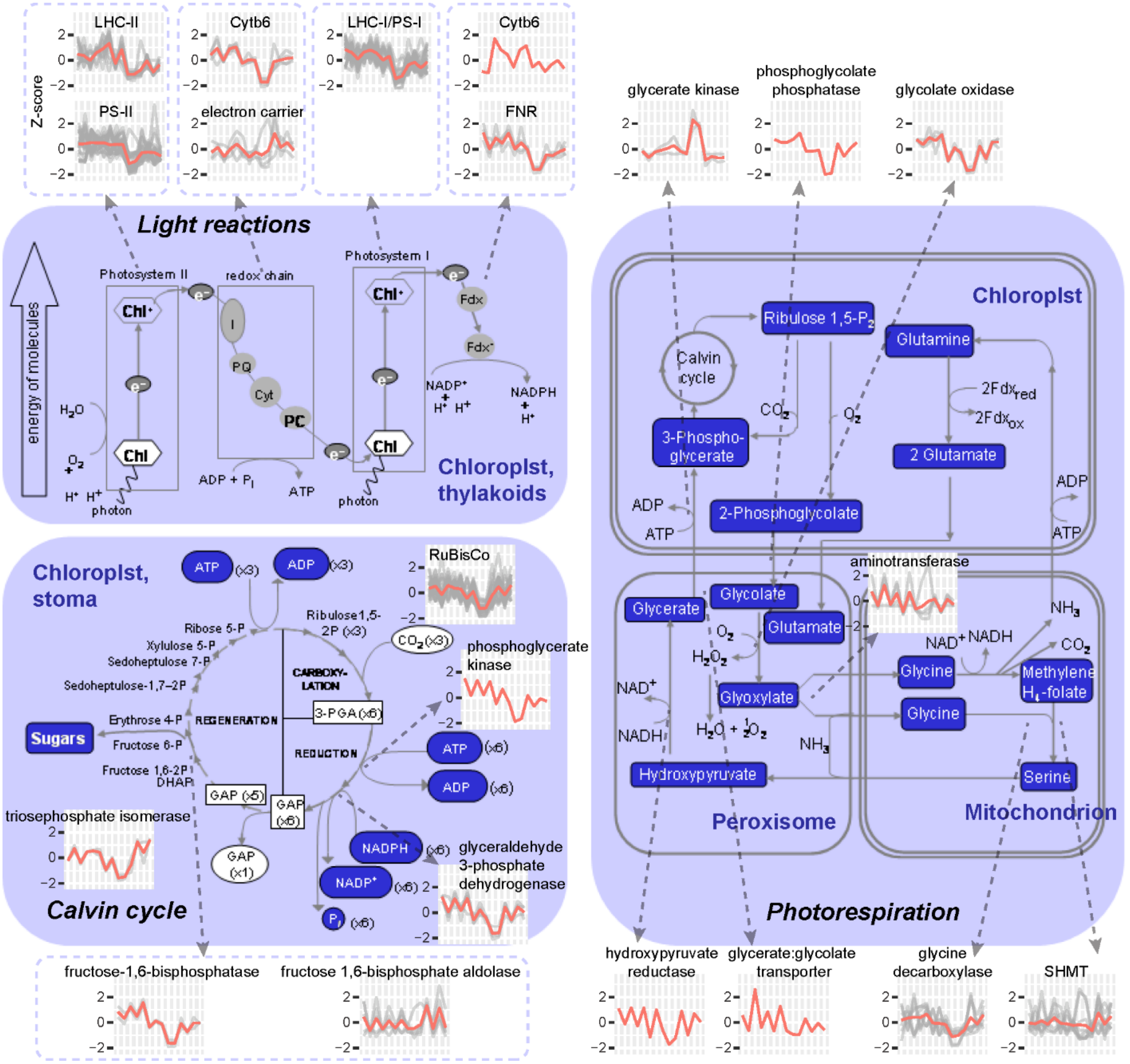

Fig. 3. Schematic representation of gene expression patterns in the photosynthesis pathway.

The figure was modified based on the MapMan visualization platform. Line plots represent Z-score normalized (*y*-axis) expression patterns of DEGs under various light quality and light intensity conditions. The *x*-axis from left to right represents the samples of WL_Dawn, WL_Dusk, BL_Dawn, BL_Dusk, RL_Dawn, RL_Dusk, FRL_Dawn, FRL_Dusk, DG_Dawn, DG_Dusk, LL_Dawn, LL_Dusk, HL_Dawn and HL_Dusk. Detailed gene list and expression data were shown in Supplementary Table S4.

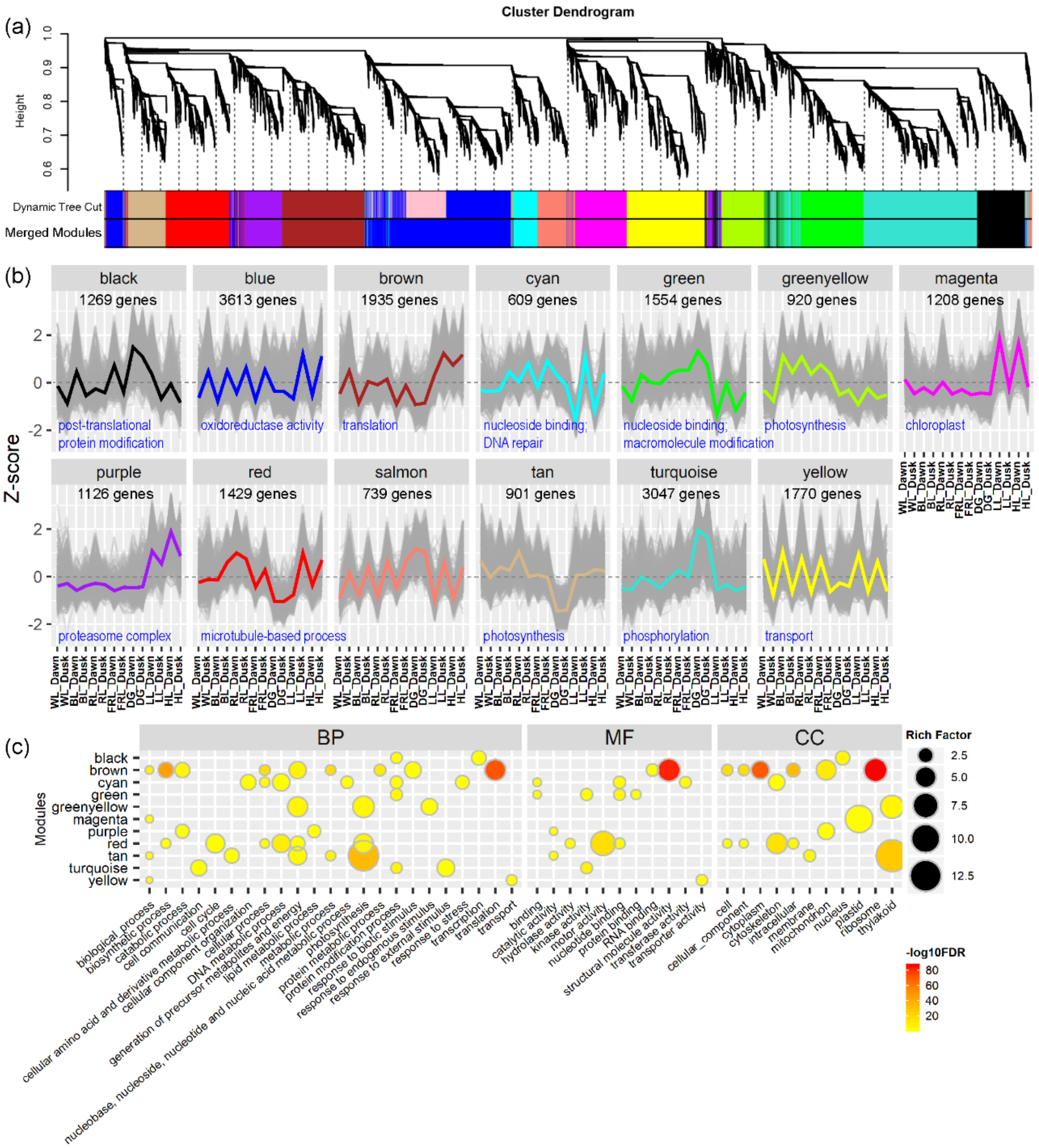

Fig. 4. Weighted Gene Co-expression Network Analysis (WGCNA) of DEGs in *Kalanchoë fedtschenkoi* under various light quality and light intensity conditions.

(a) Cluster dendrogram of DEGs in *Kalanchoë fedtschenkoi* under various light quality and light intensity conditions. Different colors in merged modules column represent 13 different modules (MEs). **(b)** Z-score normalized expression patterns of DEGs in different modules. **(c)** GO enrichment analysis of DEGs in different MEs. Node color represents -$\log_{10}$ transformed FDR

corrected *P* value. Node size represents rich factor. Full list of enriched GO terms was shown in Supplementary Table S5.

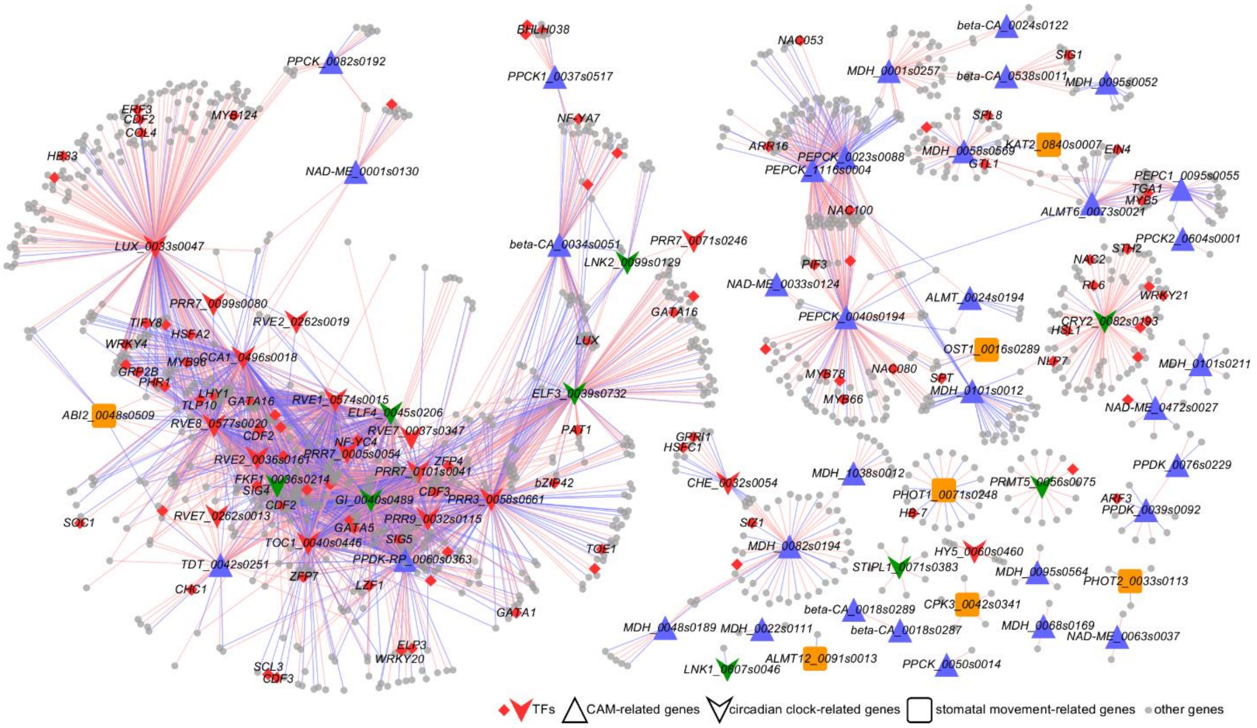

Fig. 5. Sub-network of CAM, circadian clock and stomatal movement.

Red nodes represent transcription factors (TFs). Triangle, arrowhead and rounded-rectangle shapes of nodes represent CAM-, circadian clock- and stomatal movement-related genes, respectively. Red and blue edges represent positive correlation (PCC>0.95 and $p \leq 0.01$) and negative correlation (PCC<-0.95 and $p \leq 0.01$), respectively.

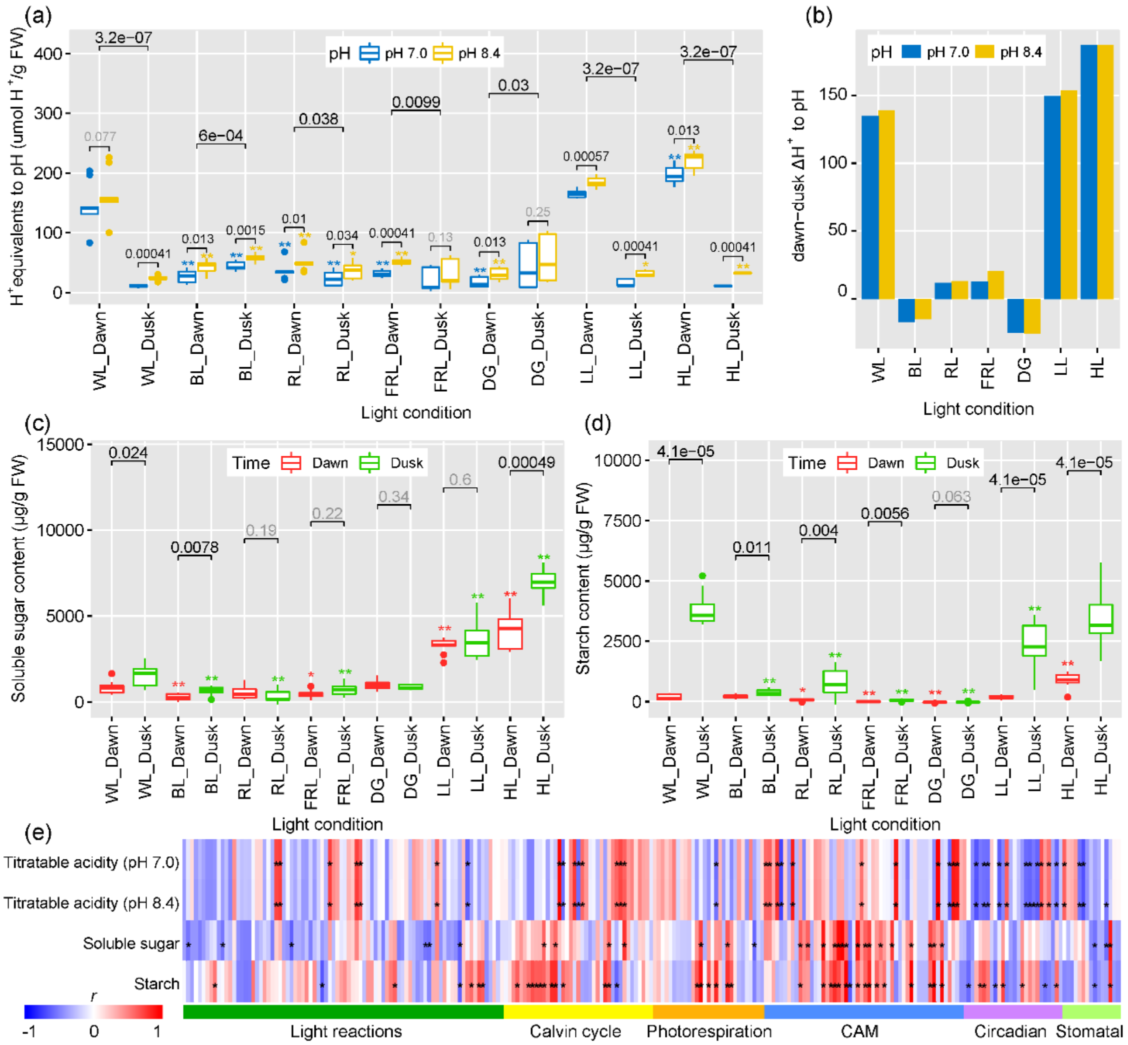

Fig. 6. Physiological changes of *Kalanchoë fedtschenkoi* under various light quality and light intensity conditions.

(a) Nocturnal malic and citric acid accumulation was assessed by the difference in H^+ concentration between dawn and dusk samples under different light conditions (WL, white light; BL, blue light; RL, red light; FRL, far-red light; DG, darkness; LL, low-light; HL, high-light). **(b)** Dawn-dusk ΔH^+ values under different light conditions. **(c)** Soluble sugar contents under different light conditions. **(d)** Starch accumulation under different light conditions. Embedded *P* values indicate statistical differences between dawn and dusk in (a), (c) and (d) and differences between two pH treatments (a); while asterisks (*, $P < 0.05$; **, $P < 0.01$) indicate significant differences between light quality/intensity treatments and WL control at dawn or dusk. **(e)** Correlation of

physiological parameters and gene expression. asterisks (*) indicate significant correlation (P < 0.05).

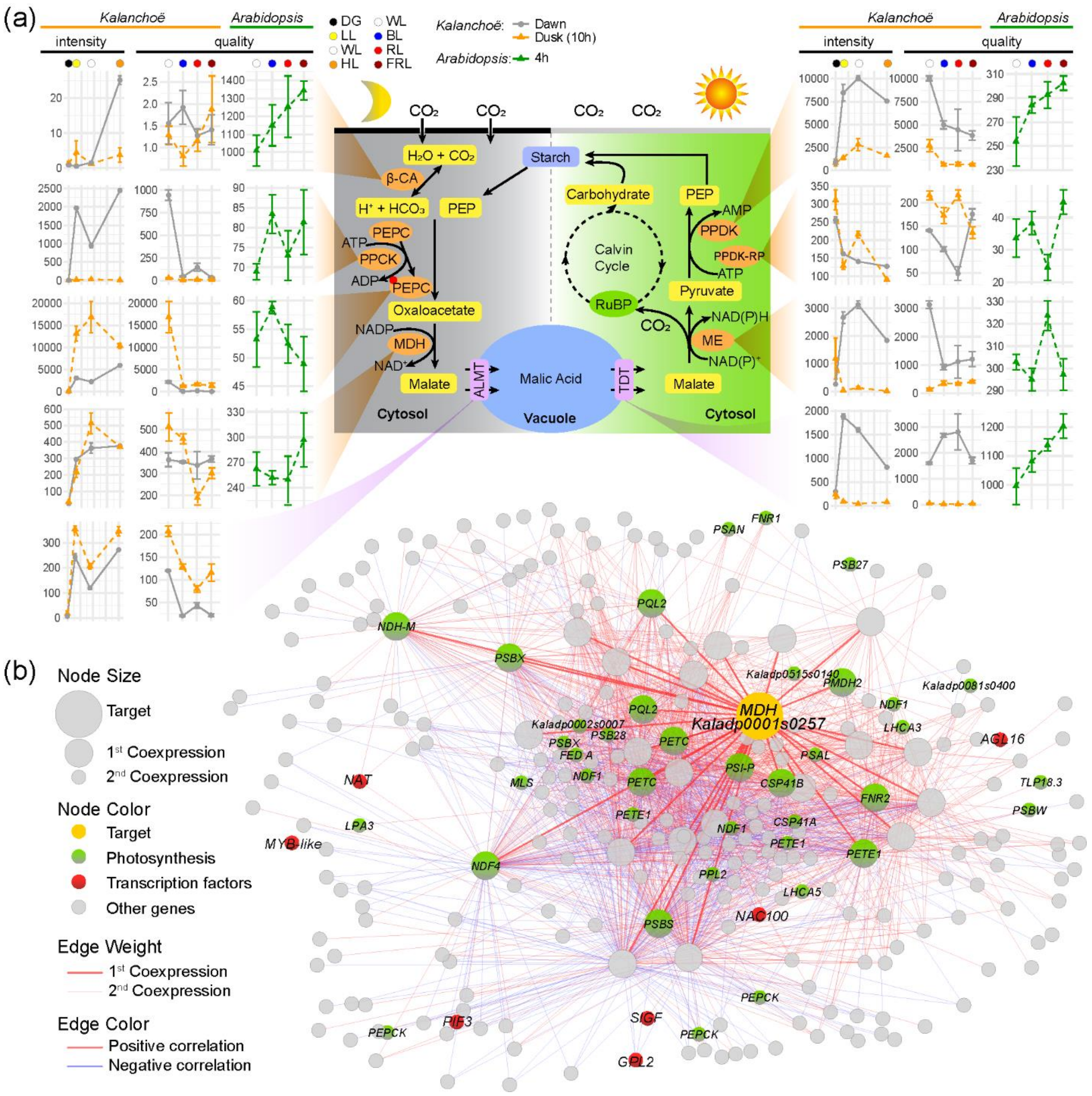

Fig. 7. Expression profiles of genes involved in CAM pathway.

(a) Expression pattern of CAM-related genes under different light intensity (DG, 0 μmol m^{-2} s^{-1}; LL, 150 μmol m^{-2} s^{-1}; WL, 440 μmol m^{-2} s^{-1}; HL, 1000 μmol m^{-2} s^{-1}) and under different light quality (WL, BL, RL and FRL). The CAM pathway was modified from Yang et al. (2017). Gene name (*Kalanchoë* gene ID, *Arabidopsis* gene ID): *β-CA* (Kaladp0018s0287, AT5G14740), *PPCK1* (Kaladp0037s0517, AT3G04530), *PEPC1* (Kaladp0095s0055, AT3G14940), *MDH2* (Kaladp0001s0257, AT5G09660), *ALMT6* (Kaladp0073s0021, AT1G25480 – missed expression

data), *PPDK* (Kaladp0076s0229, AT4G15530), *PPDK-RP* (Kaladp0010s0106, AT4G21210), *NADP-ME* (Kaladp0092s0166, AT1G79750), *TDT* (Kaladp0042s0251, AT5G47560). The expression of *Kalanchoë* genes was detected at dawn (2 h before the starting of lighting period) and dusk (2 h before the dark period, i.e. 10 h after light treatments). The expression data of *Arabidopsis* responsive to light quality (WL, BL, RL and FRL) was obtained from *Arabidopsis* eFP browser (light series). *Arabidopsis* seeds were plated on 1.2% MS agar plats and stratified at 8°C for 48 h in the dark. Germination was induced with 2 h red light, followed by growth for 94 h in complete darkness at 22°C. Plants were then irradiated at different light conditions. Samples (mainly hypocotyl and cotyledons) were collected at 4 h after treatments. **(b)** Subnetwork of *MDH2* (Kaladp0001s0257). Orange nodes represent center of the subnetworks; red and green nodes represent TFs and photosynthesis-related genes, respectively. Large and small nodes represent the 1st and 2nd co-expressed genes, respectively. Thick and thin edges indicate the 1st and 2nd co-expression relationships, respectively. Red and blue edges indicate the positive and negative correlation, respectively.

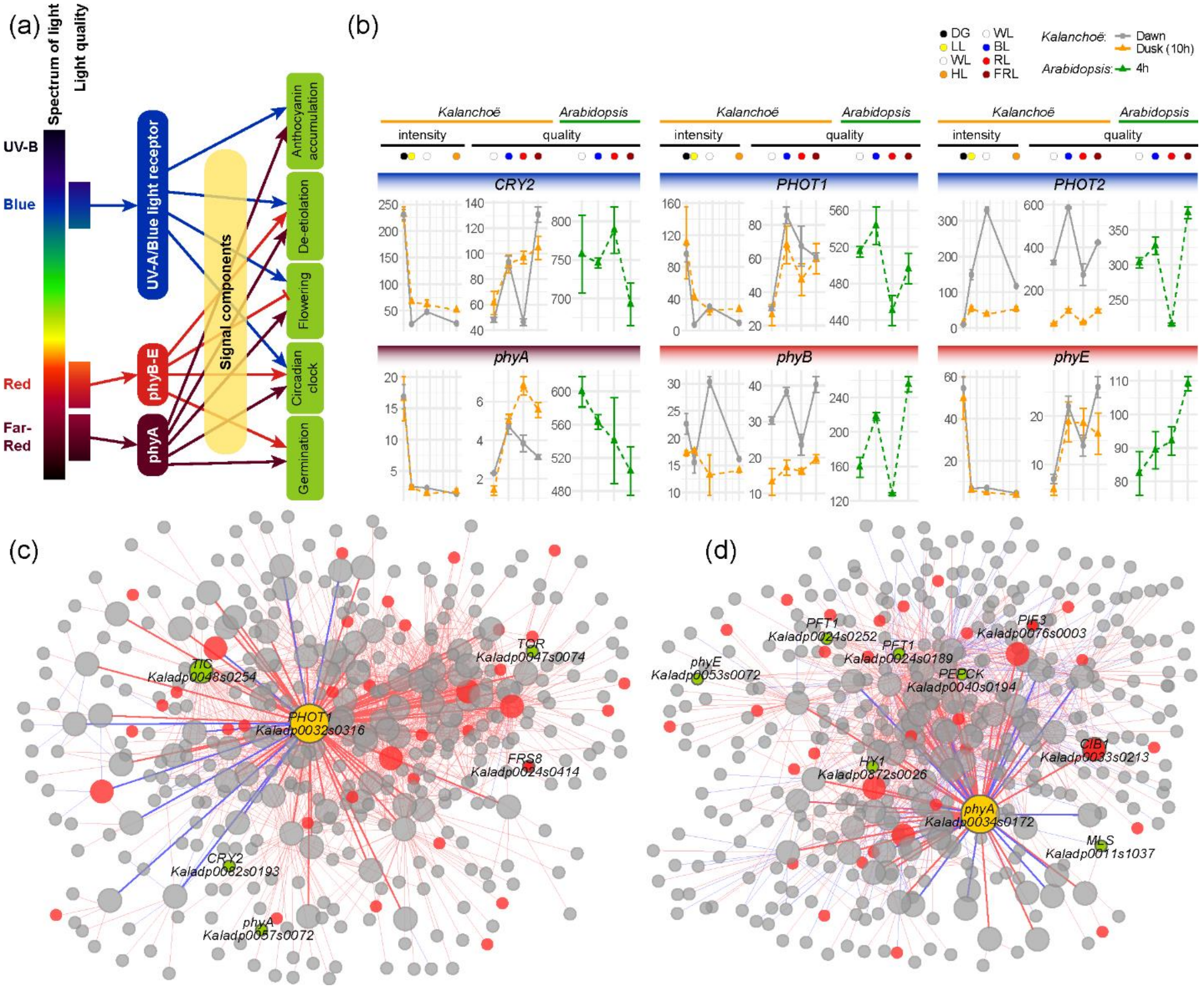

Fig. 8. Expression patterns of photoreceptors in *Kalanchoë* leaf.

(a) Light signaling model from photoreceptor to photo-responsiveness. **(b)** Expression patterns of photoreceptors under different light conditions. Gene name (*Kalanchoë* gene ID, *Arabidopsis* gene ID): *CRY2* (Kaladp0082s0193, AT1G04400), *PHOT1* (Kaladp0032s0316, AT3G45780), *PHOT2* (Kaladp0055s0063, AT5G58140), *phyA* (Kaladp0034s0172, AT1G09570), *phyB* (Kaladp0039s0298, AT2G18790), *phyE* (Kaladp0053s0072, AT4G18130). The expression data of *Arabidopsis* responsive to light quality (WL, BL, RL and FRL) was obtained from *Arabidopsis* eFP browser (light series). **(c)** Subnetwork of *PHOT1*. **(d)** Subnetwork of *phyA*. Orange nodes represent center of the subnetworks; red and green nodes represent TFs and circadian-/light responsive-genes, respectively. Large and small nodes represent the 1st and 2nd co-expressed genes, respectively. Thick and thin edges indicate the 1st and 2nd co-expression relationships, respectively.

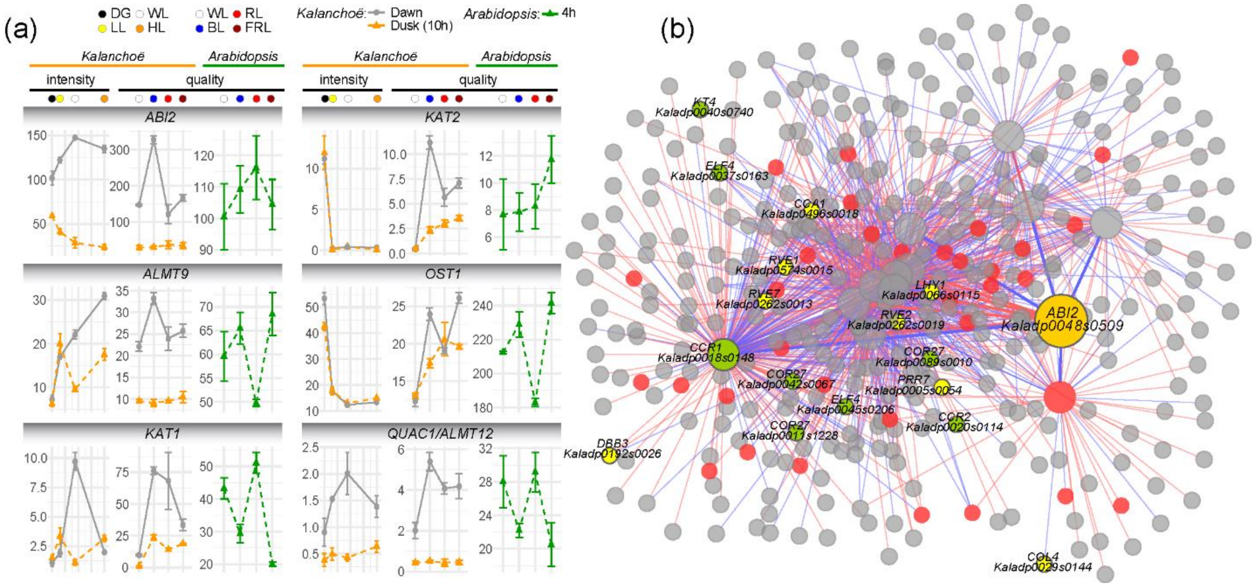

Fig. 9. The expression pattern of *Kalanchoë fedtschenkoi* stomatal movement-related genes.

(a) Expression of stomatal movement-related genes under different light conditions. Gene name (*Kalanchoë* gene ID, *Arabidopsis* gene ID): *ABI2* (Kaladp0048s0509, AT5G57050), *ALMT9* (Kaladp0062s0038, AT3G18440), *KAT1* (Kaladp0008s0789, AT5G46240), *KAT2* (Kaladp0840s0007, AT4G18290), *OST1* (Kaladp0016s0289, AT4G33950), *QUAC1/ALMT12* (Kaladp0091s0013, AT4G17970). The expression data of *Arabidopsis* responsive to light quality (WL, BL, RL and FRL) was obtained from *Arabidopsis* eFP browser (light series). **(b)** Subnetwork of stomatal movement-related gene *ABI2*. Orange nodes represent center of the subnetworks; green, yellow and red nodes represent TFs and circadian genes, circadian TFs and other TFs, respectively. Large and small nodes represent the 1st and 2nd co-expressed genes, respectively. Thick and thin edges indicate the 1st and 2nd co-expression relationships, respectively.

Supporting information

Additional Supporting Information may be found online in the Supporting Information section at the end of the article:

Figure S1. Expression distribution and correlation of 42 RNA-seq libraries.

(a) Distribution of gene expression levels of all the samples in this study. The gene expression levels were transformed by $\log_{10}$(TPM+1). (b) Pearson correlation between samples.

Figure S2. Gene ontology (GO) enrichment of DEGs in different comparisons.

BP, biological process; MF, molecular function; and CC, cellular component. GOslim terms were shown in here, full list of enriched GO terms was shown in Supplementary Table S3.

Figure S3. Functional classification of DEGs in MapMan BINs.

DEG number (a) and DEG percentage (b) of different comparisons in 29 MapMan BINs.

Figure S4. Expression pattern of DEGs in different MapMan BINs.

Color scale of blue-white-red represents Z-score normalized relative expression in the 14 samples.

Figure S5. Transcription factor (TF) number and enrichment in the co-expression modules.

Figure S6. Scatter plots (lower triangle) and correlations (upper triangle) among four physiological traits of *K. fedtschenkoi* under different light treatments.

Figure S7. The expression pattern of *Kalanchoë fedtschenkoi* circadian rhythm-related genes at dawn and dusk under different light conditions.

Table S1. Experimental conditions and statistic of RNA-Seq data in this study.

Table S2. Differentially expressed genes (DEGs) in pairwise comparisons.

Table S3. Full list of enriched GO terms of DEGs in different comparisons.

Table S4. Expression patterns of photosynthetic genes.

Table S5. Full list of enriched GO terms of different WGCNA modules.

Table S6. Transcription factors in WGCNA modules.

Table S7. Gene list and functional annotation of the sub-network.

Table S8. Expression patterns of CAM-, circadian-, stomatal movement-related and photosynthetic genes.

Table S9. Correlation of physiological parameters and gene expression.

Figure1

Click here to access/download;Figure;Figure 1.tiff

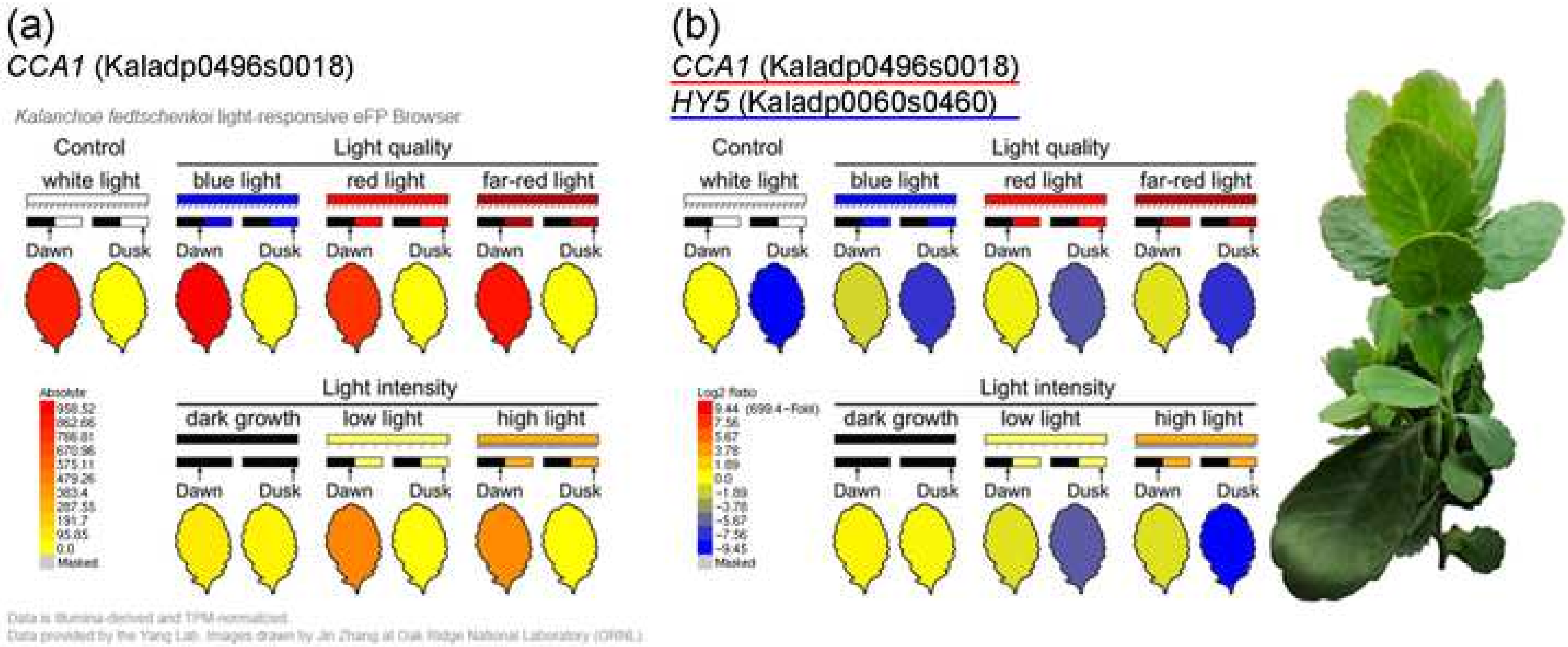

Figure2

Click here to access/download;Figure;Figure 2.tif

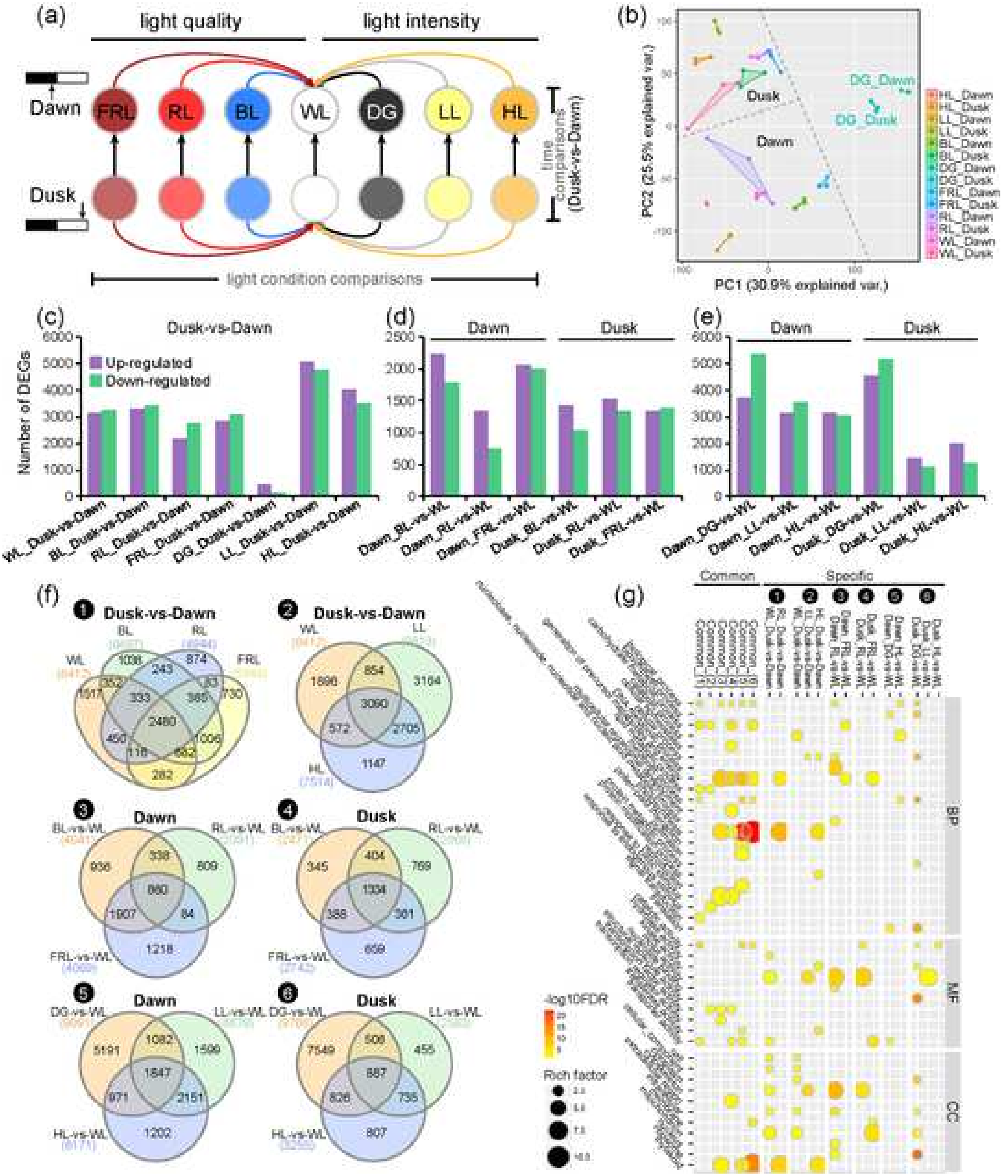

Figure3

Click here to access/download;Figure;Figure 3_Mapman.tif

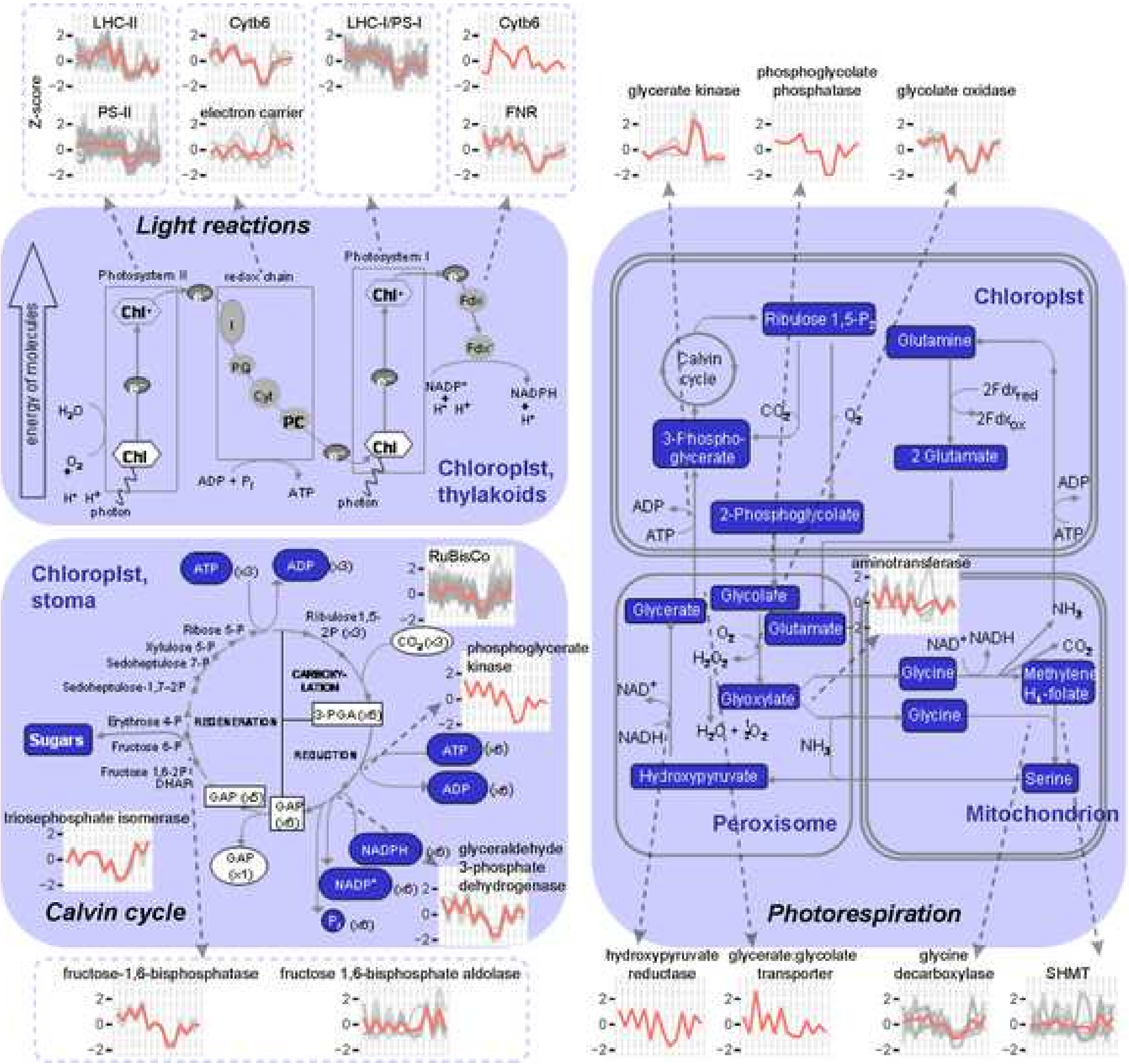

Figure4

Click here to access/download;Figure;Figure 4_WGCNA.tif

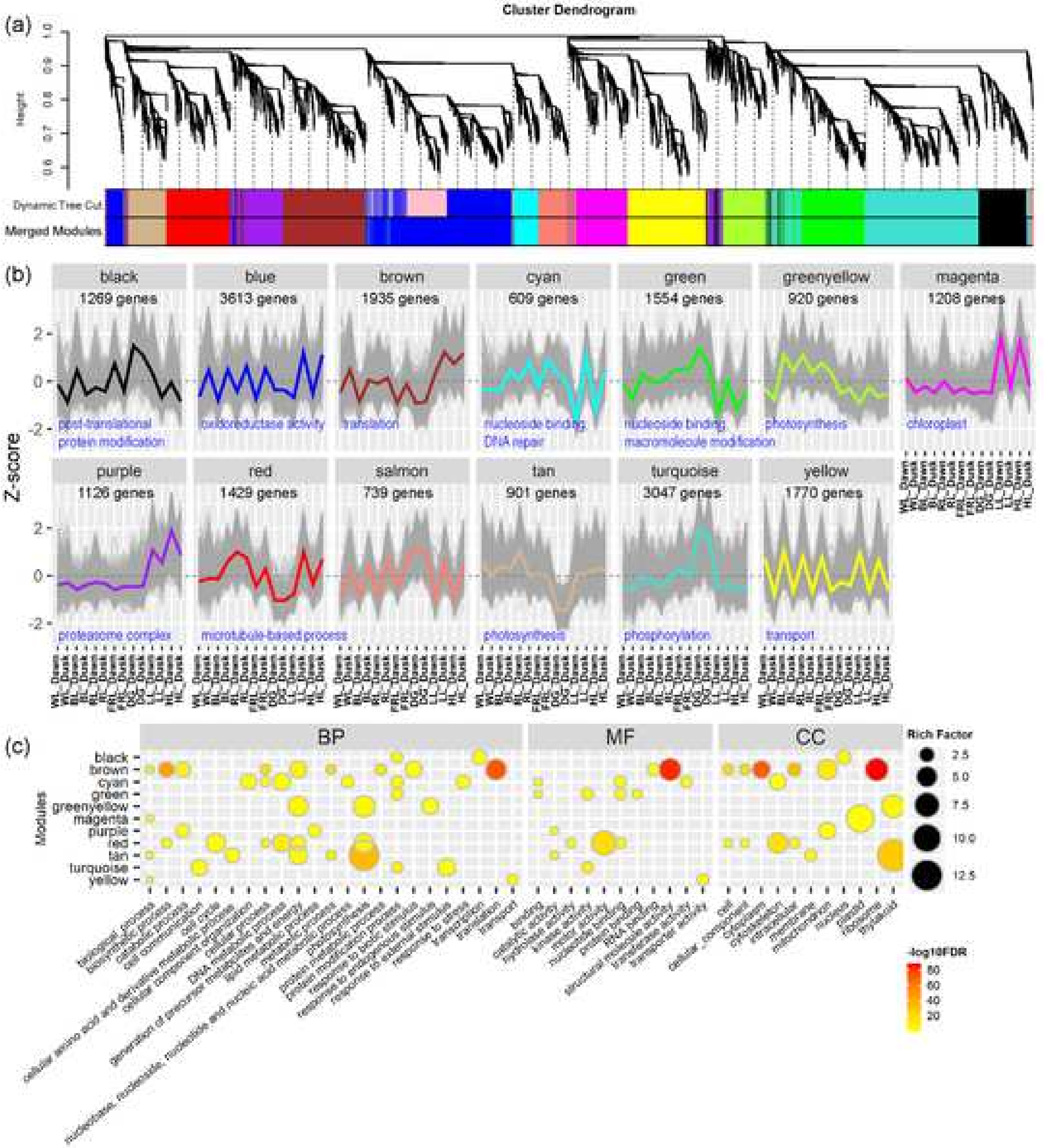

Figure5

Click here to access/download;Figure;Figure 5.tif

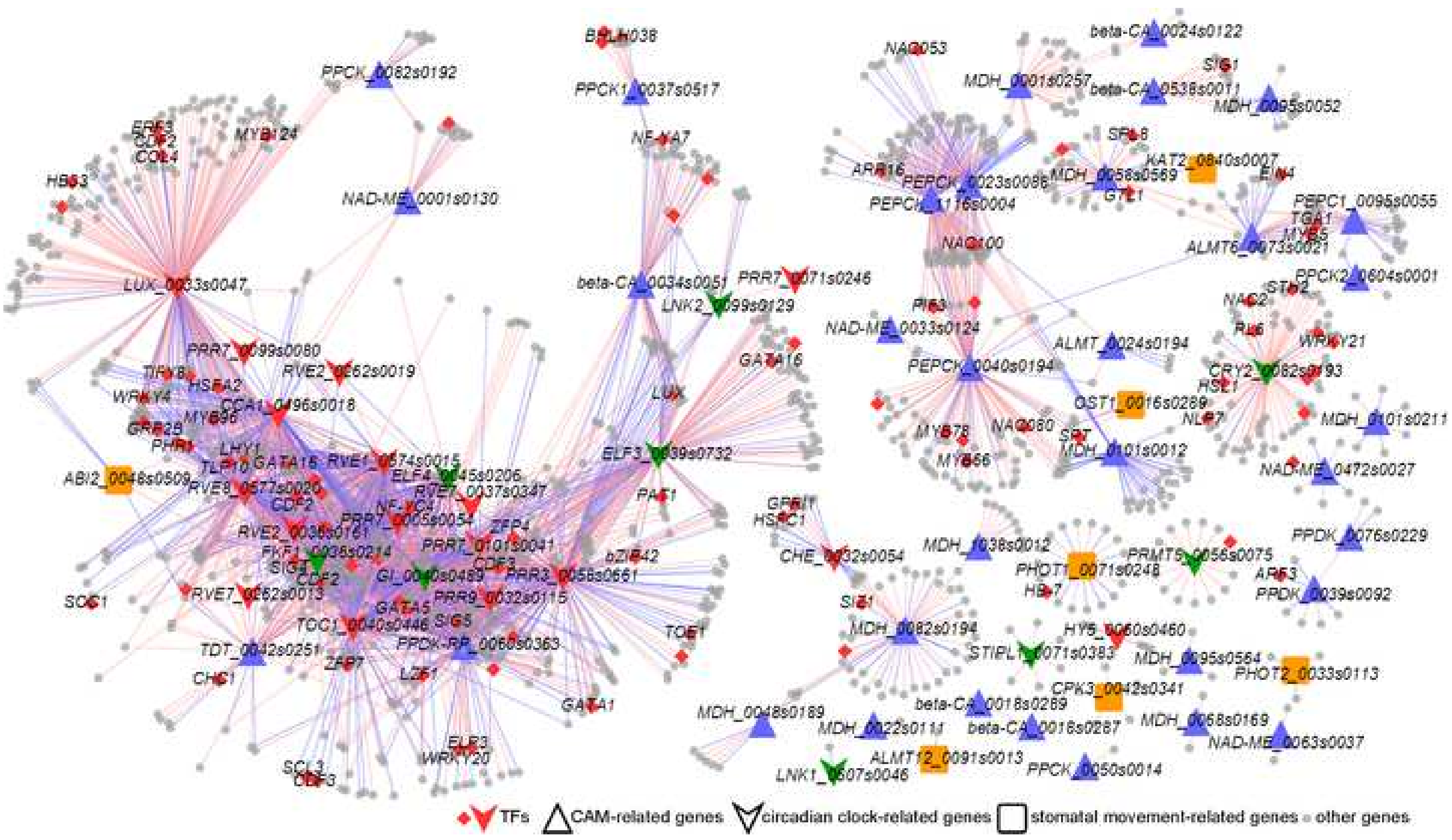

Figure6

Click here to access/download;Figure;Figure 6 _ TA.SS.Starch_V2.Jin.tif

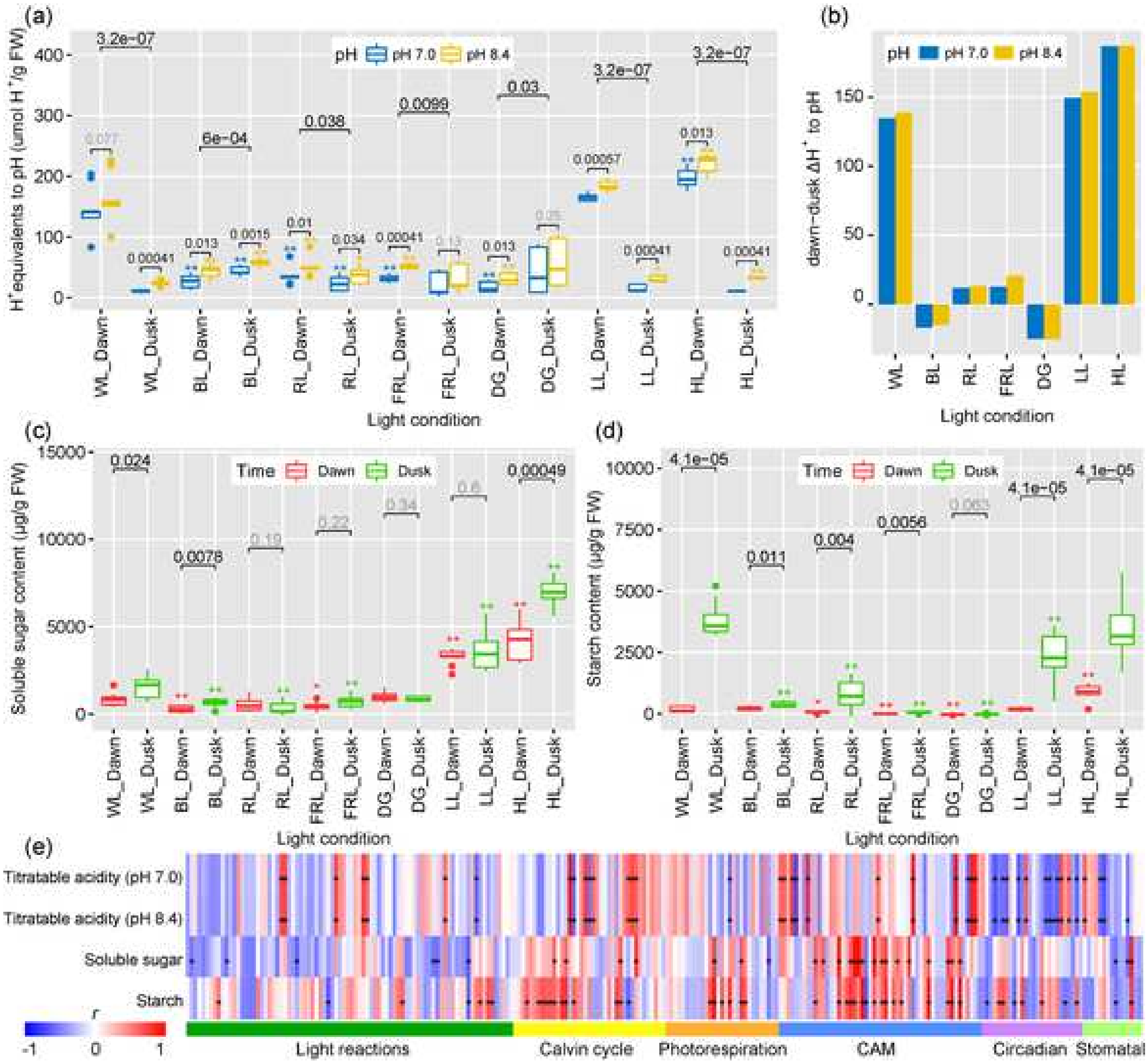

Figure7

Click here to access/download;Figure;Figure 7 _V3.tiff

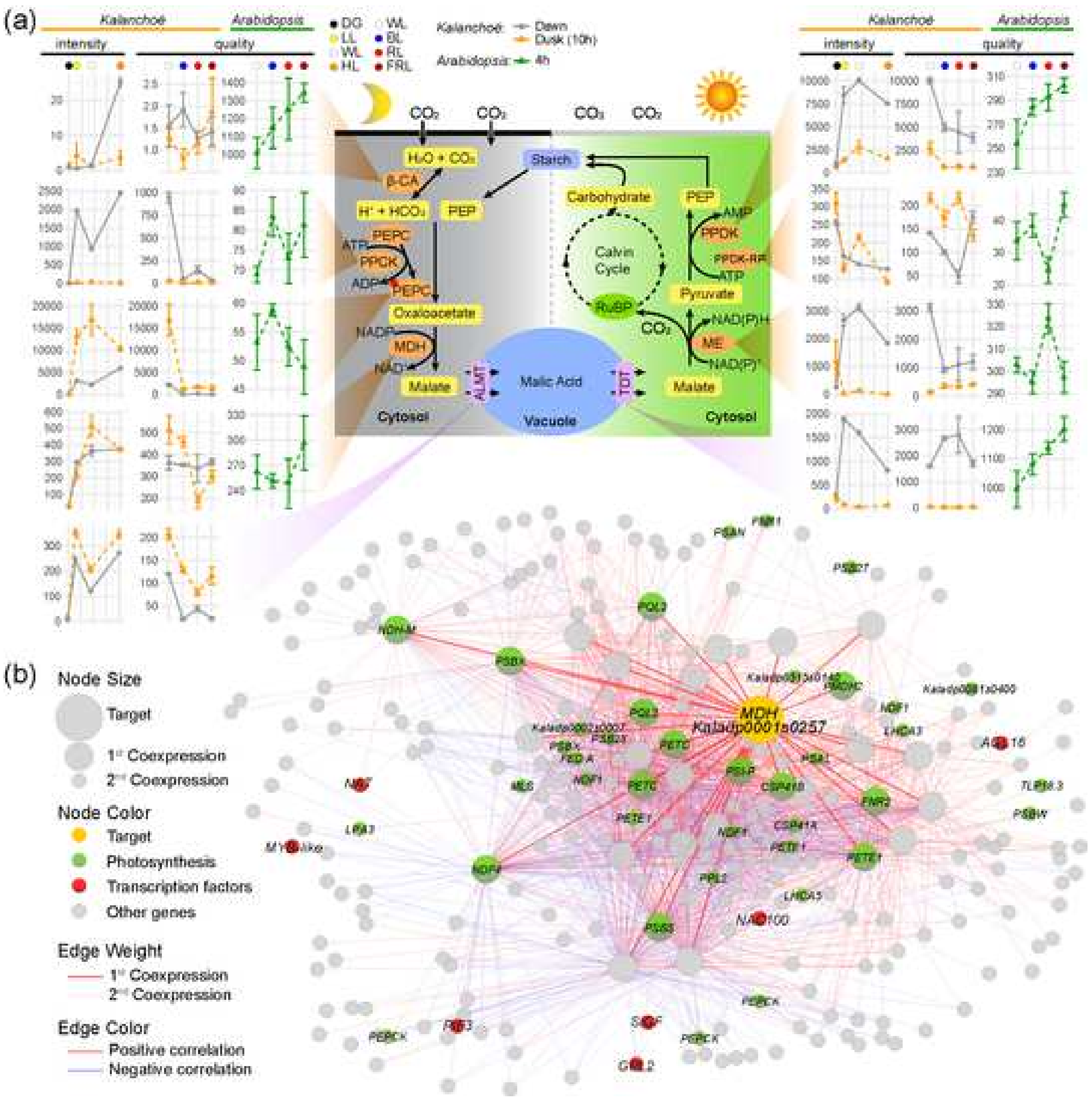

Figure8

Click here to access/download;Figure;Figure 8 _ light receptor_V4.tif

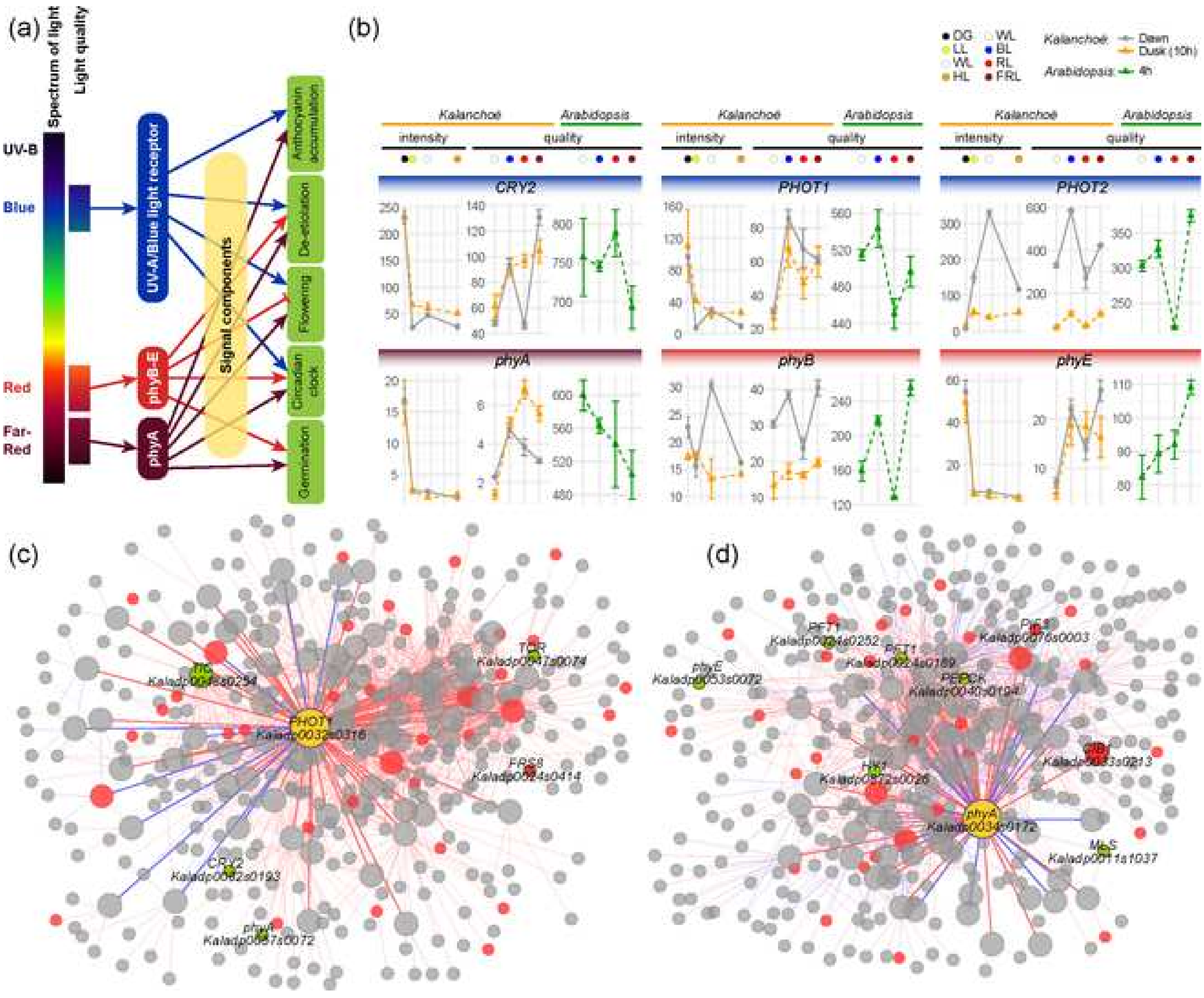

Figure9

Click here to access/download;Figure;Figure 9 _V2.tif

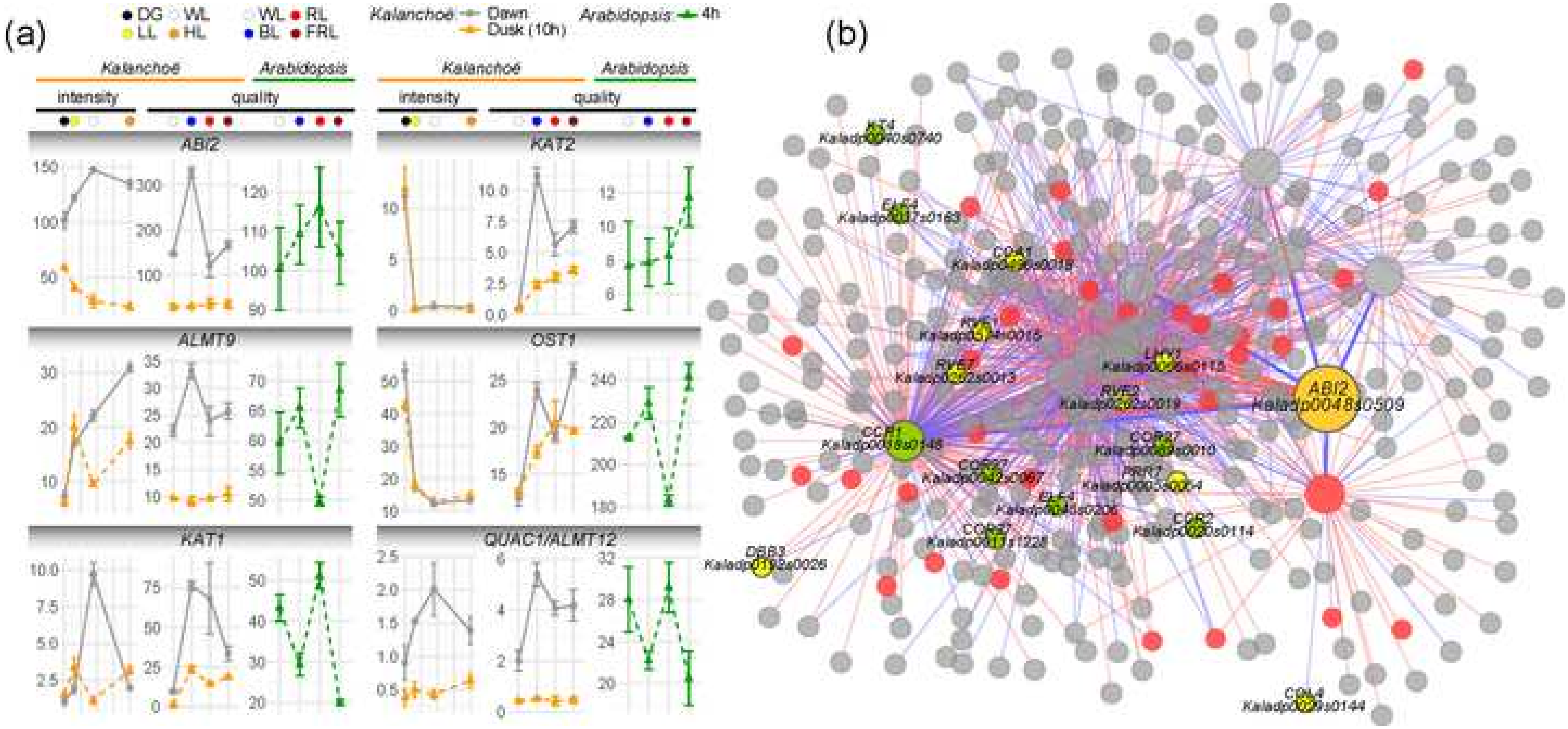

Click here to access/download
Supplementary Material
SI_JZ.docx

Supplementary Table S1

Click here to access/download
Supplementary Material
Table S1 _ Samples.xlsx

Supplementary Table S3

Click here to access/download
Supplementary Material
Table S3 _ GO all DEGs.xlsx

Supplementary Table S4

Click here to access/download
Supplementary Material
Table S4 _ Photosynthesis Mapman.xlsx

Supplementary Table S5

Click here to access/download
Supplementary Material
Table S5 _ WGCNA GO.xlsx

Supplementary Table S6

Click here to access/download

Supplementary Material

Table S6 _ WGCNA TFs.xlsx

Supplementary Table S7

Click here to access/download
Supplementary Material
Table S7 _ subnetwork.xlsx

Supplementary Table S8

Click here to access/download
Supplementary Material
Table S8 _ CAM genes.xlsx

Supplementary Table S9

Click here to access/download
Supplementary Material
Table S9 _ correlation.xlsx
