# Supplementary material for: Light-responsive expression atlas reveals the effects of light quality and intensity in Kalanchoë fedtschenkoi, a plant with crassulacean acid metabolism
Source: Gigascience. 2020 Mar 5;9(3):giaa018. doi: 10.1093/gigascience/giaa018 (PMC7058158; doi:10.1093/gigascience/giaa018)
Supplement: giaa018_GIGA-D-19-00095_Revision_2 [file giaa018_giga-d-19-00095_revision_2.pdf]

GigaScience

Light-responsive expression atlas reveals the effects of light quality and intensity in Kalanchoë fedtschenkoi, a plant with crassulacean acid metabolism

--Manuscript Draft--

**Manuscript Number:**	GIGA-D-19-00095R2
**Full Title:**	Light-responsive expression atlas reveals the effects of light quality and intensity in Kalanchoë fedtschenkoi, a plant with crassulacean acid metabolism
**Article Type:**	Data Note
**Funding Information:**	Genomic Science Program (DE-SC0008834) — Dr Xiaohan Yang<br>Community Science Program (503025) — Dr Xiaohan Yang
**Abstract:**	Background: Crassulacean acid metabolism (CAM), a specialized mode of photosynthesis, enables plant adaptation to water-limited environments and improves photosynthetic efficiency via an inorganic carbon-concentrating mechanism. Kalanchoë fedtschenkoi is an obligate CAM model featuring a relatively small genome and easy stable transformation. However, the molecular responses to light quality and intensity in CAM plants remain understudied.<br>Results: Here we present a genome-wide expression atlas of K. fedtschenkoi plants grown under 12h/12h photoperiod with different light quality (blue, red, far-red, white light) and intensity (0, 150, 440, and 1000 μmol m^{-2} s^{-1}) based on RNA-Seq performed for mature leaf samples collected at dawn (2-h before the starting of lighting period) and dusk (2-h before the dark period). An eFP web browser was created for easy access of the gene expression data. Based on the expression atlas, we constructed a light-responsive co-expression network to reveal the potential regulatory relationships in K. fedtschenkoi. Measurements of leaf titratable acidity, soluble sugar and starch turnover provided metabolic indicators of the magnitude of CAM under the different light treatments and were used to provide biological context for the expression dataset. Furthermore, CAM-related subnetworks were highlighted to showcase genes relevant to CAM pathway, circadian clock and stomatal movement. In comparison with white light, monochrome blue/red/far-red light treatments repressed the expression of several CAM-related genes at dusk, along with a major reduction in acid accumulation. Increasing light intensity from an intermediate level (440 mmol m^{-2} s^{-1}) of white light to a high light treatment (1000 μmol m^{-2} s^{-1}) increased expression of several genes involved in dark CO_2 fixation and malate transport at dawn, along with an increase in organic acid accumulation.<br>Conclusions: This study provides a highly useful genomics resource for investigating the molecular mechanism underlying the light regulation of physiology and metabolism in CAM plants. Our results support the hypothesis that both light intensity and light quality can modulate the CAM pathway through regulation of CAM-related genes in K. fedtschenkoi .
**Corresponding Author:**	Jin Zhang<br>Oak Ridge National Laboratory<br>oak ridge, TN UNITED STATES
**Corresponding Author Secondary Information:**	
**Corresponding Author's Institution:**	Oak Ridge National Laboratory
**Corresponding Author's Secondary Institution:**	
**First Author:**	Jin Zhang
**First Author Secondary Information:**	
**Order of Authors:**	Jin Zhang<br>Rongbin Hu

Powered by Editorial Manager® and ProduXion Manager® from Aries Systems Corporation

	Avinash Sreedasyam
	Travis Garcia
	Anna Lipzen
	Mei Wang
	Pradeep Yerramsetty
	Degao Liu
	Vivian Ng
	Jeremy Schmutz
	John Cushman
	Anne Borland
	Asher Pasha
	Nicholas Provart
	Jin-Gui Chen
	Wellington Muchero
	Gerald Tuskan
	Xiaohan Yang
**Order of Authors Secondary Information:**	
**Response to Reviewers:**	Dear Dr. Zhang,<br>Your revised manuscript "Light-responsive expression atlas reveals the effects of light quality and intensity in Kalanchoë fedtschenkoi, a plant with crassulacean acid metabolism" (GIGA-D-19-00095R1) has been re-assessed by our reviewers. There are some further comments which we hope you can address in a second revised version.<br><br>In light of the latest reports (included below), we had to discuss two main issues before making a decision (and I apologize for the delay this has caused):<br><br>1) There is still some concern whether the paper presents substantial biological insights.<br>2) Reviewer 1 raised a methodological issue that became apparent in the revised version: that only one plant per replicate was used, rather than three per replicate, which in the reviewer's opinion is a better approach.<br><br>Regarding point 1, I somewhat agree, but both reviewers highlight the value of your data as a resource. We therefore decided to consider your paper for our "Data Note" section of the journal, where we highlight exceptional datasets and use cases thereof. Please let us know if you agree with this change of article type. You do not need to make any major changes to the manuscript itself for our "Data note" section, and you can fit your existing structure (headings etc) as sub-headings to the structure of the "Data Note" article type, with a few minor changes (see the "information for authors" on our homepage).<br>[Response:] Thanks for your comments. We agree to change the article type as “Data Note” without major changes to the current version of our manuscript.<br><br>Regarding point 2), we also discussed the issue with the other reviewer and don't see it as a critical flaw. However, the reviewers agree that pooling is generally a comparatively easy and cost effective method to reduce the variance, and ultimately increase the statistical power. Please discuss in the manuscript the potential advantage of the alternative "three and three" approach, and how your approach with only one plant per replicate may affect the results - i.e. more false negatives are expected with higher variance, etc.<br>[Response:] Actually, one individual for each biological replicate has been widely used in plant and animal studies. For example, researchers used one individual as a biological replicate for RNA-Seq and/or qPCR analyses in mouse (Burger et al. 2018), bird (Mead et al. 2017), soybean (Corcilius et al. 2017), etc. According to Bell (2016),

biological replicates are biologically distinct samples, which show biological variation. An individual plant is a biologically distinct unit with a complete set of genetics and physiological characteristics, and therefore can be treated as a biological replicate. Pooling multiple plants into each biological replicate would be a good choice if one single plant cannot provide enough material for RNA isolation. Our strategy using one individual plant as a biological replicate can capture the variation in gene expression among individual plants, and therefore could potentially reduce the false positive rate of differential gene expression in comparison with biological replicates containing multiple plants.
According to your suggestion, we have added the relevant description in Discussion line 393: “We used one plant for each of the three biological replicates for RNA-Seq analysis, which could potentially reduce the false positive rate of differential gene expression in comparison with pooling multiple plants into each biological replicate.”

•Burger, Laura L., et al. "Identification of genes enriched in GnRH neurons by translating ribosome affinity purification and RNAseq in mice." Endocrinology 159.4 (2018): 1922-1940.
•Mead, Andrew F., et al. "Fundamental constraints in synchronous muscle limit superfast motor control in vertebrates." Elife 6 (2017): e29425.
•Corcilius, Leo, et al. "Arabinosylation modulates the growth-regulating activity of the peptide hormone CLE40a from soybean." Cell chemical biology 24.11 (2017): 1347-1355.
•Bell, Graham. "Replicates and repeats." BMC biology 14.1 (2016): 28.

Please also address all other comments of both reviewers (see the reports below).
=================

Reviewer #1: Summary of the key results
Overall evaluation
===========
As I stated in my previous revision, I think that the authors developed an useful resource for the scientific community working in the photosynthesis field, specially for CAM plants. The new version of the manuscript describes with more detail some of the pathways, but I still think that its hypothesis is not well developed. The new measures for leaf titratable acidity, soluble sugar and starch are interesting, but now that the authors added more details for the experimental design, it is worrying the low number of plants used per replicate (line 519: "three biological replicates of plants (each biological replicate was one independent plant)"). Personally I think that this type of experiments should use at least three independent plant per replicate (although five are highly recommended), with a total of at least nine plants per condition. The use of one plant per replicate may not be necessarily wrong, but it is not an strong design. Summarizing, I think that the manuscript is still an useful resource although it is descriptive (less than in the previous version). Discussion has been improved but some of the details about the experiment design show a low number of independent plants used in the experiment.

Major concerns
==========
*The proposed hypothesis behind the experiment is still not well developed. The manuscript is still mostly descriptive. I see the article as an useful resource to mine information as the author mentioned at the end of the "Background" section. Nevertheless, in my opinion, this manuscript still does not have a strong hypothesis behind the experiment. The authors wrote (line 116) "We hypothesize that both light intensity and light quality can influence the expression of CAM related metabolic and signaling genes in the obligate CAM species K. fedtschenkoi". Indeed this type of hypothesis are simple (light should influence a photosynthetic mechanism) and difficult to test (e.g. how you measure or test the influence?).
[Response:] Thanks for your comments. According to the editor’s suggestion, we would like to submit our manuscript as “Data Note”.

*Poor experimental design. One major issue that pop-up reading this revised version of the manuscript is that the authors used only one plant per replicate (three total plants per condition). Although the use of a low number of plants per replicates may not be wrong if a minimum number of three replicates are used, I think that represent a poor

experimental design because it the source of variation using only one individual per replicate may drive to wrong interpretations or not solid enough results. It is recommended at least three replicates and at least three independent plants per replicate. Probably statisticians will argue that three plants per replicate is not solid either but I think that there is some kind of compromise in the community of what it is a good number of replicates and individuals per replicates and how many resources can be invested, and as I know it is three and three.

[Response:] Thanks for your comments.

Actually, one individual for each biological replicate has been widely used in plant and animal studies. For example, researchers used one individual as a biological replicate for RNA-Seq and/or qPCR analyses in mouse (Burger et al. 2018), bird (Mead et al. 2017), soybean (Corcilius et al. 2017), etc. According to Bell (2016), biological replicates are biologically distinct samples, which show biological variation. An individual plant is a biologically distinct unit with a complete set of genetics and physiological characteristics, and therefore can be treated as a biological replicate. Pooling multiple plants into each biological replicate would be a good choice if one single plant cannot provide enough material for RNA isolation. Our strategy using one individual plant as a biological replicate can capture the variation in gene expression among individual plants, and therefore could potentially reduce the false positive rate of differential gene expression in comparison with biological replicates containing multiple plants.

•Burger, Laura L., et al. "Identification of genes enriched in GnRH neurons by translating ribosome affinity purification and RNAseq in mice." Endocrinology 159.4 (2018): 1922-1940.
•Mead, Andrew F., et al. "Fundamental constraints in synchronous muscle limit superfast motor control in vertebrates." Elife 6 (2017): e29425.
•Corcilius, Leo, et al. "Arabinosylation modulates the growth-regulating activity of the peptide hormone CLE40a from soybean." Cell chemical biology 24.11 (2017): 1347-1355.
•Bell, Graham. "Replicates and repeats." BMC biology 14.1 (2016): 28.

Reviewer #2: The authors have made major improvements to the analyses and manuscript, and the resource they provide to the community is now much more approachable, and, inasmuch, more valuable than before.

I have only a few rather minor comments remaining:

- at, e.g., line 196, and Figure S2, Fig2 c-e:

Be consistent in how you refer to up-regulated and down-regulated genes. My personal suggestion would be to avoid terms with DEGs (e.g. "increased abundance Differentially Expressed Genes" or "up DEGs") and simply call them "up-regulated genes".

The same idea goes for down-regulated genes.

[Response:] According to your suggestion, we have revised the relevant description to “up-regulated genes” and “down-regulated genes” throughout the manuscript.

- Fig 2g:

This has improved, but I am unfortunately still confused about this figure.

First aesthetics:

The labels are very small and too close to each other to be easily read.

Can MF and CC be moved to the supplemental?

Or can the specific comparisons be split off?

Or can this have it's own figure / somehow be larger?

[Response:] According to your suggestion, we have moved MF and CC to the supplementary Figure S3.

Second content:

I know it's probably somewhere in the paper, but I can't find a clear description of what "specific" DEGs are in the figure or the figure legend.

Clearly the specific "WL_Dusk_vs_Dawn" out of the light quality comparisons (common 1) does not match "WL_Dusk_vs_Dawn" out of the light intensity comparisons (common 2).

But I can't tell if the former is just the 1517 DEGs that are significantly different (dusk vs

dawn) _only_ under white light, or if it is the (6412 - 2480) DEGs that are significantly different under white light, but not for all light quality dusk vs dawn comparisons.
Or perhaps it's something else entirely?
Similarly, I'm left unsure if the "common" category refers to DEGs shared by _all_ the comparisons in a given set, or if they are shared by any two-or more of the comparisons in a given set.

[Response:] To clarify the “common” and “specific” DEGs, we added the description in Figure 2 legend:
(g) Gene ontology (GO) enrichment of common DEGs shared by different comparisons in (f) Venn diagrams (DEGs shared by all the comparisons in a given set) or specific DEGs (genes only differentially expressed in one of the overlapped comparisons). Biological process (BP). Detailed enrichment of molecular function (MF) and cellular component (CC) was shown in Supplementary Figure S3. GOslim terms were shown in here.

Further, still taking light quality (common 1) as an example, I'm confused about why only some of the comparisons' are reported here; why only WL and RL, why not BL or FRL?

[Response:] There is no significant enriched GO terms for those comparisons, which are not included in the figure. In updated Figure 2g and Figure S3, we have added all the comparisons.

Finally, one small detail: "Enrichment factor" not "Rich factor" (fix throughout the paper please)

[Response:] We have revised it as “Enrichment factor” throughout the manuscript, including Figure 2g, Figure 4c, Table S3, and Table S5.

If a full explanation is hard to fit in the figure legend, I'd be happy with including a reference to a sub-header in the methods or perhaps supplemental where all the details can be found. As long as the reader doesn't have to search.

[Response:] To clarify the “common” and “specific” DEGs, we added the description in Figure 2 legend:
(g) Gene ontology (GO) enrichment of common DEGs shared by different comparisons in (f) Venn diagrams (DEGs shared by all the comparisons in a given set) or specific DEGs (genes only differentially expressed in one of the overlapped comparisons). Biological process (BP). Detailed enrichment of molecular function (MF) and cellular component (CC) was shown in Supplementary Figure S3. GOslim terms were shown in here.

- at 338:
I don't think that the Arabidopsis efp browser is the _only_ publicly available light-quality expression data. It could well be the most comparable data and it's totally reasonable to use the data, I just wouldn't use this particular sentence.

[Response:] We have removed this particular sentence in the revised manuscript.

-- End --

**Additional Information:**	
**Question**	**Response**
Are you submitting this manuscript to a special series or article collection?	No
**Experimental design and statistics**<br><br>Full details of the experimental design and statistical methods used should be given in the Methods section, as detailed in our Minimum Standards Reporting Checklist. Information essential to interpreting the data presented should be made available	Yes

Powered by Editorial Manager® and ProduXion Manager® from Aries Systems Corporation

in the figure legends.<br><br>Have you included all the information requested in your manuscript?	
**Resources**<br><br>A description of all resources used, including antibodies, cell lines, animals and software tools, with enough information to allow them to be uniquely identified, should be included in the Methods section. Authors are strongly encouraged to cite Research Resource Identifiers (RRIDs) for antibodies, model organisms and tools, where possible.<br><br>Have you included the information requested as detailed in our Minimum Standards Reporting Checklist?	Yes
**Availability of data and materials**<br><br>All datasets and code on which the conclusions of the paper rely must be either included in your submission or deposited in publicly available repositories (where available and ethically appropriate), referencing such data using a unique identifier in the references and in the "Availability of Data and Materials" section of your manuscript.<br><br>Have you have met the above requirement as detailed in our Minimum Standards Reporting Checklist?	Yes

Powered by Editorial Manager® and ProduXion Manager® from Aries Systems Corporation

Manuscript
Click here to access/download;Manuscript;Kalanchoe_Light_mainText_forPub

Article type: Data Note

Light-responsive expression atlas reveals the effects of light quality and intensity in *Kalanchoë fedtschenkoi*, a plant with crassulacean acid metabolism

Jin Zhang[1,2,a], Rongbin Hu[1,a], Avinash Sreedasyam[3], Travis Garcia[4], Anna Lipzen[5], Mei Wang[5], Pradeep Yerramsetty[4], Degao Liu[1], Vivian Ng[5], Jeremy Schmutz[3,5], John C. Cushman[4], Anne M. Borland[1,6], Asher Pasha[7], Nicholas J. Provart[7], Jin-Gui Chen[1,2], Wellington Muchero[1,2], Gerald A. Tuskan[1,2], Xiaohan Yang[1,2,*]

[1]*Biosciences Division, Oak Ridge National Laboratory, Oak Ridge, TN 37831-6422, USA;*
[2]*The Center for Bioenergy Innovation, Oak Ridge National Laboratory, Oak Ridge, TN 37831, USA;*
[3]*HudsonAlpha Institute for Biotechnology, 601 Genome Way, Huntsville, AL 35801, USA;*
[4]*Department of Biochemistry and Molecular Biology, University of Nevada, Reno, NV 89557, USA;*
[5]*US Department of Energy Joint Genome Institute, 2800 Mitchell Drive, Walnut Creek, CA 94598, USA;*
[6]*School of Natural and Environmental Science, Newcastle University, Newcastle upon Tyne NE1 7RU, UK;*
[7]*Department of Cell and Systems Biology, Centre for the Analysis of Genome Evolution and Function, University of Toronto, Toronto, ON M5S 3B2, Canada*

[a] These authors contributed equally to this manuscript.
*Correspondence: Xiaohan Yang (yangx@ornl.gov) Tel +1 865 241 6895; fax +1 865 576 9939

Emails: Jin Zhang (zhangj1@ornl.gov), Rongbin Hu (hu.rongbin@gmail.com), Avinash Sreedasyam (asreedasyam@hudsonalpha.org), Travis Garcia (tgarcia2@unr.edu), Anna Lipzen (alipzen@lbl.gov), Mei Wang (mwang@lbl.gov), Pradeep Yerramsetty (ypradeepkalyan@gmail.com), Degao Liu (liudegao909@gmail.com), Vivian Ng (vng@lbl.gov), Jeremy Schmutz (jschmutz@hudsonalpha.com), John C. Cushman (jcushman@unr.edu), Anne M. Borland (anne.borland@newcastle.ac.uk), Asher Pasha (asher.pasha@utoronto.ca), Nicholas J. Provart (nicholas.provart@utoronto.ca), Jin-Gui Chen (chenj@ornl.gov), Wellington Muchero (mucherow@ornl.gov), Gerald A. Tuskan (tuskanga@ornl.gov), Xiaohan Yang (yangx@ornl.gov)

Figures: 9 figures (in color).
Supplementary Data: 7 supplementary figures and 9 supplementary tables.

Abstract

Background: Crassulacean acid metabolism (CAM), a specialized mode of photosynthesis, enables plant adaptation to water-limited environments and improves photosynthetic efficiency via an inorganic carbon-concentrating mechanism. *Kalanchoë fedtschenkoi* is an obligate CAM model featuring a relatively small genome and easy stable transformation. However, the molecular responses to light quality and intensity in CAM plants remain understudied.

Results: Here we present a genome-wide expression atlas of *K. fedtschenkoi* plants grown under 12h/12h photoperiod with different light quality (blue, red, far-red, white light) and intensity (0, 150, 440, and 1000 μmol m^{-2} s^{-1}) based on RNA-Seq performed for mature leaf samples collected at dawn (2-h before the starting of lighting period) and dusk (2-h before the dark period). An eFP web browser was created for easy access of the gene expression data. Based on the expression atlas, we constructed a light-responsive co-expression network to reveal the potential regulatory relationships in *K. fedtschenkoi*. Measurements of leaf titratable acidity, soluble sugar and starch turnover provided metabolic indicators of the magnitude of CAM under the different light treatments and were used to provide biological context for the expression dataset. Furthermore, CAM-related subnetworks were highlighted to showcase genes relevant to CAM pathway, circadian clock and stomatal movement. In comparison with white light, monochrome blue/red/far-red light treatments repressed the expression of several CAM-related genes at dusk, along with a major reduction in acid accumulation. Increasing light intensity from an intermediate level (440 μmol m^{-2} s^{-1}) of white light to a high light treatment (1000 μmol m^{-2} s^{-1}) increased expression of several genes involved in dark CO_2 fixation and malate transport at dawn, along with an increase in organic acid accumulation.

Conclusions: This study provides a highly useful genomics resource for investigating the molecular mechanism underlying the light regulation of physiology and metabolism in CAM plants. Our results support the hypothesis that both light intensity and light quality can modulate the CAM pathway through regulation of CAM-related genes in *K. fedtschenkoi*.

Keywords: eFP browser; gene atlas; transcriptome; *Kalanchoë fedtschenkoi*; crassulacean acid metabolism

Background

Sunlight is a critical energy resource for plant growth and development, and functions as an important input signal for circadian clock, stomatal movement, and photosynthesis pathway. The light spectra that affect plant photosynthesis are UV-A/blue, red, and far-red [1, 2]. Blue light, with wavelength of 400 to 500 nm, has a higher energy than red light (wavelength from 600 to 700 nm) and far-red light (wavelength above 700 nm) [1, 3]. There are three types of photoreceptors (i.e., cryptochromes, phototropins and phytochromes) that perform important roles in plant light response [2, 4]. Cryptochromes and phototropins have been identified as important photoreceptors of UV-A/blue light [2, 5, 6]. Phytochromes are known to play a role in detecting red and far-red spectra [2, 7]. In addition to the light quality, light intensity is another essential factor that affects plant growth and development, where either too much or little light can cause stress, including serious damage to photosynthetic apparatus under excess light exposure and limited photosynthetic activity with insufficient light input [8-10].

Plants using crassulacean acid metabolism (CAM) pathway for photosynthesis show enhanced water-use efficiency (WUE) and heat/drought stress tolerance in comparison with C_3 and C_4 photosynthesis plants [11, 12]. The CAM pathway has two major features: (1) a carboxylation process that takes place at night where stomata are open for nocturnal CO_2 fixation and accumulation of malic acid in the vacuole and (2) a decarboxylation process that occurs during the daytime where CO_2 is released from malate for refixation via ribulose-1,5-bisphosphate carboxylase/oxygenase (Rubisco)-mediated photosynthesis, along with stomatal closure which reduces evapotranspiration [11, 13, 14]. *Kalanchoë fedtschenkoi* is a model dicot CAM species, featuring a relatively small genome and a facile stable transformation system [11, 15]. The genome of *K. fedtschenkoi* was recently sequenced and annotated [13], providing a foundation for CAM genomics research. Comparative and evolutionary genomics analyses revealed convergent signatures in diel gene expression pattern and protein sequences underlying independent emergences of CAM from C_3 ancestor, providing new insights into CAM evolution [13]. However, the complex regulatory mechanisms that underpin the diel optimization of the CAM pathway under various light conditions remain largely unexplored.

The temporal separation of C_3 and C_4 carboxylation processes that defines CAM provides plasticity for optimizing carbon gain and water use in response to changing environmental

conditions by extending or curtailing the period of net CO_2 uptake over a 24-h period [16]. Light intensity (photosynthetic photon flux density, PPFD) and light quality are critical factors for controlling the performance of CAM, which implies cardinal roles for the light reactions of photosynthesis and for different photoreceptors in achieving metabolic and circadian synchronization of carboxylation processes across the diel cycle. In some facultative CAM species, high-light intensity can trigger the switch from C_3-photosynthesis to CAM, which is mediated by a UV-A/blue light receptor [17]. Metabolic and physiological adaptation in constitutive CAM species plants to light quantity and quality has been reported previously [18-21]. For instance, physiological and metabolic responses under severe light stress under short- and long-term treatments were reported [18]. Metabolic changes under different light spectrum (i.e., blue, green and red light) were also reported in an obligate CAM species *Aechmea 'Maya'* [19]. Exposure of a woody CAM species *Clusia hilariana* to low- or high-light affected the production of malate and citrate [21]. However, the molecular basis of the metabolic and signaling pathways that underpin changes to the operation of CAM in response to various spectral light qualities and intensity has not been investigated extensively.

High-throughput, next-generation sequencing has been widely applied to genome-wide expression analysis. As a new type of web-based tool, a genome-wide atlas of gene expression can provide comprehensive gene expression profiles in different tissues or different development stages. To date, gene expression atlases have been established for several C_3 photosynthesis species, including dicot *Arabidopsis* [22], *Medicago* [23], tomato [24], monocot wheat [25], and *Brachypodium* [26]. However, a genome-wide expression atlas has not been created for a CAM species. In addition, gene expression patterns and gene modules associated with plant response to light quality and light intensity are largely unknown, especially for CAM plants.

We hypothesize that both light intensity and light quality can influence the expression of CAM-related metabolic and signaling genes in the obligate CAM species *K. fedtschenkoi*. To test this hypothesis, we performed transcriptome-sequencing (RNA-Seq) of mature *K. fedtschenkoi* leaf samples collected at dawn (i.e., 2-h before the starting of lighting period) and dusk (2-h before the dark period) from plants grown under 12h/12h photoperiod with different light quality (i.e., blue, red, far-red, white light) and intensity (0, 150, 440, and 1000 μmol m^{-2} s^{-1}). Based on our analysis of the RNA-Seq data, we generated a comprehensive light-responsive gene expression atlas for

this obligate CAM species. We also constructed a genome-wide co-expression network based on the light-responsive gene expression atlas. To provide a metabolic context for the genome-wide co-expression network, we measured the titratable acidity (nocturnal malate and citrate accumulation) and soluble sugar and starch turnover. Nocturnal acid accumulation provides a quantitative measure of CAM activity whilst diel turnover of sugars and starch, provides an indication of the nocturnal supply and day-time demand for carbon processing. The subnetworks of CAM-related genes, photoreceptors, and stomatal movement-related genes indicated that the light-responsive expression atlas provides molecular clues for various physiological phenotypes. As comprehensive light-responsive gene atlas and co-expression network for CAM plants, this study provides an unprecedented genomics resource for investigating molecular mechanisms underlying the light regulation of biological processes in CAM plants.

Data Description

A total of 42 libraries (7 light conditions × 2 time points × 3 biological replicates) were constructed and performed RNA-seq independently. In total, we obtained 981 million read pairs (2×150 bp) with 287.78 Gb high quality data (Qscore≥25) from the 42 libraries, with an average size of ~23.4 million read pairs per library (Supplementary Table S1).

Analyses

Light-responsive expression atlas for *K. fedtschenkoi*

To obtain a comprehensive light-responsive gene-expression atlas of the CAM plant *K. fedtschenkoi*, we treated plants under control condition [white light (WL) with 440 μmol m^{-2} s^{-1} intensity], various light quality including blue light (BL), red light (RL), far-red light (FRL), and different light intensities, including dark grown (DG), low-light (LL) and high-light (HL) (Supplementary Table S1). As CAM pathway is likely regulated by the circadian clock, we compared if circadian rhythm-related processes were also affected by different light conditions. The samples were collected at two time points [dawn (2 h before light period) and dusk (2 h before dark period)] for each light condition. To provide easy access to the expression data, we created a

Kalanchoë light-responsive eFP browser (http://bar.utoronto.ca/efp_kalanchoe/cgi-bin/efpWeb.cgi), which provides a color-coding tissue visualization in an image corresponding to the average gene expression level (Fig. 1).

The Pearson correlation analysis and principle component analysis showed that the biological replicates of each treatment group were closely clustered, indicating the high reproducibility and reliability of our RNA-seq data (Fig. 2 and Supplementary Fig. S1). The principal component 1 (PC1) and PC2 explained 30.9% and 25.5% of the variance in the expression data, respectively. As expected, the samples collected at dawn and dusk were grouped separately under different light quality and light intensity except dark grown (Fig. 2b), and the expression variation of samples under various light conditions was stronger at dawn than the variation at dusk.

Differentially expressed genes (DEGs) regulated by light quality and light intensity

As shown in Fig. 2a, we performed a comparative transcriptomic analysis for screening of DEGs by using two different strategies (i.e., time comparison and light condition comparison). The time comparison was defined as the comparison between two samples collected at two different time points (i.e., dawn and dusk) under each light condition (i.e., dusk *vs*. dawn). The light condition comparison reflected a comparison between treatments and control at the same sample collection time point (i.e., BL/RL/FRL *vs*. WL) at dawn or dusk for light quality (i.e., DG/LL/HL *vs*. WL) at dawn or dusk for light intensity, respectively (Fig. 2a and Supplementary Table S2).

Under normal light condition (WL), 6,412 DEGs were identified in dusk vs. dawn. Of these DEGs, 3,137 and 3,275 genes showed either up- or down-regulated, respectively (Fig. 2). For different light intensity, both the low- and high-intensity light treatments enhanced the differential gene expression between dusk and dawn. Under the dark-grown condition, only 631 DEGs (458 up-regulated and 173 down-regulated) were identified in dusk *vs*. dawn comparison, which was significantly less than the comparisons made under the other light conditions (Fig. 2 and Supplementary Table S2).

Under the different light qualities, a total of 2,480 DEGs between dusk and dawn were shared by the four light spectrums (i.e., WL, BL, RF and FRL), indicating these genes might play essential roles in response to changes in light quality (Fig. 3a). Under various light intensity conditions,

3,090 genes were consistently differentially expressed under the three light intensities (WL, LL and HL), suggesting that these genes might play key roles in responding to diel or circadian cues and were not affected by light intensity (Fig. 3b).

Light condition comparisons were based on differences between different light quality or light intensity (Fig. 2a). The DEG number in most light-quality comparisons was greater at dawn than those at dusk (Fig. 2d). However, the overlapped DEGs were fewer at dawn (860 common DEGs, Fig. 2f) than at dusk (1,334 common DEGs, Fig. 2f) under the different light quality treatments. In contrast, the light quality-specific DEGs were greater at dawn than at dusk under blue light and far-red light. For the different light intensities, more than half of the DEGs under low light and high light were shared at both dawn and dusk (Fig. 2f).

Predicted function of DEGs

To explore the functional differences of DEGs induced by various light quality and light intensity treatments, we performed a gene ontology (GO) enrichment analysis of DEGs in different comparisons according to the three major GO categories of biological process (BP), molecular function (MF), and cellular component (CC) (Supplementary Fig. S2). Notably, all DEGs in the light intensity and light quality comparisons were enriched in "photosynthesis" process; but when the up-regulated or down-regulated DEGs were separated, only up-regulated genes in light quality comparisons and down-regulated genes in light intensity comparisons were highly enriched in "photosynthesis" process. Similarly, the "response to biotic stimulus" term in light quality for all DEGs was depleted (Supplementary Fig. S2 and Table S3).

In addition, we compared the enriched GO terms of common and light-specific DEGs in response to the light treatments (Fig. 2g, Supplementary Fig. S3 and Table S4). Among the DEGs between dusk and dawn, the 2,480 DEGs that overlapped across different light-quality treatments (Fig. 2f) were enriched in "carbohydrate metabolic process", "lipid metabolic process", "metabolic process" and "signal transduction"; whereas the 3,090 DEGs that overlapped across different light-intensity treatments were enriched in "carbohydrate metabolic process" and "response to endogenous stimulus" (Fig. 2g). When comparing the common DEGs in different light quality or light intensity at dawn and dusk separately (Fig. 2f), we found that "generation of precursor metabolites and energy" term was enriched in all the four common DEGs sets of different light

quality and light intensity at both dawn and dusk. In contrast, the "photosynthesis" term was enriched in common DEGs of light quality at dusk and common DEGs of light intensity at both dawn and dusk (Fig. 2g), whereas the "carbohydrate metabolic process" term was only enriched at the dusk time point of different light quality and light intensity (Fig. 2g).

For condition-specific DEGs at different time points or different light conditions, the "photosynthesis" term was strongly enriched in dusk *vs*. dawn DEGs in RL-specific and HL-specific from the light-quality comparison and light-intensity comparison, respectively (Fig. 2g). This indicates that RL and HL significantly affected photosynthesis-related changes in transcript abundance between dawn and dusk. When different light quality or light intensity treatments were compared to the white light control at dawn and dusk, the "photosynthesis" term was also enriched in the DG *vs*. WL (Fig. 2f) and HL *vs*. WL comparisons (Fig. 2g) at both dawn and dusk, indicating HL and DG strongly affected photosynthesis-related changes in transcript abundance independent of dawn or dusk sampling.

To further understand the functional differences of DEGs under different light quality and intensity treatments, we then classified the DEGs into hierarchical categories 'BINs' using MapMan. Based on the DEG number and percentage in each BIN, we found that the percentage of "photosynthesis" class genes was higher in the comparison "DG_Dusk-vs-Dawn" than the other comparisons (Supplementary Fig. S4). And the expression pattern of "photosynthesis" class genes showed down-regulation in samples of DG_Dawn and DG_Dusk (Supplementary Fig. S5). To better understand the gene expression patterns, we mapped the DEGs in the photosynthetic pathway. As shown in Fig. 3, most of the DEGs with functions involved in the light reactions (e.g., LHC-II, PS-II, Cytb6, LHC-I/PS-I and FNR), Calvin cycle (e.g., Rubisco, phosphoglycerate kinase, glyceraldehyde 3-phosphate dehydrogenase and fructose-1,6-bisphosphatase), and photorespiration (e.g., phosphoglycolate phosphatase, glycolate oxidase and glycine decarboxylase) showed down-regulation in DG_Dawn and DG_Dusk. However, glycerate kinases in photorespiration were highly expressed in DG_Dawn and DG_Dusk.

Co-expression network

To determine the relationships among genes responsive to different light quality and light intensity treatments in *K. fedtschenkoi*, we constructed a WGCNA co-expression network using

the DEGs identified from the previous comparisons (Fig. 2). After combining the modules with highly similar expression patterns, a total of 13 co-expression modules were obtained and labelled as different colors (Fig. 4a). The module size ranged from 609 genes (module 'cyan') to 3,047 genes (module 'turquoise'). Among the 13 modules, modules 'blue' and 'salmon' showed low expression at dawn and high expression at dusk under most of the light conditions, except module 'blue' that showed low expression and module 'salmon' that showed high expression under DG. Based on the GO enrichment analysis, module 'blue' was enriched in "oxidoreductase activity" but no significantly enriched GO terms were identified in module 'salmon'. Module 'yellow' showed the opposite expression pattern with modules 'blue' and 'salmon', which showed high expression at dawn and low expression at dusk and was functionally enriched in "transport". The expression of modules 'magenta' and 'purple' were induced at dawn by LL and HL, but the induction of module 'purple' was stronger under HL. These two modules were functionally enriched in "chloroplast" (module 'magenta') and "proteasome complex" (module 'purple'), respectively. Enriched TF analysis indicated that MYB TFs in module 'purple' was stronger than that in module 'magenta' (Supplementary Fig. S6). Two opposite modules 'tan' and 'turquoise' showed low and high expression under DG, which were enriched in "photosynthesis" and "phosphorylation", respectively (Fig. 4 and Supplementary Table S5).

To further understand the response of different pathways in CAM plants under various light quality and light intensity treatments, we extracted sub-networks from the global co-expression network. Here, we selected genes related to CAM, the circadian clock, and stomatal movement [13] as case studies to demonstrate the sub-networks. In order to simplify the sub-network, we set a high threshold of Pearson correlation coefficient (|PCC|>0.95 and $P \leq 0.01$) to show the strong co-expression relationships. The genes involved in CAM, circadian clock, and stomatal movement pathways were highly associated and were co-expressed with numerous transcription factors (TFs) (Fig. 5), implying that the expression of CAM pathway genes may be directly or indirectly regulated by circadian clock TFs. Based on the sub-network, we identified several known and novel TFs that were related with these pathways. For instance, *LHY1* (*Kaladp0066s0115*) was positively co-expressed with *CCA1* (*Kaladp0496s0018*, PCC=0.995), *RVE8* (*Kaladp0577s0020*, PCC=0.993) and *RVE1* (*Kaladp0574s0015*, PCC=0.983); and was negatively co-expressed with *ELF4* (*Kaladp0045s0206*, PCC=-0.978) and *LUX* (*Kaladp0033s0047*, PCC=-0.969). Similarly,

MYB96 (*Kaladp0095s0568*) and *WRKY4* (*Kaladp0096s0082*) were positively co-expressed with *CCA1* and *RVE8* and were negatively co-expressed with *LUX* (Fig. 5 and Supplementary Table S7). In addition, several TFs not previously reported to be associated with CAM were identified in the sub-network, such as *LZF1* (*Kaladp0192s0026*), *SOC1* (*Kaladp0016s0148*), *CDF2* (*Kaladp0009s0042* and *Kaladp0095s0211*), *COL4* (*Kaladp0029s0144*), *ZFP4* (*Kaladp0035s0036*), *ZFP7* (*Kaladp0001s0233*), *SIG1* (*Kaladp0538s0007*), *SIG4* (*Kaladp0515s0145*), and *SIG5* (*Kaladp0055s0328*).

Metabolic changes of *K. fedstchenkoi* response to light quality and light intensity

To explore if this light-responsive expression atlas could provide molecular clues for metabolic changes that are diagnostic of CAM activity, we measured leaf titratable acidity (nocturnal malate and citrate accumulation) and diel turnover of soluble sugars and starch. Nocturnal malic and citric acid accumulation was assessed by the difference in H^+ concentration between dawn and dusk samples (ΔH^+). Under full-spectrum light, ΔH^+ increased with the intensity of light, indicating elevated CAM activity. The levels of nocturnal malate and citrate accumulation for these samples was comparable to that observed under acclimating conditions. In contrast, filtered monochrome light (i.e., BL, RL and FRL) and dark treatment dampened the acid accumulation. RL and FRL treatments, both at photon flux densities of 280 μmol m^{-2} s^{-1}, resulted in very low ΔH^+ values between dawn and dusk samples, indicating reduced CAM activity. Extremely low or inverted dawn/dusk ΔH^+ values were observed for BL and DG plants with higher acid accumulation in dusk samples relative to dawn samples (Fig. 6a, b).

Full-spectrum light treatments of both LL and HL resulted in the elevated quantities of soluble sugars in *Kalanchoë* leaf tissue, with somewhat higher levels observed in dusk samples relative to the corresponding dawn samples. Soluble sugar accumulation in WL was markedly lower than in LL and HL grown plants although control conditions utilized a full-spectrum intermediate light intensity. All colored-light and dark treatments resulted in dramatically reduced soluble sugar contents compared with the LL and HL treatments, which is likely a result of the lower photon flux densities delivered to the plants under these conditions (Fig. 6c). Similar to the trends noted for soluble sugars, high levels of leaf starch accumulation were observed in dusk samples for both LL and HL treatments. WL also resulted in large starch content at dusk. Significant accumulation of starch from dawn to dusk was observed for all full-spectrum samples. Lower starch contents

were observed under all filtered light treatments with no detectable starch in dawn FRL samples or dawn and dusk DG plants. The greatest starch content observed for any filtered-light treatment was seen in dusk samples grown under RL, although these starch levels were still much lower than in plants grown under full-spectrum light (Fig. 6d). Again, these low starch accumulation patterns were likely due to the lower photon flux densities delivered to the plants under these light-filtering conditions.

To understand the potential molecular mechanisms regulating these metabolic phenotypes, we performed a "gene expression – phenotypes" correlation analysis. We selected photosynthetic genes including genes involved in light reactions, Calvin cycle and photorespiration (Fig. 3) and CAM pathway genes including CAM-, circadian-, and stomatal-related genes (Supplementary Table S8) as the candidate genes. Due to the highly positive correlation between titratable acidities (r = 1.00, P < 0.001) and between soluble sugar and starch contents (r = 0.62, P < 0.01) (Supplementary Fig. S7), the correlation patterns of genes with titratable acidities or genes with soluble sugar and starch contents were similar. Notably, different gene sets in the Calvin cycle or CAM pathway were positively correlated with titratable acidities and soluble sugar/starch contents. Most of the circadian-related genes were positively correlated with starch content, but negatively correlated with titratable acidity (Fig. 6e and Supplementary Table S9).

CAM-related genes responsive to light quality and light intensity

To further investigate the CAM-specific response to various light quality and light intensity treatments, the expression patterns of CAM-related genes were analyzed (Fig. 7a). The key genes that are involved in nocturnal CO_2 assimilation and malate storage include *beta-carbonic anhydrase* (*β-CA*), *phosphoenolpyruvate carboxylase* (*PEPC*), *phosphoenolpyruvate carboxylase kinase* (*PPCK*), *NAD(P)-malate dehydrogenase* (*MDH*) and *aluminum-activated malate transporter* (*ALMT*). *β-CA* (*Kaladp0018s0287*) was highly expressed at dawn under high light compared to other light quality and light intensity treatments. *PEPC1* (*Kaladp0095s0055*) showed relatively higher expression levels at dusk than dawn under different light quality and light intensity conditions, yet *PEPC1* expression levels at both dawn and dusk were repressed by filtered monochrome light (i.e., BL, RL and FRL). Similarly, the expression of *PPCK1* (*Kaladp0037s0517*) was also repressed by filtered monochrome light. *MDH2* (*Kaladp0001s0257*) was constitutively induced by increasing light intensity at dawn; but had a peak value under WL at dusk (Fig. 7a).

During daytime, the CO_2 release from malate and refixation is mediated by a series of genes that include *tonoplast dicarboxylate transporter* (*TDT*), *NAD(P)-malic enzyme* [*NAD(P)-ME*], and *pyruvate phosphate dikinase* (*PPDK*). As the first-step transporter for the daytime reactions, *TDT* (*Kaladp0042s0251*) showed an obvious trend of greater transcript abundance at dawn and down-regulation at dusk, and expression at dawn was stronger under BL and RL than under the WL control. In contrast, *NADP-ME* (*Kaladp0092s0166*) showed similar expression patterns with TDT under light intensity treatment, but its expression at dawn were low under BL and RL. *PPDK* (*Kaladp0076s0229*) was increased by light intensity increase and reached the highest level under WL at both dawn and dusk, but was decreased by filtered monochrome light (Fig. 7a). In addition, we compared the light-quality responses of CAM-related genes between *Kalanchoë* and *Arabidopsis*, although the sampling time is different compared to our study – *Arabidopsis* samples were collected at 4h after treatments and our *Kalanchoë* dusk samples were collected at 10h after treatments. All these CAM-related genes showed different expression patterns between *Kalanchoë* and *Arabidopsis* (Fig. 7a).

Based on our light-responsive co-expression network, we extracted the subnetwork of *MDH*. This subnetwork consists of 274 genes, 39 of which are photosynthesis-related and seven of which are TFs (Fig. 7b). Those photosynthesis-related genes in the subnetwork include photosystem I subunits (i.e., PSAL, PSAN, PSAP), photosystem II subunits (i.e., PSB17, PSB28, PSBP, PSBW and PSBX), photosystem I light harvesting complex genes (i.e., LHCA3, LHCA5). Three TFs, *AGL16* (*Kaladp0067s0150*), *SIGF* (*SIG6*, *Kaladp0872s0002*), and *PIF3* (*Kaladp0076s0003*), in this subnetwork are known regulators in photosynthesis or circadian rhythm. In addition, we compared the expression pattern of circadian rhythm-related genes at dawn and dusk under different light conditions. Most of the circadian rhythm-related genes such as *CCA1*, *CRY2*, *ELF3/4*, *HY5*, *PRR7/9*, *RVE1/6/8* and *TOC1* have disordered expression patterns between dawn and dusk under DG (Supplementary Fig. S8), indicating that the circadian clock is likely disrupted under DG.

Photoreceptors and stomatal movement-related genes responsive to light quality in *Kalanchoë*

We tested the expression patterns of photoreceptors in our *Kalanchoë* dataset. As shown in Fig. 8, blue-light receptors *PHOT1* (*Kaladp0032s0316*) and *PHOT2* (*Kaladp0055s0063*) showed up-

regulation under BL at both dawn and dusk. *phyA* (*Kaladp0034s0172*) showed up-regulation under both RL and FRL at dusk. The expression of *PHOT1*, *PHOT2* and *phyB* at dusk (10h after treatments) in *Kalanchoë* showed similar patterns with their orthologs at 4h after treatments in *Arabidopsis* (Fig. 8b). We then constructed the subnetworks of *PHOT1* (*Kaladp0032s0316*) and *phyA* (*Kaladp0034s0172*) based on our co-expression database. Five known regulators involved in light responses were co-expressed with *PHOT1* (*Kaladp0032s0316*), which included *TIC* (*Kaladp0048s0254*) *phyA* (*Kaladp0057s0072*), *FRS8* (*Kaladp0024s0414*), *TOR* (*Kaladp0047s0074*), and *CRY2* (*Kaladp0082s0193*). Similarly, we identified six known light-responsive regulators that were co-expressed with *phyA* (*Kaladp0034s0172*), which included cryptochrome-interacting basic-helix-loop-helix TF CIB1 (*Kaladp0033s0213*), two copies of phytochrome and flowering time regulatory protein PFT1 (*Kaladp0024s0189* and *Kaladp0024s0252*), phyE (Kaladp0053s0072), phytochrome-interacting factor 3 (*PIF3*, *Kaladp0076s0003*), HY1 (*Kaladp0872s0026*). In addition, two CAM-related genes, *PEPCK* (*Kaladp0040s0194*) and *MLS* (*Kaladp0011s1037*) were co-expressed with *phyA* (*Kaladp0034s0172*) (Fig. 8c).

Most of the stomatal movement-related genes were induced by BL, especially at dawn (e.g., *ABI2*, *ALMT9*, *KAT1*, *KAT2* and *QUAC1/ALMT12*) (Fig. 9a). To explore the potential regulatory mechanism, we tested the subnetwork of *ABI2*. Notably, numerous circadian rhythm-related regulators were co-expressed with *ABI2*, which included *CCA1* (*Kaladp0496s0018*), *CCR1* (*Kaladp0018s0148*), *CCR2* (*Kaladp0020s0114*), COL4 (*Kaladp0029s0144*), three copies of *COR27* (*Kaladp0011s1228*, *Kaladp0042s0067* and *Kaladp0089s0010*), *DBB3* (*Kaladp0192s0026*), two copies of *ELF4* (*Kaladp0037s0163* and *Kaladp0045s0206*), *KT4* (*Kaladp0040s0740*), *LHY1* (*Kaladp0066s0115*), *PRR7* (*Kaladp0005s0054*), *RVE1* (*Kaladp0574s0015*), *RVE2* (*Kaladp0262s0019*), and *RVE7* (*Kaladp0262s0013*).

Discussion

As one of the most important environmental factors, light affects plant growth and development, plant physiology and metabolism [27, 28]. Although light quality effects at the metabolic and molecular levels have been studied in several plant species [29-33], genome-wide transcriptomic

studies of the effects of light quality and light intensity on CAM species are lacking. Low-fluorescence red and blue light were shown to modulate the diel metabolic processes in an obligate CAM species *Aechmea* 'Maya' [19]. However, the regulatory mechanisms underpinning the metabolic reprogramming such light-induced metabolic reprogramming in CAM species remains largely unexplored.

In this study, we created a comprehensive, genome-wide, light-responsive gene expression atlas for *K. fedtschenkoi*. We used one plant for each of the three biological replicates for RNA-Seq analysis, which could potentially reduce the false positive rate of differential gene expression in comparison with pooling multiple plants into each biological replicate. The eFP browser provides a useful web interface for easy data access, facilitating comparative and functional genomics research. Furthermore, the RNA-Seq data was analyzed by pairwise comparisons between different light conditions and different time points to identify DEGs in *K. fedtschenkoi*. These DEGs were then subject to clustering and co-expression analyses. A similar approach was effectively utilized to discover the regulatory networks in *Brachypodium distachyon* [26], pigeon pea [34], and chickpea [35]. Combined with functional analysis, such as GO enrichment analysis, we found that the overlapped DEGs at dusk were mainly involved in "carbohydrate metabolism" and "response to endogenous stimulus" processes, consistent with previous studies showing that light quality affects the regulation of endogenous hormone stimulus such as gibberellin, auxins, cytokinins and abscisic acid [3, 36-38].

We found that high level (1,000 μmol m^{-2} s^{-1}) of white light increased expression of three dark CO_2 fixation genes (i.e., *β-CA*, *PPCK*, *PEPC*) and one malate transporter gene (*ALMT*) at dawn (i.e., 2 h before the beginning of light period) in comparison with intermediate level (440 μmol m^{-2} s^{-1}) of white light (Fig. 7a). This up-regulation of genes involved in dark CO_2 fixation and malate import into the vacuoles was consistent with the higher acid accumulation (i.e., dawn-dusk ΔH^+) under high light relative to intermediate light intensity (Fig. 6b). On the other hand, we found that high level of white light repressed expression of several CAM pathway genes (e.g., *PEPC*, *MDH*, *PPDK*, *PPDK-RP*) at dusk (i.e., 2 h before the beginning of dark period) in comparison with intermediate level of white light (Fig. 7a). These results suggest that the high-light treatment was still within the normal physiological range of the plant and not yet necessarily saturating the photosynthetic machinery.

In comparison with white light, blue light repressed the expression of three genes (*β-CA*, *PEPC* and *ALMT*) involved in dark CO_2 fixation and malate transport as well as two genes (*PPDK* and *PPDK-RP*) in the light phase of CAM pathway at dusk (i.e., 2 h before the beginning of dark period) (Fig. 7a). Similarly, red light/far-red light repressed the expression of three genes (*PEPC*, *MDH* and *ALMT*) involved in dark CO_2 fixation and malate transport as well as one gene (*PPDK*) in the light phase of CAM pathway at dusk (i.e., 2 h before the beginning of dark period) (Fig. 7a). This monochrome light-induced gene repression was consistent with the much lower acid accumulation (i.e., dawn-dusk ΔH^+) under blue/red/far-red light conditions (Fig. 6b). These results indicate that blue/red/far-red light treatment interfere with the optimal performance of the CAM pathway in *K. fedtschenkoi*.

K. fedtschenkoi is a model plant species for CAM functional genomics research [13, 15]. Our co-expression analysis highlighted a sub-network of CAM-related regulatory/signaling genes, such as *LHY1*, which was positively co-expressed with *CCA1*, *RVE1* and *RVE8*, but was negatively co-expressed with *ELF4* and *LUX* (Fig. 5). In *Arabidopsis*, ELF4, ELF3, and LUX can form an ELF4-ELF3-LUX protein complex (the evening complex), which is regulated by light and the circadian clock [39]. MYB-related protein CCA1 and LHY1 can form homodimers and regulate the expression of evening-element-containing genes [40]. There is a negative-feedback loop among these transcription factors. EFL4 and LUX are required for the red-light induction of *CCA1* and *LHY1*, whereas CCA1 and LHY1 negatively regulate the expression of ELF4 [41] and LUX [42]. The co-expression relationships of these TFs in *K. fedtschenkoi* reported here indicate that the circadian rhythm regulatory mechanism among these genes is conserved between *K. fedtschenkoi* and *Arabidopsis*.

MYB96, a TF involved in the circadian clock in *Arabidopsis*, was also identified in our sub-network as positively co-expressed with *CCA1* and *RVE8* (Fig. 5). As a key regulator connecting the circadian clock and the environment, *MYB96* is induced by high levels of ABA and can directly bind to the promoter of *TOC1* to active its expression. It is directly regulated by CCA1 through multiple CCA1-binding sites (CBS: AAAATCT) and evening elements (EE: AAATATCT). Interestingly, CCA1 binds to the promoter of MYB96 at dawn, but not at dusk [43, 44]. These findings suggest that our constructed co-expression network is reliable for conserved light-responsive regulator identification.

Several transcription factors with unknown CAM function were identified in the *K. fedtschenkoi* co-expression network. These transcription factors potentially represent novel regulatory mechanisms. As shown in Fig. 5, *WRKY4* was positively co-expressed with *CCA1* and *RVE8*, but negatively co-expressed with *LUX*. Although there is no direct evidence for the involvement of *WRKY4* gene in circadian regulation, its homolog in tomato showed up-regulation at eight hours after dawn, presumptive dusk, and four hours after dusk in comparison with presumptive dawn under long-day conditions [45]. Furthermore, some abscisic acid and light signaling-related genes were identified in our CAM gene-enriched sub-network (e.g., *ZINC FINGER PROTEINS* (*ZFP4* and *ZFP7*), *SIGMA FACTORS* (*SIG1*, *SIG4* and *SIG5*) and B-BOX protein *STH2/BBX21*). In *Arabidopsis*, homologs of *ZFP* are involved in light-responsive pathways, where *ZFP3* can interfere with ABA and light signal in plant development and seed germination [46], while *ZFP1* is expressed in a pathway that is downstream of photomorphogenesis [47]. In prokaryotes, sigma factors are well known for their participation in the control of RNA polymerase activity. The phosphorylation of SIG1 selectively inhibits the expression of gene encoding photosystem I [48]. *SIG1* is strongly induced by red and blue light, but *SIG5* is only induced by blue light under mediating of *CRY1* and *CRY2* [49, 50]. As a key component involved in the COP1-HY5 hub, *STH2/BBX21* is controlled by COP1 through its E3 ubiquitin ligase activity in darkness and promotes photomorphogenesis by activating *HY5* in the light [51]. Our results provide a powerful resource for light-responsive regulator identification.

In addition, the subnetworks of specific genes provide insight on the molecular mechanisms underpinning the different light-induced metabolic phenotypes. For example, the subnetwork of *MDH* (*Kaladp0001s0257*) shows an enrichment of photosynthetic genes (Fig. 7b). The TFs (*AGL16*, *SIGF* and *PIF3*) in this subnetwork might be the dominant regulators of this subnetwork. The ortholog of *AGL16* in *Arabidopsis* is targeted for sequence-specific degradation by *miR824*, expression of a *miR824*-resistant *AGL16* increased the incidence of stomata in high-order complexes in transgenic plants [52]. *SIGF* (*SIG6*) encodes a general sigma factor in chloroplasts. Expression chimeric sigma factor genes in *Arabidopsis sigf* mutant affects the expression of numerous plastid genes [53]. *PIF3* is a key basic helix-loop-helix transcription factor of *Arabidopsis* that negatively regulates light responses, repressing chlorophyll biosynthesis, photosynthesis, and photomorphogenesis in the dark. PIF3 and HDA15 are dissociated from the

target genes upon exposure to red light. PIF3 associates with HDA15 to repress chlorophyll biosynthetic and photosynthetic genes in etiolated seedlings [54].

In the subnetwork of stomatal movement-related gene *ABI2*, 16 known circadian rhythm-related regulators were identified (Fig. 9b), which included synergistically functional *CCA1* and *LHY1*, and their downstream gene *PPR7* [55]. *RVE1* is homologous to *CCA1* and *LHY1*, but inactivation of *RVE1* does not affect circadian rhythmicity, but instead causes a growth phenotype [56]. However, another two *RVE* genes (*RVE2* and *RVE7*) in this subnetwork are directly involved in circadian regulation. RVE2 (CIR1) is possibly part of a regulatory feedback loop that controls a subset of the circadian outputs and modulates the central oscillator [57]. RVE7 (EPR1) is a component of a slave oscillator that contributes to the refinement of output pathways, ultimately mediating the correct oscillatory behavior of target genes [58]. Our dataset provides a additional insights into the photo-responsiveness of CAM plants. In addition, the "gene expression – metabolic phenotypes" correlation analysis identified a series of positively and negatively correlated genes associated with photosynthesis and CAM pathways, which provided molecular clues for understanding the physiological changes which accompany light quality and light intensity responses in *Kalanchoë*.

In conclusion, the comprehensive light-responsive gene expression atlas of *K. fedtschenkoi* provides an extremely useful genomics resource for investigating the molecular mechanisms underlying responses to light quantity and quality in CAM plants. The genome-wide co-expression network lays a solid foundation for discovering novel gene function in CAM plants. Furthermore, the results from our comparative analyses of gene expression and acid accumulation between different light treatments support our hypothesis that both light intensity and light quality can affect the expression of CAM-related genes in *K. fedtschenkoi*.

Methods

Plant Material and Experimental Treatments

Kalanchoë fedtschenkoi (ORNL diploid accession M2) plants originally started from meristem cuttings were grown in soil for 4 weeks in a Percival Model AR-75L2 growth chamber on a 12-h

light (26°C)/12-h dark (18°C) cycle at a photon flux density of 280 μmol m^{-2} s^{-1}. For acclimation prior to light quality or light intensity treatments, plants were placed for at least 2 d in the growth chamber on a 12-h light (26°C)/ 12-h dark (18°C) cycle at a photon flux density of 440 μmol m^{-2} s^{-1}. Light quality treatments consisted of then growing plants under blue light (blue light, 270 μmol m^{-2} s^{-1}) provided by a dark blue gel filter (#119), red light (red light, 280 μmol m^{-2} s^{-1}) provided by primary red gel filter (#106), far-red light (far-red light, 280 μmol m^{-2} s^{-1}) provided by a medium red Roscolux filter (Barndoor Lighting Outfitters, Inc., North Branford, CT), or constant darkness. For all treatments, except constant darkness, a 12-h light (26°C)/12-h dark (18°C) cycle was used. Light intensity treatments consisted of growing the plants under dark growth (dark grown), low light (low light, 150 μmol m^{-2} s^{-1}) or high light (high light, 1,000 μmol m^{-2} s^{-1}) with a 12-h light (26°C) / 12-h dark (18°C) cycle, respectively. All plants used for light quality and light intensity experiments were grown under the indicated conditions for 48 h prior to any tissue collection. All photon flux density measurements described above were taken at leaf level of the apical meristem as these leaves were closest to the light source.

Tissue Collection and RNA Isolation

Fully-expanded leaves (i.e., leaf pair 4-5 counting from the top of the plants) were collected from three biological replicates of plants (each biological replicate was one independent plant) grown under each of the light quality and light intensity experimental conditions. Each sample was collected at both dawn (2 h before the starting of lighting period) and dusk (2 h before the dark period) time points, wrapped in aluminum foil, immediately frozen in liquid nitrogen, and stored at -80°C until processing. For RNA isolation, frozen leaf tissue samples were ground to a fine powder under liquid nitrogen with a mortar and pestle. Isolation of total RNA then proceeded by using the QIAGEN RNeasy® Plant Mini Kit (Cat No. 74904, Qiagen Inc., Valencia, CA, USA) with the following modifications: 600 mg of frozen ground tissue from each sample was mixed thoroughly with 2.57 ml of Fruit Mate™ (TaKaRa Bio USA, Inc., Mountain View, CA). The resulting suspension was centrifuged at 14,000 × *g* at 4°C for 5 min. The supernatant was then mixed with 1.8 ml of QIAGEN buffer RLT/2-mercaptoethanol mix. This solution was centrifuged at 14,000 × *g* at 25°C for 1 min. The supernatant was then mixed with 0.5 volumes of 100% ethanol and remaining steps were performed according to kit instructions. On-column DNase

digestions were performed for all samples according to RNeasy® kit instructions with the QIAGEN RNase-Free DNase Set (Cat No. 79254). Final RNA elution was performed with 50 µl of RNase free water which was run through the column twice. RNA purity and approximate quantity was assessed with a Thermo Scientific™ NanoDrop 2000c spectrophotometer and precise quantity assessed with Quant-iT™ RiboGreen® fluorescence (Thermo Scientific, Rockford, IL). RNA integrity was evaluated on a 1% (w/v) agarose gel using 300 ng RNA.

RNA-Seq Libraries construction and RNA-seq

A total of 42 libraries (7 light conditions × 2 time points × 3 biological replicates) were constructed and performed RNA-seq independently. The total RNA samples were sequenced in the Department of Energy Joint Genome Institute (Walnut Creek, CA). Briefly, the integrity and concentration of the RNA preparations were checked initially using Nano-Drop ND-1000 (Nano-Drop Technologies) and then by BioAnalyzer (Agilent Technologies). Plate-based RNA sample prep was performed on the PerkinElmer Sciclone NGS robotic liquid handling system using Illumina's TruSeq Stranded mRNA HT sample prep kit utilizing poly-A selection of mRNA with the following conditions: total RNA starting material was 1 µg per sample and 8 cycles of PCR was used for library amplification. The prepared libraries were then quantified by qPCR using the Kapa SYBR Fast Illumina Library Quantification Kit (Kapa Biosystems) and run on a Roche LightCycler 480 real-time PCR instrument. The quantified libraries were then prepared utilizing a TruSeq paired-end cluster kit, v4, and Illumina's cBot instrument to generate a clustered flowcell for sequencing. Sequencing of the flowcell was performed on the Illumina HiSeq2500 platform using HiSeq TruSeq SBS sequencing kits, v4, following a 2 x 150 indexed run recipe.

Reads mapping and data analysis

After filtering out low-quality reads, RNA-seq reads from each library were aligned to the *Kalanchoë fedtschenkoi* reference genome [13] using GSNAP ~~(~~v2018-07-04 (GSNAP, RRID:SCR_005483)~~)~~ [59]. FeatureCounts function of Subread ~~(~~v1.6.1 (Subread, RRID:SCR_009803)~~)~~ [60] was used to generate raw gene counts and only reads that mapped uniquely to one locus were counted. Gene expression was estimated as transcripts per million (TPM) [61]. DESeq2 ~~(~~v1.2.10 (DESeq2, RRID:SCR_015687)~~)~~ [62] was subsequently used to determine which genes were differentially expressed between pairs of conditions. The parameters

used to "call a gene" between conditions was determined at a false discovery rate (FDR) adjusted *P*-value≤0.05. DEGs were classified into hierarchical categories 'BINs' using MapMan (version 3.6.0RC1 (MapMan, RRID:SCR_003543)) [63]. Gene Ontology (GO) enrichment analysis was applied to predict gene function and calculate the functional category using BiNGO (BiNGO: A Biological Networks Gene Ontology tool, RRID:SCR_005736) [64]. Heatmap and bubble plots were generated by the R package ggplot2. All tools were run with default parameters.

Kalanchoë light-responsive eFP browser

TPM-normalized values of the RNA-Seq data sets were uploaded into the *Kalanchoë* eFP browser of the Bio-Analytic Resource (BAR). Representative images of *Kalanchoë* leaf under different light condition were created and an XML file was generated to power a view within the *Kalanchoë* eFP browser at http://bar.utoronto.ca/efp_kalanchoe/cgi-bin/efpWeb.cgi.

Co-expression analysis

For co-expression analysis, the $\log_2$ normalized TPM values of all the samples were used to construct a weighted gene co-expression network using the R package WGCNA [65].

Titratable acidity assays of leaf tissue

For titratable acidity experiments, approximately 0.5 g of fine frozen, ground leaf tissue was added to 10.0 ml of 50% (v/v) methanol and mixed well. This suspension was then boiled at 80°C for 10 min. Additional 50% (v/v) methanol was added after boiling to any samples that showed volume loss. The boiled samples were then centrifuged at 2,000 × *g* for 10 min at room temperature. Supernatants were titrated with 10 mM KOH to pH = 7.0 and 8.4 corresponding to malate and citrate, respectively. Leaf titratable acidities were expressed as μmol H^+ g^{-1} fresh weight.

Carbohydrate analysis of leaf tissue

For soluble sugar and starch assays, ground leaf tissue was boiled in 10.0 ml of 50% (v/v) methanol at 80°C for 30 minutes. Sample volumes were adjusted back to original volumes with 50% (v/v) methanol. Samples were then centrifuged at 2,000 × *g* for 10 min at room temperature. The resulting supernatants were reserved for soluble sugar analysis. For starch extraction, tissue pellets were washed twice with 10 ml of Nanopure water with centrifugation at 2,000 × *g* after each wash. 1.2 ml of acetate buffer (prepared from 86 ml of 0.1 M sodium acetate and 114 ml of

0.1 N acetic acid, pH 4.5) was added to the washed pellet and resuspended by vortexing. Then, 0.2 ml of starch digestion solution (300 units α-amyloglucosidase and 25 units α-amylase prepared in 20 ml acetate buffer) was added and the mixture incubated overnight at 45°C. Starch-digested samples were centrifuged at 2,000 × *g* for 10 min at room temperature and the supernatant used for starch analysis. Colorimetric assays for determination of starch and soluble sugar content were performed as described [66].

Availability of supporting data

All raw short reads are available in the NCBI SRA database (SRA accessions: SRP146136, SRP146139, SRP146175, SRP146204, SRP146205, SRP146213, SRP148019 – SRP148030, SRP148037 – SRP148060) (Supplementary Table S1). Supporting data is also available via the *Kalanchoë* eFP Browser [67] and via the GigaScience database GigaDB [68].

Abbreviations

BAR: Bio-Analytic Resource; CAM: Crassulacean acid metabolism; CBS: CCA1 binding sites; DEG: differentially expressed gene; EE: evening elements; FDR: false discovery rate; GO: gene ontology; MDS: multidimensional scaling; PC: principal component; PCC: Pearson correlation coefficient; PPFD: photosynthetic photon flux density; RNA-Seq: RNA-Sequencing; Rubisco: ribulose-1,5-bisphosphate carboxylase/oxygenase; TF: transcription factor; TPM: transcripts per million; WGCNA: weighted gene co-expression network analysis; WUE: water-use efficiency.

Competing interests

The authors declare that they have no competing interests.

Funding

This research was supported by the U.S. Department of Energy, Office of Science, Genomic Science Program under Award Number DE-SC0008834. Additional support was provided by the

Community Science Program (project 503025) at the Department of Energy Joint Genome Institute and the DOE Center for Bioenergy Innovation at the Oak Ridge National Laboratory.

Author contributions

X.Y., J.Z. and R.H. conceived and designed the research. R.H., T.G., P.Y., and J.C.C. performed the experiments. J.Z., A.S., A.L., M.W., D.L., V.N., J.S. and A.M.B. analyzed the data. J.Z., A.P. and N.J.P. provided eFP browser instance. J.Z. and R.H. drafted the manuscript. X.Y., J.G.C., W.M., J.C.C., and G.A.T. revised the manuscript. All authors read and approved the manuscript.

Acknowledgements

This manuscript has been authored by UT-Battelle, LLC under Contract No. DE-AC05-00OR22725 with the U.S. Department of Energy. The work conducted by the U.S. Department of Energy Joint Genome Institute is supported by the Office of Science of the U.S. Department of Energy under Contract No. DE-AC02-05CH11231. This research used resources of the Compute and Data Environment for Science (CADES) and the Oak Ridge Leadership Computing Facility at the Oak Ridge National Laboratory.

References

1. Mølmann JA, Junttila O, Johnsen Ø and Olsen JE. Effects of red, far- red and blue light in maintaining growth in latitudinal populations of Norway spruce (Picea abies). Plant, Cell & Environment. 2006;29 2:166-72.
2. Quail PH. Phytochrome photosensory signalling networks. Nature Reviews Molecular Cell Biology. 2002;3 2:85.
3. OuYang F, Mao J-F, Wang J, Zhang S and Li Y. Transcriptome analysis reveals that red and blue light regulate growth and phytohormone metabolism in Norway spruce [Picea abies (L.) Karst.]. PloS One. 2015;10 8:e0127896.
4. Briggs WR and Olney MA. Photoreceptors in plant photomorphogenesis to date. Five phytochromes, two cryptochromes, one phototropin, and one superchrome. Plant Physiology. 2001;125 1:85-8.
5. Lin C and Shalitin D. Cryptochrome structure and signal transduction. Annual Review of Plant Biology. 2003;54 1:469-96.
6. Briggs W, Beck C, Cashmore A, Christie J, Hughes J, Jarillo J, et al. The phototropin family of photoreceptors. Plant Cell. 2001;13 5:993-7.
7. Nagy F and Schäfer E. Phytochromes control photomorphogenesis by differentially regulated, interacting signaling pathways in higher plants. Annual Review of Plant Biology. 2002;53 1:329-55.
8. Fan X-X, Xu Z-G, Liu X-Y, Tang C-M, Wang L-W and Han X-l. Effects of light intensity on the growth and leaf development of young tomato plants grown under a combination of red and blue light. Scientia Horticulturae. 2013;153:50-5.
9. Rossel JB, Wilson IW and Pogson BJ. Global changes in gene expression in response to high light in *Arabidopsis*. Plant Physiology. 2002;130 3:1109-20.
10. Zavala J and Ravetta D. Allocation of photoassimilates to biomass, resin and carbohydrates in *Grindelia chiloensis* as affected by light intensity. Field Crops Research. 2001;69 2:143-9.
11. Yang X, Cushman JC, Borland AM, Edwards EJ, Wullschleger SD, Tuskan GA, et al. A roadmap for research on crassulacean acid metabolism (CAM) to enhance sustainable food and bioenergy production in a hotter, drier world. New Phytologist. 2015;207 3:491-504.
12. Borland AM, Hartwell J, Weston DJ, Schlauch KA, Tschaplinski TJ, Tuskan GA, et al. Engineering crassulacean acid metabolism to improve water-use efficiency. Trends in Plant Science. 2014;19 5:327-38.
13. Yang X, Hu R, Yin H, Jenkins J, Shu S, Tang H, et al. The *Kalanchoë* genome provides insights into convergent evolution and building blocks of crassulacean acid metabolism. Nature Communications. 2017;8 1:1899.
14. Borland AM, Wullschleger SD, Weston DJ, Hartwell J, Tuskan GA, Yang X, et al. Climate-resilient agroforestry: physiological responses to climate change and engineering of crassulacean acid metabolism (CAM) as a mitigation strategy. Plant, Cell & Environment. 2015;38 9:1833-49.
15. Hartwell J, Dever LV and Boxall SF. Emerging model systems for functional genomics analysis of Crassulacean acid metabolism. Current Opinion in Plant Biology. 2016;31:100-8.
16. Dodd AN, Borland AM, Haslam RP, Griffiths H and Maxwell K. Crassulacean acid metabolism: plastic, fantastic. Journal of experimental botany. 2002;53 369:569-80.
17. Grams TE and Thiel S. High light- induced switch from C 3- photosynthesis to Crassulacean acid metabolism is mediated by UV- A/blue light. Journal of Experimental Botany. 2002;53 373:1475-83.
18. Ceusters J, Borland AM, Godts C, Londers E, Croonenborghs S, Van Goethem D, et al. Crassulacean acid metabolism under severe light limitation: a matter of plasticity in the shadows? Journal of Experimental Botany. 2010;62 1:283-91.

19. Ceusters J, Borland AM, Taybi T, Frans M, Godts C and De Proft MP. Light quality modulates metabolic synchronization over the diel phases of crassulacean acid metabolism. Journal of Experimental Botany. 2014;65 13:3705-14.
20. Kornas A, Fischer-Schliebs E, Lüttge U and Miszalski Z. Adaptation of the obligate CAM plant *Clusia alata* to light stress: metabolic responses. Journal of Plant Physiology. 2009;166 17:1914-22.
21. Miszalski Z, Kornas A, Rozpądek P, Fischer-Schliebs E and Lüttge U. Independent fluctuations of malate and citrate in the CAM species *Clusia hilariana* Schltdl. under low light and high light in relation to photoprotection. Journal of Plant Physiology. 2013;170 5:453-8.
22. Klepikova AV, Kasianov AS, Gerasimov ES, Logacheva MD and Penin AA. A high resolution map of the Arabidopsis thaliana developmental transcriptome based on RNA-seq profiling. Plant Journal. 2016;88 6:1058-70. doi:doi:10.1111/tpj.13312.
23. Benedito VA, Torres-Jerez I, Murray JD, Andriankaja A, Allen S, Kakar K, et al. A gene expression atlas of the model legume *Medicago truncatula*. Plant Journal. 2008;55 3:504-13. doi:doi:10.1111/j.1365-313X.2008.03519.x.
24. Matas AJ, Yeats TH, Buda GJ, Zheng Y, Chatterjee S, Tohge T, et al. Tissue- and cell-type specific transcriptome profiling of expanding tomato fruit provides insights into metabolic and regulatory specialization and cuticle formation. Plant Cell. 2011;23 11:3893-910. doi:10.1105/tpc.111.091173 %J The Plant Cell.
25. Ramírez-González RH, Borrill P, Lang D, Harrington SA, Brinton J, Venturini L, et al. The transcriptional landscape of polyploid wheat. Science. 2018;361 6403:eaar6089. doi:10.1126/science.aar6089 %J Science.
26. Sibout R, Proost S, Hansen BO, Vaid N, Giorgi FM, Ho-Yue-Kuang S, et al. Expression atlas and comparative coexpression network analyses reveal important genes involved in the formation of lignified cell wall in *Brachypodium distachyon*. New Phytologist. 2017;215 3:1009-25. doi:10.1111/nph.14635.
27. Li Q and Kubota C. Effects of supplemental light quality on growth and phytochemicals of baby leaf lettuce. Environmental and Experimental Botany. 2009;67 1:59-64.
28. Fukuda N, Fujita M, Ohta Y, Sase S, Nishimura S and Ezura H. Directional blue light irradiation triggers epidermal cell elongation of abaxial side resulting in inhibition of leaf epinasty in geranium under red light condition. Scientia Horticulturae. 2008;115 2:176-82.
29. Kitazaki K, Fukushima A, Nakabayashi R, Okazaki Y, Kobayashi M, Mori T, et al. Metabolic reprogramming in leaf lettuce grown under different light quality and intensity conditions using narrow-band LEDs. Scientific Reports. 2018;8 1:7914.
30. Li C-X, Xu Z-G, Dong R-Q, Chang S-X, Wang L-Z, Khalil-Ur-Rehman M, et al. An RNA-seq analysis of grape plantlets grown in vitro reveals different responses to blue, green, red LED light, and white fluorescent light. Frontiers in Plant Science. 2017;8:78.
31. Tardu M, Dikbas UM, Baris I, Kavakli IHJF and genomics i. RNA-seq analysis of the transcriptional response to blue and red light in the extremophilic red alga, *Cyanidioschyzon merolae*. Functional & Integrative Genomics. 2016;16 6:657-69.
32. Hao X, Li L, Hu Y, Zhou C, Wang X, Wang L, et al. Transcriptomic analysis of the effects of three different light treatments on the biosynthesis of characteristic compounds in the tea plant by RNA-Seq. Tree Genetics & Genomes. 2016;12 6:118.
33. Sellaro R, Hoecker U, Yanovsky M, Chory J and Casal JJ. Synergism of red and blue light in the control of Arabidopsis gene expression and development. Current Biology. 2009;19 14:1216-20.
34. Pazhamala LT, Purohit S, Saxena RK, Garg V, Krishnamurthy L, Verdier J, et al. Gene expression atlas of pigeonpea and its application to gain insights into genes associated with pollen fertility implicated in seed formation. Journal of Experimental Botany. 2017;68 8:2037-54.
35. Kudapa H, Garg V, Chitikineni A and Varshney RK. The RNA-Seq-based high resolution gene expression atlas of chickpea (*Cicer arietinum* L.) reveals dynamic spatio-temporal changes

associated with growth and development. Plant, Cell & Environment. 2018;41 9:2209-25. doi:doi:10.1111/pce.13210.

36. Kurepin LV, Emery RN, Pharis RP and Reid DM. The interaction of light quality and irradiance with gibberellins, cytokinins and auxin in regulating growth of *Helianthus annuus* hypocotyls. Plant, Cell & Environment. 2007;30 2:147-55.
37. Zhang Z, Ji R, Li H, Zhao T, Liu J, Lin C, et al. CONSTANS-LIKE 7 (COL7) is involved in phytochrome B (phyB)-mediated light-quality regulation of auxin homeostasis. Molecular Plant. 2014;7 9:1429-40.
38. Gubler F, Hughes T, Waterhouse P and Jacobsen J. Regulation of dormancy in barley by blue light and after-ripening: effects on abscisic acid and gibberellin metabolism. Plant Physiology. 2008;147 2:886-96.
39. Nusinow DA, Helfer A, Hamilton EE, King JJ, Imaizumi T, Schultz TF, et al. The ELF4-ELF3-LUX complex links the circadian clock to diurnal control of hypocotyl growth. Nature. 2011;475 7356:398-402. doi:10.1038/nature10182.
40. Lu SX, Knowles SM, Andronis C, Ong MS and Tobin EM. CIRCADIAN CLOCK ASSOCIATED1 and LATE ELONGATED HYPOCOTYL function synergistically in the circadian clock of *Arabidopsis*. Plant Physiology. 2009;150 2:834-43. doi:10.1104/pp.108.133272.
41. Kikis EA, Khanna R and Quail PH. ELF4 is a phytochrome-regulated component of a negative-feedback loop involving the central oscillator components CCA1 and LHY. Plant Journal. 2005;44 2:300-13. doi:10.1111/j.1365-313X.2005.02531.x.
42. Hazen SP, Schultz TF, Pruneda-Paz JL, Borevitz JO, Ecker JR and Kay SA. LUX ARRHYTHMO encodes a Myb domain protein essential for circadian rhythms. Proc Natl Acad Sci U S A. 2005;102 29:10387-92. doi:10.1073/pnas.0503029102.
43. Lee HG, Mas P and Seo PJ. MYB96 shapes the circadian gating of ABA signaling in *Arabidopsis*. Scientific Reports. 2016;6:17754. doi:10.1038/srep17754.
44. Muchapirei CI, Valentine S-L and Roden LC. Plant circadian networks and responses to the environment. Functional Plant Biology. 2018;45 4:393-9.
45. Facella P, Lopez L, Carbone F, Galbraith DW, Giuliano G and Perrotta G. Diurnal and Circadian Rhythms in the Tomato Transcriptome and Their Modulation by Cryptochrome Photoreceptors. PLOS ONE. 2008;3 7:e2798. doi:10.1371/journal.pone.0002798.
46. Joseph MP, Papdi C, Kozma-Bognar L, Nagy I, Lopez-Carbonell M, Rigo G, et al. The *Arabidopsis* ZINC FINGER PROTEIN3 interferes with abscisic acid and light signaling in seed germination and plant development. Plant Physiology. 2014;165 3:1203-20. doi:10.1104/pp.113.234294.
47. Chrispeels HE, Oettinger H, Janvier N and Tague BW. *AtZFP1*, encoding *Arabidopsis thaliana* C2H2 zinc-finger protein 1, is expressed downstream of photomorphogenic activation. Plant Molecular Biology. 2000;42 2:279-90.
48. Shimizu M, Kato H, Ogawa T, Kurachi A, Nakagawa Y and Kobayashi H. Sigma factor phosphorylation in the photosynthetic control of photosystem stoichiometry. Proc Natl Acad Sci U S A. 2010;107 23:10760-4. doi:10.1073/pnas.0911692107.
49. Tsunoyama Y, Morikawa K, Shiina T and Toyoshima Y. Blue light specific and differential expression of a plastid σ factor, Sig5 in *Arabidopsis thaliana*. FEBS Letters. 2002;516 1:225-8. doi:10.1016/S0014-5793(02)02538-3.
50. Onda Y, Yagi Y, Saito Y, Takenaka N and Toyoshima Y. Light induction of *Arabidopsis SIG1* and *SIG5* transcripts in mature leaves: differential roles of cryptochrome 1 and cryptochrome 2 and dual function of SIG5 in the recognition of plastid promoters. Plant Journal. 2008;55 6:968-78. doi:10.1111/j.1365-313X.2008.03567.x.
51. Xu D, Jiang Y, Li J, Lin F, Holm M and Deng XW. BBX21, an *Arabidopsis* B-box protein, directly activates HY5 and is targeted by COP1 for 26S proteasome-mediated degradation. Proceedings of the National Academy of Sciences of the United States of America. 2016;113 27:7655-60. doi:10.1073/pnas.1607687113.

52. Kutter C, Schob H, Stadler M, Meins F, Jr. and Si-Ammour A. MicroRNA-mediated regulation of stomatal development in Arabidopsis. Plant Cell. 2007;19 8:2417-29. doi:10.1105/tpc.107.050377.
53. Schweer J, Geimer S, Meurer J and Link G. Arabidopsis mutants carrying chimeric sigma factor genes reveal regulatory determinants for plastid gene expression. Plant Cell Physiol. 2009;50 7:1382-6. doi:10.1093/pcp/pcp069.
54. Liu X, Chen CY, Wang KC, Luo M, Tai R, Yuan L, et al. PHYTOCHROME INTERACTING FACTOR3 associates with the histone deacetylase HDA15 in repression of chlorophyll biosynthesis and photosynthesis in etiolated Arabidopsis seedlings. Plant Cell. 2013;25 4:1258-73. doi:10.1105/tpc.113.109710.
55. Nagel DH, Doherty CJ, Pruneda-Paz JL, Schmitz RJ, Ecker JR and Kay SA. Genome-wide identification of CCA1 targets uncovers an expanded clock network in Arabidopsis. Proc Natl Acad Sci U S A. 2015;112 34:E4802-10. doi:10.1073/pnas.1513609112.
56. Rawat R, Schwartz J, Jones MA, Sairanen I, Cheng Y, Andersson CR, et al. REVEILLE1, a Myb-like transcription factor, integrates the circadian clock and auxin pathways. Proc Natl Acad Sci U S A. 2009;106 39:16883-8. doi:10.1073/pnas.0813035106.
57. Zhang X, Chen Y, Wang ZY, Chen Z, Gu H and Qu LJ. Constitutive expression of CIR1 (RVE2) affects several circadian- regulated processes and seed germination in Arabidopsis. The Plant Journal. 2007;51 3:512-25.
58. Kuno N, Moller SG, Shinomura T, Xu XM, Chua NH and Furuya M. The novel MYB protein EARLY-PHYTOCHROME-RESPONSIVE1 is a component of a slave circadian oscillator in Arabidopsis. Plant Cell. 2003;15 10:2476-88. doi:DOI 10.1105/tpc.014217.
59. Wu TD and Nacu S. Fast and SNP-tolerant detection of complex variants and splicing in short reads. Bioinformatics. 2010;26 7:873-81.
60. Liao Y, Smyth GK and Shi W. featureCounts: an efficient general purpose program for assigning sequence reads to genomic features. Bioinformatics. 2013;30 7:923-30.
61. Li B and Dewey CN. RSEM: accurate transcript quantification from RNA-Seq data with or without a reference genome. BMC Bioinformatics. 2011;12 1:323.
62. Love MI, Huber W and Anders S. Moderated estimation of fold change and dispersion for RNA-seq data with DESeq2. Genome Biology. 2014;15 12:550.
63. Thimm O, Bläsing O, Gibon Y, Nagel A, Meyer S, Krüger P, et al. MAPMAN: a user- driven tool to display genomics data sets onto diagrams of metabolic pathways and other biological processes. The Plant Journal. 2004;37 6:914-39.
64. Maere S, Heymans K and Kuiper M. BiNGO: a Cytoscape plugin to assess overrepresentation of gene ontology categories in biological networks. Bioinformatics. 2005;21 16:3448-9.
65. Langfelder P and Horvath S. WGCNA: an R package for weighted correlation network analysis. BMC Bioinformatics. 2008;9 1:559.
66. Dubois M, Gilles KA, Hamilton JK, Rebers Pt and Smith F. Colorimetric method for determination of sugars and related substances. Analytical chemistry. 1956;28 3:350-6.
67. The Kalanchoe eFP Browser. http://bar.utoronto.ca/efp_kalanchoe/cgi-bin/efpWeb.cgi. Accessed 11 February 2020.
68. Zhang J, Hu R, Garcia T, Lipzen A, Wang M, Yerramsetty P et al. Supporting data for "Light-responsive expression atlas reveals the effects of light quality and intensity in Kalanchoë fedtschenkoi, a plant with crassulacean acid metabolism" GigaScience Database 2020. http://dx.doi.org/10.5524/100706

Figure Legends

Fig. 1. *Kalanchoë* light-responsive eFP browser.

(a) View of the eFP browser including the RNA-Seq data set described in this study. Expression values in the samples are indicated by a color gradient, where yellow indicates low expression and red indicates high expression. The legend describing the color gradient and expression values is shown in the bottom left corner. *CCA1* gene Kaladp0496s0018 is used as an example. The leaf samples were collected at dawn (i.e., 2 h before the beginning of light period) and dusk (i.e., 2 h before the beginning of dark period) under control condition (WL, white light) and various light quality conditions (BL, blue light; RL, red light; and FRL, far-red light) and light intensity conditions (DG, dark grown; LL, low-light intensity; and HL, high-light intensity).

(b) *Kalanchoë* genes *CCA1* (Kaladp0496s0018) and *HY5* (Kaladp0060s0460) displayed in a comparative view of the expression level extracted from the eFP browser. The legend describing the color gradient and log_2 ratio is shown in the bottom left corner.

Fig. 2. Transcriptomic comparison of *Kalanchoë fedtschenkoi* under various light quality and light intensity conditions.

(a) Schematic of sample collection and comparisons. The leaf samples were collected at dawn (2-hour before light period) and dusk (2-hour before dark period) under control condition (WL, white light) and various light quality conditions (BL, blue light; RL, red light; and FRL, far-red light) and light intensity conditions (DG, dark grown; LL, low-light intensity; and HL, high-light intensity). For differentially expressed genes (DEGs) identification, the comparisons were classified into time comparisons (Dusk-vs-Dawn) and light condition comparisons (BL/RL/FRL-vs-WL for light quality comparisons and DG/LL/HL-vs-WL for light intensity comparisons).

(b) Principal component analysis (PCA) of the 14 groups of transcriptome data.

(c-e) Statistic of DEGs between dawn and dusk in time comparisons **(c)** and among various light quality **(d)** or light intensity **(e)** in light condition comparisons.

(f) Venn diagrams represent DEGs overlapped in different comparisons. ① dusk-vs-dawn under different light quality; ② dusk-vs-dawn under different light intensity; ③ different light quality (BL/RL/FRL-vs-WL) at dawn; ④ different light quality at dusk; ⑤ different light intensity (DG/LL/HL-vs-WL) at dawn; ⑥ different light intensity at dusk.

(g) Gene ontology (GO) enrichment of common DEGs shared by different comparisons in (f) Venn diagrams (DEGs shared by all the comparisons in a given set) or specific DEGs (genes only differentially expressed in one of the overlapped comparisons). Biological process (BP). Detailed enrichment of molecular function (MF) and cellular component (CC) was shown in Supplementary Figure S3. GOslim terms were shown in here.

Fig. 3. Schematic representation of gene expression patterns in the photosynthesis pathway.

The figure was modified based on the MapMan visualization platform. Line plots represent Z-score normalized (*y*-axis) expression patterns of DEGs under various light quality and light intensity conditions. The *x*-axis from left to right represents the samples of WL_Dawn, WL_Dusk, BL_Dawn, BL_Dusk, RL_Dawn, RL_Dusk, FRL_Dawn, FRL_Dusk, DG_Dawn, DG_Dusk, LL_Dawn, LL_Dusk, HL_Dawn and HL_Dusk. Detailed gene list and expression data were shown in Supplementary Table S4.

Fig. 4. Weighted Gene Co-expression Network Analysis (WGCNA) of DEGs in *Kalanchoë fedtschenkoi* under various light quality and light intensity conditions.

(a) Cluster dendrogram of DEGs in *Kalanchoë fedtschenkoi* under various light quality and light intensity conditions. Different colors in merged modules column represent 13 different modules (MEs).

(b) Z-score normalized expression patterns of DEGs in different modules.

(c) GO enrichment analysis of DEGs in different MEs. Node color represents -$\log_{10}$ transformed FDR corrected *P* value. Node size represents rich factor. Full list of enriched GO terms was shown in Supplementary Table S5.

Fig. 5. Sub-network of CAM, circadian clock and stomatal movement.

Red nodes represent transcription factors (TFs). Triangle, arrowhead and rounded-rectangle shapes of nodes represent CAM-, circadian clock- and stomatal movement-related genes, respectively. Red and blue edges represent positive correlation (PCC>0.95 and $p \leq 0.01$) and negative correlation (PCC<-0.95 and $p \leq 0.01$), respectively.

Fig. 6. Physiological changes of *Kalanchoë fedtschenkoi* under various light quality and light intensity conditions.

(a) Nocturnal malic and citric acid accumulation was assessed by the difference in H^+ concentration between dawn and dusk samples under different light conditions (WL, white light; BL, blue light; RL, red light; FRL, far-red light; DG, darkness; LL, low-light; HL, high-light).

(b) Dawn-dusk ΔH^+ values under different light conditions.

(c) Soluble sugar contents under different light conditions.

(d) Starch accumulation under different light conditions. Embedded *P* values indicate statistical differences between dawn and dusk in (a), (c) and (d) and differences between two pH treatments (a); while asterisks (*, $P < 0.05$; **, $P < 0.01$) indicate significant differences between light quality/intensity treatments and WL control at dawn or dusk. **(e)** Correlation of physiological parameters and gene expression. asterisks (*) indicate significant correlation ($P < 0.05$).

Fig. 7. Expression profiles of genes involved in CAM pathway.

(a) Expression pattern of CAM-related genes under different light intensity (DG, 0 μmol m^{-2} s^{-1}; LL, 150 μmol m^{-2} s^{-1}; WL, 440 μmol m^{-2} s^{-1}; HL, 1000 μmol m^{-2} s^{-1}) and under different light

quality (WL, BL, RL and FRL). The CAM pathway was modified from Yang et al. (2017). Gene name (*Kalanchoë* gene ID, *Arabidopsis* gene ID): *β-CA* (Kaladp0018s0287, AT5G14740), *PPCK1* (Kaladp0037s0517, AT3G04530), *PEPC1* (Kaladp0095s0055, AT3G14940), *MDH2* (Kaladp0001s0257, AT5G09660), *ALMT6* (Kaladp0073s0021, AT1G25480 – missed expression data), *PPDK* (Kaladp0076s0229, AT4G15530), *PPDK-RP* (Kaladp0010s0106, AT4G21210), *NADP-ME* (Kaladp0092s0166, AT1G79750), *TDT* (Kaladp0042s0251, AT5G47560). The expression of *Kalanchoë* genes was detected at dawn (2 h before the starting of lighting period) and dusk (2 h before the dark period, i.e. 10 h after light treatments). The expression data of *Arabidopsis* responsive to light quality (WL, BL, RL and FRL) was obtained from *Arabidopsis* eFP browser (light series). *Arabidopsis* seeds were plated on 1.2% MS agar plats and stratified at 8°C for 48 h in the dark. Germination was induced with 2 h red light, followed by growth for 94 h in complete darkness at 22°C. Plants were then irradiated at different light conditions. Samples (mainly hypocotyl and cotyledons) were collected at 4 h after treatments.

(b) Subnetwork of *MDH2* (Kaladp0001s0257). Orange nodes represent center of the subnetworks; red and green nodes represent TFs and photosynthesis-related genes, respectively. Large and small nodes represent the 1st and 2nd co-expressed genes, respectively. Thick and thin edges indicate the 1st and 2nd co-expression relationships, respectively. Red and blue edges indicate the positive and negative correlation, respectively.

Fig. 8. Expression patterns of photoreceptors in *Kalanchoë* leaf.

(a) Light signaling model from photoreceptor to photo-responsiveness. **(b)** Expression patterns of photoreceptors under different light conditions. Gene name (*Kalanchoë* gene ID, *Arabidopsis* gene ID): *CRY2* (Kaladp0082s0193, AT1G04400), *PHOT1* (Kaladp0032s0316, AT3G45780), *PHOT2* (Kaladp0055s0063, AT5G58140), *phyA* (Kaladp0034s0172, AT1G09570), *phyB* (Kaladp0039s0298, AT2G18790), *phyE* (Kaladp0053s0072, AT4G18130). The expression data of *Arabidopsis* responsive to light quality (WL, BL, RL and FRL) was obtained from *Arabidopsis* eFP browser (light series). **(c)** Subnetwork of *PHOT1*. **(d)** Subnetwork of *phyA*. Orange nodes represent center of the subnetworks; red and green nodes represent TFs and circadian-/light

responsive-genes, respectively. Large and small nodes represent the 1st and 2nd co-expressed genes, respectively. Thick and thin edges indicate the 1st and 2nd co-expression relationships, respectively.

Fig. 9. The expression pattern of *Kalanchoë fedtschenkoi* stomatal movement-related genes.

(a) Expression of stomatal movement-related genes under different light conditions. Gene name (*Kalanchoë* gene ID, *Arabidopsis* gene ID): *ABI2* (Kaladp0048s0509, AT5G57050), *ALMT9* (Kaladp0062s0038, AT3G18440), *KAT1* (Kaladp0008s0789, AT5G46240), *KAT2* (Kaladp0840s0007, AT4G18290), *OST1* (Kaladp0016s0289, AT4G33950), *QUAC1/ALMT12* (Kaladp0091s0013, AT4G17970). The expression data of *Arabidopsis* responsive to light quality (WL, BL, RL and FRL) was obtained from *Arabidopsis* eFP browser (light series). **(b)** Subnetwork of stomatal movement-related gene *ABI2*. Orange nodes represent center of the subnetworks; green, yellow and red nodes represent TFs and circadian genes, circadian TFs and other TFs, respectively. Large and small nodes represent the 1st and 2nd co-expressed genes, respectively. Thick and thin edges indicate the 1st and 2nd co-expression relationships, respectively.

Supporting information

Additional Supporting Information may be found online in the Supporting Information section at the end of the article:

Figure S1. Expression distribution and correlation of 42 RNA-seq libraries.

(a) Distribution of gene expression levels of all the samples in this study. The gene expression levels were transformed by $\log_{10}$(TPM+1). (b) Pearson correlation between samples.

Figure S2. Gene ontology (GO) enrichment of DEGs in different comparisons.

BP, biological process; MF, molecular function; and CC, cellular component. GOslim terms were shown in here, full list of enriched GO terms was shown in Supplementary Table S3.

Figure S3. Functional classification of DEGs in MapMan BINs.

DEG number (a) and DEG percentage (b) of different comparisons in 29 MapMan BINs.

Figure S4. Expression pattern of DEGs in different MapMan BINs.

Color scale of blue-white-red represents Z-score normalized relative expression in the 14 samples.

Figure S5. Transcription factor (TF) number and enrichment in the co-expression modules.

Figure S6. Scatter plots (lower triangle) and correlations (upper triangle) among four physiological traits of *K. fedtschenkoi* under different light treatments.

Figure S7. The expression pattern of *Kalanchoë fedtschenkoi* circadian rhythm-related genes at dawn and dusk under different light conditions.

Table S1. Experimental conditions and statistic of RNA-Seq data in this study.

Table S2. Differentially expressed genes (DEGs) in pairwise comparisons.

Table S3. Full list of enriched GO terms of DEGs in different comparisons.

Table S4. Expression patterns of photosynthetic genes.

Table S5. Full list of enriched GO terms of different WGCNA modules.

Table S6. Transcription factors in WGCNA modules.

Table S7. Gene list and functional annotation of the sub-network.

Table S8. Expression patterns of CAM-, circadian-, stomatal movement-related and photosynthetic genes.

Table S9. Correlation of physiological parameters and gene expression.

Click here to access/download;Figure;Figure 1.tiff

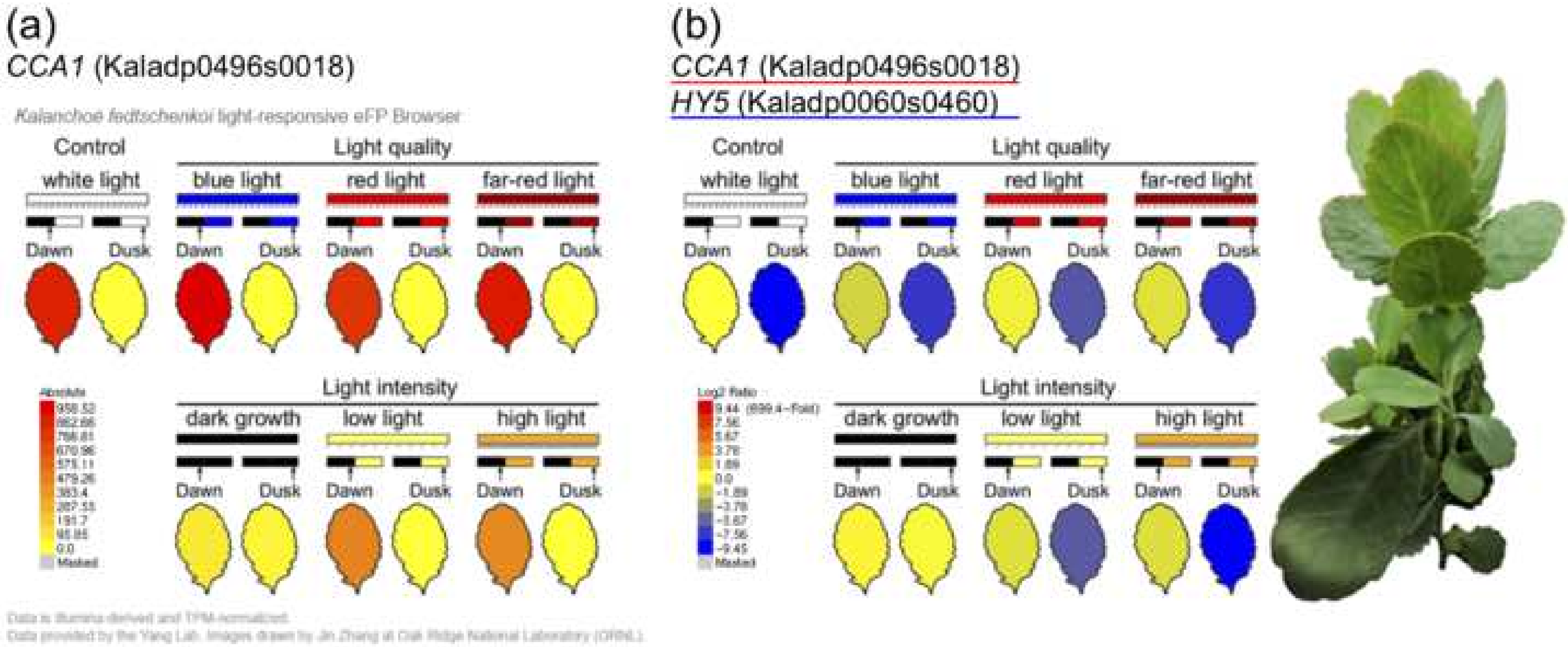

Click here to access/download;Figure;Figure 2.tiff

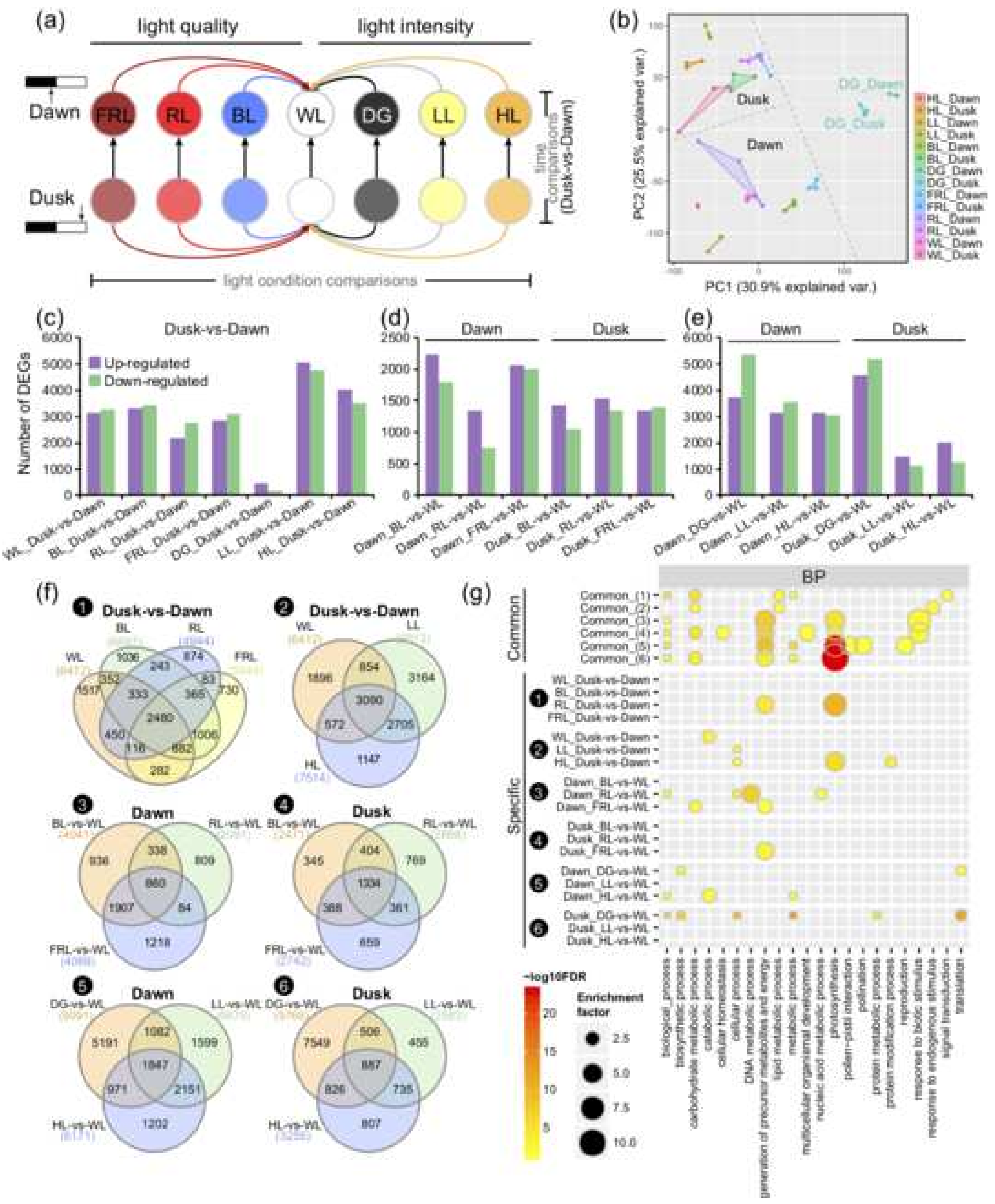

Click here to access/download;Figure;Figure 3_Mapman.tiff

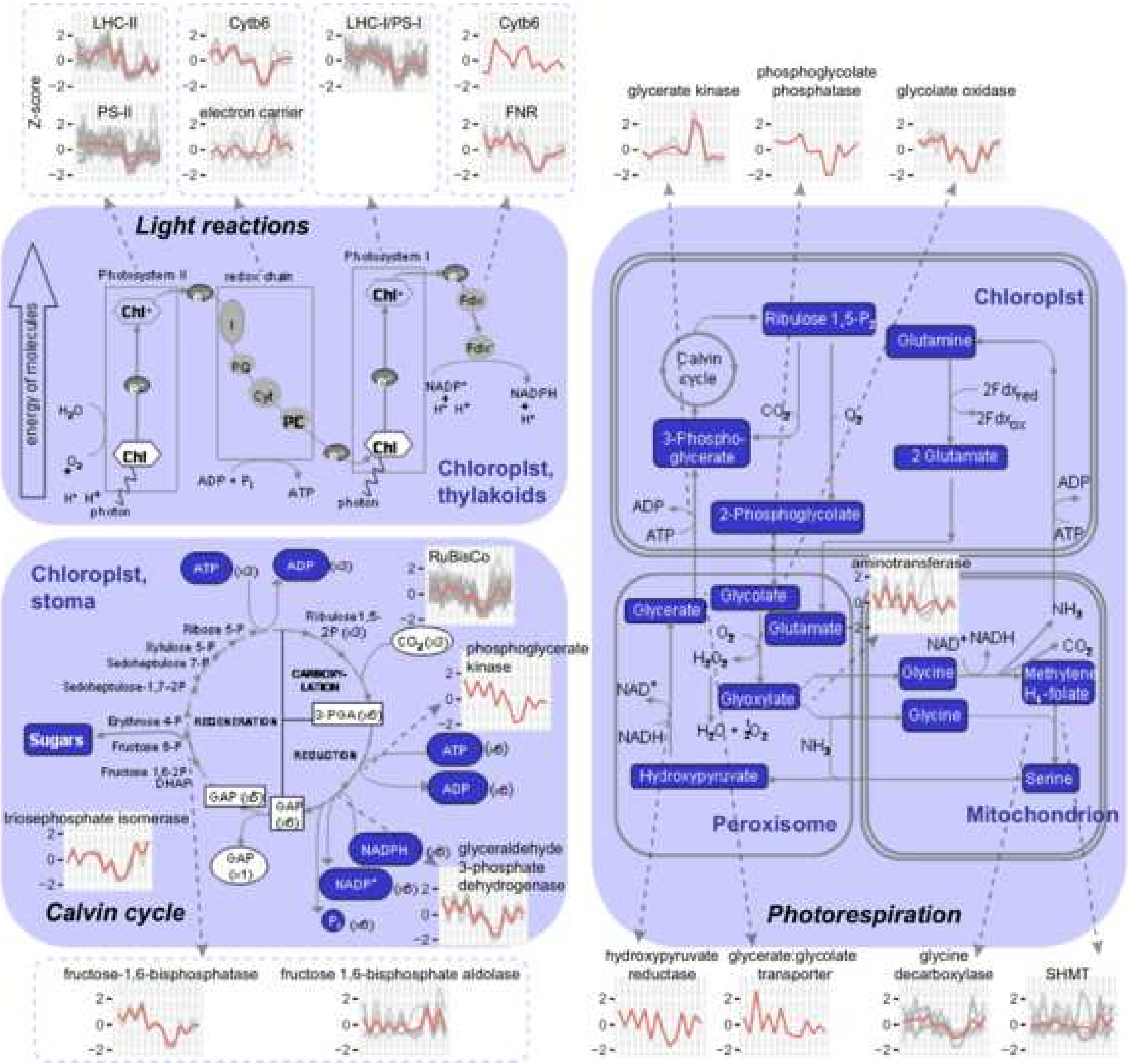

Click here to access/download;Figure;Figure 4_WGCNA.tiff

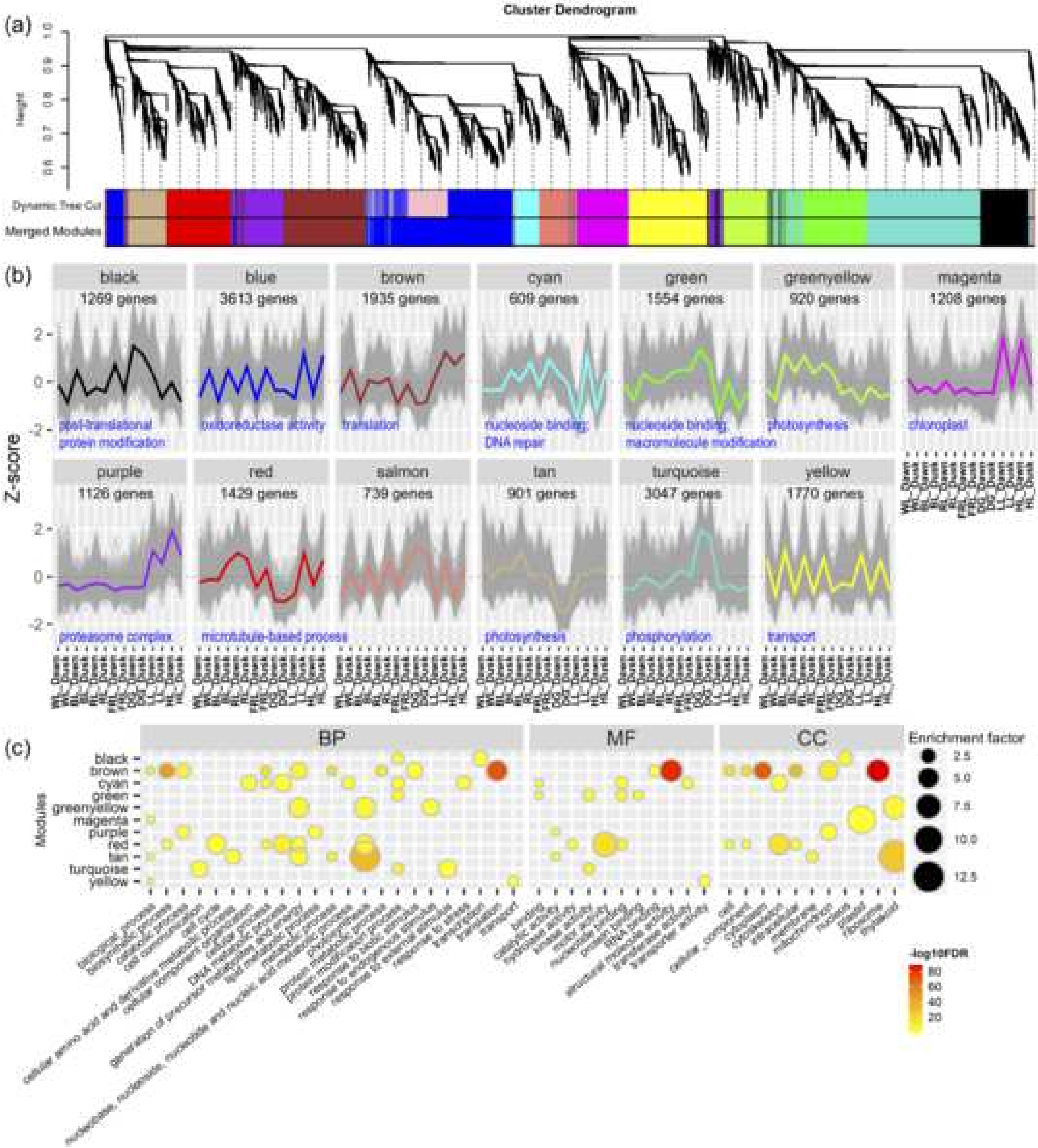

Click here to access/download;Figure;Figure 5.tiff

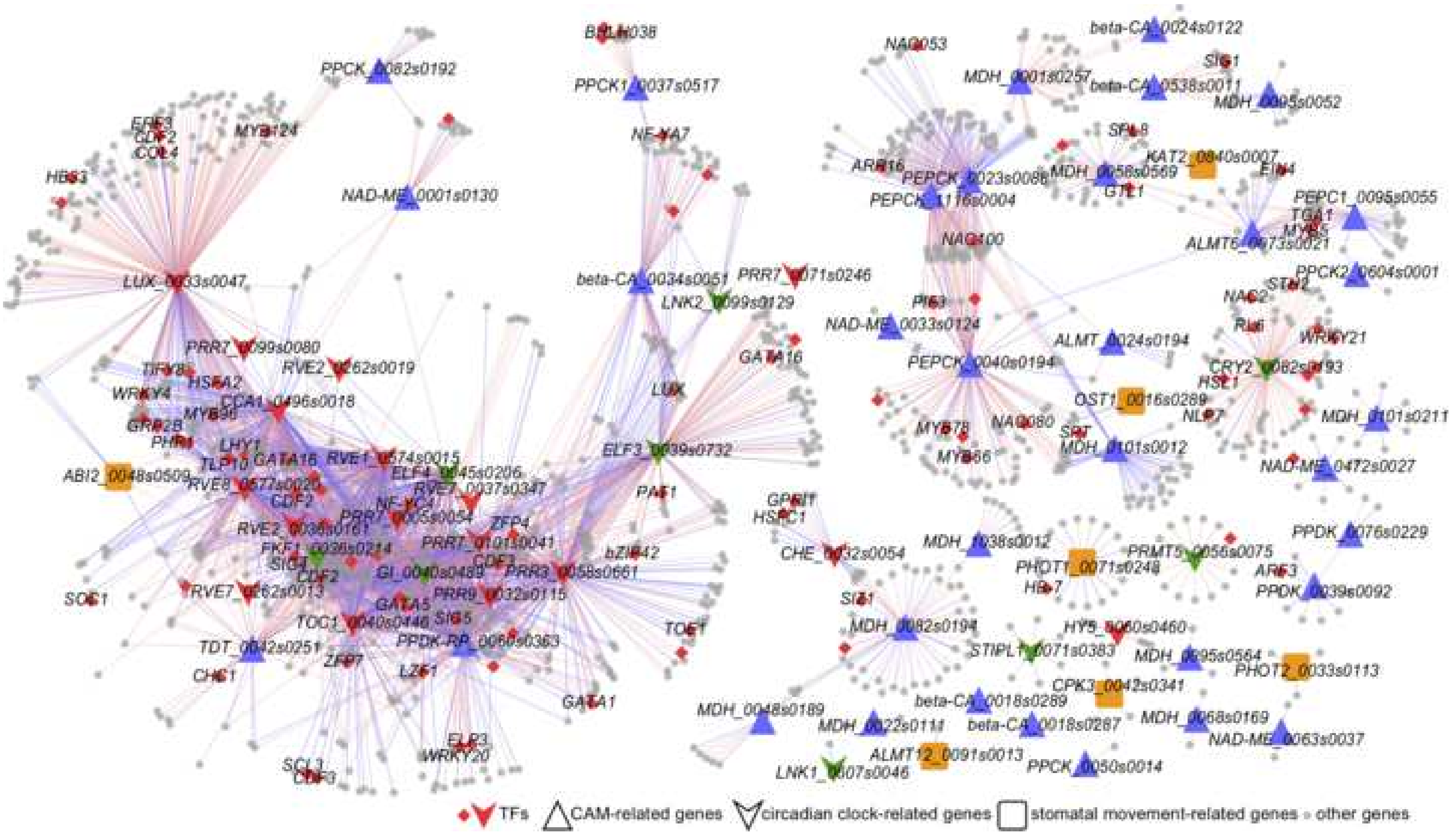

Click here to access/download;Figure;Figure 6 _ TA.SS.Starch_V2.Jin.tiff

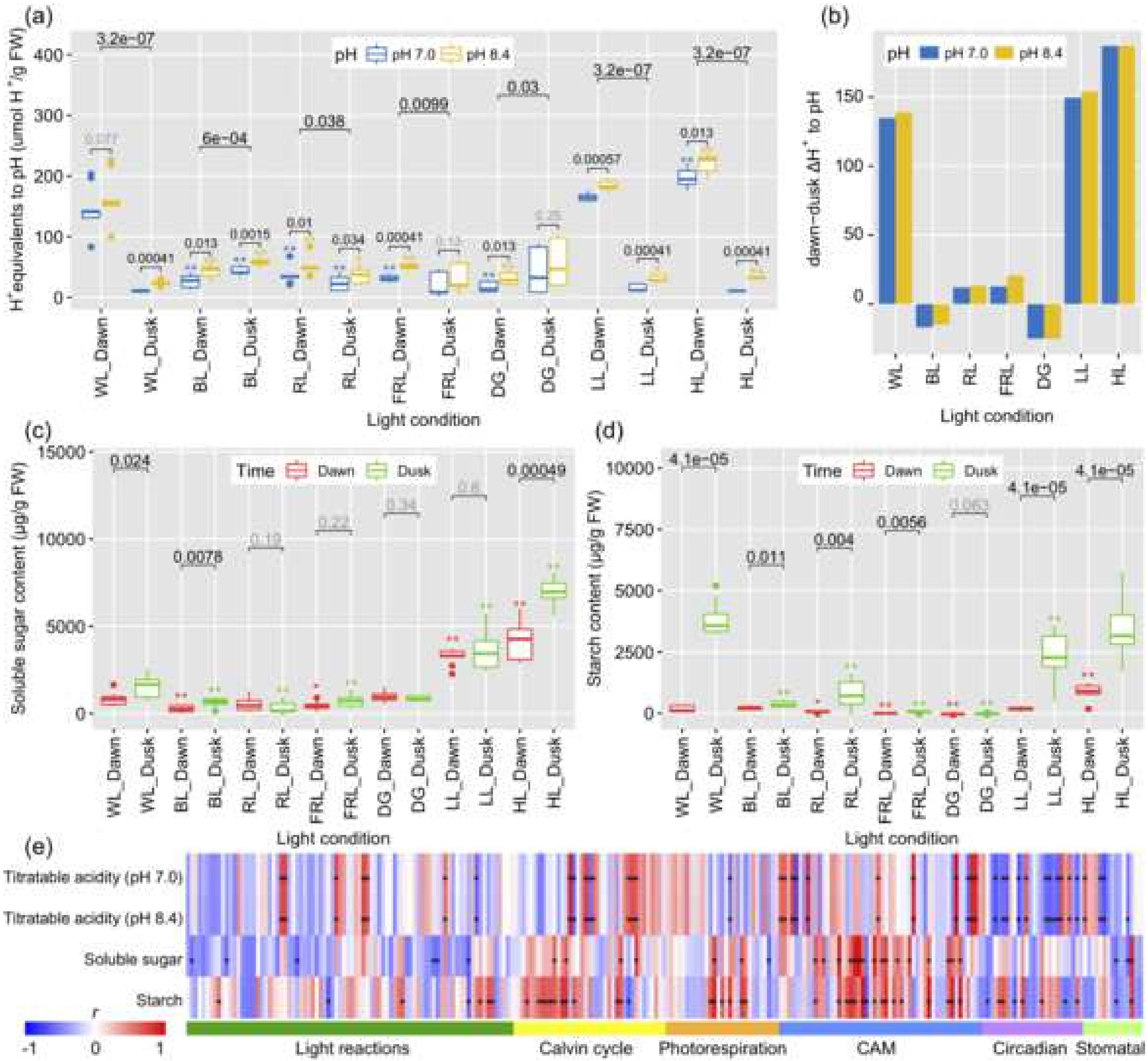

Click here to access/download;Figure;Figure 7 _V3.tiff

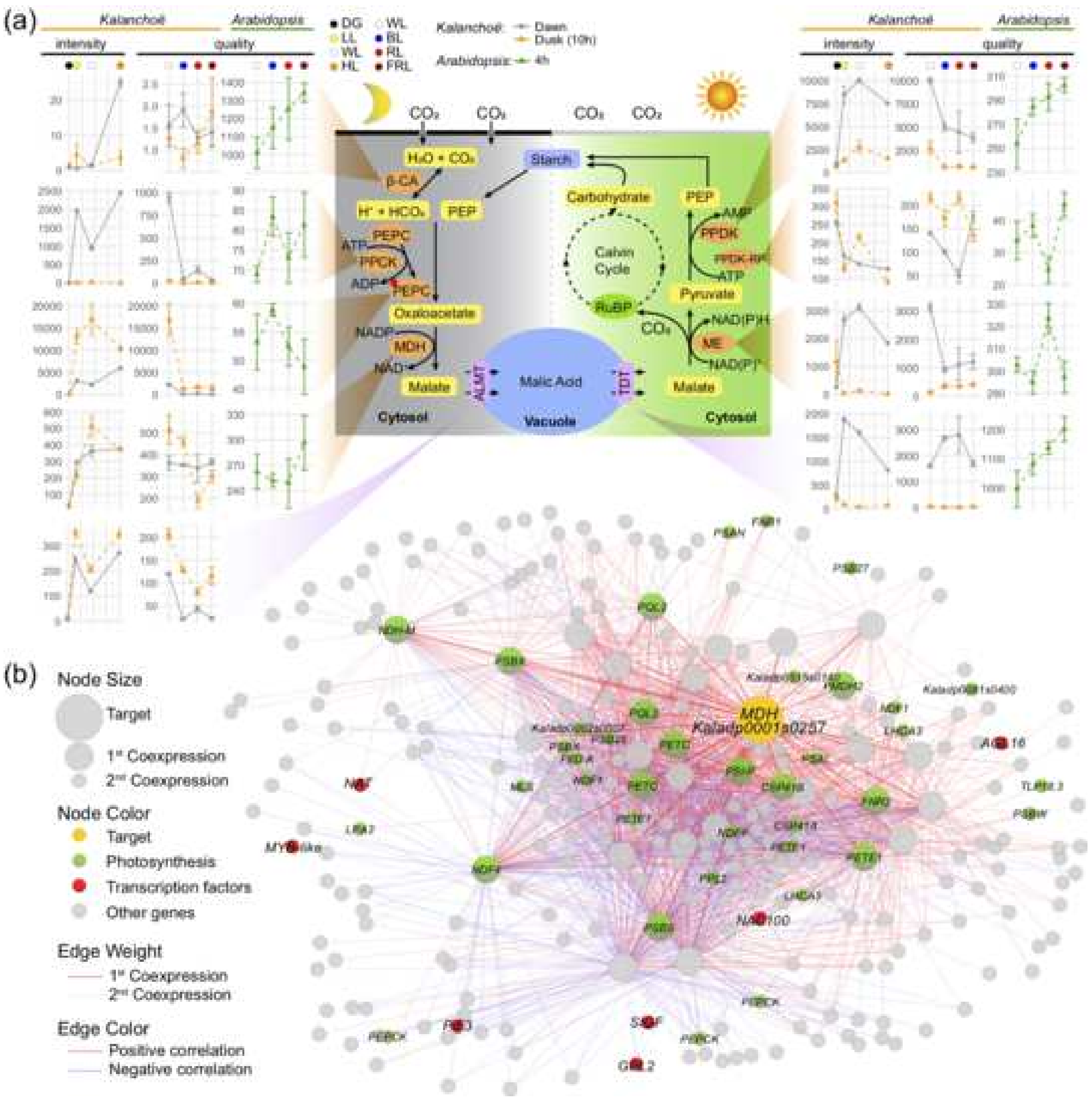

Click here to access/download;Figure;Figure 8 _ light receptor_V4.tiff

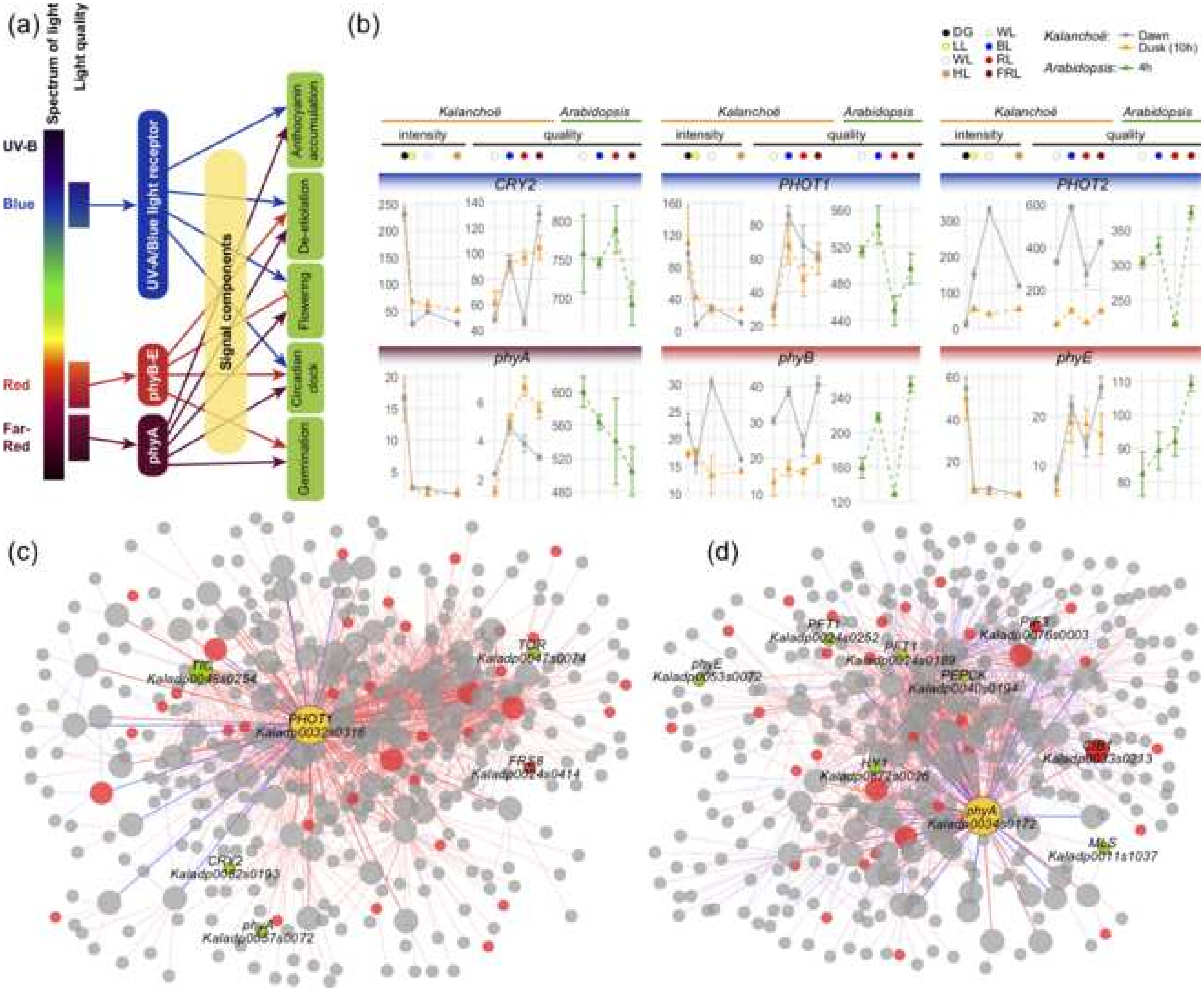

Click here to access/download;Figure;Figure 9 _V2.tiff

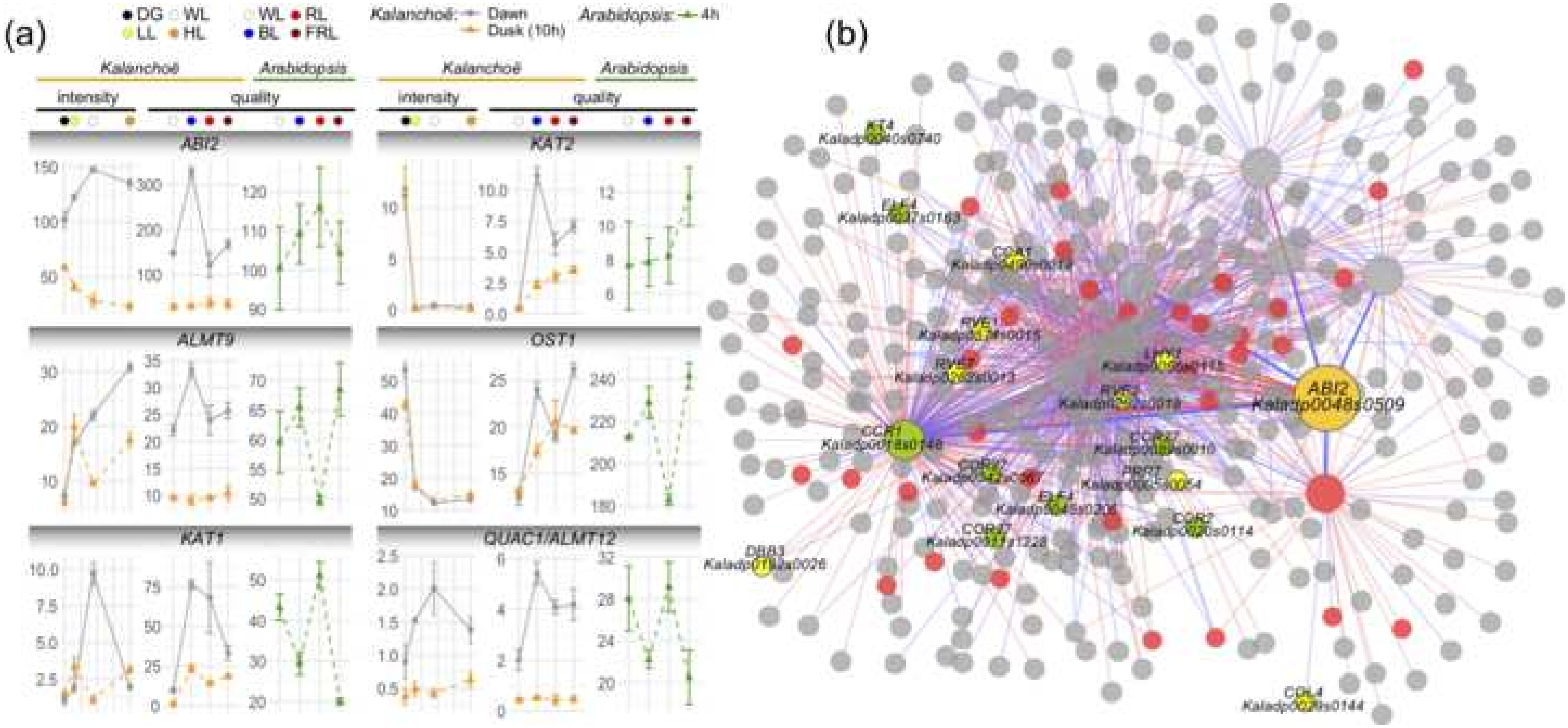

Click here to access/download
Supplementary Material
SI_JZ.docx

Table S1

Click here to access/download
Supplementary Material
Table S1 _ Samples.xlsx

Table S3

Click here to access/download
Supplementary Material
Table S3 _ GO all DEGs.xlsx

Click here to access/download
Supplementary Material
Table S4 _ Photosynthesis Mapman.xlsx

Table S5

Click here to access/download
Supplementary Material
Table S5 _ WGCNA GO.xlsx

Table S6

Click here to access/download

Supplementary Material

Table S6 _ WGCNA TFs.xlsx

Table S7

Click here to access/download
Supplementary Material
Table S7 _ subnetwork.xlsx

Table S8

Click here to access/download
Supplementary Material
Table S8 _ CAM genes.xlsx

Table S9

Click here to access/download
Supplementary Material
Table S9 _ correlation.xlsx
