# Supplementary material for: Light-responsive expression atlas reveals the effects of light quality and intensity in Kalanchoë fedtschenkoi, a plant with crassulacean acid metabolism
Source: Gigascience. 2020 Mar 5;9(3):giaa018. doi: 10.1093/gigascience/giaa018 (PMC7058158; doi:10.1093/gigascience/giaa018)
Supplement: giaa018_Supplemental_Files [file giaa018_supplemental_files.zip › SI_JZ.docx]

**Supplementary Information**

**Jin Zhang et al.** Light-responsive expression atlas reveals the effects of light quality and intensity in *Kalanchoë fedtschenkoi*, a plant with crassulacean acid metabolism


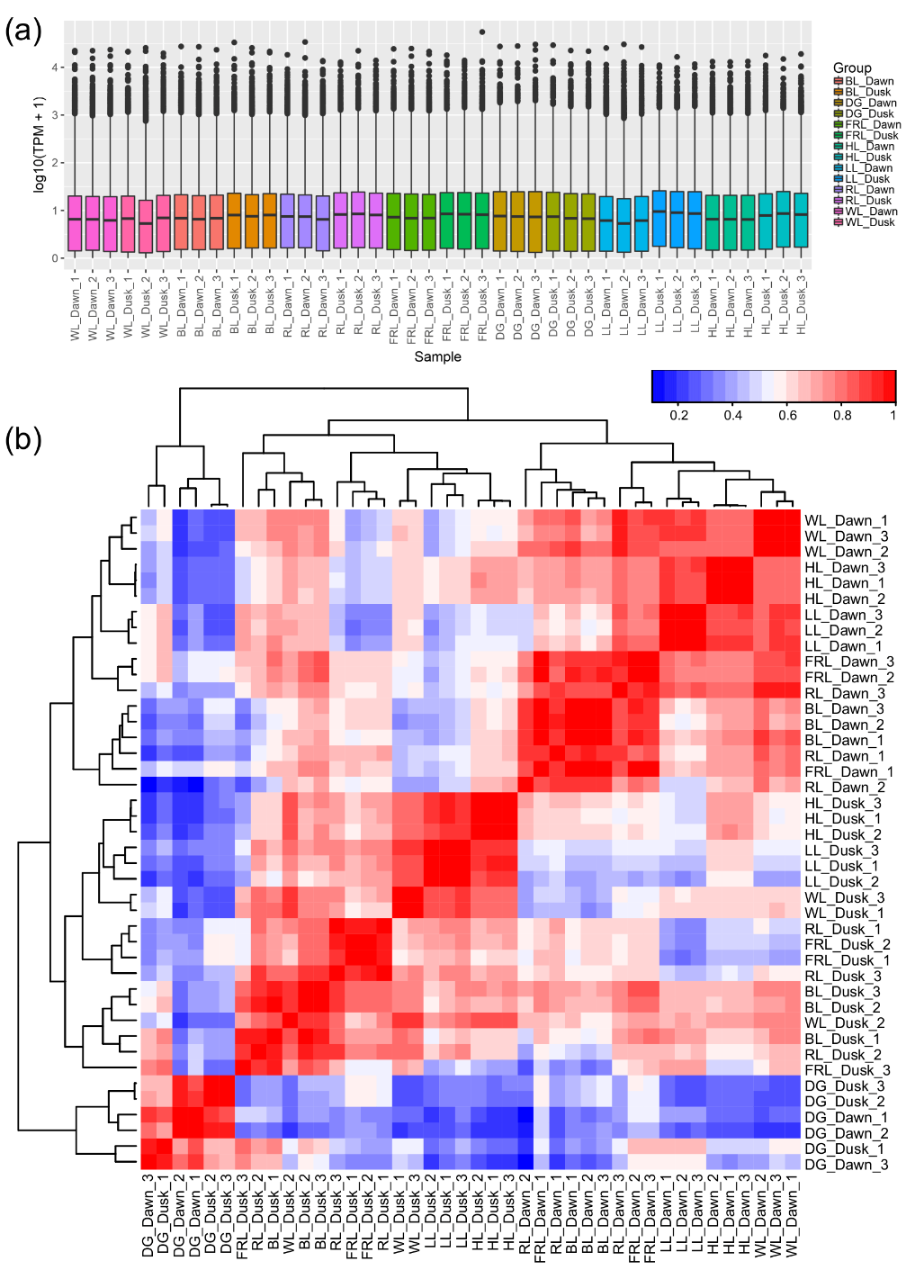


**Figure S1. Expression distribution and correlation of 42 RNA-seq libraries.**

(a) Distribution of gene expression levels of all the samples in this study. The gene expression levels were transformed by log_10_(TPM+1). (b) Pearson correlation between samples.


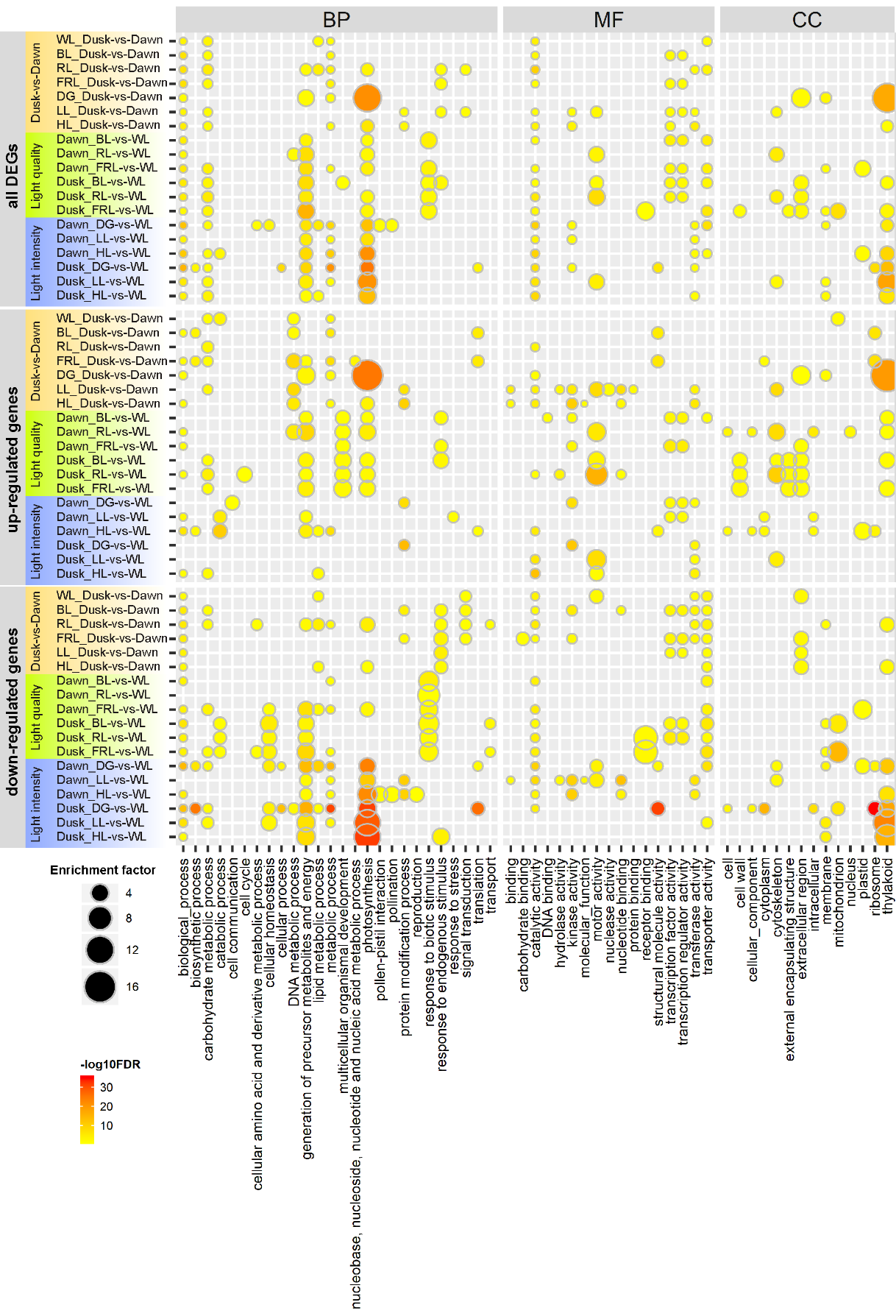


**Figure S2. Gene ontology (GO) enrichment of DEGs in different comparisons.**

BP, biological process; MF, molecular function; and CC, cellular component. GOslim terms were shown in here, full list of enriched GO terms was shown in Supplementary Table S3.


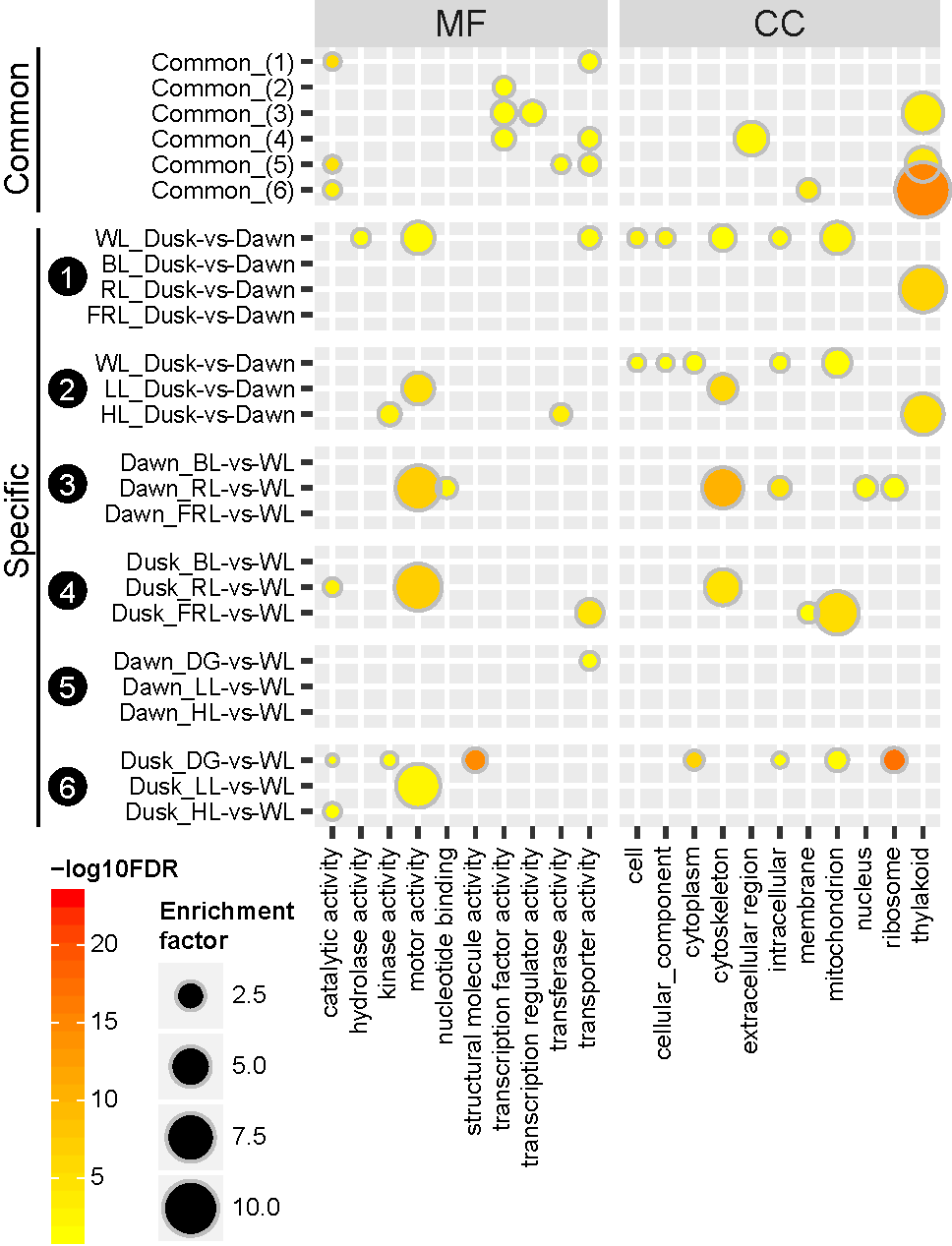


**Figure S3.** **Gene ontology (GO) enrichment of DEGs shared by different comparisons in Figure 2f Venn diagrams (Common) or specific DEGs in each Venn diagram.**

Detailed enrichment of molecular function (MF) and cellular component (CC) was shown in Supplementary Figure S. GOslim terms were shown in here.


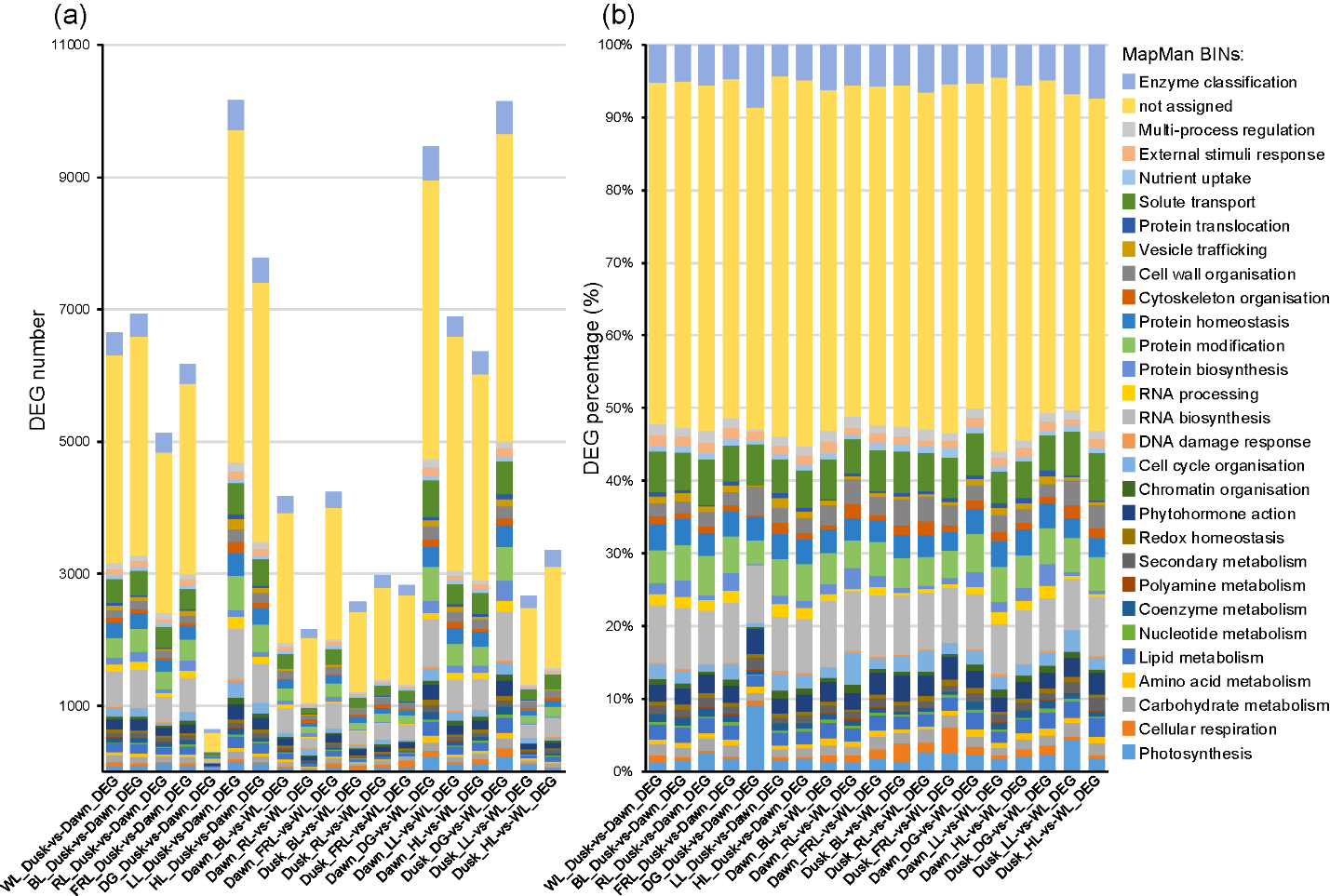


**Figure S4. Functional classification of DEGs in MapMan BINs.**

DEG number (a) and DEG percentage (b) of different comparisons in 29 MapMan BINs.


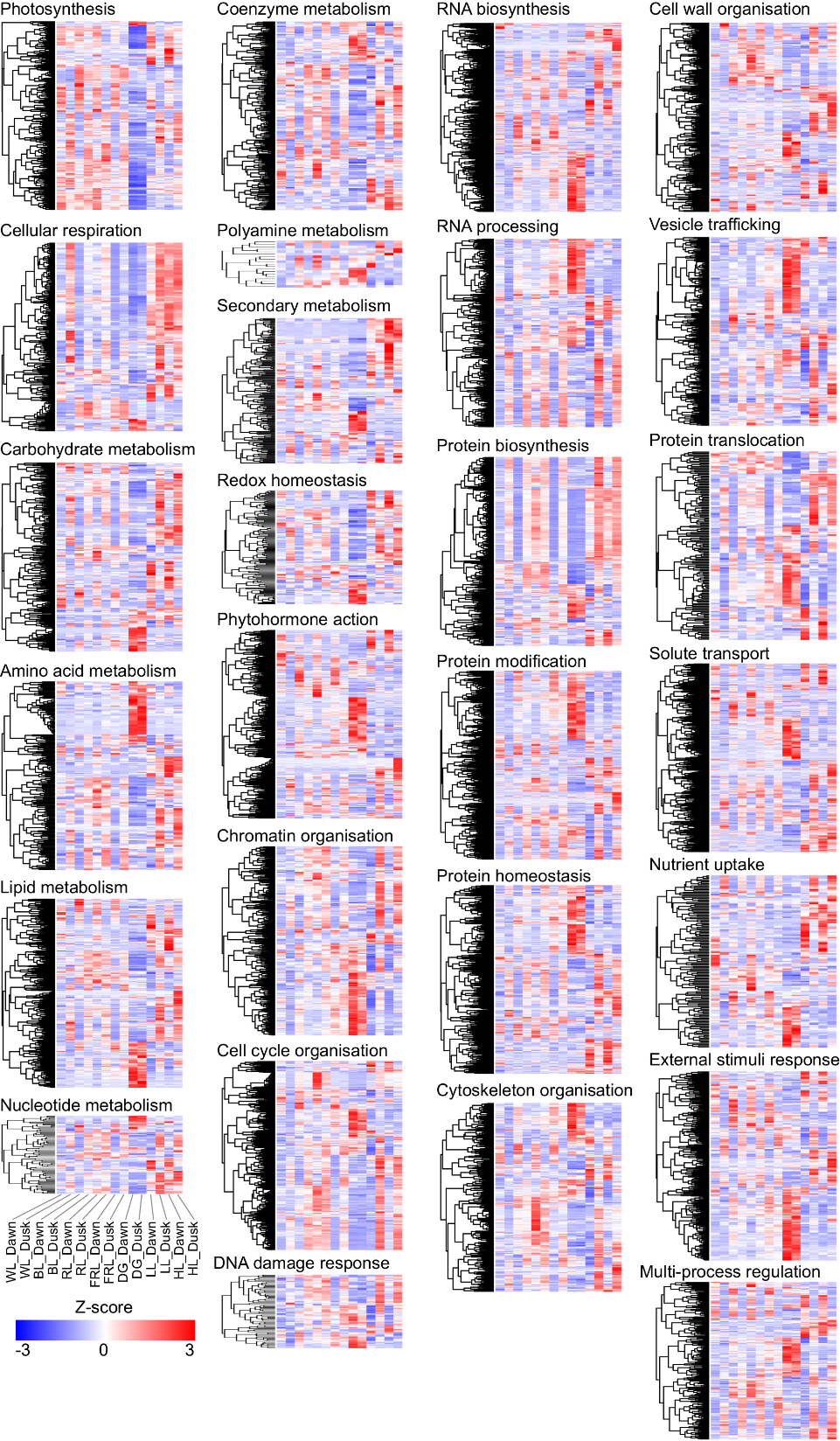


**Figure S5. Expression pattern of DEGs in different MapMan BINs.**

Color scale of blue-white-red represents Z-score normalized relative expression in the 14 samples.


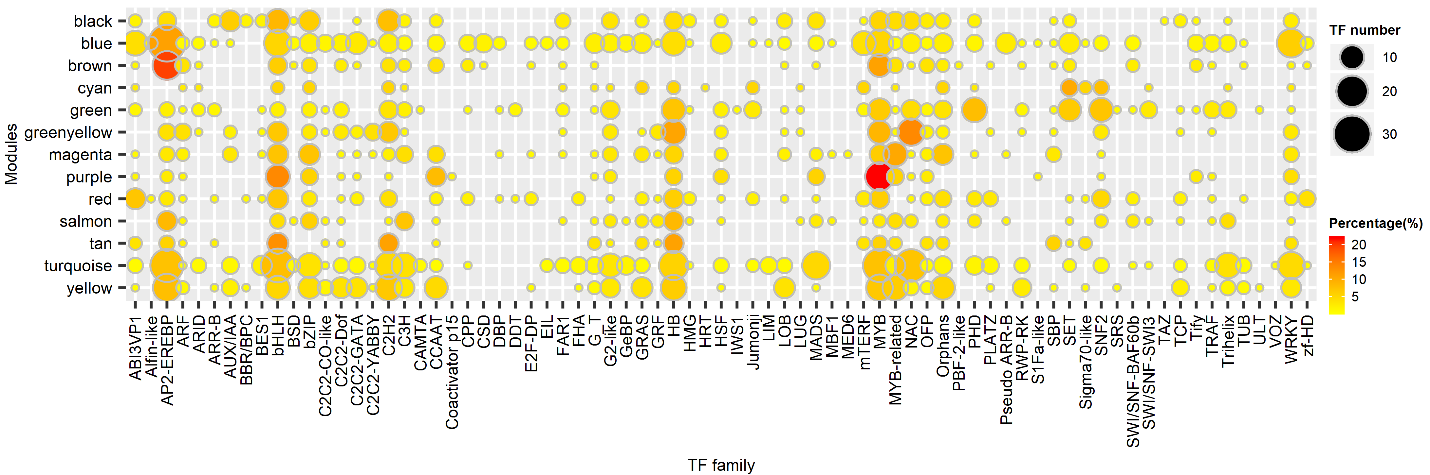


**Figure S6. Transcription factor (TF) number and enrichment in the co-expression modules.**


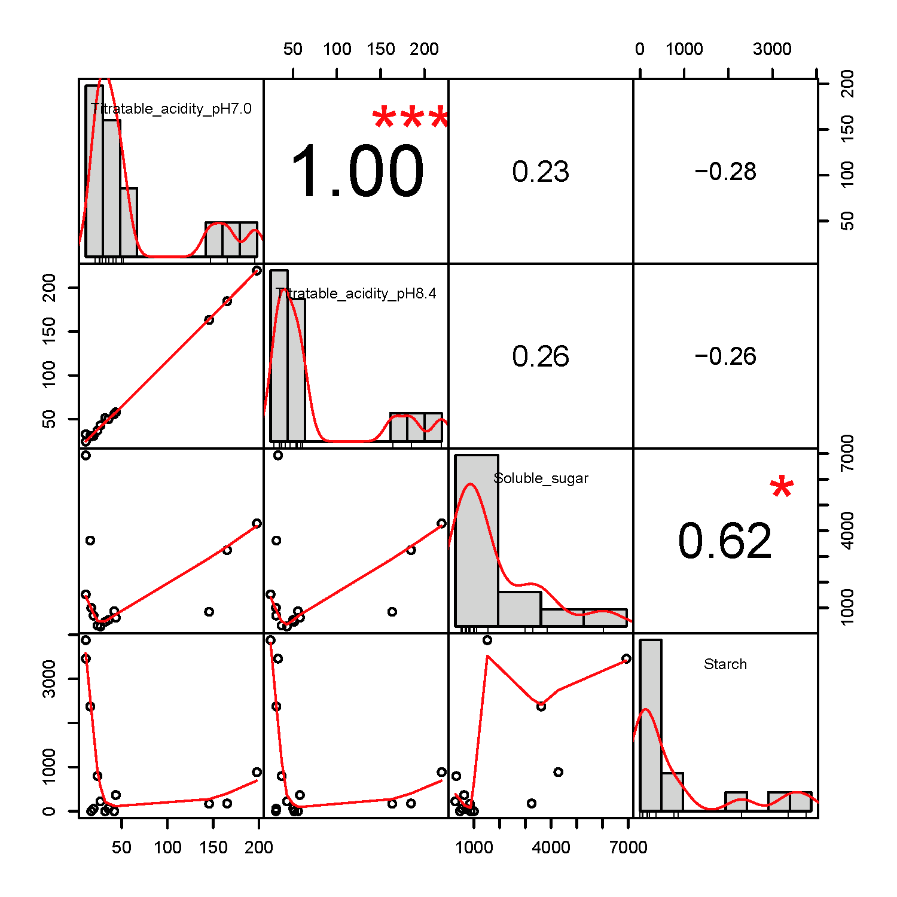


**Figure S7.** **Scatter plots (lower triangle) and correlations (upper triangle) among four physiological traits of *K. fedtschenkoi* under different light treatments.**

* and *** significant difference at the *P* < 0.01 and 0.001 levels, respectively.


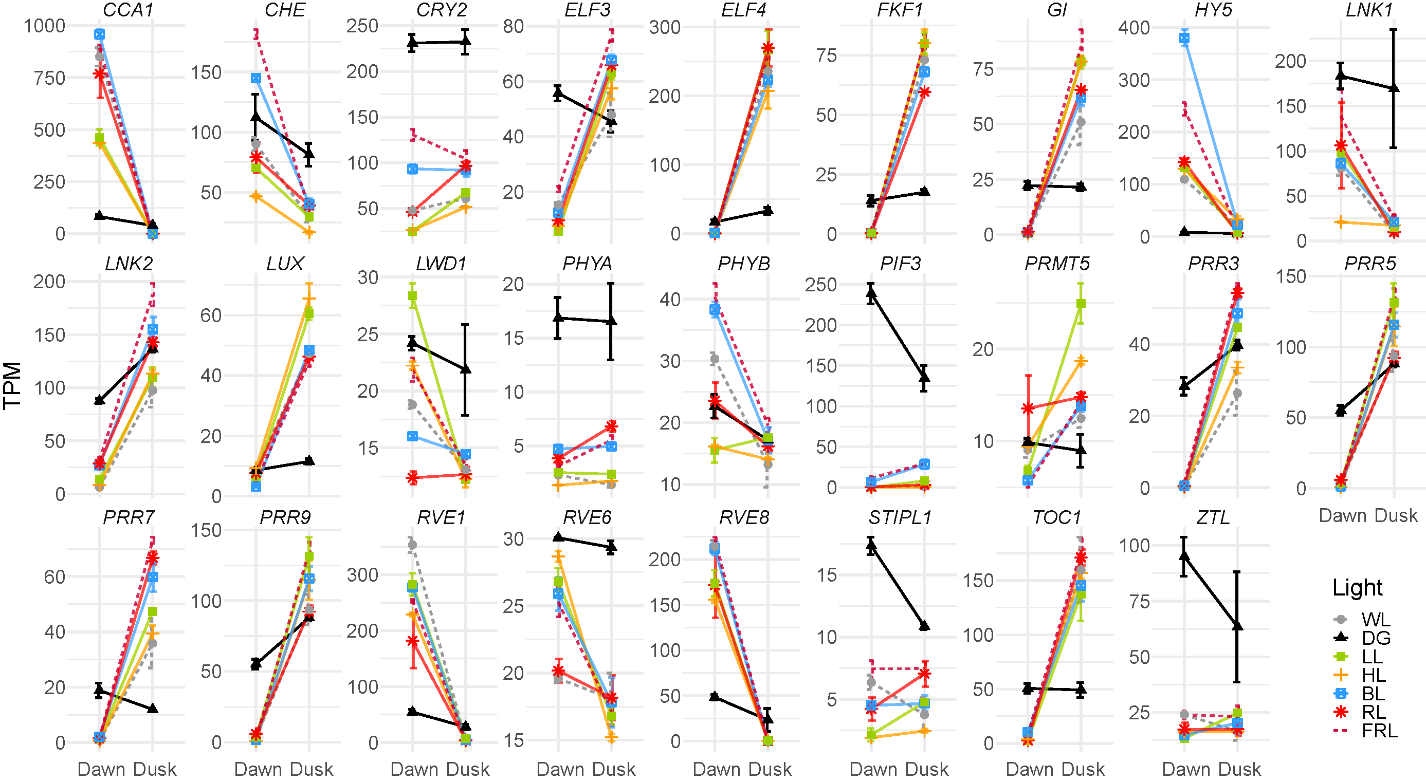


**Figure S8. The expression pattern of *Kalanchoë fedtschenkoi* circadian rhythm-related genes at dawn and dusk under different light conditions.**

*CCA1* (Kaladp0496s0018), *CHE* (Kaladp0032s0054), *CRY2* (Kaladp0082s0193), *ELF3* (Kaladp0039s0732), *ELF4* (Kaladp0045s0206), *FKF1* (Kaladp0036s0214), *GI* (Kaladp0040s0489), *HY5* (Kaladp0060s0460), *LNK1* (Kaladp0607s0046), *LNK2* (Kaladp0099s0129), *LUX* (Kaladp0033s0047), *LWD1* (Kaladp0048s0797), *phyA* (Kaladp0034s0172), *phyB* (Kaladp0039s0298), *PIF3* (Kaladp0057s0097), *PRMT5* (Kaladp0056s0075), *PRR3* (Kaladp0058s0661), *PRR5* (Kaladp0032s0115), *PRR7* (Kaladp0101s0041), *PRR9* (Kaladp0032s0115), *RVE1* (Kaladp0574s0015), *RVE6* (Kaladp0055s0349), *RVE8* (Kaladp0577s0020), *STIPL1* (Kaladp0071s0383), *TOC1* (Kaladp0040s0446), *ZTL* (Kaladp0809s0098).

**Table S1. Experimental conditions and statistic of RNA-Seq data in this study.** (included as a separate excel file)

**Table S2. Differentially expressed genes (DEGs) in pairwise comparisons.**

| Comparison group | Comparison | Up-regulated DEGs | Down-regulated DEGs | Total DEGs |
| --- | --- | --- | --- | --- |
| Time comparisons | WL_Dusk-vs-Dawn | 3137 | 3275 | 6412 |
|  | BL_Dusk-vs-Dawn | 3287 | 3410 | 6697 |
|  | RL_Dusk-vs-Dawn | 2168 | 2776 | 4944 |
|  | FRL_Dusk-vs-Dawn | 2850 | 3094 | 5944 |
|  | DG_Dusk-vs-Dawn | 458 | 173 | 631 |
|  | LL_Dusk-vs-Dawn | 5050 | 4763 | 9813 |
|  | HL_Dusk-vs-Dawn | 4001 | 3513 | 7514 |
| Light condition comparisons | Dawn_BL-vs-WL | 2241 | 1800 | 4041 |
|  | Dawn_RL-vs-WL | 1339 | 752 | 2091 |
|  | Dawn_FRL-vs-WL | 2061 | 2008 | 4069 |
|  | Dusk_BL-vs-WL | 1433 | 1038 | 2471 |
|  | Dusk_RL-vs-WL | 1531 | 1337 | 2868 |
|  | Dusk_FRL-vs-WL | 1349 | 1393 | 2742 |
|  | Dawn_DG-vs-WL | 3722 | 5369 | 9091 |
|  | Dawn_LL-vs-WL | 3125 | 3554 | 6679 |
|  | Dawn_HL-vs-WL | 3129 | 3042 | 6171 |
|  | Dusk_DG-vs-WL | 4569 | 5199 | 9768 |
|  | Dusk_LL-vs-WL | 1457 | 1126 | 2583 |
|  | Dusk_HL-vs-WL | 2015 | 1240 | 3255 |

**Table S3. Full list of enriched GO terms of DEGs in different comparisons.** (included as a separate excel file)

**Table S4. Expression patterns of photosynthetic genes.** (included as a separate excel file)

**Table S5. Full list of enriched GO terms of different WGCNA modules.** (included as a separate excel file)

**Table S6. Transcription factors in WGCNA modules.** (included as a separate excel file)

**Table S7. Gene list and functional annotation of the sub-network.** (included as a separate excel file)

**Table S8. Expression patterns of CAM-, circadian-, stomatal movement-related and photosynthetic genes.** (included as a separate excel file)

**Table S9. Correlation of physiological parameters and gene expression.** (included as a separate excel file)
