# Supplementary material for: Light-responsive expression atlas reveals the effects of light quality and intensity in Kalanchoë fedtschenkoi, a plant with crassulacean acid metabolism
Source: Gigascience. 2020 Mar 5;9(3):giaa018. doi: 10.1093/gigascience/giaa018 (PMC7058158; doi:10.1093/gigascience/giaa018)
Supplement: giaa018_Response_to_Reviewer_Comments_Original_Submission [file giaa018_response_to_reviewer_comments_original_submission.pdf]

Author's Response To Reviewer Comments

Close

The Editor
Dr. Hans Zauner
GigaScience

November 8th, 2019

Dear Editor,
Thank you for your evaluation of our manuscript GIGA-D-19-00095 (Expression atlas and co-expression network reveal effects of light quality and intensity in Kalanchoë fedtschenkoi, a plant with crassulacean acid metabolism) and the invitation to submit a revised manuscript. Enclosed please find the revised manuscript in which all the comments raised by the reviewers and you have been addressed. A detailed, point-by-point rebuttal to these comments in included below. For your convenience, we highlighted all changes in the manuscript text file. We hope this modified version is now suitable for publication in GigaScience.

Sincerely,
Xiaohan Yang
Senior Staff Scientist
Biosciences Division
Oak Ridge National Laboratory, USA
Phone: (865)-241-6895
E-mail: yangx@ornl.gov
https://www.ornl.gov/staff-profile/xiaohan-yang

Dear Dr. Zhang,

Your manuscript "Expression atlas and co-expression network reveal effects of light quality and intensity in Kalanchoë fedtschenkoi, a plant with crassulacean acid metabolism" (GIGA-D-19-00095) has been assessed by our reviewers. Although it is of interest, we are unable to consider it for publication in its current form. The reviewers have raised a number of points which we believe would improve the manuscript and may allow a revised version to be published in GigaScience.

Their reports are below.
Generally speaking, in light of the reviewers's reports, I get the impression that your article falls in between two article types we publish in GigaScience:
We publish "Data Notes", which present useful resources and describe in detail how the data have been generated, including test / validation data and discussing use cases. "Data Notes" don't need to answer biological questions.
In contrast, our "Research Articles" should answer biological questions, in addition to describing the datasets.
Both reviewers agree that your manuscript presents a useful resource, but they got the impression that it is lacking a bit in terms of the biological insights presented. I suggest there are two ways forward during the revision: You may choose to re-submit as a "Research Article". To convince the reviewers I feel you will need to expand on the biological insights gained from your work, also including some further analyses. Alternatively, you may decide to reformat the submission as a "Data Note", and focus on presenting the data as a resource and showing some use cases.
[Response]: Thanks for your suggestion. We have added the additional experiments according to reviewers' comments, performed further data analyses and added a substantial amount of additional description and discussion into the biological insights. Thus, we have re-submitted the manuscript as a "Research Article".

In any case, please address the questions and comments from both reviewers and fill in missing details

regarding the methodology (e.g. number of replicates, method for library construction etc - see below). Please also carefully address reviewer 2's comments regarding the clustering procedures. In particular, I agree with the reviewer that "outliers" should not be arbitrarily removed, please include all replicates in the analyses.
[Response]: Yes, we have added all the replicates in the revised manuscript according to your and reviewer #2's suggestions.

If you are able to fully address these points, we would encourage you to submit a revised manuscript to GigaScience. Once you have made the necessary corrections, please submit online at:
https://www.editorialmanager.com/giga/

If you have forgotten your username or password please use the "Send Login Details" link to get your login information. For security reasons, your password will be reset.

Please include a point-by-point within the 'Response to Reviewers' box in the submission system. Please ensure you describe additional experiments that were carried out and include a detailed rebuttal of any criticisms or requested revisions that you disagreed with. Please also ensure that your revised manuscript conforms to the journal style, which can be found in the Instructions for Authors on the journal homepage.

The due date for submitting the revised version of your article is 01 Aug 2019.

I look forward to receiving your revised manuscript soon.

Best wishes,

Hans Zauner
GigaScience
www.gigasciencejournal.com

Reviewer reports:
Reviewer #1: Summary of the key results
=================
The manuscript titled "Expression atlas and co-expression network reveal effects of light quality and intensity in Kalanchoë fedtschenkoi, a plant with crassulacean acid metabolism" describe c es the transcriptomic characterization of the K. fedtschenkoi leaves under different light spectrums (blue, red, infrared and white), intensities (0, 150, 250 and 1000 μmol m-2 s-1) and time points associated to the circadian clock (2h after dusk and after dawn). The authors analyze the differential expression between different light quality conditions (light spectrums and intensity) and time points. Additionally the authors performed a clustering and co-expression network analysis associated with gene ontology terms. Finally to increase the usability of the data for the scientific community, the authors implemented a gene expression graphical interface using the eFP browser at the BAR.

Overall evaluation
===========
This manuscript presents an interesting and useful resource for the plant scientific community, specially the one that it is working in photosynthesis and CAM metabolism. Nevertheless, I found that the manuscript is mostly descriptive without a clear hypothesis and a discussion of the results in agreement with that hypothesis. Probably there is more than enough data to discuss specific aspects of the experiment (e.g. how the light spectrum activate some pathways but not others, why the transcriptomic response is higher with low and high light...). Overall, I think that the authors develop an useful resource but at the same time, I think that the presentation of the results and the discussion should be improved with a stronger biological support.
[Response]: Thanks for your critical review and constructive suggestions. We have revised the manuscript according to your suggestions. Please see the following point-by-point response.

Major concerns
==========
* Lack of a clear hypothesis behind the experiment. The manuscript is mostly descriptive. I see the article as an useful resource to mine information as the author mentioned at the end of the "Background" section. Nevertheless, in my opinion, this manuscript does not have a clear hypothesis behind the experiment or at least it is not clear from the narrative of the results and the discussion.

[Response]:
We added this following hypothesis to the Introduction section (Lines 116 in revised manuscript):
"We hypothesize that both light intensity and light quality can influence the expression of CAM-related metabolic and signaling genes in the obligate CAM species K. fedtschenkoi."
Also, we added the following text to the end of the Discussion section (Lines 491 in revised manuscript):
"Furthermore, the results from our comparative analyses of gene expression and acid accumulation between different light treatments support our hypothesis that both light intensity and light quality can affect the expression of CAM-related genes in K. fedtschenkoi".

* Poor result discussion. The discussion does not contain any element related with the light quality being in more or the cases a comparison which what is known in Arabidopsis rather than a discussion that drives to new hypothesis about the regulatory cascades of the CAM.
[Response]: We added the following text to the Discussion section: (lines 404 – 414 in revised manuscript):
"We found that high level (1,000 μmol m-2 s-1) of white light increased expression of three dark CO2 fixation genes (i.e., β-CA, PPCK, PEPC) and one malate transporter gene (ALMT) at dawn (i.e., 2 h before the beginning of light period) in comparison with intermediate level (440 μmol m-2 s-1) of white light (Fig. 7a). This increased transcript abundance of genes involved in dark CO2 fixation and malate import into the vacuoles was consistent with the higher acid accumulation (i.e., dawn-dusk ΔH+) under high light relative to intermediate light intensity (Fig. 6b). On the other hand, we found that high level of white light repressed expression of several CAM pathway genes (e.g., PEPC, MDH, PPDK, PPDK-RP) at dusk (i.e., 2 h before the beginning of dark period) in comparison with intermediate level of white light (Fig. 7a). These results suggest that the high-light treatment was still within the normal physiological range of the plant and not yet necessarily saturating the photosynthetic machinery."

Minor concerns
=========
* Language polishing: Although the manuscript is easy to read and there is not major mistakes that I can detect, probably a polishing in the writing will help to do the manuscript more fluid. For example, there are long sentences that written in a different way will improve the readability (e.g. Abstract lines 37-40. Background lines 59-62…).
[Response]: We have revised those long sentences and the current draft of the manuscript has gone through several cycles of editing.

* No summary of number of sequenced and mapped reads per replicate. The authors should provide this information in the supplementary data to have a better evaluation of the process (e.g. if only half of the reads map to the genome is an indication of a problem with the samples).
[Response]: We have added the summary of number of sequenced and mapped reads per replicate in Supplementary Table S1. The mapping rate is ranged from 95.02% to 98.21%.

* Section "Differentially expressed genes (DEGs) regulated by light quality and light intensity": This result section is already summarized in the figure 2 and 3. It is interesting to know if the transcriptomic response is higher depending of the light spectrum, quality and/or time point, but I think that the continuous description of what it can be observed in the figures 2 and 3 blurs the result.
[Response]: Thanks for your suggestion, we removed some excessive descriptions in this section to make the results clearer.

* Section "Predicted function of DEGs": I think that it could be convenient to complement the GSEA with a metabolic pathway analysis using tools such as Mapman (https://mapman.gabipd.org/home) to have a better idea of the members involve in the response to those stimuli rather than a general view. It makes sense that "photosynthesis" is an enriched term comparing dawn and dusk, but probably it could be more interesting to know specifically which members of the photosynthetic apparatus are involved.
[Response]: Yes, we re-analyzed the DEGs using MapMan and added the results in Supplementary Figure S3, S4 and new Figure 3. To explore the role of photosynthetic pathway genes in different light responses, we added the expression patterns of photosynthetic genes in new Figure 3. In addition, we analyzed the correlation between selected genes (related to photosynthetic light reactions, Calvin cycle and photorespiration, CAM pathway, circadian rhythm and stomatal movement) and the physiological parameters (titratable acidity at pH 7.0, titratable acidity at pH 8.4, soluble sugar content and starch content) in new Figure 6 panel (e).

* Section "Clusters of DEGs": As in previous sections, the authors describe the composition of the

clusters, but not the meaning of those in relation with the biological experiment. For example, "genes from clusters 3 and 11 show an strong response to the circadian clock" could be more informative than "clusters 3 and 11 are composed by genes with DE between dawn and dusk".
[Response]: According to Reviewer #2's suggestion, we have removed the section of K-means clustering analysis.

* Lack of details in the Material and Methods: How many plants per biological replicate did the authors used? How the RNA-Seq libraries were prepared? Did the authors perform a size selection of the fragments? Where the samples were sequenced? Which version was used for TopHat2 and FeatureCounts? (other software versions are also missing).
[Response]: We used one plant per biological replicate and three biological replicates per treatment condition. A total of 42 RNA-Seq libraries were prepared. RNAs were fragmented into ~300bp sizes and <200bp sizes of libraries (RNA+adapter) were removed by SPRI beads. The RNA samples were sequenced in US Department of Energy Joint Genome Institute (Walnut Creek, CA). We used GSNAP (v2018-07-04), (not TopHat2), for mapping and FeatureCounts (in Subread v1.6.1) for reads counting. We have added the technical details in the Material and Methods. (line 539-554).

Discretionary revisions
==============
* Background, line 56: Parenthesis is opened but not close.
[Response]: We have revised the sentence: "There are three types of photoreceptors (i.e., cryptochromes, phototropins and phytochromes) that perform important roles in plant light response [2, 4]."

* Background, line 92: "However, gene regulation at the transcriptional level in CAM plants in response to various spectral light qualities and intensity has not been reported yet". I am not sure that "reported" is the most adequate verb here. I will suggest "analyzed", "studied" or "investigated". "Report" is more concrete and linked to a result or an observation.
[Response]: We have revised the sentence: "However, gene regulation at the transcriptional level in CAM plants in response to various light qualities and intensities has not been investigated yet."

* Data description, line 116: "we obtained ~138 Gb of high-quality data". High-quality data in this context is an unprecise term. Please, be more precise (e.g. qscore > 30…).
[Response]: We have added the description in line 137-139: "In total, we obtained 981 million read pairs (2×150 bp) with 287.78 Gb high quality data (Qscore≥25) from the 42 libraries, with an average size of ~23.4 million read pairs per library (Supplementary Table S1)."

* Analysis, line 176: Probably the authors forget to replace the note "cite a figure or table here" by a real figure.
[Response]: The relevant excessive description was removed.

* Analysis, line 217: Why 20 clusters?
[Response]: We have removed the cluster analysis according to your and reviewer #2's suggestion.

* Discussion, line 335: "carbohydrate metabolic" should be "carbohydrate metabolism".
[Response]: Yes, revised.

* Figure 2: Abbreviations for each conditions should be described in the legend (e.g. LL=Low Light…).
[Response]: Yes, we have added the explanation of the abbreviations in the figure legend.

Reviewer #2: The Authors present their comparative transcriptomics work examining the effect of different light regimes on the new model CAM plant, Kalanchoë fedtschenkoi. The authors perform some large scale typical analyses including differential gene expression and clustering, which indicate the data is of workable quality. Finally the authors touch briefly on a few co-expressed known and novel regulatory factors that may-be of interest in understanding how light signals are integrated into regulating CAM photosynthesis.

Major comments:
1-
The dataset that the authors have generated appears to be of good quality and I anticipate that it will be very useful for the CAM research community.

I also appreciate that both the raw data and the included eFP browser are already available. That said, if the authors are still able to provide some contextual data; I think this might make it much easier to interpret the transcriptomics data.
[Response]: We added some contextual data to the caption of Fig. 1.
"The leaf samples were collected at dawn (i.e., 2 h before the beginning of light period) and dusk (i.e., 2 h before the beginning of dark period) under control condition (WL, white light) and various light quality conditions (BL, blue light; RL, red light; and FRL, far-red light) and light intensity conditions (DG, dark grown; LL, low-light intensity; and HL, high-light intensity)."

For instance, the authors cite a variety of papers focusing on more metabolic or physiological read outs of how CAM plants respond to light quality or intensity; yet at a glance (and please correct me if I'm wrong) none of these cited papers is very comparable in species and experimental design to what was presented here. In as much, I found myself at a loss to basic questions like (A): does the blue/red light treatment disrupt signaling and CAM cycle integration enough that carbon fixation is substantially reduced over the time period (e.g. does one expect a mixture of _hunger_ and _light signalling_ response from the plants).
[Response]: We added the following text to the Discussion section (line 415-424):
"In comparison with white light, blue light repressed the expression of three genes (β-CA, PEPC and ALMT) involved in dark CO_2 fixation and malate transport as well as two genes (PPDK and PPDK-RP) in the light phase of CAM pathway at dusk (i.e., 2 h before the beginning of dark period) (Fig. 7a). Similarly, red light/far-red light repressed the expression of three genes (PEPC, MDH and ALMT) involved in dark CO_2 fixation and malate transport as well as one gene (PPDK) in the light phase of CAM pathway at dusk (i.e., 2 h before the beginning of dark period) (Fig. 7a). This monochrome light-induced gene repression was consistent with the much lower acid accumulation (i.e., dawn-dusk $\Delta H+$) under blue/red/far-red light conditions (Fig. 6b). These results indicate that blue/red/far-red light treatment interfere with the optimal performance of the CAM pathway in K. fedtschenkoi."

Or (B): is the high light stressing the plant (e.g. causing excess reactive oxygen species production and high non-photochemical quenching), or is it perhaps still within it's normal physiological range / not yet necessarily saturating the photosynthetic machinery?
[Response]: We added the following text to the Discussion section (line 404-414):
"We found that high level (1,000 μmol m-2 s-1) of white light increased expression of three dark CO_2 fixation genes (i.e., β-CA, PPCK, PEPC) and one malate transporter gene (ALMT) at dawn (i.e., 2 h before the beginning of light period) in comparison with intermediate level (440 μmol m-2 s-1) of white light (Fig. 7a). This increased transcript abundance of genes involved in dark CO_2 fixation and malate import into the vacuoles was consistent with the higher acid accumulation (i.e., dawn-dusk $\Delta H+$) under high light relative to intermediate light intensity (Fig. 6b). On the other hand, we found that high level of white light repressed expression of several CAM pathway genes (e.g., PEPC, MDH, PPDK, PPDK-RP) at dusk (i.e., 2 h before the beginning of dark period) in comparison with intermediate level of white light (Fig. 7a). These results suggest that the high-light treatment was still within the normal physiological range of the plant and not yet necessarily saturating the photosynthetic machinery."

I appreciate that the authors can neither know nor address the questions of each individual reader, but providing a few basics would be very helpful.

In particular, if the authors still have material in the freezer:
Measurements of titratable acidity and starch and sugar content, would provide a (comparatively) easy and reliable way to check the overall functioning of the CAM cycle.
[Response]: Yes, we have added the nocturnal malic and citric acid accumulation, soluble sugar content and starch content in new Figure 6 and the relevant description in Results and Discussion sections.

If the authors are able to reliably reproduce their setup:
Measurements on live plants of photosynthetic traits/activity (such as gas-exchange, Fv/Fm, NPQ), would help a lot in understanding what's ultimately active in the plant.
Where further analyses are unfeasible, it would help a lot if the authors would bring to bear more literature or background information (e.g. simply knowing the typical light intensity the plant experiences in the wild would go a ways towards helping answer question B, above).
[Response]: Thanks for your suggestion. Unfortunately, at the moment we do not have enough living plants for the photosynthetic traits' measurement. It will take at least another six months to prepare the materials. Although we cannot include living plant photosynthetic result in current study, we have added titratable acidity and sugar and starch results in the new Figure 6 to provide the physiological clues for understanding the association between genes and physiological traits. We believe it will not affect the

conclusion for this study. Thanks for your consideration!

2-
This dataset certainly provides intrinsic value to the scientific (and particularly the CAM) community. But the analyses they have performed on the data unfortunately strike me as primarily untargeted and thin. Don't get me wrong, I appreciate the data overview, and very much like seeing that the replicates cluster closely in the PCA, but there are a couple reasons (in my opinion) to provide some more targeted analyses on top.
First, the authors presumably generated this dataset in order to answer particular questions of interest to their lab. Perhaps they were unable to answer their questions (it happens) or perhaps the dataset was used to generate candidates which will be analyzed one by one for years down the line. Fair enough.
Second however, (and not unrelated to major point #1) anyone else considering working with this data probably wants to have some sort of general summary of what is happening in this dataset before they know if they could / should use it for their own research. In my experience this is true from people looking up their favorite gene of interest in an eFP browser to people looking at including the study in some large-scale meta analysis.
Understanding the data quality goes beyond a bit of clustering and co-expression.
In as much, I would highly encourage the authors to spend less of the paper talking about different clustering runs or numbers of differentially expressed genes and instead focus some on providing a functional summary of what is happening in the plants. In particular, the authors state that they provide "a novel genomics resource for investigating the molecular mechanism underlying the light regulation in CAM plants". So please, walk us through some examples:
- do we see the same pathways responding to the phytochromes as we would expect from other plants in red/blue light?
[Response]: According to your suggestions, we have added a new Figure 8 to indicate the expression patterns of photoreceptors in Kalanchoë leaves.

- do we rather see stress response or photosynthesis going up in high light?
[Response]: Based on the GO enrichment analysis of DEGs under high light (Figure 2), we didn’t find the stress response going up in high light. We also compared the expression of CAM-related photosynthetic genes in response to light intensity (Figure 6b), we found several genes were constitutively up-regulated with the increasing of the light intensity at dawn or dusk (such as MDH and ALMT). We have added the additional analysis (subnetwork of MDH in Figure 6c) and the relevant description in Section of Results and Discussion.

- dark grown plants show very few DEGs between Dusk/Dawn, is the clock still working properly or already strongly tapering?
[Response]: Most of the circadian rhythm-related genes such as CCA1, CRY2, ELF3/4, HY5, PRR7/9, RVE1/6/8 and TOC1 have disordered expression patterns between dawn and dusk under DG (Supplementary Fig. S7), indicating that circadian clock regulation is strongly repressed under DG.

These are just a few examples, I'd be happy to see anything the authors think is important in understanding the data and in making sure the plants are responding give or take as expected to the treatment.

However, considering the introduction and experimental design, I would strongly encourage the authors to consider making a graphic for what's known about molecular light response, not unlike what they did with the CAM pathway; and generally discuss if the observed expression here is in line with what is known from other plants/studies or not.
[Response]: According to your suggestions, we have added a new Figure 8 to indicate the expression patterns of photoreceptors in Kalanchoë leaf. We tried to find the similar expression dataset from other plant species, but we can only find a dataset of Arabidopsis which used seedlings treated 45 min and 4 h under BL, RL or FRL conditions. Concerning the differences of detected tissue and treatment time, we just included the 4h treatments data in Arabidopsis and compared it with our dusk data (10h treatment) in Kalanchoë. In addition, we analyzed the subnetworks of specific photoreceptors (Fig. 8c and d). The results indicate that conserved and novel regulatory mechanisms existed in circadian rhythm and light response in CAM plant Kalanchoë.

I'm sorry to brush aside the GO-enrichments and the bubble plots here but I'm looking for a summary, not a data-dump.
What the authors have provided in terms of functional analyses could be modified to make it more helpful.

I would need the enrichments of DEGs to be broken down by up/down regulated to make sense of them. I'd like to see terms like "response to endogenous stimulus" or "cellular processes" be pre-processed by the author's a little bit (if and where they are focused on in the prose) so the reader knows what major sub-terms or perhaps even what a few example genes are that are found in this term with this expression pattern. The reader can't readily connect the WGCNA clusters to an actual expression pattern from what I can see? So I really don't know what to make of these enrichments.
Is it necessary to put molecular function or cellular compartment into the same plots? Or could some of this be transferred to the supplement, so the rest could be larger and interpreted more readily?
[Response]: Yes, we have pointed out the key terms of the enrichment analysis in the results.
As well as all DEGs, we also performed the functional enrichment analysis of DEGs with increased or decreased relative transcript abundance patterns (Supplementary Figure S2 and Table S3).
According to your suggestion, we have added the expression patterns of each WGCNA clusters in new Figure 4, and have listed the GO enrichment of each cluster.
The full list of GO enrichment including biological process, molecular function and cellular compartment were listed as Supplementary Table S3 and S5. We just provided the GOslim terms (top levels of GO terms) in the main text figures.

3-
The clustering is a bit overwhelming. I counted _7_ clustering analyses, _5_ of which were directly or indirectly clustering by gene.
It's not that I've never been there, but using this many different rounds/forms of clustering is not making it easier for the reader to follow and I expect it is not making it easier for the authors to analyze the data themselves either.
Here I would suggest a few things:
First, at Figure 4b and figure 5c,d-
I do not see much added value in re-clustering the clusters. This is redundant and confusing for the reader. Particularly so for the re-clustering of the WGCNA clusters, which, as WGCNA implements a type of hierarchical clustering, _already_ have relative positioning.
[Response]: We have removed the original Figure 4b and Figure 5c,d according to your suggestion.

Second, even after removing the clusters of clusters, there would still be several occasions where the authors cluster the same basic information multiple times
- PCA & hierarchical clustering of the samples
- k-means, WGCNA hierarchical, and MDS clustering of the DGEs.
[Response]: We have removed those confusing cluster analyses. We only left PCA clustering to indicate the replicates cluster closely and WGCNA analysis to indicate the co-expression relationships.

I would encourage the authors to think carefully about the different methods and the characteristics / unique value of each and whether they really need (or even particularly talk about) so many different types.
My impression is that the authors could have presented the same essential "story" with only the WGCNA and PCA clustering, assuming at least, that they provided the expression pattern for the WGCNA clusters. But it is not unlikely that I missed something.
[Response]: Thanks for your comments. According to your suggestions, we removed those confusing cluster analyses. We only left PCA clustering to indicate the replicates cluster closely and WGCNA analysis to indicate the co-expression relationships. In the new Figure 5, we have added expression patterns of different modules in panel (b).

Inline / minor comments -
Title, pg 1, line 2-
As far as I have seen "expression Atlas" used before, it has normally referred to a wide survey of different tissues / developmental stages. I would recommend choosing a more precise term for clarity as the presented data is all from mature leaves.
[Response]: Yes, we have revised the title as "Light-responsive expression atlas reveals the effects of light quality and intensity in Kalanchoë fedtschenkoi, a plant with crassulacean acid metabolism"

page 4, line 89-
Is "metabolic synchronization" some sort of standard term? If not, it might be better to write something more descriptive.
[Response]: "metabolic synchronization" was changed to "metabolic changes"

page 5, line 115-

Ultimately the gigabytes of data produced is subject to the selected compression method. Personally, I prefer to know the number (and of course length) of reads/read pairs that were produced.
[Response]: Yes, we have added the detailed information including reads number, unique/multiple mapped reads and mapping rates in the updated Table S1, and revised the gigabytes to the number of read pairs. We added the following text to Line 137: "In total, we obtained 981 million read pairs (2×150 bp) with 287.78 Gb high quality data (Qscore≥25) from the 42 libraries, with an average size of ~23.4 million read pairs per library (Supplementary Table S1)."

page 5, line 121-
The use of the word 'cultured' here makes it sound like plants had been cultivated long-term under the different light conditions instead of simply having had two days to acclimate.
[Response]: We have revised it as "treated".

page 5, line 131-
Two things: first, "obvious" is not a sufficient reason to throw out "outliers".
Second, I must beg to differ that they were obvious at all, I had to stare at Supplemental Fig. 1b for a while to figure out what the authors were even seeing.
The authors' data is all for mature leaves, and excepting dark grown, it seems to all be quite healthy / active. Considering this, the clustering is very nice and actually separates samples better than I would have expected.
Please put the replicates back in for all the analyses!
[Response]: Yes. Thanks for your suggestion. We have put all the replicates back in the revised manuscript. All the analyses were re-analyzed based on the updated data.

page 6, line 146-
Are the few saved characters really worth it to write "C1_1" instead of giving the comparisons a name that doesn't require a reference figure (e.g. WL_Dawn-vs-Dusk)?
[Response]: Yes, we have replaced the comparison label according to your suggestion.

page 6, line 158-
I would discourage over interpretation of different numbers of DGEs. Yes, you can get fewer DGEs because a pair of samples were more similar to each other than the reference pair, but it can also simply be that there was a little more variance between the replicates.
[Response]: Yes, we have removed the excessive descriptions in this section.

page 7, line 166-
"may be" => "might" or "maybe"
[Response]: Revised as suggested.

page 7, line 176-
fix "(cite a figure or table here)"
[Response]: Fixed, with the relevant excessive description removed.

page 9, line 246-
The authors have projected thousands of DGEs from 39 clusters down to two dimensions, of course there is some overlap. No need to cluster again.
[Response]: Yes, we have removed the two dimensions MDS plot and the following clustering analysis.

page 30, Fig. 7-
The authors might be able to focus more on the expression patterns that are best proxies for the enzymes displayed if this figure more directly represented absolute expression. Either, focusing on the highly expressed paralog(s), summing up the expression of paralogs, or displaying some form of log(TPM) so that the lowly / highly expressed paralogs could be identified. If we're lucky and which paralogs are involved in the CAM cycle is established, the authors could focus only on these.
[Response]: Thanks for your suggestion, we have removed the heatmap in Figure 7 and use TPM value to generate the line plots in the revised Figure 7.

page 30, line 722-
fold change vs what? I presume it's the average, but this should be stated.
[Response]: we have removed the heatmap in Figure 7 and use TPM value to generate the line plots in the revised Figure 7.

-- End --

Close
