# Supplementary material for: Light-responsive expression atlas reveals the effects of light quality and intensity in Kalanchoë fedtschenkoi, a plant with crassulacean acid metabolism
Source: Gigascience. 2020 Mar 5;9(3):giaa018. doi: 10.1093/gigascience/giaa018 (PMC7058158; doi:10.1093/gigascience/giaa018)
Supplement: giaa018_Response_to_Reviewer_Comments_Revision_1 [file giaa018_response_to_reviewer_comments_revision_1.pdf]

Author's Response To Reviewer Comments

Close

Dear Dr. Zhang,
Your revised manuscript "Light-responsive expression atlas reveals the effects of light quality and intensity in Kalanchoë fedtschenkoi, a plant with crassulacean acid metabolism" (GIGA-D-19-00095R1) has been re-assessed by our reviewers. There are some further comments which we hope you can address in a second revised version.

In light of the latest reports (included below), we had to discuss two main issues before making a decision (and I apologize for the delay this has caused):

1) There is still some concern whether the paper presents substantial biological insights.
2) Reviewer 1 raised a methodological issue that became apparent in the revised version: that only one plant per replicate was used, rather than three per replicate, which in the reviewer's opinion is a better approach.

Regarding point 1, I somewhat agree, but both reviewers highlight the value of your data as a resource. We therefore decided to consider your paper for our "Data Note" section of the journal, where we highlight exceptional datasets and use cases thereof. Please let us know if you agree with this change of article type. You do not need to make any major changes to the manuscript itself for our "Data note" section, and you can fit your existing structure (headings etc) as sub-headings to the structure of the "Data Note" article type, with a few minor changes (see the "information for authors" on our homepage).
[Response:] Thanks for your comments. We agree to change the article type as “Data Note” without major changes to the current version of our manuscript.

Regarding point 2), we also discussed the issue with the other reviewer and don't see it as a critical flaw. However, the reviewers agree that pooling is generally a comparatively easy and cost effective method to reduce the variance, and ultimately increase the statistical power. Please discuss in the manuscript the potential advantage of the alternative "three and three" approach, and how your approach with only one plant per replicate may affect the results - i.e. more false negatives are expected with higher variance, etc.
[Response:] Actually, one individual for each biological replicate has been widely used in plant and animal studies. For example, researchers used one individual as a biological replicate for RNA-Seq and/or qPCR analyses in mouse (Burger et al. 2018), bird (Mead et al. 2017), soybean (Corcilius et al. 2017), etc. According to Bell (2016), biological replicates are biologically distinct samples, which show biological variation. An individual plant is a biologically distinct unit with a complete set of genetics and physiological characteristics, and therefore can be treated as a biological replicate. Pooling multiple plants into each biological replicate would be a good choice if one single plant cannot provide enough material for RNA isolation. Our strategy using one individual plant as a biological replicate can capture the variation in gene expression among individual plants, and therefore could potentially reduce the false positive rate of differential gene expression in comparison with biological replicates containing multiple plants.
According to your suggestion, we have added the relevant description in Discussion line 393: “We used one plant for each of the three biological replicates for RNA-Seq analysis, which could potentially reduce the false positive rate of differential gene expression in comparison with pooling multiple plants into each biological replicate.”

• Burger, Laura L., et al. "Identification of genes enriched in GnRH neurons by translating ribosome affinity purification and RNAseq in mice." Endocrinology 159.4 (2018): 1922-1940.
• Mead, Andrew F., et al. "Fundamental constraints in synchronous muscle limit superfast motor control in vertebrates." Elife 6 (2017): e29425.
• Corcilius, Leo, et al. "Arabinosylation modulates the growth-regulating activity of the peptide hormone CLE40a from soybean." Cell chemical biology 24.11 (2017): 1347-1355.
• Bell, Graham. "Replicates and repeats." BMC biology 14.1 (2016): 28.

Please also address all other comments of both reviewers (see the reports below).
=================

Reviewer #1: Summary of the key results
Overall evaluation
===========
As I stated in my previous revision, I think that the authors developed an useful resource for the scientific community working in the photosynthesis field, specially for CAM plants. The new version of the manuscript describes with more detail some of the pathways, but I still think that its hypothesis is not well developed. The new measures for leaf titratable acidity, soluble sugar and starch are interesting, but now that the authors added more details for the experimental design, it is worrying the low number of plants used per replicate (line 519: "three biological replicates of plants (each biological replicate was one independent plant)"). Personally I think that this type of experiments should use at least three independent plant per replicate (although five are highly recommended), with a total of at least nine plants per condition. The use of one plant per replicate may not be necessarily wrong, but it is not an strong design. Summarizing, I think that the manuscript is still an useful resource although it is descriptive (less than in the previous version). Discussion has been improved but some of the details about the experiment design show a low number of independent plants used in the experiment.

Major concerns
==========
* The proposed hypothesis behind the experiment is still not well developed. The manuscript is still mostly descriptive. I see the article as an useful resource to mine information as the author mentioned at the end of the "Background" section. Nevertheless, in my opinion, this manuscript still does not have a strong hypothesis behind the experiment. The authors wrote (line 116) "We hypothesize that both light intensity and light quality can influence the expression of CAM related metabolic and signaling genes in the obligate CAM species K. fedtschenkoi". Indeed this type of hypothesis are simple (light should influence a photosynthetic mechanism) and difficult to test (e.g. how you measure or test the influence?).
[Response:] Thanks for your comments. According to the editor’s suggestion, we would like to submit our manuscript as “Data Note”.

* Poor experimental design. One major issue that pop-up reading this revised version of the manuscript is that the authors used only one plant per replicate (three total plants per condition). Although the use of a low number of plants per replicates may not be wrong if a minimum number of three replicates are used, I think that represent a poor experimental design because it the source of variation using only one individual per replicate may drive to wrong interpretations or not solid enough results. It is recommended at least three replicates and at least three independent plants per replicate. Probably statisticians will argue that three plants per replicate is not solid either but I think that there is some kind of compromise in the community of what it is a good number of replicates and individuals per replicates and how many resources can be invested, and as I know it is three and three.
[Response:] Thanks for your comments.
Actually, one individual for each biological replicate has been widely used in plant and animal studies. For example, researchers used one individual as a biological replicate for RNA-Seq and/or qPCR analyses in mouse (Burger et al. 2018), bird (Mead et al. 2017), soybean (Corcilius et al. 2017), etc. According to Bell (2016), biological replicates are biologically distinct samples, which show biological variation. An individual plant is a biologically distinct unit with a complete set of genetics and physiological characteristics, and therefore can be treated as a biological replicate. Pooling multiple plants into each biological replicate would be a good choice if one single plant cannot provide enough material for RNA isolation. Our strategy using one individual plant as a biological replicate can capture the variation in gene expression among individual plants, and therefore could potentially reduce the false positive rate of differential gene expression in comparison with biological replicates containing multiple plants.

• Burger, Laura L., et al. "Identification of genes enriched in GnRH neurons by translating ribosome affinity purification and RNAseq in mice." Endocrinology 159.4 (2018): 1922-1940.
• Mead, Andrew F., et al. "Fundamental constraints in synchronous muscle limit superfast motor control in vertebrates." Elife 6 (2017): e29425.
• Corcilius, Leo, et al. "Arabinosylation modulates the growth-regulating activity of the peptide hormone CLE40a from soybean." Cell chemical biology 24.11 (2017): 1347-1355.
• Bell, Graham. "Replicates and repeats." BMC biology 14.1 (2016): 28.

Reviewer #2: The authors have made major improvements to the analyses and manuscript, and the resource they provide to the community is now much more approachable, and, inasmuch, more valuable than before.
I have only a few rather minor comments remaining:
- at, e.g., line 196, and Figure S2, Fig2 c-e:
Be consistent in how you refer to up-regulated and down-regulated genes. My personal suggestion would be to avoid terms with DEGs (e.g. "increased abundance Differentially Expressed Genes" or "up DEGs") and simply call them "up-regulated genes".
The same idea goes for down-regulated genes.
[Response:] According to your suggestion, we have revised the relevant description to “up-regulated genes” and “down-regulated genes” throughout the manuscript.

- Fig 2g:
This has improved, but I am unfortunately still confused about this figure.
First aesthetics:
The labels are very small and too close to each other to be easily read.
Can MF and CC be moved to the supplemental?
Or can the specific comparisons be split off?
Or can this have it's own figure / somehow be larger?
[Response:] According to your suggestion, we have moved MF and CC to the supplementary Figure S3.

Second content:
I know it's probably somewhere in the paper, but I can't find a clear description of what "specific" DEGs are in the figure or the figure legend.
Clearly the specific "WL_Dusk_vs_Dawn" out of the light quality comparisons (common 1) does not match "WL_Dusk_vs_Dawn" out of the light intensity comparisons (common 2).
But I can't tell if the former is just the 1517 DEGs that are significantly different (dusk vs dawn) _only_ under white light, or if it is the (6412 - 2480) DEGs that are significantly different under white light, but not for all light quality dusk vs dawn comparisons.
Or perhaps it's something else entirely?
Similarly, I'm left unsure if the "common" category refers to DEGs shared by _all_ the comparisons in a given set, or if they are shared by any two-or more of the comparisons in a given set.
[Response:] To clarify the “common” and “specific” DEGs, we added the description in Figure 2 legend: (g) Gene ontology (GO) enrichment of common DEGs shared by different comparisons in (f) Venn diagrams (DEGs shared by all the comparisons in a given set) or specific DEGs (genes only differentially expressed in one of the overlapped comparisons). Biological process (BP). Detailed enrichment of molecular function (MF) and cellular component (CC) was shown in Supplementary Figure S3. GOslim terms were shown in here.

Further, still taking light quality (common 1) as an example, I'm confused about why only some of the comparisons' are reported here; why only WL and RL, why not BL or FRL?
[Response:] There is no significant enriched GO terms for those comparisons, which are not included in the figure. In updated Figure 2g and Figure S3, we have added all the comparisons.

Finally, one small detail: "Enrichment factor" not "Rich factor" (fix throughout the paper please)
[Response:] We have revised it as “Enrichment factor” throughout the manuscript, including Figure 2g, Figure 4c, Table S3, and Table S5.

If a full explanation is hard to fit in the figure legend, I'd be happy with including a reference to a sub-header in the methods or perhaps supplemental where all the details can be found. As long as the reader doesn't have to search.
[Response:] To clarify the “common” and “specific” DEGs, we added the description in Figure 2 legend: (g) Gene ontology (GO) enrichment of common DEGs shared by different comparisons in (f) Venn diagrams (DEGs shared by all the comparisons in a given set) or specific DEGs (genes only differentially expressed in one of the overlapped comparisons). Biological process (BP). Detailed enrichment of molecular function (MF) and cellular component (CC) was shown in Supplementary Figure S3. GOslim terms were shown in here.

- at 338:
I don't think that the Arabidopsis efp browser is the _only_ publicly available light-quality expression data. It could well be the most comparable data and it's totally reasonable to use the data, I just wouldn't use this particular sentence.

[Response:] We have removed this particular sentence in the revised manuscript.

-- End --

Close
