# Supplementary material for: Light-responsive expression atlas reveals the effects of light quality and intensity in Kalanchoë fedtschenkoi, a plant with crassulacean acid metabolism
Source: Gigascience. 2020 Mar 5;9(3):giaa018. doi: 10.1093/gigascience/giaa018 (PMC7058158; doi:10.1093/gigascience/giaa018)
Supplement: giaa018_Reviewer_1_Report_Original_Submission — Aureliano Bombarely, Ph.D. -- 4/15/2019 Reviewed [file giaa018_reviewer_1_report_original_submission.pdf]

Reviewer Report

Title: Light-responsive expression atlas reveals the effects of light quality and intensity in KalanchoÃ« fedtschenkoi, a plant with crassulacean acid metabolism

Version: Original Submission **Date:** 4/15/2019

Reviewer name: Aureliano Bombarely, Ph.D.

Reviewer Comments to Author:

Summary of the key results
=================
The manuscript titled "Expression atlas and co-expression network reveal effects of light quality and intensity in KalanchoeÌˆ fedtschenkoi, a plant with crassulacean acid metabolism" describes the transcriptomic characterization of the K. fedtschenkoi leaves under different light spectrums (blue, red, infrared and white), intensities (0, 150, 250 and 1000 Î¼mol m-2 s-1) and time points associated to the circadian clock (2h after dusk and after dawn). The authors analyze the differential expression between different light quality conditions (light spectrums and intensity) and time points. Additionally the authors performed a clustering and co-expression network analysis associated with gene ontology terms. Finally to increase the usability of the data for the scientific community, the authors implemented a gene expression graphical interface using the eFP browser at the BAR.
Overall evaluation
===========
This manuscript presents an interesting and useful resource for the plant scientific community, specially the one that it is working in photosynthesis and CAM metabolism. Nevertheless, I found that the manuscript is mostly descriptive without a clear hypothesis and a discussion of the results in agreement with that hypothesis. Probably there is more than enough data to discuss specific aspects of the experiment (e.g. how the light spectrum activate some pathways but not others, why the transcriptomic response is higher with low and high light...). Overall, I think that the authors develop an useful resource but at the same time, I think that the presentation of the results and the discussion should be improved with a stronger biological support.
Major concerns
==========
* Lack of a clear hypothesis behind the experiment. The manuscript is mostly descriptive. I see the article as an useful resource to mine information as the author mentioned at the end of the "Background" section. Nevertheless, in my opinion, this manuscript does not have a clear hypothesis behind the experiment or at least it is not clear from the narrative of the results and the discussion.
* Poor result discussion. The discussion does not contain any element related with the light quality being in more or the cases a comparison which what is known in Arabidopsis rather than a discussion that drives to new hypothesis about the regulatory cascades of the CAM.
Minor concerns
=========

* Language polishing: Although the manuscript is easy to read and there is not major mistakes that I can detect, probably a polishing in the writing will help to do the manuscript more fluid. For example, there are long sentences that written in a different way will improve the readability (e.g. Abstract lines 37-40. Background lines 59-62…).
* No summary of number of sequenced and mapped reads per replicate. The authors should provide this information in the supplementary data to have a better evaluation of the process (e.g. if only half of the reads map to the genome is an indication of a problem with the samples).
* Section "Differentially expressed genes (DEGs) regulated by light quality and light intensity": This result section is already summarized in the figure 2 and 3. It is interesting to know if the transcriptomic response is higher depending of the light spectrum, quality and/or time point, but I think that the continuous description of what it can be observed in the figures 2 and 3 blurs the result.
* Section "Predicted function of DEGs": I think that it could be convenient to complement the GSEA with a metabolic pathway analysis using tools such as Mapman (https://mapman.gabipd.org/home) to have a better idea of the members involve in the response to those stimuli rather than a general view. It makes sense that "photosynthesis" is an enriched term comparing dawn and dusk, but probably it could be more interesting to know specifically which members of the photosynthetic apparatus are involved.
* Section "Clusters of DEGs": As in previous sections, the authors describe the composition of the clusters, but not the meaning of those in relation with the biological experiment. For example, "genes from clusters 3 and 11 show an strong response to the circadian clock" could be more informative than "clusters 3 and 11 are composed by genes with DE between dawn and dusk".
* Lack of details in the Material and Methods: How many plants per biological replicate did the authors used? How the RNA-Seq libraries were prepared? Did the authors perform a size selection of the fragments? Where the samples were sequenced? Which version was used for TopHat2 and FeatureCounts? (other software versions are also missing).

Discretionary revisions
==============

* Background, line 56: Parenthesis is opened but not close.
* Background, line 92: "However, gene regulation at the transcriptional level in CAM plants in response to various spectral light qualities and intensity has not been reported yet". I am not sure that "reported" is the most adequate verb here. I will suggest "analyzed", "studied" or "investigated". "Report" is more concrete and linked to a result or an observation.
* Data description, line 116: "we obtained ~138 Gb of high-quality data". High-quality data in this context is an unprecise term. Please, be more precise (e.g. qscore > 30…).
* Analysis, line 176: Probably the authors forget to replace the note "cite a figure or table here" by a real figure.
* Analysis, line 217: Why 20 clusters?
* Discussion, line 335: "carbohydrate metabolic" should be "carbohydrate metabolism".
* Figure 2: Abbreviations for each conditions should be described in the legend (e.g. LL=Low Light…).

Level of Interest

Please indicate how interesting you found the manuscript: Choose an item.

Quality of Written English

Please indicate the quality of language in the manuscript: Choose an item.

Declaration of Competing Interests

Please complete a declaration of competing interests, considering the following questions:

- Have you in the past five years received reimbursements, fees, funding, or salary from an organisation that may in any way gain or lose financially from the publication of this manuscript, either now or in the future?
- Do you hold any stocks or shares in an organisation that may in any way gain or lose financially from the publication of this manuscript, either now or in the future?
- Do you hold or are you currently applying for any patents relating to the content of the manuscript?
- Have you received reimbursements, fees, funding, or salary from an organization that holds or has applied for patents relating to the content of the manuscript?
- Do you have any other financial competing interests?
- Do you have any non-financial competing interests in relation to this paper?

If you can answer no to all of the above, write 'I declare that I have no competing interests' below. If your reply is yes to any, please give details below.

I declare that I have no competing interests

I agree to the open peer review policy of the journal. I understand that my name will be included on my report to the authors and, if the manuscript is accepted for publication, my named report including any attachments I upload will be posted on the website along with the authors' responses. I agree for my report to be made available under an Open Access Creative Commons CC-BY license (http://creativecommons.org/licenses/by/4.0/). I understand that any comments which I do not wish to be included in my named report can be included as confidential comments to the editors, which will not be published.

Choose an item.

To further support our reviewers, we have joined with Publons, where you can gain additional credit to further highlight your hard work (see: https://publons.com/journal/530/gigascience). On publication of this paper, your review will be automatically added to Publons, you can then choose whether or not to claim your Publons credit. I understand this statement.

Yes Choose an item.
