# Supplementary material for: Light-responsive expression atlas reveals the effects of light quality and intensity in Kalanchoë fedtschenkoi, a plant with crassulacean acid metabolism
Source: Gigascience. 2020 Mar 5;9(3):giaa018. doi: 10.1093/gigascience/giaa018 (PMC7058158; doi:10.1093/gigascience/giaa018)
Supplement: giaa018_Reviewer_1_Report_Revision_1 — Aureliano Bombarely, Ph.D. -- 11/24/2019 Reviewed [file giaa018_reviewer_1_report_revision_1.pdf]

Reviewer Report

Title: Light-responsive expression atlas reveals the effects of light quality and intensity in KalanchoÃ« fedtschenkoi, a plant with crassulacean acid metabolism

Version: Revision 1 **Date:** 11/24/2019

Reviewer name: Aureliano Bombarely, Ph.D.

Reviewer Comments to Author:

Summary of the key results
=================
See the previous revision
Overall evaluation
===========
As I stated in my previous revision, I think that the authors developed an useful resource for the scientific community working in the photosynthesis field, specially for CAM plants. The new version of the manuscript describes with more detail some of the pathways, but I still think that its hypothesis is not well developed. The new measures for leaf titratable acidity, soluble sugar and starch are interesting, but now that the authors added more details for the experimental design, it is worrying the low number of plants used per replicate (line 519: "three biological replicates of plants (each biological replicate was one independent plant)"). Personally I think that this type of experiments should use at least three independent plant per replicate (although five are highly recommended), with a total of at least nine plants per condition. The use of one plant per replicate may not be necessarily wrong, but it is not an strong design. Summarizing, I think that the manuscript is still an useful resource although it is descriptive (less than in the previous version). Discussion has been improved but some of the details about the experiment design show a low number of independent plants used in the experiment.
Major concerns
==========
* The proposed hypothesis behind the experiment is still not well developed. The manuscript is still mostly descriptive. I see the article as an useful resource to mine information as the author mentioned at the end of the "Background" section. Nevertheless, in my opinion, this manuscript still does not have a strong hypothesis behind the experiment. The authors wrote (line 116) "We hypothesize that both light intensity and light quality can influence the expression of CAM related metabolic and signaling genes in the obligate CAM species K. fedtschenkoi". Indeed this type of hypothesis are simple (light should influence a photosynthetic mechanism) and difficult to test (e.g. how you measure or test the influence?).
* Poor experimental design. One major issue that pop-up reading this revised version of the manuscript is that the authors used only one plant per replicate (three total plants per condition). Although the use of a low number of plants per replicates may not be wrong if a minimum number of three replicates are used, I think that represent a poor experimental design because it the source of variation using only one individual per replicate may drive to wrong interpretations or not solid enough

results. It is recommended at least three replicates and at least three independent plants per replicate. Probably statisticians will argue that three plants per replicate is not solid either but I think that there is some kind of compromise in the community of what it is a good number of replicates and individuals per replicates and how many resources can be invested, and as I know it is three and three.

Level of Interest

Please indicate how interesting you found the manuscript: Choose an item.

Quality of Written English

Please indicate the quality of language in the manuscript: Choose an item.

Declaration of Competing Interests

Please complete a declaration of competing interests, considering the following questions:

- Have you in the past five years received reimbursements, fees, funding, or salary from an organisation that may in any way gain or lose financially from the publication of this manuscript, either now or in the future?
- Do you hold any stocks or shares in an organisation that may in any way gain or lose financially from the publication of this manuscript, either now or in the future?
- Do you hold or are you currently applying for any patents relating to the content of the manuscript?
- Have you received reimbursements, fees, funding, or salary from an organization that holds or has applied for patents relating to the content of the manuscript?
- Do you have any other financial competing interests?
- Do you have any non-financial competing interests in relation to this paper?

If you can answer no to all of the above, write 'I declare that I have no competing interests' below. If your reply is yes to any, please give details below.

I declare that I have no competing interests

I agree to the open peer review policy of the journal. I understand that my name will be included on my report to the authors and, if the manuscript is accepted for publication, my named report including any attachments I upload will be posted on the website along with the authors' responses. I agree for my report to be made available under an Open Access Creative Commons CC-BY license (http://creativecommons.org/licenses/by/4.0/). I understand that any comments which I do not wish to be included in my named report can be included as confidential comments to the editors, which will not be published.

Choose an item.

To further support our reviewers, we have joined with Publons, where you can gain additional credit to further highlight your hard work (see: https://publons.com/journal/530/gigascience). On publication of this paper, your review will be automatically added to Publons, you can then choose whether or not to claim your Publons credit. I understand this statement.

Yes Choose an item.
