# Supplementary material for: Light-responsive expression atlas reveals the effects of light quality and intensity in Kalanchoë fedtschenkoi, a plant with crassulacean acid metabolism
Source: Gigascience. 2020 Mar 5;9(3):giaa018. doi: 10.1093/gigascience/giaa018 (PMC7058158; doi:10.1093/gigascience/giaa018)
Supplement: giaa018_Reviewer_2_Report_Original_Submission — Alisandra Denton -- 4/25/2019 Reviewed [file giaa018_reviewer_2_report_original_submission.pdf]

Reviewer Report

Title: Light-responsive expression atlas reveals the effects of light quality and intensity in KalanchoÃ« fedtschenkoi, a plant with crassulacean acid metabolism

Version: Original Submission **Date:** 4/25/2019

Reviewer name: Alisandra Denton

Reviewer Comments to Author:

The Authors present their comparative transcriptomics work
examining the effect of different light regimes on the new model CAM plant,
KalanchoÃ« fedtschenkoi. The authors perform some large scale typical
analyses including differential gene expression and clustering,
which indicate the data is of workable quality. Finally the authors
touch briefly on a few co-expressed known and novel regulatory factors
that may-be of interest in understanding how light signals are integrated
into regulating CAM photosynthesis.
Major comments:
1-
The dataset that the authors have generated appears to be
of good quality and I anticipate that it will be very useful
for the CAM research community.
I also appreciate that both the raw data and the included eFP
browser are already available. That said, if the authors are still
able to provide some contextual data; I think this might make it
much easier to interpret the transcriptomics data.
For instance, the authors cite a variety of papers focusing on more
metabolic or physiological read outs of how CAM plants respond
to light quality or intensity; yet at a glance (and please correct me if I'm wrong)
none of these cited papers is very comparable in species and experimental
design to what was presented here. In as much, I found myself at a
loss to basic questions like (A): does the blue/red light treatment
disrupt signaling and CAM cycle integration enough that carbon fixation
is substantially reduced over the time period (e.g. does one expect
a mixture of _hunger_ and _light signalling_ response from the plants).
Or (B): is the high light stressing the plant (e.g. causing excess
reactive oxygen species production and high non-photochemical quenching),
or is it perhaps still within it's normal physiological range / not
yet necessarily saturating the photosynthetic machinery?
I appreciate that the authors can neither know nor address the
questions of each individual reader, but providing a few basics

would be very helpful.
In particular, if the authors still have material in the freezer:
Measurements of titratable acidity and starch and sugar content,
would provide a (comparatively) easy and reliable way to check the
overall functioning of the CAM cycle.
If the authors are able to reliably reproduce their setup:
Measurements on live plants of photosynthetic traits/activity
(such as gas-exchange, Fv/Fm, NPQ), would help a lot in understanding
what's ultimately active in the plant.
Where further analyses are unfeasible, it would help a lot if the
authors would bring to bear more literature or background information
(e.g. simply knowing the typical light intensity the plant experiences
in the wild would go a ways towards helping answer question B, above).
2-
This dataset certainly provides intrinsic value to the scientific
(and particularly the CAM) community. But the analyses they
have performed on the data unfortunately strike me as primarily untargeted and
thin.
Don't get me wrong, I appreciate the data overview, and very much like seeing that
the replicates cluster closely in the PCA, but there are a couple reasons (in my opinion)
to provide some more targeted analyses on top.
First, the authors presumably generated this dataset in order to answer
particular questions of interest to their lab. Perhaps they were unable
to answer their questions (it happens) or perhaps the dataset was used
to generate candidates which will be analyzed one by one for years down the
line. Fair enough.
Second however, (and not unrelated to major point #1) anyone else considering
working with this data probably wants to have some sort of general summary
of what is happening in this dataset before they know if they could / should
use it for their own research. In my experience this is true from people
looking up their favorite gene of interest in an eFP browser
to people looking at including the study in some large-scale meta analysis.
Understanding the data quality goes beyond a bit of clustering and co-expression.
In as much, I would highly encourage the authors to spend less of the paper
talking about different clustering runs or numbers of differentially
expressed genes and instead focus some on providing a functional summary
of what is happening in the plants. In particular, the authors state that
they provide "a novel genomics resource for investigating the molecular
mechanism underlying the light regulation in CAM plants". So please,
walk us through some examples:
- do we see the same pathways responding to the phytochromes as we would
expect from other plants in red/blue light?

- do we rather see stress response or photosynthesis going up in high light?
- dark grown plants show very few DEGs between Dusk/Dawn, is the clock still
 working properly or already strongly tapering?

These are just a few examples, I'd be happy to see anything the authors
think is important in understanding the data and in making sure the plants
are responding give or take as expected to the treatment.
However, considering the introduction and experimental design,
I would strongly encourage the authors to consider making a graphic for
what's known about molecular light response, not unlike what they did
with the CAM pathway; and generally discuss if the observed expression
here is in line with what is known from other plants/studies or not.
I'm sorry to brush aside the GO-enrichments and the bubble plots here
but I'm looking for a summary, not a data-dump.
What the authors have provided in terms of functional analyses
could be modified to make it more helpful.
I would need the enrichments of DEGs to be broken down by up/down regulated to make sense of
them.
I'd like to see terms like "response to endogenous stimulus" or
"cellular processes" be pre-processed by the author's a little bit
(if and where they are focused on in the prose)
so the reader knows what major sub-terms or perhaps even what a few example
genes are that are found in this term with this expression pattern. The reader can't readily
connect the WGCNA clusters to an actual expression pattern from what I
can see? So I really don't know what to make of these enrichments.
Is it necessary to put molecular function or cellular compartment into
the same plots? Or could some of this be transferred to the supplement,
so the rest could be larger and interpreted more readily?

3-

The clustering is a bit overwhelming. I counted _7_
clustering analyses, _5_ of which were directly or indirectly
clustering by gene.
It's not that I've never been there, but using this many different rounds/forms
of clustering is not making it easier for the reader to follow
and I expect it is not making it easier for the authors to analyze the
data themselves either.
Here I would suggest a few things:
First, at Figure 4b and figure 5c,d-
I do not see much added value in re-clustering the clusters. This is
redundant and confusing for the reader. Particularly so for the
re-clustering of the WGCNA clusters, which, as WGCNA implements a type
of hierarchical clustering, _already_ have relative positioning.
Second, even after removing the clusters of clusters, there would still

be several occasions where the authors cluster the same basic information
multiple times
- PCA & hierarchical clustering of the samples
- k-means, WGCNA hierarchical, and MDS clustering of the DGEs.

I would encourage the authors to think carefully about the different
methods and the characteristics / unique value of each and whether they really
need (or even particularly talk about) so many different types.
My impression is that the authors could have presented the same essential
"story" with only the WGCNA and PCA clustering, assuming at least, that
they provided the expression pattern for the WGCNA clusters. But it is not
unlikely that I missed something.
Inline / minor comments -
Title, pg 1, line 2-
As far as I have seen "expression Atlas" used before, it has normally referred
to a wide survey of different tissues / developmental stages. I would recommend
choosing a more precise term for clarity as the presented data is all from
mature leaves.
page 4, line 89-
Is "metabolic synchronization" some sort of standard term? If not, it might
be better to write something more descriptive.
page 5, line 115-
Ultimately the gigabytes of data produced is subject to the selected
compression method. Personally, I prefer to know the number (and of course length)
of reads/read pairs that were produced.
page 5, line 121-
The use of the word 'cultured' here makes it sound like plants had been
cultivated long-term under the different light conditions instead of simply
having had two days to acclimate.
page 5, line 131-
Two things: first, "obvious" is not a sufficient reason to throw out "outliers".
Second, I must beg to differ that they were obvious at all, I had to stare at
Supplemental Fig. 1b for a while to figure out what the authors were even seeing.
The authors' data is all for mature leaves, and excepting dark grown,
it seems to all be quite healthy / active. Considering this, the clustering is
very nice and actually separates samples better than I would have expected.
Please put the replicates back in for all the analyses!
page 6, line 146-
Are the few saved characters really worth it to write "C1_1" instead
of giving the comparisons a name that doesn't require a reference
figure (e.g. WL_Dawn-vs-Dusk)?
page 6, line 158-
I would discourage over interpretation of different numbers of

DGEs. Yes, you can get fewer DGEs because a pair of samples were
more similar to each other than the reference pair, but it can also simply be that
there was a little more variance between the replicates.
page 7, line 166-
"may be" => "might" or "maybe"
page 7, line 176-
fix "(cite a figure or table here)"
page 9, line 246-
The authors have projected thousands of DGEs from 39 clusters down
to two dimensions, of course there is some overlap. No need to
cluster again.
page 30, Fig. 7-
The authors might be able to focus more on the expression patterns that
are best proxies for the enzymes displayed if this figure more directly
represented absolute expression. Either, focusing on the highly expressed
paralog(s), summing up the expression of paralogs, or displaying some
form of log(TPM) so that the lowly / highly expressed paralogs could be
identified. If we're lucky and which paralogs are involved in the CAM cycle
is established, the authors could focus only on these.
page 30, line 722-
fold change vs what? I presume it's the average, but this should be stated.

Level of Interest

Please indicate how interesting you found the manuscript: Choose an item.

Quality of Written English

Please indicate the quality of language in the manuscript: Choose an item.

Declaration of Competing Interests

Please complete a declaration of competing interests, considering the following questions:

- Have you in the past five years received reimbursements, fees, funding, or salary from an organisation that may in any way gain or lose financially from the publication of this manuscript, either now or in the future?
- Do you hold any stocks or shares in an organisation that may in any way gain or lose financially from the publication of this manuscript, either now or in the future?
- Do you hold or are you currently applying for any patents relating to the content of the manuscript?

- Have you received reimbursements, fees, funding, or salary from an organization that holds or has applied for patents relating to the content of the manuscript?
- Do you have any other financial competing interests?
- Do you have any non-financial competing interests in relation to this paper?

If you can answer no to all of the above, write 'I declare that I have no competing interests' below. If your reply is yes to any, please give details below.

I declare that I have no competing interests

I agree to the open peer review policy of the journal. I understand that my name will be included on my report to the authors and, if the manuscript is accepted for publication, my named report including any attachments I upload will be posted on the website along with the authors' responses. I agree for my report to be made available under an Open Access Creative Commons CC-BY license (http://creativecommons.org/licenses/by/4.0/). I understand that any comments which I do not wish to be included in my named report can be included as confidential comments to the editors, which will not be published.

Choose an item.

To further support our reviewers, we have joined with Publons, where you can gain additional credit to further highlight your hard work (see: https://publons.com/journal/530/gigascience). On publication of this paper, your review will be automatically added to Publons, you can then choose whether or not to claim your Publons credit. I understand this statement.

Yes Choose an item.
