# Supplementary material for: Light-responsive expression atlas reveals the effects of light quality and intensity in Kalanchoë fedtschenkoi, a plant with crassulacean acid metabolism
Source: Gigascience. 2020 Mar 5;9(3):giaa018. doi: 10.1093/gigascience/giaa018 (PMC7058158; doi:10.1093/gigascience/giaa018)
Supplement: giaa018_Reviewer_2_Report_Revision_1 — Alisandra Denton -- 11/22/2019 Reviewed [file giaa018_reviewer_2_report_revision_1.pdf]

**Reviewer Report**

**Title: Light-responsive expression atlas reveals the effects of light quality and intensity in KalanchoÃ« fedtschenkoi, a plant with crassulacean acid metabolism**

**Version: Revision 1** **Date:** 11/22/2019

**Reviewer name: Alisandra Denton**

**Reviewer Comments to Author:**

The authors have made major improvements to the analyses and manuscript, and the resource they provide to the community is now much more approachable, and, inasmuch, more valuable than before.
I have only a few rather minor comments remaining:
- at, e.g., line 196, and Figure S2, Fig2 c-e:
Be consistent in how you refer to up-regulated and down-regulated genes. My personal suggestion would be to avoid terms with DEGs (e.g. "increased abundance Differentially Expressed Genes" or "up DEGs") and simply call them "up-regulated genes".
The same idea goes for down-regulated genes.
- Fig 2g:
This has improved, but I am unfortunately still confused about this figure.
First aesthetics:
The labels are very small and too close to each other to be easily read.
Can MF and CC be moved to the supplemental?
Or can the specific comparisons be split off?
Or can this have it's own figure / somehow be larger?
Second content:
I know it's probably somewhere in the paper, but I can't find a clear description of what "specific" DEGs are in the figure or the figure legend.
Clearly the specific "WL_Dusk_vs_Dawn" out of the light quality comparisons (common 1) does not match "WL_Dusk_vs_Dawn" out of the light intensity comparisons (common 2).
But I can't tell if the former is just the 1517 DEGs that are significantly different (dusk vs dawn) _only_ under white light, or if it is the (6412 - 2480) DEGs that are significantly different under white light, but not for all light quality dusk vs dawn comparisons.
Or perhaps it's something else entirely?
Similarly, I'm left unsure if the "common" category refers to DEGs shared by _all_ the comparisons in a given set, or if they are shared by any two-or more of the comparisons in a given set.
Further, still taking light quality (common 1) as an example, I'm confused about why only some of the comparisons' are reported here; why only WL and RL, why not BL or FRL?
Finally, one small detail: "Enrichment factor" not "Rich factor" (fix throughout the paper please)

If a full explanation is hard to fit in the figure legend, I'd be happy with including a
reference to a sub-header in the methods or perhaps supplemental where all the details can
be found. As long as the reader doesn't have to search.
- at 338:
I don't think that the Arabidopsis efp browser is the _only_ publicly available
light-quality expression data. It could well be the most comparable data and it's totally
reasonable to use the data, I just wouldn't use this particular sentence.

**Level of Interest**

Please indicate how interesting you found the manuscript: Choose an item.

**Quality of Written English**

Please indicate the quality of language in the manuscript: Choose an item.

**Declaration of Competing Interests**

Please complete a declaration of competing interests, considering the following questions:

- Have you in the past five years received reimbursements, fees, funding, or salary from an organisation that may in any way gain or lose financially from the publication of this manuscript, either now or in the future?
- Do you hold any stocks or shares in an organisation that may in any way gain or lose financially from the publication of this manuscript, either now or in the future?
- Do you hold or are you currently applying for any patents relating to the content of the manuscript?
- Have you received reimbursements, fees, funding, or salary from an organization that holds or has applied for patents relating to the content of the manuscript?
- Do you have any other financial competing interests?
- Do you have any non-financial competing interests in relation to this paper?

If you can answer no to all of the above, write 'I declare that I have no competing interests' below. If your reply is yes to any, please give details below.

I declare that I have no competing interests

I agree to the open peer review policy of the journal. I understand that my name will be included on my report to the authors and, if the manuscript is accepted for publication, my named report including any attachments I upload will be posted on the website along with the authors' responses. I agree for my report to be made available under an Open Access Creative Commons CC-BY license

(http://creativecommons.org/licenses/by/4.0/). I understand that any comments which I do not wish to be included in my named report can be included as confidential comments to the editors, which will not be published.

Choose an item.

To further support our reviewers, we have joined with Publons, where you can gain additional credit to further highlight your hard work (see: https://publons.com/journal/530/gigascience). On publication of this paper, your review will be automatically added to Publons, you can then choose whether or not to claim your Publons credit. I understand this statement.

Yes Choose an item.
